# Quantification and physiological significance of the rightward shift of the V-slope during incremental cardiopulmonary exercise testing

**DOI:** 10.1186/s13102-017-0073-1

**Published:** 2017-04-20

**Authors:** Hirotaka Nishijima, Kazuo Kondo, Kazuya Yonezawa, Hiroki Hashimoto, Masayuki Sakurai

**Affiliations:** 1 Cardiology, Hokko Memorial Hospital, 1-6 Kita-27 Higashi-8, Higashiku, Sapporo, 065-0027 Japan; 2 Cardiac Rehabilitation, Hokko Memorial Hospital, 1-6 Kita-27 Higashi-8, Higashiku, Sapporo, 065-0027 Japan; 3 Department of Clinical Research, National Hospital Organization Hakodate Hospital, 18-16 Kawahara-cho, Hakodate, 041-8512 Japan; 4 Rehabilitation, Histujigaoka Hospital, 1-10 Aoba-cho 3-Chome, Atsubetsu-ku, Sapporo, 004-0021 Japan; 5 Current address: 2-5-16 Sakaigawa, Chuoku, Sapporo, 064-0943 Japan

**Keywords:** Exercise tolerance, Ventilatory anaerobic threshold, CO_2_ storage

## Abstract

**Background:**

Ventilatory anaerobic threshold (VAT) is frequently used as a measure of exercise tolerance, with the V-slope method being the standard; however, this needs to be visually determined. Over the years, we have observed that the V-slope itself often appears to shift rightward before the appearance of the VAT (RtShift: rightward shift of V-slope). This phenomenon has long been known to occur during the first 1–2 min of steady-state exercise and disappears thereafter; it is attributed to CO_2_ storage, presumably in active muscle. However, during incremental exercise, we have observed that the RtShift persists; furthermore, it seems to be related to the level of VAT. Therefore, we attempted to objectively quantify the RtShift, and to confirm its relationship to an index of exercise tolerance (VAT).

**Methods:**

This study was based on a retrospective analysis of data from 100 cardiopulmonary ramp exercise tests (submaximal) performed by patients with cardiac disease. VAT was determined with the visual V-slope method. The horizontal distances between the diagonal *R* = 1 line and each data point on the V-slope plot to the right of *R* = 1 were measured; the average of these measurements was used as an objectively determined estimate of RtShift.

**Results:**

The predominant portion of RtShift occurred earlier than VAT. The mean RtShift was 33.9 ± 25.0 mL⋅min^−1^ VO_2_, whereas the mean VAT was 635 ± 220 mL⋅min^−1^. RtShift positively correlated with VAT (*r* = 718, *p* < 0.001), confirming previous visual observations. It also significantly correlated with ΔVO_2_/Δwork rate, a marker of oxygen uptake efficiency (*r* = 0.531, *p* < 0.001).

**Conclusions:**

We identified that among patients with cardiac disease, V-slope is shifted rightward to varying degrees. The objectively quantified rightward shift of V-slope is significantly correlated with an index of exercise tolerance (VAT). Furthermore, it appears to occur at even lower work rates. This may offer a new objective means of estimating exercise tolerance; however, its exact biological basis still needs to be elucidated.

**Electronic supplementary material:**

The online version of this article (doi:10.1186/s13102-017-0073-1) contains supplementary material, which is available to authorized users.

## Background

Ventilatory anaerobic threshold (VAT, or anaerobic threshold, AT) has been widely used as an index of exercise tolerance, primarily because it does not require maximal exercise [1, 2]. It is also recommended as an indicator of the optimal exercise training intensity during cardiac rehabilitation [3]. Among the methods for determining VAT, the V-slope method is considered to be the most basic; it directly assesses the relationship between VO_2_ and “excess CO_2_,” which is presumed to be derived from increased blood lactate levels [1]. It detects a breakpoint on the V-slope plotted on the x: VO_2_ versus y: VCO_2_ coordinates. The determination of the breakpoint (VAT), however, must be made visually, therefore making this parameter primarily a subjective measurement.

While using the V-slope method for determining VAT during routine cardiopulmonary incremental exercise tests (CPX) over a period of many years, we have found that the position of the V-slope itself is, from the early exercise stage, often shifted rightward to varying degrees from the reference diagonal line of the respiratory gas exchange ratio (R) of 1.0 in patients with cardiac disease as well as in normal subjects (Additional file 1: Figure S1). It also manifests itself as an initial drop in R. Since the 1960s, this phenomenon has been known to occur primarily in normal subjects during the first 1–2 min of steady-state exercise and disappears thereafter; it has been attributed to CO_2_ storage, presumably in active muscle [4–7]. However, we have noted that it also appears to occur during incremental exercise. We have also observed that the higher the VAT, the greater the rightward shift of the V-slope. We hypothesized that this rightward shift of the V-slope (RtShift) might be of clinical use as an index of exercise tolerance, if it could be quantified mathematically. This paper describes a method we have developed to mathematically derive RtShift and to elucidate whether this objective measure is in fact related to the level of VAT.

## Methods

### Patient characteristics

The CPX records of 100 patients with cardiac disease who underwent routine exercise testing and cardiac rehabilitation were retrospectively analyzed. There were 91 men and nine women, with a mean age of 63.8 ± 10.2 years. The underlying heart diseases were post-acute myocardial infarction (*n* = 41), angina (*n* = 21), post-cardiovascular surgery (*n* = 19), congestive heart failure (*n* = 14), and others. The characteristics of the study population are summarized in Table 1. New York Heart Association classification was not performed.

**Table 1:** Patient characteristics

Variables	
Age, yr	63.8 ± 10.2
Male/Female	91/9
Body weight, kg	63.4 ± 11.7
BMI, kg.m^−2^	23.6 ± 3.2
Hemoglobin, g.dL^−1^	13.1 ± 1.5
Serum creatinine, mg.dL^−1^	1.1 ± 0.5
LVEF, %	54.1 ± 12.3
LVDd, mm	51.3 ± 7.3
Current medication	
Ca-antagonists	25
ACE-inhibitors/ARB	61
Diuretics	28
β-blockers	48
Nitrates	33
Digoxin	3
Anti-arrhythmics	18

Data are presented as mean ± SD or number. BMI indicates body mass index; LVEF, left ventricular ejection fraction; LVDd, left ventricular diastolic dimension; Ca, calcium; ACE, angiotensin-converting enzyme; and ARB, Angiotensin II Receptor Blocker.

In a prospective substudy, the effect of different ramp exercise protocols on RtShift was assessed in 12 healthy young male students belonging to various college sports clubs; their mean age, body weight, and height were 20.8 ± 1.0 years, 66.0 ± 5.2 kg, and 172.6 ± 5.7 cm, respectively. This substudy was performed at a different institution (National Hospital Organization Hakodate Hospital, Hakodate, Japan).

The research plan was approved by the institutional review board of two institutions: Hokko Memorial Hospital and National Hospital Organization Hakodate Hospital. The study was conducted according to the Declaration of Helsinki.

### Exercise test

Exercise tests were performed by using an upright bicycle with a breath-by-breath gas analyzer (AE300S; Minato Ikagaku, Tokyo, Japan). The ramp protocol of 5–15 W⋅min^−1^ (10 W⋅min^−1^ in 84 cases, 15 W⋅min^−1^ in 13 cases, and 5 W⋅min^−1^ in three cases) was used, preceded by a 3-min warm-up. For the ramp protocol: 5 W⋅min^−1^, 10 ⋅Wmin^−1^, and 15 W⋅min^−1^, the warm-up load (W) was 0, 10 and 15, respectively; and the ramp start load (W) was 0, 10 and 15, respectively. The test was mostly terminated shortly after a VAT point was identified on-screen; therefore, the test protocol was submaximal, primarily performed for the purpose of identifying an initial exercise training intensity [3]: the work rate or heart rate at VAT. The Borg scale (ranging from 6 to 20) was used for the evaluation of perceived exertion. For the respiratory data analysis, a 10-s average was used. The heart rate was monitored continuously and blood pressure was measured once every minute.

For the substudy to assess the effect of different ramp protocols, three ramps (15, 25, and 50 W⋅min^−1^) were employed (symptomatic maximal exercise, on three different days 1 week apart).

Additionally, one normal volunteer performed an exercise protocol with three six-min steady state steps; this was compared with a ramp protocol exercise to graphically demonstrate the effects of steady state exercise on CO_2_ storage in the framework of V-slope. In the past, the phenomenon has been repeatedly shown, but depicted graphically always with the elapsed time (s or min) on the x-axis, and VCO_2_ on the y-axis (1,7). Written informed consent was obtained before each exercise test.

### Determination of VAT

**Fig. 1:**
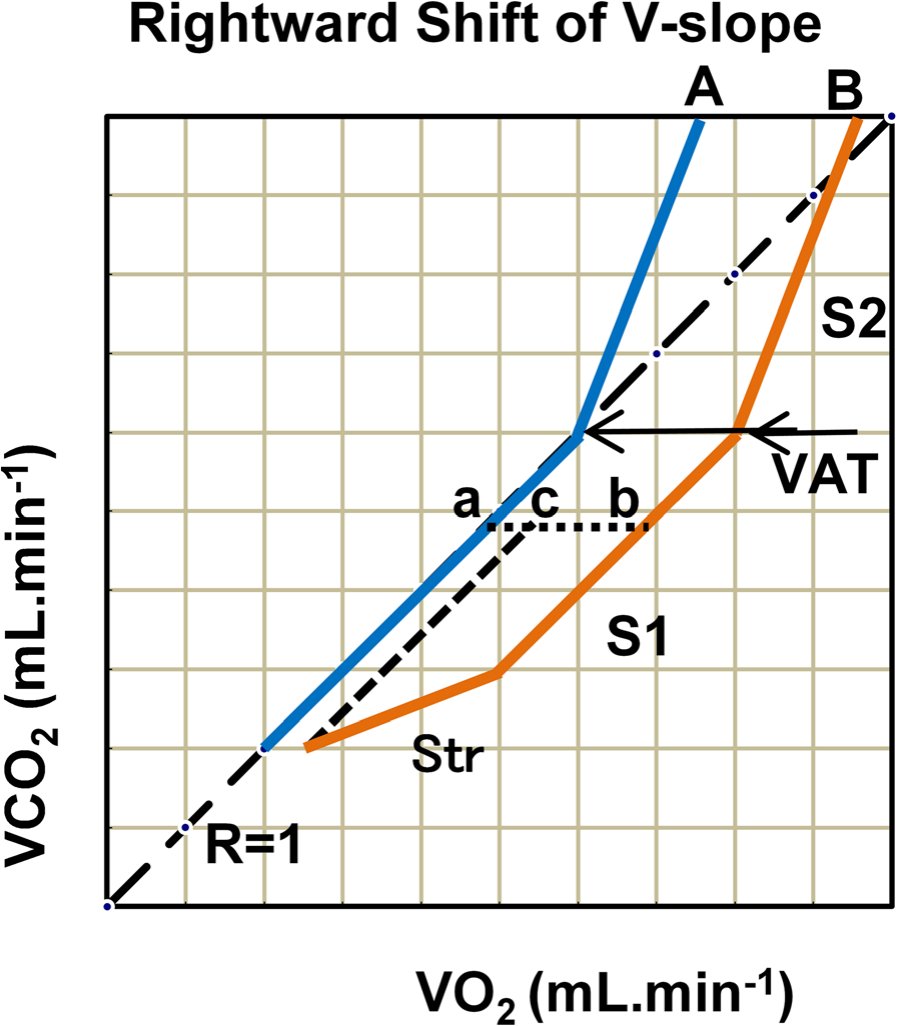
Schema showing the concept of rightward shift of V-slope (RtShift) and the determination of ventilatory anaerobic threshold (VAT) based on RtShift. At the outset of exercise, V-slope B shifts rightward to varying degrees (Str, slope transient) before it becomes stable in a line parallel to the *R* = 1 line (S1). At VAT, the V-slope deflects upward to continue its ascent (S2). V-slope A is shown as a control without any RtShift. The distance (b–a) is the rightward shift of V-slope. When adjustment is made for the initial resting shift, the distance (c–a) is subtracted

VAT was primarily determined by using the V-slope method, as defined by Wasserman et al. [1]. However, the details of determination were based on those defined by Beaver et al. [8] and particularly by Sue et al. [9]. We drew the whole V-slope schematically as in Fig. 1. Two types of V-slope, types A and B, are shown. Type B is by far the most common type; the first segment of the slope starts from resting data points and moves both rightward and upward to varying degrees during the warm-up and into the early ramp periods. This segment characteristically has an angle of <45°. We call this segment Str (slope transition). The V-slope then starts to ascend in parallel with the *R* = 1 line, at an angle of 45° (S1, pre-VAT slope). A break from S1 marks the appearance of VAT. Thereafter, the V-slope becomes steeper, with an angle of >45° (S2). In contrast, type A simply ascends with the R1 line before VAT. Further details of VAT determination and the V-slopes of all 100 cardiac subjects with VAT break points marked are included in Additional file 2: Figure S2. Overall, the VAT could not be determined in five cases.

The hypothesis that the S1 is parallel to the diagonal *R* = 1 was tested as described [10]. After all of the VAT points and S1 data points (those visually interpreted as being located on or around the hypothetical S1 line parallel to *R* = 1) had been finalized, the mean of the S1 slopes was tested against the slope of 1.0. Subsequently, the next data point on the V-slope was added and again tested against 1.0. The procedure was further repeated after sequentially adding the next data point.

The 95% limit of agreement for VAT determination was 170 mL⋅min^−1^ VO_2_ as per our experience [10]. This is based on the comparison of the averages of two readings of each assessor (i.e., A and B), therefore representing the inter-individual agreement.

### Rightward shift of the V-slope (RtShift)

#### Schematic representation of the concept of RtShift

The concept of RtShift is also schematically represented in Fig. 1. The S1 of V-slope A has no RtShift, being on the *R* = 1 line. The S1 of V-slope B is shifted rightward. The RtShift is defined as the horizontal distance between the two parallel lines B and A (b minus a), represented by the dotted line. However, the V-slope graph is conventionally drawn as a regular square with identical x and y scales; therefore, on the diagonal line, the x-value is identical to the y-value. The VO_2_ at “**a**” is equal to VCO_2_ at “**a**,” which then subsequently equals VCO_2_ at “**b**.” Therefore, the RtShift (b minus a) is simply calculated as a difference in mL⋅min^−1^ (VO_2_ minus VCO_2_) at a single data point “**b**.” This diagram shows an interesting way in which RtShift affects a VAT value expressed as VO_2_ mL⋅min^−1^; by merely shifting the whole S1 V-slope rightward, the VAT on B becomes greater than that on A. Therefore, it seems reasonable to assume that the level of VAT is variably augmented by the presence of RtShift.

#### Mathematical derivation of RtShift

**Fig. 2:**
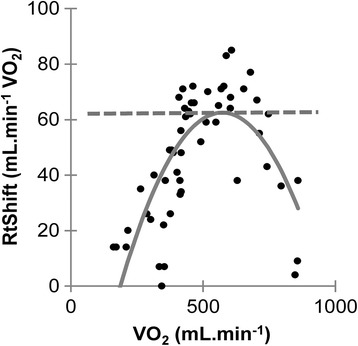
Coordinate conversion. The second objective method of estimating the representative RtShift. The conventional VO_2_ versus VCO_2_ relation is converted to the VO_2_ versus RtShift relation. The best-fit quadratic equation is obtained and the tangential line to its highest point is drawn. The vertical y value is the representative RtShift

Before the mathematical derivation of RtShift, we processed the data as follows. We employed only data points that are equal to or below *R* = 1 throughout the resting and exercise periods (warm-up and ramp). Outlier data points were eliminated from further analysis by using the Smirnov-Grubbs test; 24 data points were removed as a result. Next, we converted the conventional V-slope plot to the plot wherein RtShift is plotted against VO_2_ as in Fig. 2. On identifying a break point on the conventional V-slope, one is forced to detect a rising break point on an already rising S1 baseline. With the converted coordinate system, we have a normalized x–y-axis view of the relation between VO_2_ and the excess VCO_2_ (RtShift). A typical plot yielded a trapezoidal form; however, with a shorter S1, the plot became more like a triangle with a blunted apex.

We attempted to quantify RtShift by using following three methods.

The first method determined RtShift through the visual inspection of a V-slope. For this method we used the VO_2_ vs. RtShift graph. Additionally we also used the time (s) vs. RtShift graph, because this graph could avoid the overlapping of RtShift data points over a different time sequence, which is sometimes the problem on the VO_2_ vs. RtShift graph. The RtShift was visually taken as the highest parallel line to *X* = 0 (therefore, *R* = 1); occasionally, more than one horizontal line could be visualized before VAT. The second method involved the use of a curve-fitting program. As the plot pattern on the converted V-slope graph mostly resembled a trapezoid or blunted triangle, we used quadratic regression to represent the curve (Fig. 2). Differentiation of the quadratic equation (ax^2^ + bx + c) yields (ax + b); solving (ax + b = 0) for x gives the tangent to the apex of the curve, RtShift. In 11 cases, the quadratic equation could not yield a regression curve with the convex within the data range (i.e., failure rate of 11%), because of a small data set and/or a large dispersion of data points, in which cases, a mathematical average of all data points equal to or below *R* = 1 was used instead. The third method was simply a mathematical average of all data points at or below *R* = 1, assuming no particular type of curve. This is equivalent to the integral of all RtShift values divided by the number of data points. In Additional file 2: Figure S2, each V-slope is drawn with the diagonal *R* = 1 line and a marked VAT point, so that the relation between RtShift and VAT may be individually ascertained.

Because RtShift defined this way included a shift at rest (calculated as an average resting VO_2_ minus an average resting CO_2_), we may correct for this by subtracting this resting RtShift from the exercise RtShift. However, we decided that this would be unnecessary because we wanted to compare RtShift against VAT, which itself included a resting value; a resting VO_2_ is not subtracted from a VAT value.

The ΔVO_2_/Δwork rate was calculated in the standard way [10]. In two cases, the ratio could not be calculated because of the small number and large dispersion of the data points.

#### Left ventricular function

Left ventricular dimension and function were assessed by echocardiography (Toshiba Aplio ™400, Toshiba, Tokyo, Japan). Left ventricular diastolic dimension (LVDd) was measured on the M-mode scan. Ejection fraction was calculated with the modified Simpson method.

### Statistical analysis

Data are presented as mean ± SD. The distribution of RtShift was not Normal; therefore, the data for RtShift are presented both as mean ± SD and as median with interquartile range (IQR) and lower/upper quartile. Outlier detection was performed with the Smirnov-Grubbs test. Correlation was assessed by using Pearson’s r. Comparison between two groups was performed with Student’s t-test. Comparisons between RtShift calculated through three different methods, and between RtShift in the three ramp protocols, were performed by using one-way repeated-measures analysis of variance (ANOVA) followed by the Bonferroni multiple comparison procedure. Multiple linear regression analysis was performed to assess whether VAT, end-tidal CO_2_ concentration (as a sign of hyperventilation), resting RtShift, the use of beta-blocker, and factors such as age and weight independently contributed to RtShift. Because of the non-Normal nature of the data, we also applied non-parametric tests: namely, Spearman’s rank order correlation for correlation and the Friedman test as an alternative to repeated-measures ANOVA).

## Results

The basic exercise data are summarized in Table 2.

**Table 2:** Exercise data summary

Variables	
Exercise time (ramp), min	7.1 ± 2.3
At highest work rate	
Work rate, watt	76.1 ± 29.0
Heart rate, bpm	115 ± 19
SBP, mmHg	177 ± 29
VO_2_, mL⋅min^−1^	1002 ± 362
VO_2_, m⋅Lkg^−1^⋅min^−1^	17.3 ± 4.7
RR, breath⋅smin^−1^	26 ± 5.4
VE, L⋅min^−1^	38.4 ± 11.6
RER	1.1 ± 0.1
Borg scale: chest (/20)	12.5 ± 2.0
Borg scale: leg (/20)	14.2 ± 2.2
VAT, m⋅Lmin^−1^ VO_2_	635 ± 220
VAT, mL⋅kg^−1^⋅min^−1^ VO_2_	10.0 ± 2.6
ΔVO_2_/Δwatt, mL⋅min^−1^⋅W^−1^	9.7 ± 1.3
RtShift, mL⋅min^−1^ VO_2_	33.9 ± 25.0
RtShift, mL⋅kg^−1^⋅min^−1^ VO_2_	0.52 ± 0.33
RtShift, mL⋅min^−1^ VO_2_:	
median, IQR	29.8, 25.5
lower and upper quartile	15.3, 40.9

Data are presented as mean ± SD. SBP indicates systolic blood pressure; VO_2_, indicates minute oxygen uptake; RR, respiration rate; RER, respiratory exchange rate; VAT, ventilatory anaerobic threshold; VCO_2_, minute carbon dioxide production; RtShift, rightward shift of V-slope; IQR, interquartile range.

**Fig. 3:**
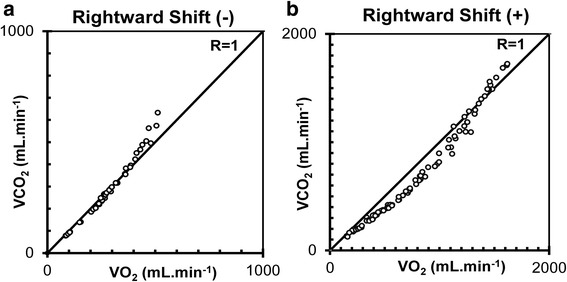
Typical examples of a V-slope without (**a**: *left*) and with (**b**: *right*) RtShift. The shift of V-slope is judged relative to the *R* = 1 diagonal line

**Fig. 4:**
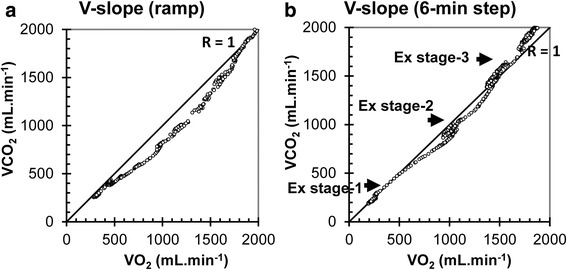
Comparison of V-slopes during ramp (**a**: *left*, ramp) and 6-min steady-state exercise (**b**: *right*, step) performed by the same subject. During ramp exercise, the V-slope maintains its RtShift up to VAT; however, during steady-state exercise, it reverts back to the *R* = 1 line

First, typical examples of a case with very little rightward shift (type A) and a moderate shift (type B) of the V-slope are shown in Fig. 3(a, b). Next, the maintenance of RtShift during a ramp protocol (Fig. 4(a)) is contrasted with its lack during a steady-state protocol (Fig. 4(b)) consisting of a V-slope during three six-min steps. The rightward shift is noted to occur only at the start of the step increase, ultimately reverting back later.

Among the visual method and the two mathematical methods for estimating RtShift, the first method (visual) yielded the highest mean RtShift, followed by the second (quadratic curve fitting) and then the third (simple averaging) method. The mean RtShift values were as follows: first, 50.8 ± 41.6; second, 42.0 ± 34.4; third, 33.9 ± 25.0 mL⋅min^−1^ VO_2_ (each different from the others, *p* < 0.001). However, the RtShift of each method correlated well with each other (first vs. second, *r* = 0.955; second vs. third, *r* = 0.989; first vs. third, *r* = 0.945). We decided to adopt the third method (simple averaging) to determine RtShift because there was no failure associated with the determination; however, with the second method, there was an 11% failure rate in determining RtShift. The third method also utilized all data points.

As mentioned in the Statistical analysis section, RtShift did not have a Normal distribution (Additional file 3: Figure S3(a)).

**Fig. 5:**
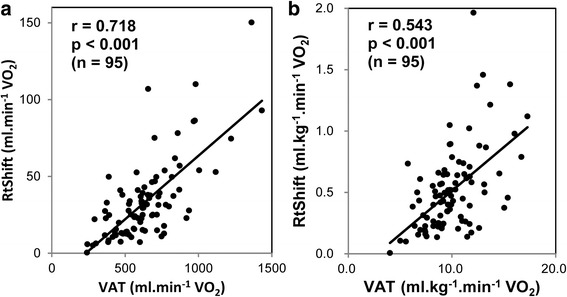
Relationship between ventilatory anaerobic threshold (VAT) and RtShift (**a**, **b**: without and with weight adjustment). VAT correlated positively with RtShift

VAT (mL⋅min^−1^) and RtShift (mL⋅min^−1^ VO_2_) were significantly correlated (*r* = 0.718, *p* < 0.001; adjusted for weight, *r* = 0.543, *p* < 0.001); the greater the VAT, the greater the rightward shift (Fig. 5). VAT was also significantly correlated with RtShift after correction for the resting RtShift (*r* = 0.402, *p* < 0.001).

VAT, resting RtShift, and resting end-tidal CO_2_ concentration were each significant (*p* < 0.001, *p* < 0.001, and *p* = 0.003; standardized correlation coefficient: 0.5699, 0.3966, and −0.2032, respectively). The univariate correlation between RtShift and resting end-tidal CO_2_ concentration was, however, only 0.094 (*p* = 0.351). The use of a β-blocker was not a significant variable in this analysis (*p* = 0.612).

Both VAT and RtShift were significantly correlated with ΔVO_2_/Δwork rate, a marker of oxygen uptake efficiency (10 ⋅Wmin^−1^, *n* = 83; *r* = 0.531, *p* < 0.001; adjusted for weight, *r* = 0.393, *p* < 0.001, respectively) (Additional file 3: Figure S3(b)).

The result of the hypothesis testing that S1 is parallel to the diagonal *R* = 1 was as follows: the first test of the mean S1 slope (1.013 ± 0.084) against the slope of 1.0 was found to be not significantly different from 1.0 (*p* = 0.132). Subsequently, the next data point on the V-slope was added and again tested against 1.0; the mean slope was 1.056 ± 0.090, significantly greater than 1.0 (*p* < 0.001). As the procedure was further repeated after sequentially adding the next data point, the mean S1 slope increased progressively. Furthermore, the individual linear regression line through the S1 data points varied depending on the number of data points included; a long S1 consisting of more data points appeared to converge to 1.0, whereas a short S1 with a few data points tended to diverge. However, the mean of all S1 linear regression lines (95 cases in total) was not significantly different from 1.0 (Additional file 3: Figure S3(c)).

The effect of the three different protocols (15, 25, and 50 W⋅min^−1^) on RtShift (mL⋅min^−1^ VO_2_) was not statistically significant (15 W⋅min^−1^⋅, 132.2 ± 52.5; 25 W⋅min^−1^, 137.8 ± 48.5; 50 W⋅min^−1^, 136.1 ± 49.3; *p* = 0.946).

Non-parametric statistical tests yielded concordant results with the parametric tests. The detailed results are presented in the additional file (Additional file 4).

## Discussion

During steady-state exercise, CO_2_ storage in the tissue occurs at the start and disappears after 1–2 min, when presumably the storage space has been filled. This has been observed since the 1960s [4–7]. In addition, the literature acknowledges that, even during incremental or ramp exercise testing, there is an artifact due to this phenomenon at the beginning of exercise and this segment of V-slope is to be excluded when VAT is determined [8, 11]. What has not been clearly pointed out is that during ramp exercise, this CO_2_ tissue storage effect never disappears and persists at least until VAT begins (Fig. 4). Because of its nature, ramp exercise never achieves a steady state. From the observed persistence of CO_2_ storage, it may be reasoned that the CO_2_ storage space also increases and its rate of increase remains constant as the work rate increases. We hypothesize that, with the constantly increasing work rate, new groups of muscle fibers are recruited, resulting in a steady increase in new CO_2_ storage space in active muscle.

CO_2_ storage was initially measured by using hyperventilation and/or rebreathing methods [4–6]. Later, CO_2_ storage was calculated as the difference between the measured CO_2_ and the predicted CO_2_, based on the premise of a fixed respiratory quotient during sublactate-threshold exercise [12–14]. Estimates from the hyperventilation methods, with the apparent inclusion of an HCO_3_^−^ diffusing space in the whole body, were much larger than those from the rebreathing methods. The former measure of CO_2_ storage was presumed to result only from tissue with a high metabolic rate, such as active muscle [14]. There is a possibility that the phenomenon of RtShift may be chiefly metabolic in origin, such as a shift to fat utilization as energy source, instead of due to CO_2_ storage. However, we believe that this is highly unlikely because the RtShift occurs very early at the outset of exercise, whereas a major shift in the energy source to fat takes much longer to manifest, such as 20 min of steady-state exercise [15]. With ramp exercise too, there is no reason to believe that the energy source change to fat occurs so early, although no such basic study has ever been done during ramp protocols. Moreover and most important, the RtShift disappears (V-slope turns leftward) in 2–3 min while the work rate is being maintained at the same level (steady state). We assumed, on the basis of visual observations, that the slope of S1 of V-slope is approximately 1.0. However, the literature suggests that the RQ (R during steady-state exercise) increases by 0.05–0.1 from the resting value to moderate work rates [16]. This magnitude of RQ change is too small compared with R of either RtShift (far below 1.0) or S2 (far above 1.0). Therefore, we do not believe that the hypothesis of the slope of S1 being exactly 1.0 is an absolute requisite in the practical application of VAT and RtShift determination. Although there are no data on the energy utilization during ramp or rapid incremental testing, during work rate transition from rest to exercise, part of the energy fuel is estimated to come from phosphocreatine breakdown and glycolysis [17], both producing H^+^ and probably excess CO_2_. Ramp exercise may be considered to consist of multiple work rate transitions; therefore, R may tend to increase slightly even during increments of light exercise intensity. Therefore, the observed slope of S1 of 1.0 (or its being parallel to *R* = 1 line) may be a result of a competing net effect of these factors (increasing slope of S1) and the RtShift (decreasing slope of S1). Wasserman states that the slope of S1 is slightly less than 1.0 [1].

The major finding of this research—that the size of the CO_2_ storage space (RtShift) is associated with increased exercise tolerance—may, at first, seem surprising. However, the above hypothesis suggests that a greater mass of aerobic muscle fibers recruited for a given workload may be a contributing mechanism. Additionally, a greater level of carbonic anhydrase (CA) activity in muscle may be a factor. As it is now known that there are subtypes of CA expressed in muscle [18], the hydration of CO_2_, and therefore the production and retention of HCO_3_^−^ in muscle, may be facilitated by this mechanism. CA activity may increase as part of the overall increase in aerobic enzyme activity in muscle [19]. The only other study evaluating the relation between exercise tolerance and CO_2_ storage is that of Chuang et al. [20], in which the authors demonstrated a significant positive correlation between O_2_ deficit and CO_2_ store in 12 relatively young healthy subjects. However, in their study, the direct relation between indices of exercise tolerance (VO_2_max or VAT) and the size of the CO_2_ store was not assessed.

The RtShift has several distinctive features that may be useful in the evaluation of exercise tolerance. Foremost among them is that RtShift does not require any assessor to determine its value. Second, its calculation is simple. Third, it has the additional advantage of an almost 100% determination rate. Fourth, RtShift, like VAT, does not require maximal exercise testing; it is mostly calculated from data points up to *R* = 1; therefore, it is unique in that it is primarily an aerobic phenomenon. Although it is significantly correlated with VO_2_ peak and VAT, the correlation coefficient is only moderate (~0.7). Therefore, although it may not be used as a substitute for VAT, it may indicate that a yet-unidentified physiological factor that is absent in both VO_2_ peak and VAT is involved. That these two parameters do not always move in parallel is obvious from the only moderate correlation efficient between them and the inspection of each case of V-slope (Additional file 2: Figure S2).

Multiple regression analysis was used to assess whether factors other than VAT accounted for the association between RtShift and VAT. It has been reported that hyperventilation before exercise caused a rightward shift of the V-slope [21, 22]. However, that was not a major factor in this study.

As shown in this study, RtShift may be quantitatively defined by more than one method. In this study, we chose a method of simple averaging. However, any other method may be devised as long as it can provide a valid estimate of RtShift during incremental exercise testing.

The substudy was performed because we were not sure how a ramp change of exercise protocol affected the size of RtShift. If the RtShift is dependent on the recruited muscle mass, then the steeper ramp may result in a greater RtShift. As shown in the Results section, ramp change did not result in a significant change in RtShift.

The RtShift data distribution was clearly not Normal, with a deviation to the lower values of RtShift. Log transformation of the data was performed; however, the statistical results of various analyses did not materially differ from those obtained with raw data. We also ran nonparametric tests with the concordant results with parametric tests. Therefore, we chose to present only nontransformed data for analysis, which has the benefit of visually corresponding to the shift on the V-slope graph. In addition, we believe that because of the relatively large size of our data set (*n* = 100), the central limit theorem applies [23].

A limitation of this study is that the exact physiological mechanism through which RtShift is related to exercise tolerance is, at present, only speculative. The exact biological basis of RtShift must be elucidated through basic science research. Another limitation is that we do not have an age- and sex-matched control population of sufficient size comparing RtShift between patients with cardiac disease and normal subjects. This issue must be systematically and prospectively addressed; in this study, we merely completed the process of quantifying RtShift.

## Conclusions

In conclusion, during incremental CPX, there were varying degrees of rightward shift of the V-slope and they correlated significantly with VAT, suggesting that RtShift may be used as a completely objective measure of exercise tolerance. It is predominantly determined before the appearance of VAT. Its physiological meaning and clinical application requires further clarification.

## Additional files

Additional file 1: Figure S1. Our past experience on the relation between ventilatory anaerobic threshold (VAT) and RtShift. (PPTX 138 kb)

Additional file 2: Figure S2. Individual V-slope plots (*n* = 100). (PPTX 3053 kb)

Additional file 3: Figure S3. (a) Histogram of RtShift data distribution. (b) RtShift versus ΔVO_2_/Δwork rate. (c) Test of S1 being parallel to *R* = 1. (PPTX 108 kb)

Additional file 4: Result: Results of non-parametric statistical analyses. (DOCX 13 kb)

Additional file 5: Table S1. Complete data set. (XLSX 26 kb)

## Abbreviations

ACE: Angiotensin-converting enzyme
ARB: Angiotensin II receptor blocker
BMI: Body mass index
Ca: Calcium
CA: Carbonic anhydrase
CPX: Cardiopulmonary exercise testing
IQR: Interquartile range
LVDd: Left ventricular diastolic dimension
LVEF: Left ventricular ejection fraction
R: Respiratory exchange rate
RQ: Respiratory quotient
RR: Respiration rate
RtShift: Rightward shift of V-slope
S1: pre-VAT slope of the V-slope or the S1 itself (if the slope is not mentioned)
S2: post-VAT slope of the V-slope or the S2 itself (if the slope is not mentioned)
SBP: Systolic blood pressure
VAT: Ventilatory anaerobic threshold
VCO_2_: Minute carbon dioxide production
VO_2_: Minute oxygen uptake.
