# Supplementary material for: Quantification and physiological significance of the rightward shift of the V-slope during incremental cardiopulmonary exercise testing
Source: BMC Sports Sci Med Rehabil. 2017 Apr 20;9:9. doi: 10.1186/s13102-017-0073-1 (PMC5397810; doi:10.1186/s13102-017-0073-1)
Supplement: Supplementary file 1 — Our past experience on the relation between ventilatory anaerobic threshold (VAT) and RtShift. (PPTX 138 kb) [file 13102_2017_73_MOESM1_ESM.pptx]

## Slide 1
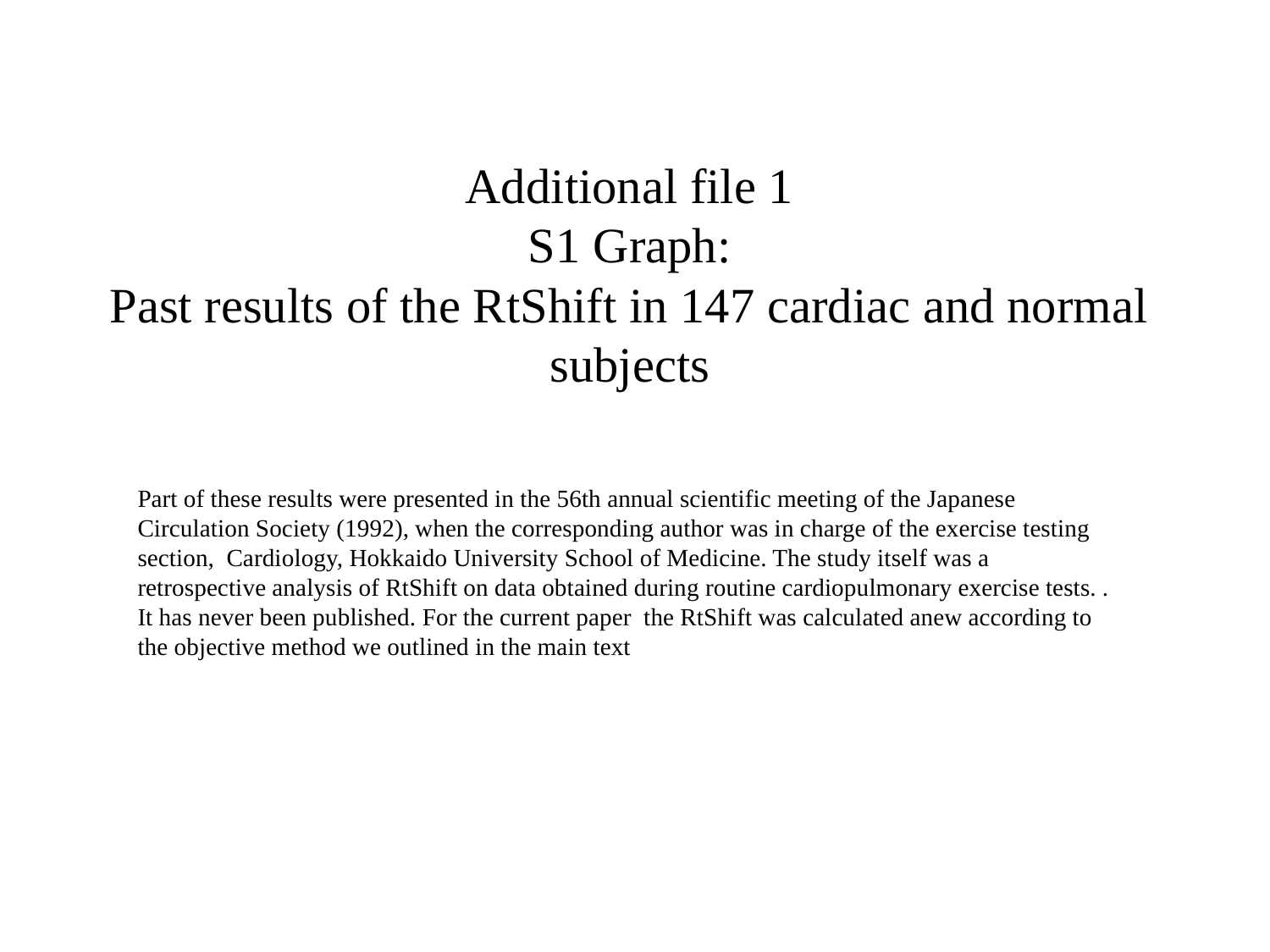

# Additional file 1 S1 Graph: Past results of the RtShift in 147 cardiac and normal subjects
Part of these results were presented in the 56th annual scientific meeting of the Japanese Circulation Society (1992), when the corresponding author was in charge of the exercise testing section, Cardiology, Hokkaido University School of Medicine. The study itself was a retrospective analysis of RtShift on data obtained during routine cardiopulmonary exercise tests. . It has never been published. For the current paper the RtShift was calculated anew according to the objective method we outlined in the main text

## Slide 2
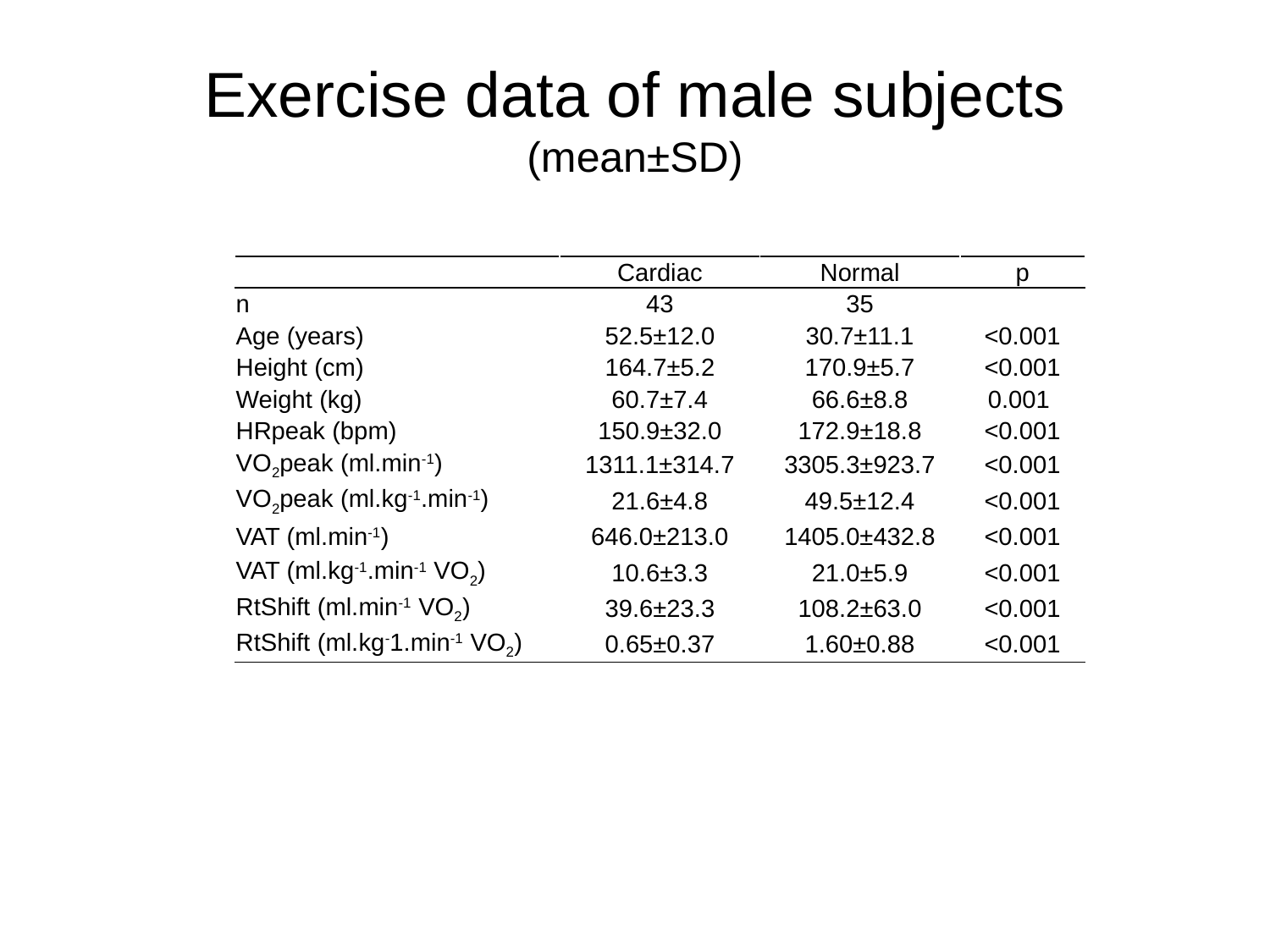

# Exercise data of male subjects (mean±SD)
| | Cardiac | Normal | p |
| --- | --- | --- | --- |
| n | 43 | 35 | |
| Age (years) | 52.5±12.0 | 30.7±11.1 | <0.001 |
| Height (cm) | 164.7±5.2 | 170.9±5.7 | <0.001 |
| Weight (kg) | 60.7±7.4 | 66.6±8.8 | 0.001 |
| HRpeak (bpm) | 150.9±32.0 | 172.9±18.8 | <0.001 |
| VO2peak (ml.min-1) | 1311.1±314.7 | 3305.3±923.7 | <0.001 |
| VO2peak (ml.kg-1.min-1) | 21.6±4.8 | 49.5±12.4 | <0.001 |
| VAT (ml.min-1) | 646.0±213.0 | 1405.0±432.8 | <0.001 |
| VAT (ml.kg-1.min-1 VO2) | 10.6±3.3 | 21.0±5.9 | <0.001 |
| RtShift (ml.min-1 VO2) | 39.6±23.3 | 108.2±63.0 | <0.001 |
| RtShift (ml.kg-1.min-1 VO2) | 0.65±0.37 | 1.60±0.88 | <0.001 |

## Slide 3
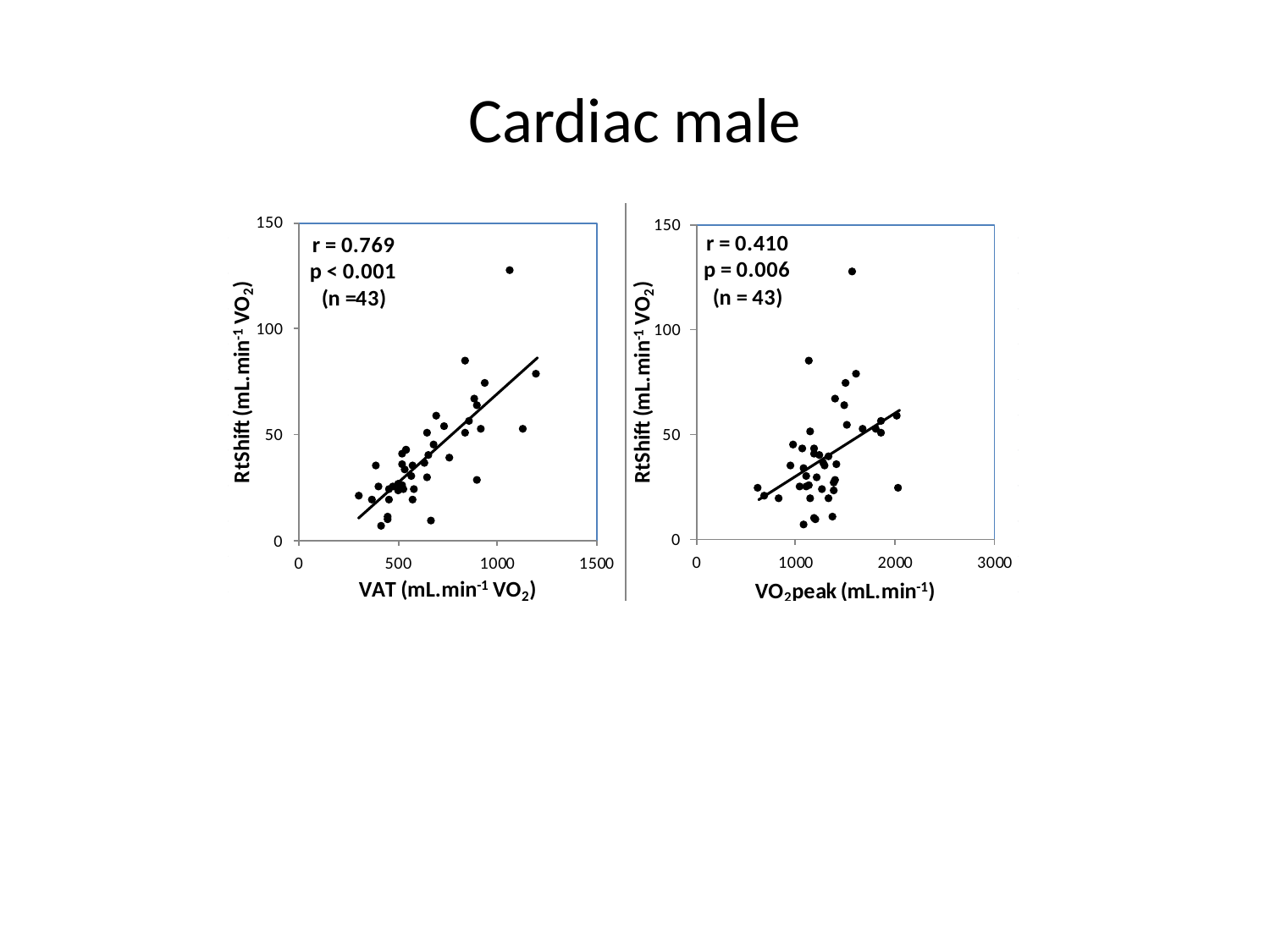

# Cardiac male

## Slide 4
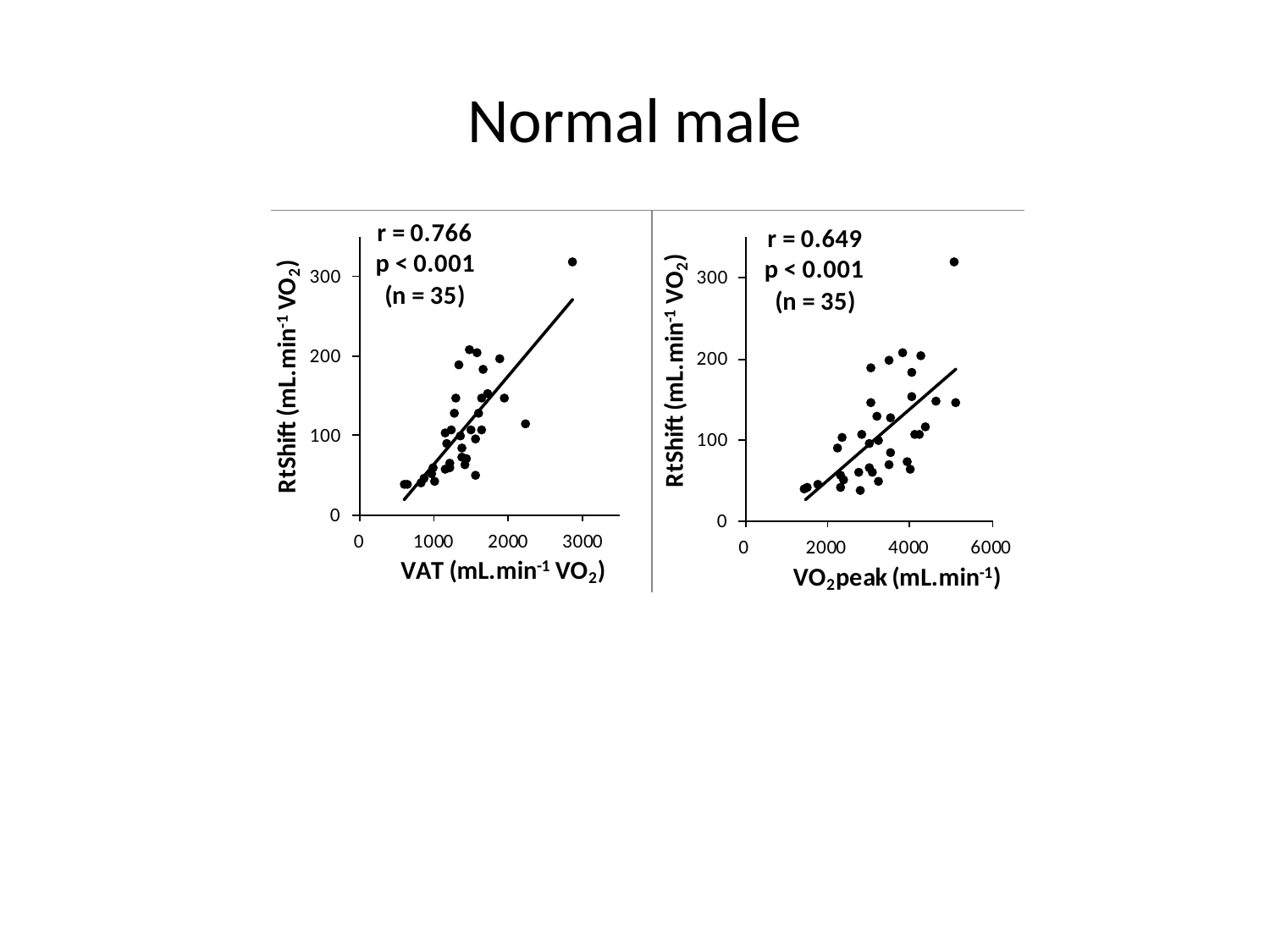

# Normal male

## Slide 5
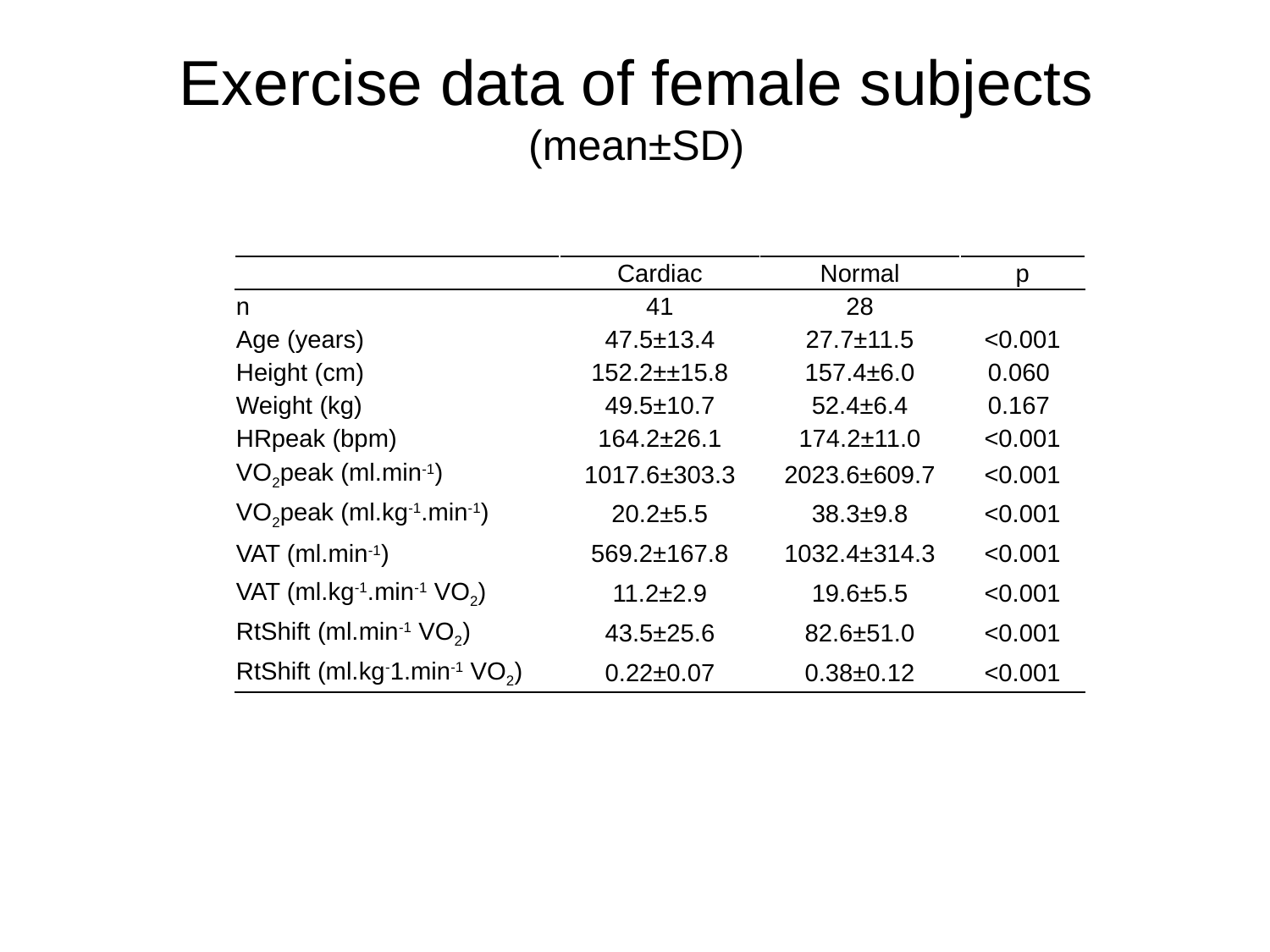

# Exercise data of female subjects (mean±SD)
| | Cardiac | Normal | p |
| --- | --- | --- | --- |
| n | 41 | 28 | |
| Age (years) | 47.5±13.4 | 27.7±11.5 | <0.001 |
| Height (cm) | 152.2±±15.8 | 157.4±6.0 | 0.060 |
| Weight (kg) | 49.5±10.7 | 52.4±6.4 | 0.167 |
| HRpeak (bpm) | 164.2±26.1 | 174.2±11.0 | <0.001 |
| VO2peak (ml.min-1) | 1017.6±303.3 | 2023.6±609.7 | <0.001 |
| VO2peak (ml.kg-1.min-1) | 20.2±5.5 | 38.3±9.8 | <0.001 |
| VAT (ml.min-1) | 569.2±167.8 | 1032.4±314.3 | <0.001 |
| VAT (ml.kg-1.min-1 VO2) | 11.2±2.9 | 19.6±5.5 | <0.001 |
| RtShift (ml.min-1 VO2) | 43.5±25.6 | 82.6±51.0 | <0.001 |
| RtShift (ml.kg-1.min-1 VO2) | 0.22±0.07 | 0.38±0.12 | <0.001 |

## Slide 6
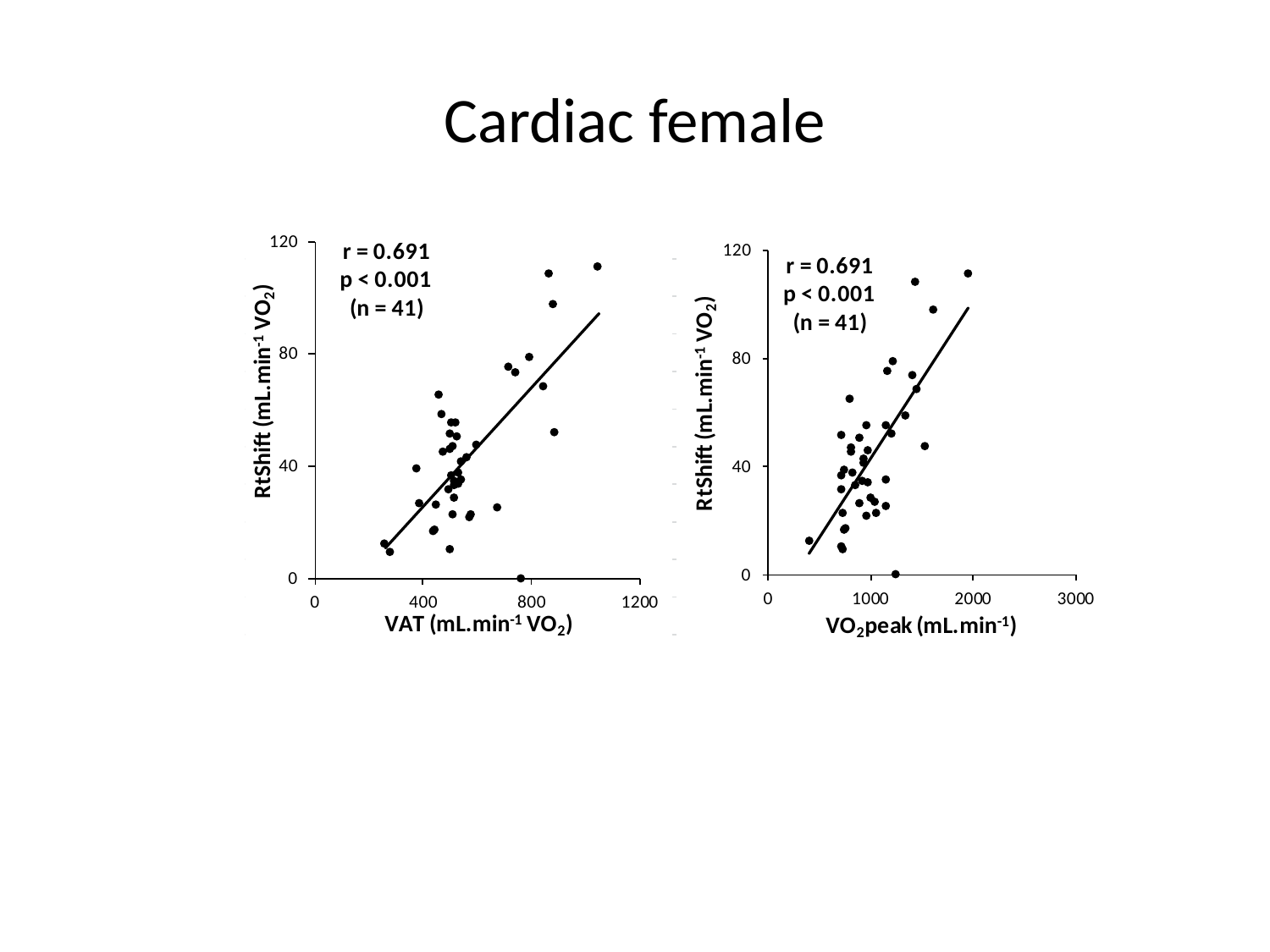

# Cardiac female

## Slide 7
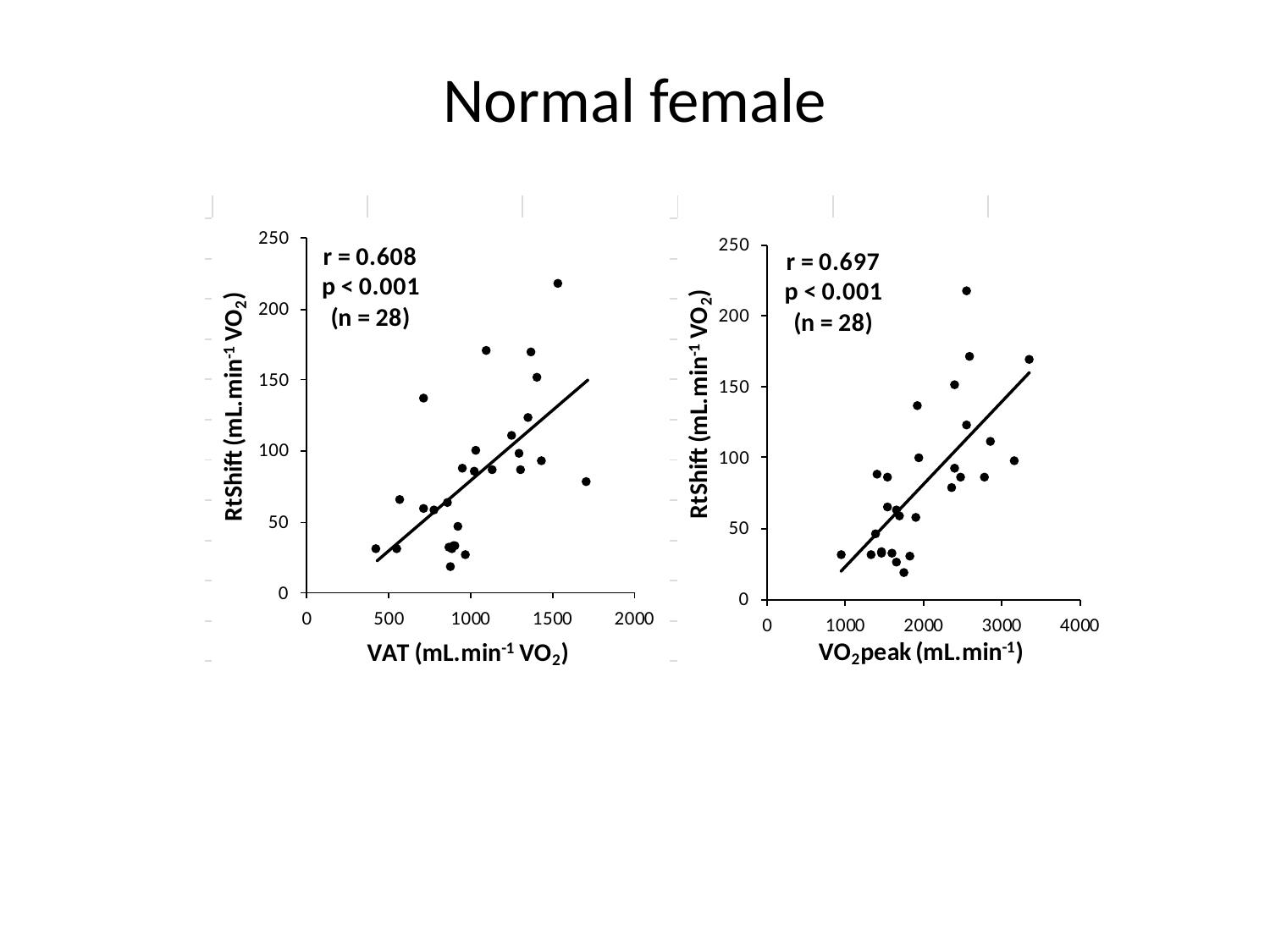

# Normal female
