# Supplementary material for: Quantification and physiological significance of the rightward shift of the V-slope during incremental cardiopulmonary exercise testing
Source: BMC Sports Sci Med Rehabil. 2017 Apr 20;9:9. doi: 10.1186/s13102-017-0073-1 (PMC5397810; doi:10.1186/s13102-017-0073-1)

## Slide 1
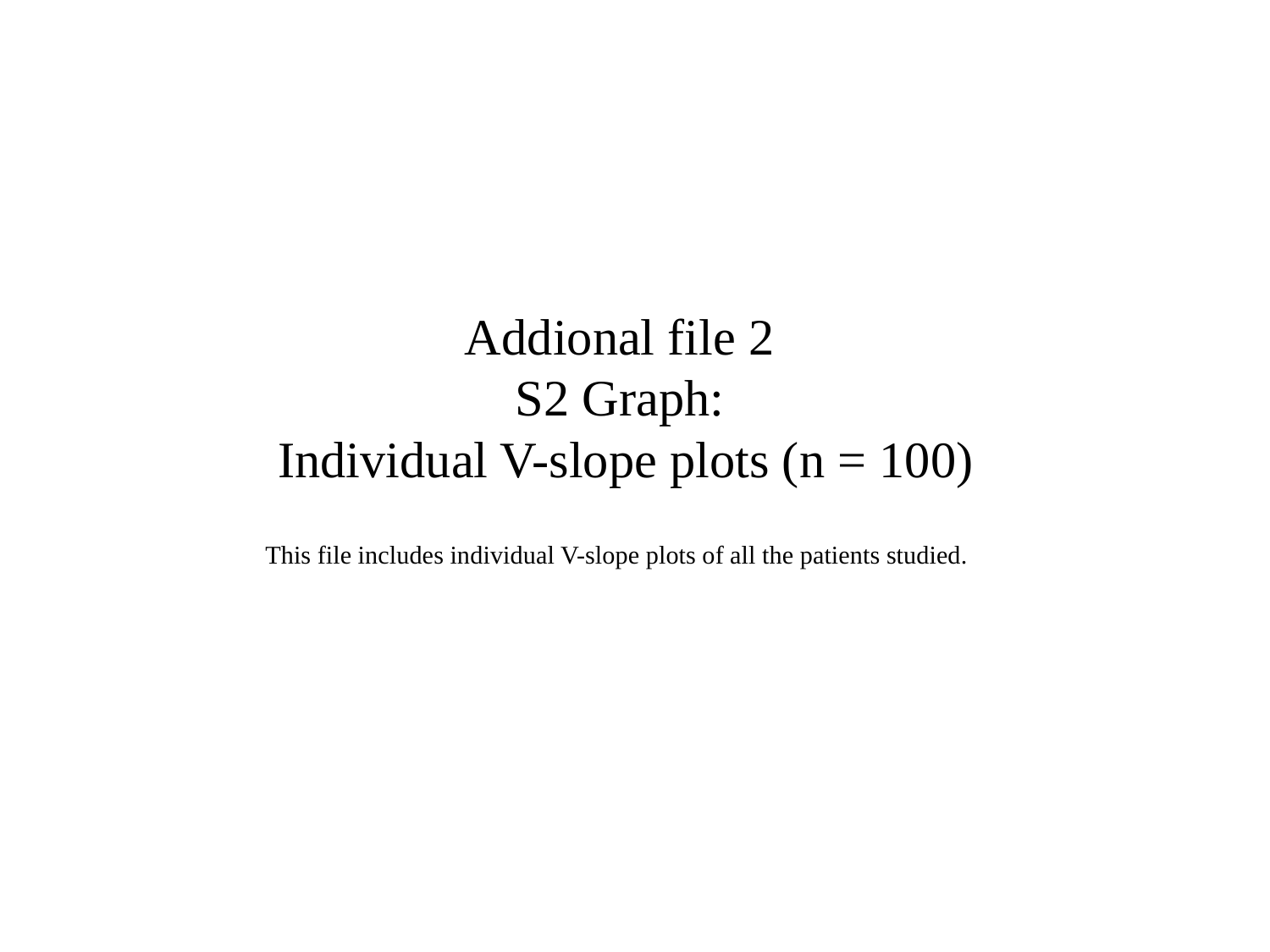

# Addional file 2 S2 Graph: Individual V-slope plots (n = 100) This file includes individual V-slope plots of all the patients studied.

## Slide 2
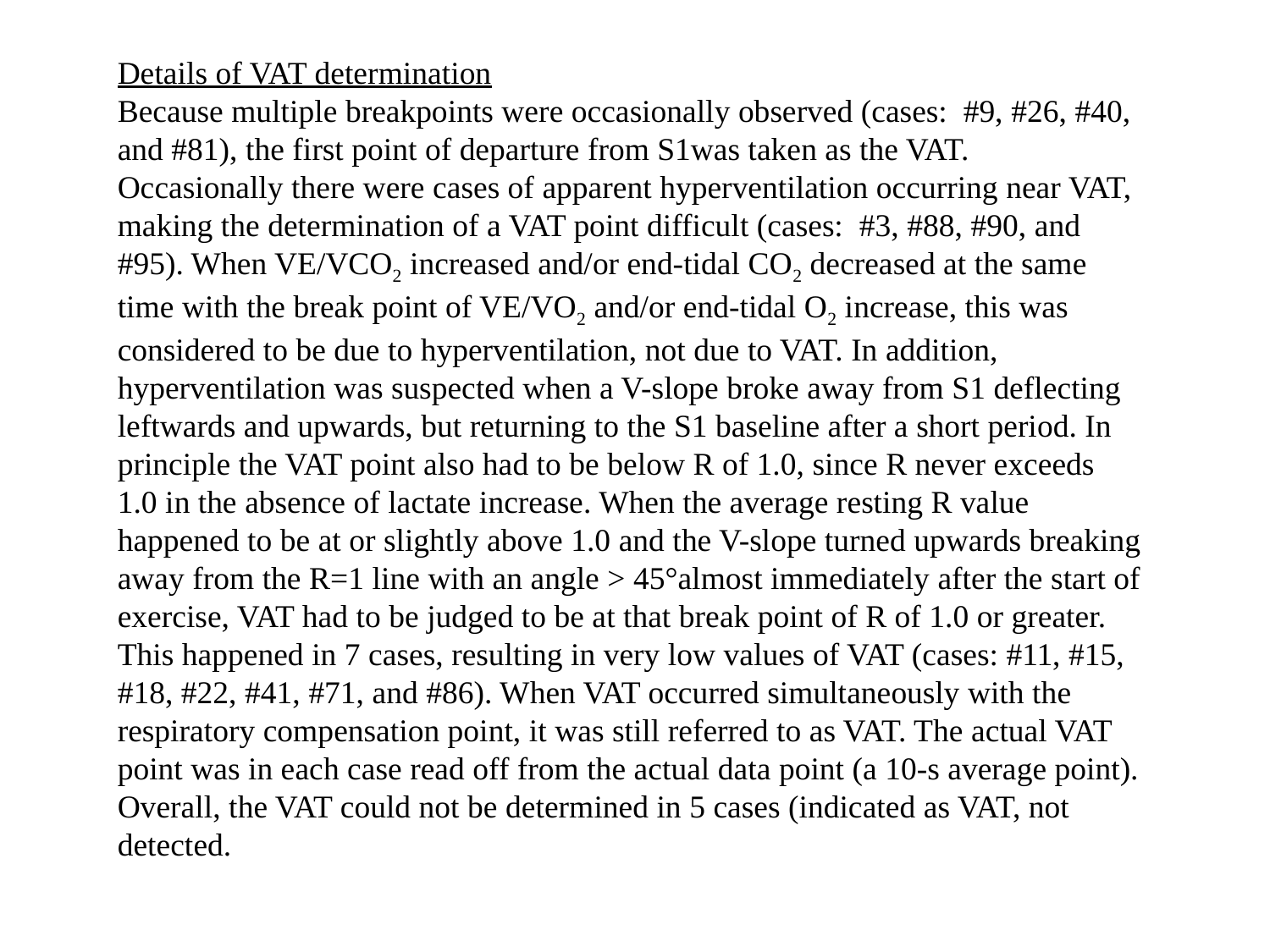

Details of VAT determination
Because multiple breakpoints were occasionally observed (cases: #9, #26, #40, and #81), the first point of departure from S1was taken as the VAT. Occasionally there were cases of apparent hyperventilation occurring near VAT, making the determination of a VAT point difficult (cases: #3, #88, #90, and #95). When VE/VCO2 increased and/or end-tidal CO2 decreased at the same time with the break point of VE/VO2 and/or end-tidal O2 increase, this was considered to be due to hyperventilation, not due to VAT. In addition, hyperventilation was suspected when a V-slope broke away from S1 deflecting leftwards and upwards, but returning to the S1 baseline after a short period. In principle the VAT point also had to be below R of 1.0, since R never exceeds 1.0 in the absence of lactate increase. When the average resting R value happened to be at or slightly above 1.0 and the V-slope turned upwards breaking away from the R=1 line with an angle > 45°almost immediately after the start of exercise, VAT had to be judged to be at that break point of R of 1.0 or greater. This happened in 7 cases, resulting in very low values of VAT (cases: #11, #15, #18, #22, #41, #71, and #86). When VAT occurred simultaneously with the respiratory compensation point, it was still referred to as VAT. The actual VAT point was in each case read off from the actual data point (a 10-s average point). Overall, the VAT could not be determined in 5 cases (indicated as VAT, not detected.

## Slide 3
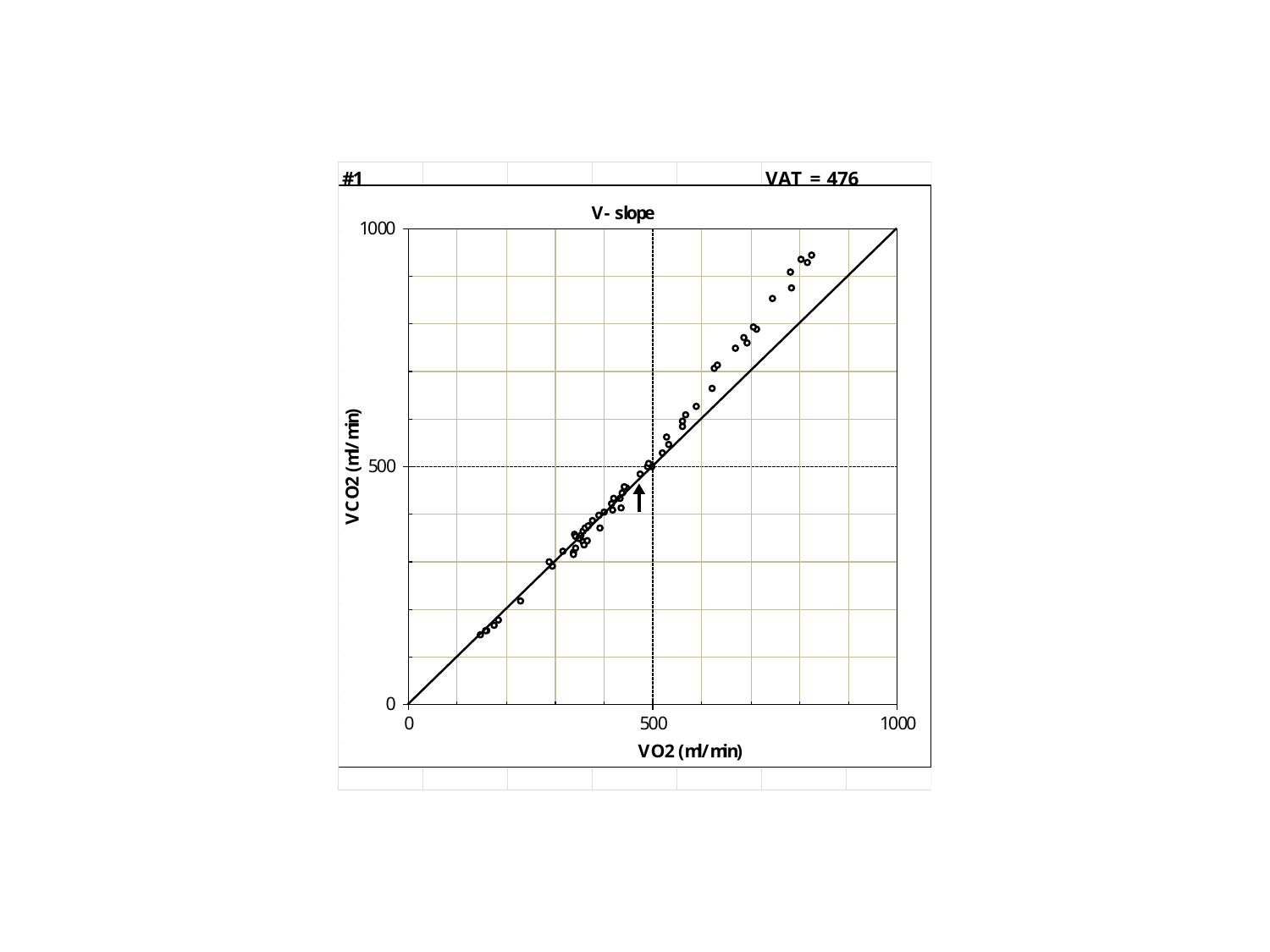

## Slide 4
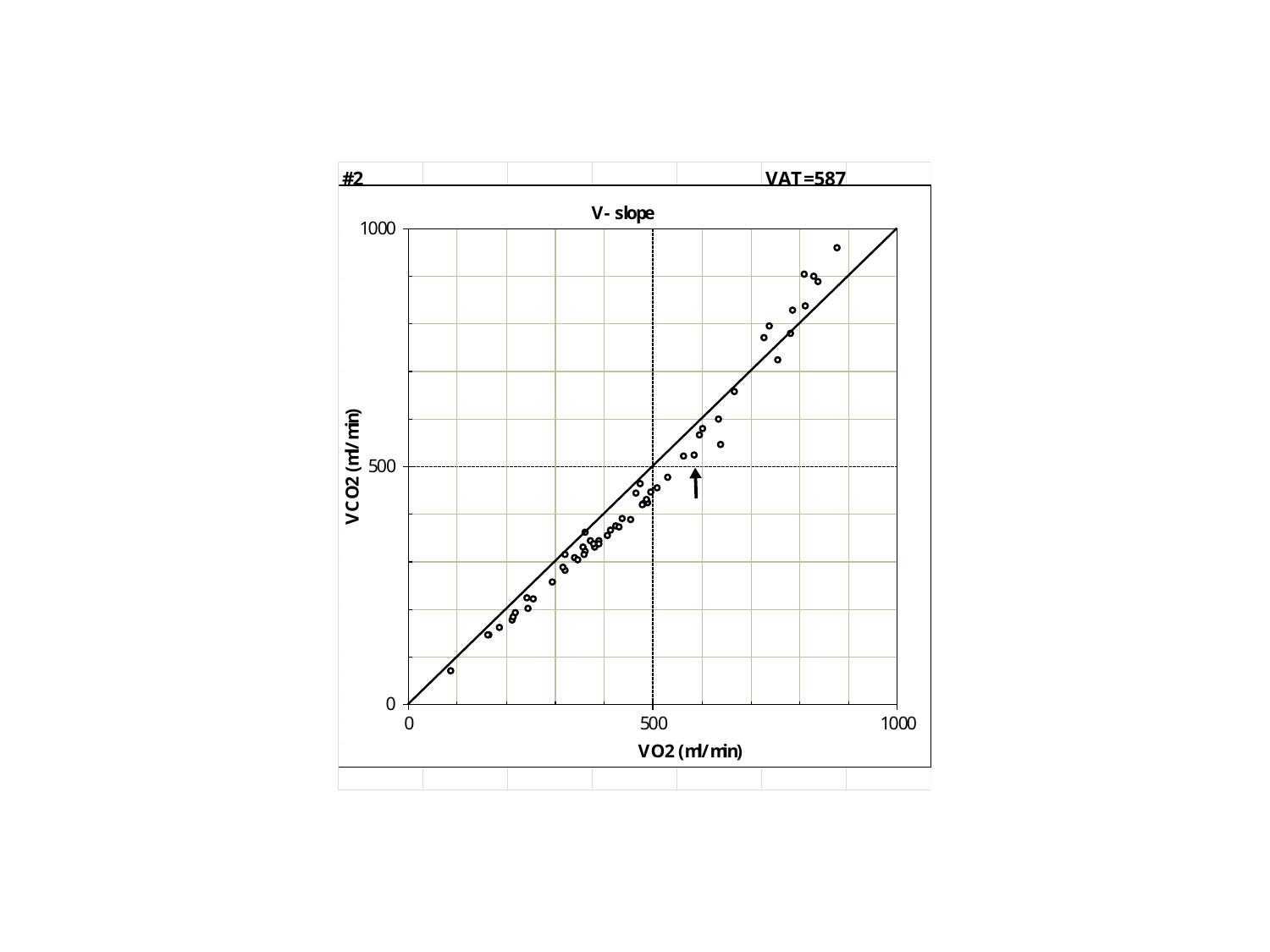

## Slide 5
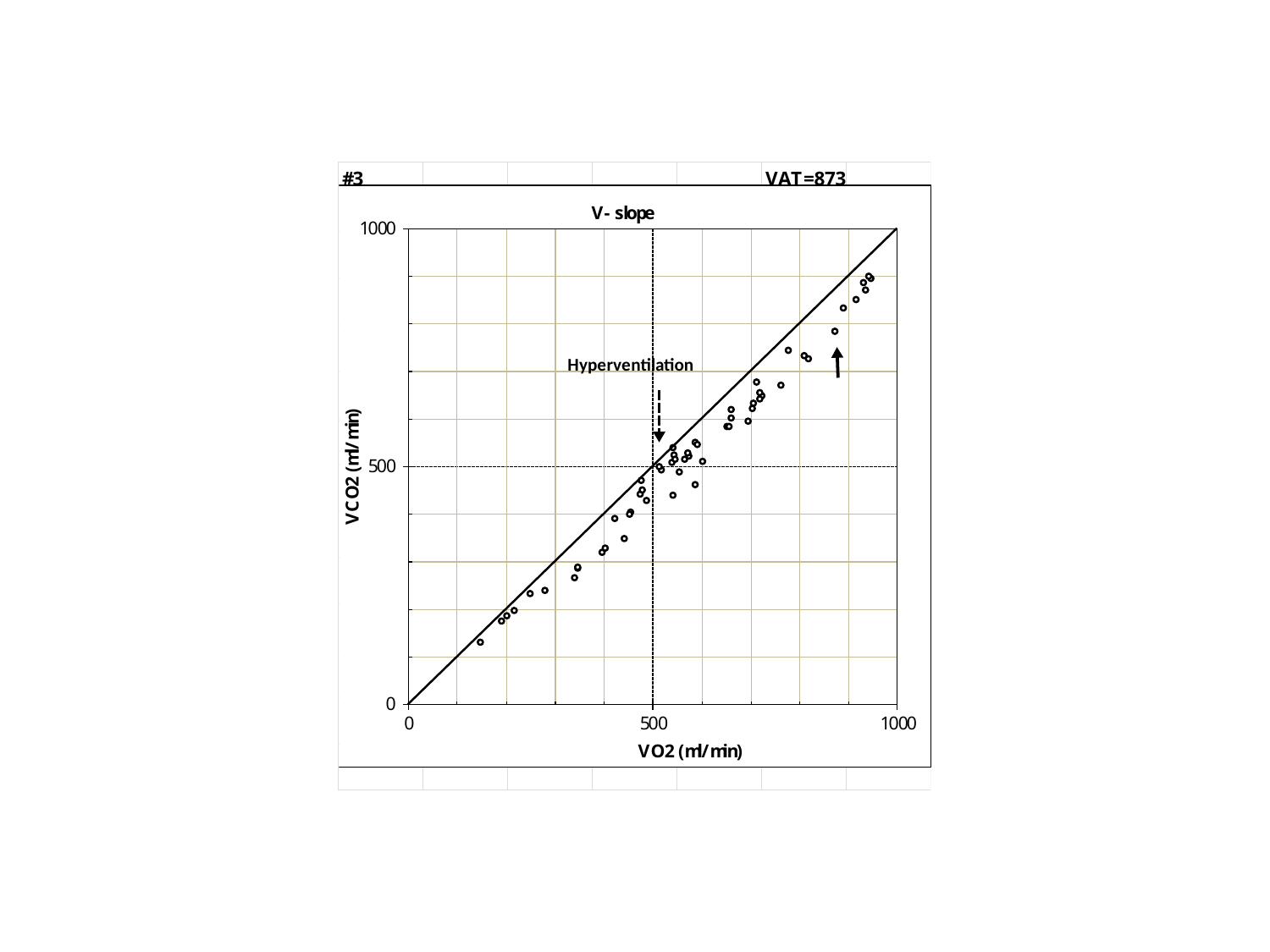

## Slide 6
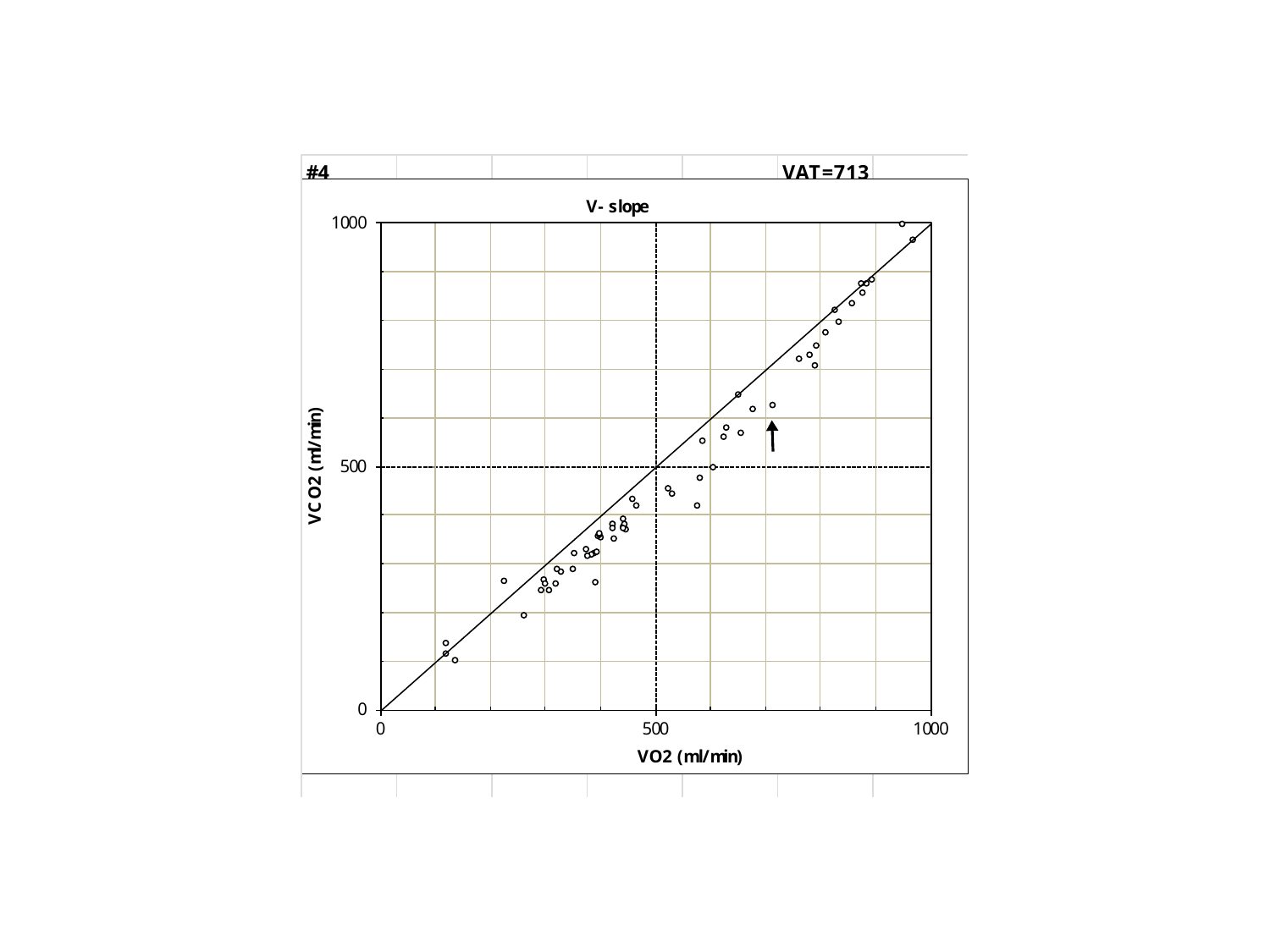

## Slide 7
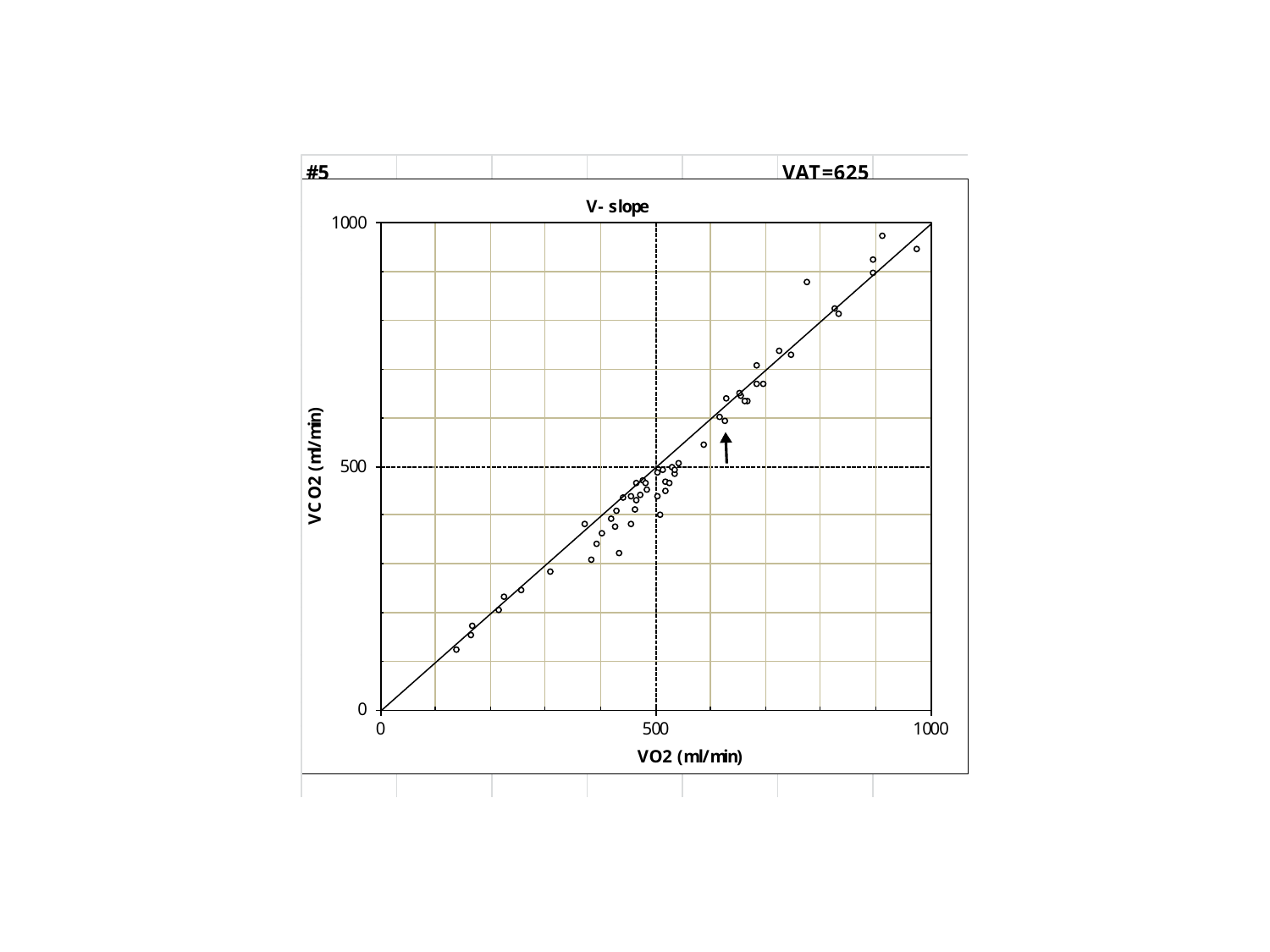

## Slide 8
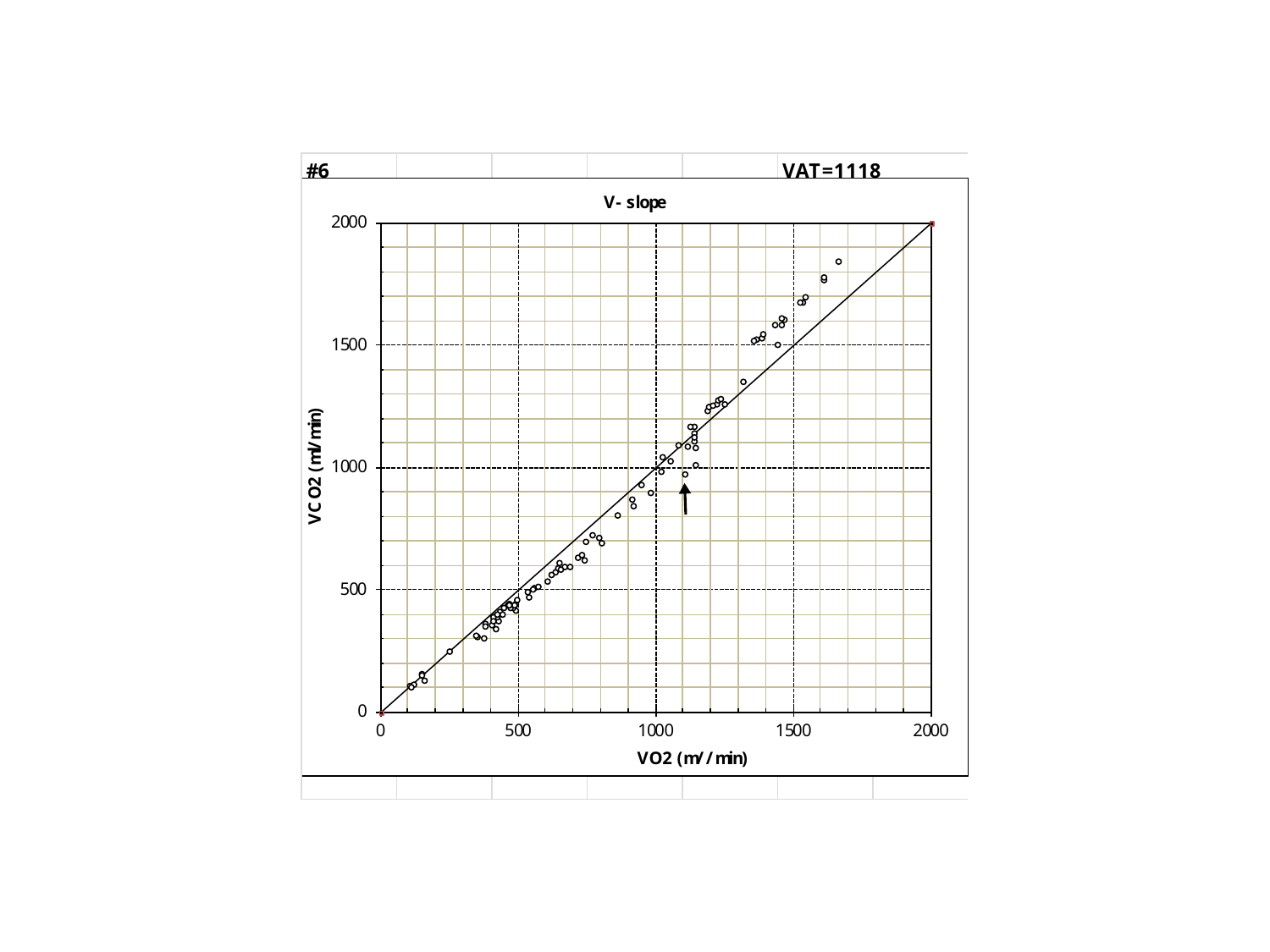

## Slide 9
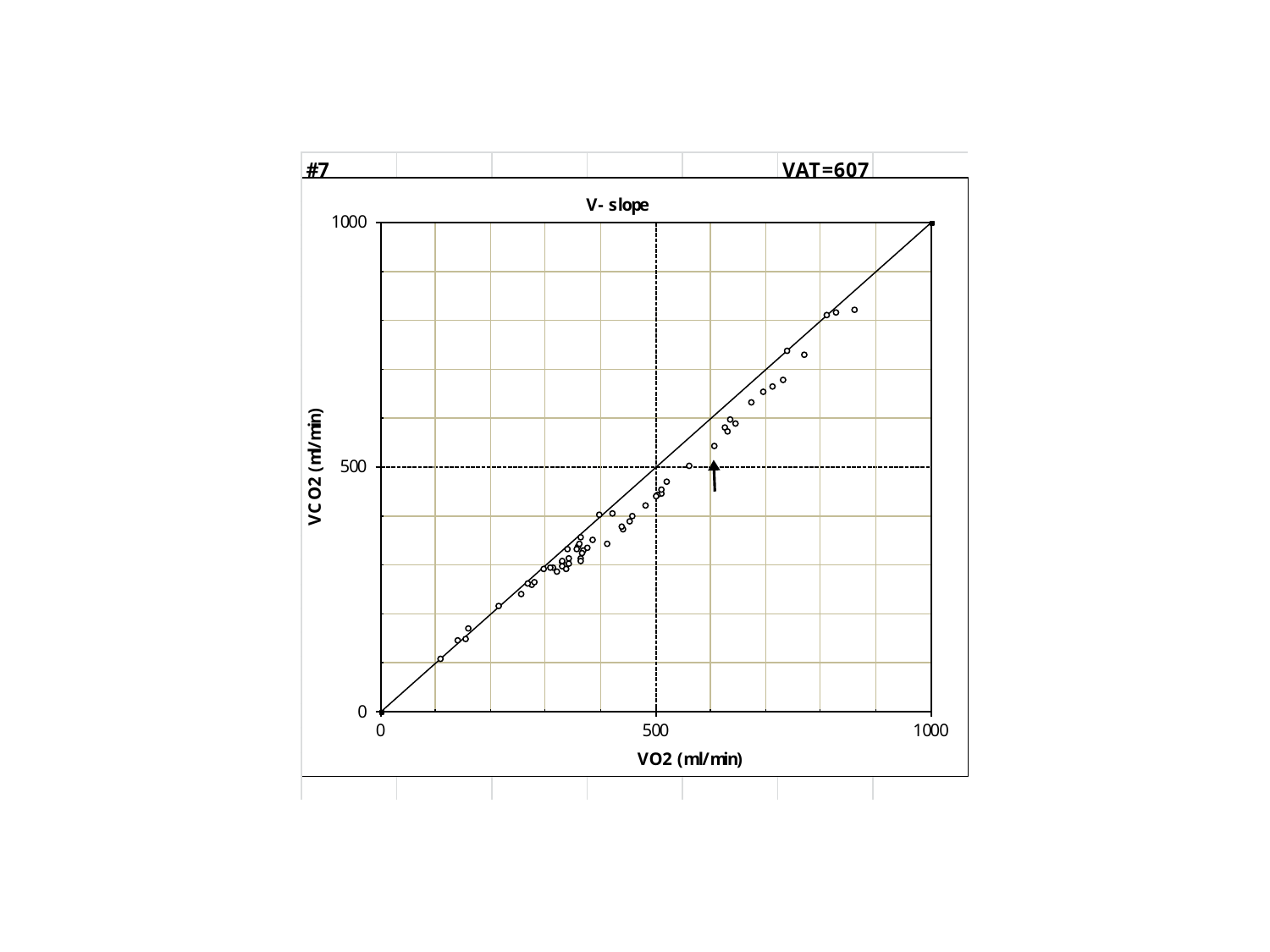

## Slide 10
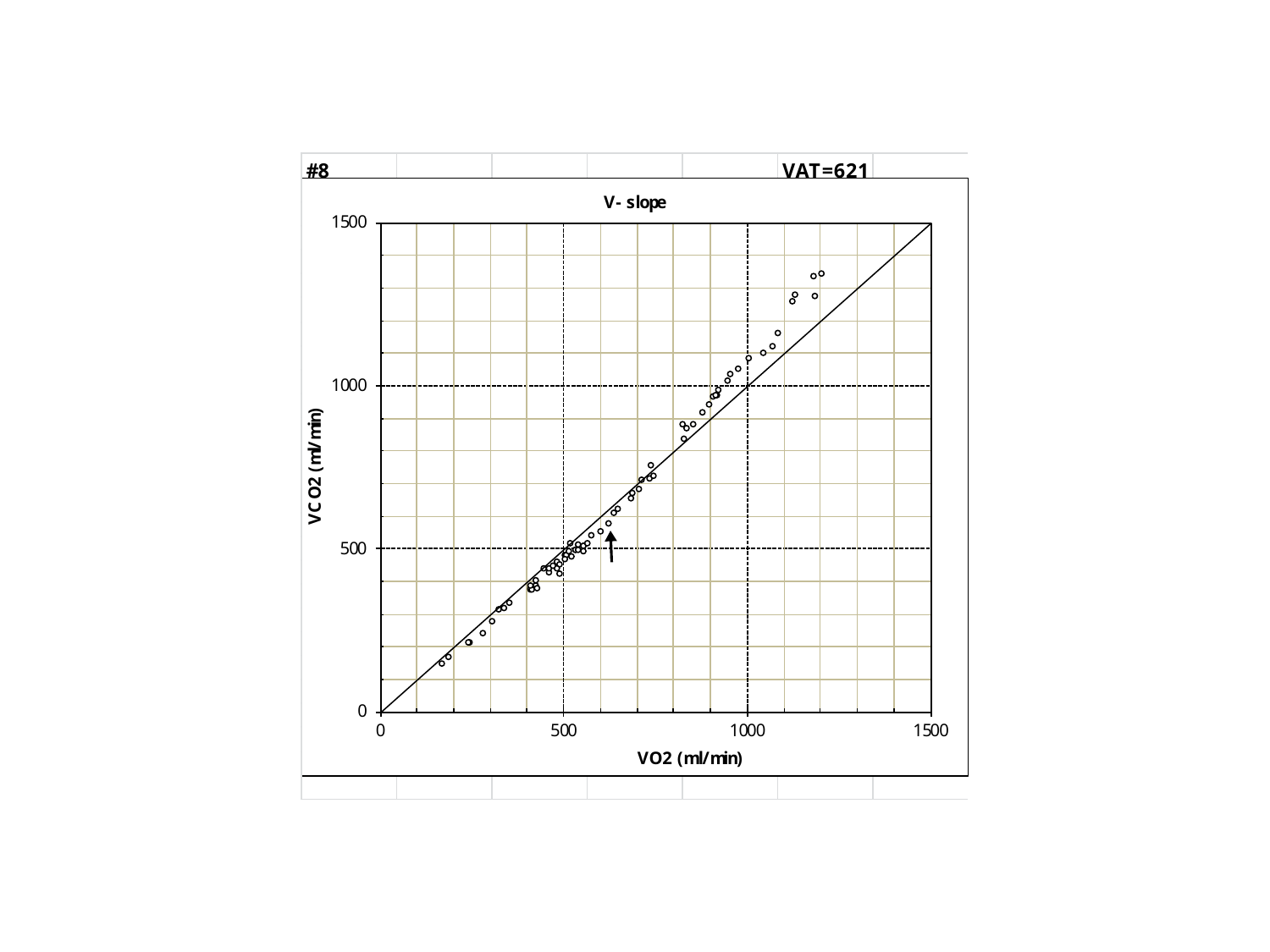

## Slide 11
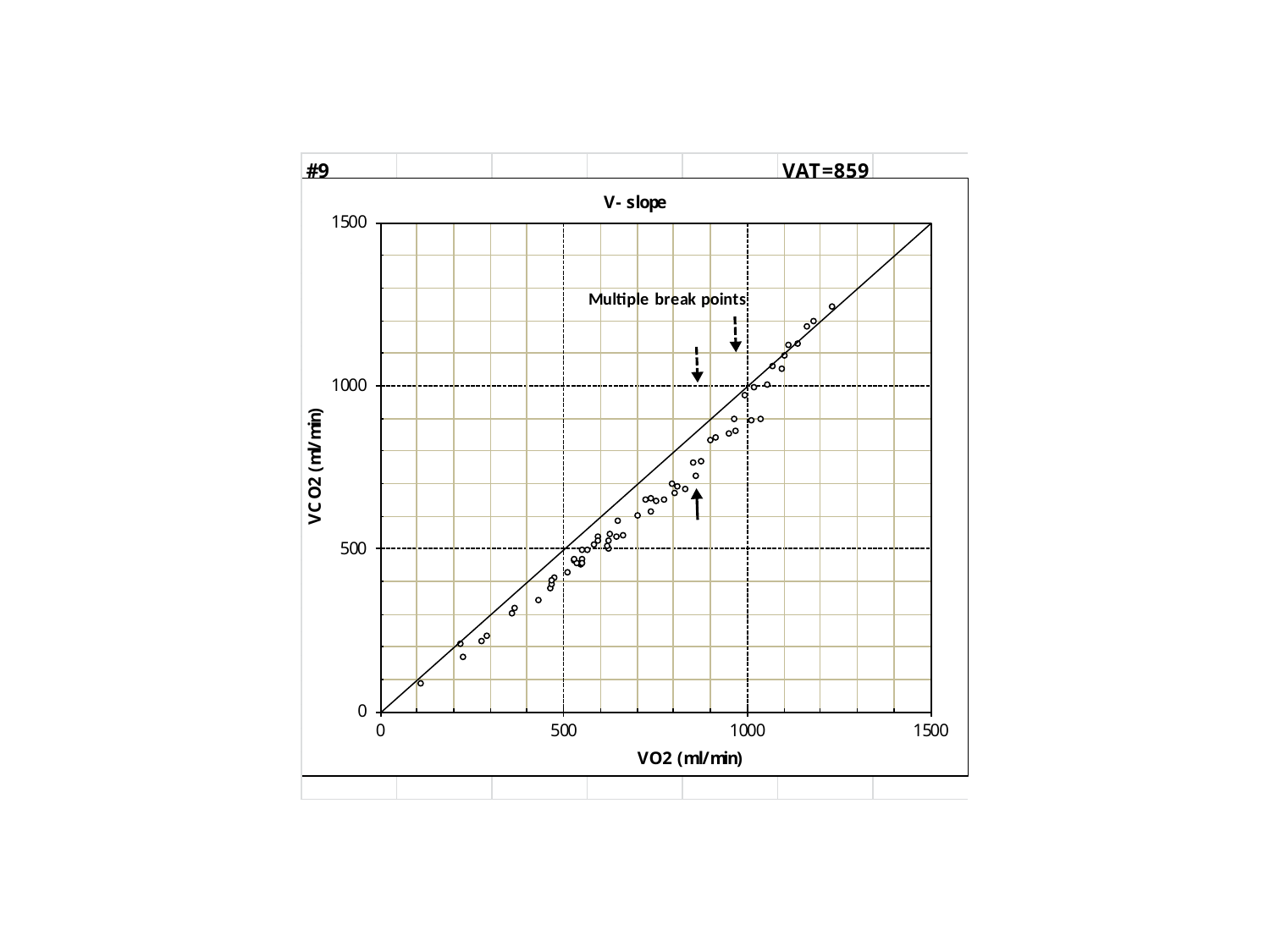

## Slide 12
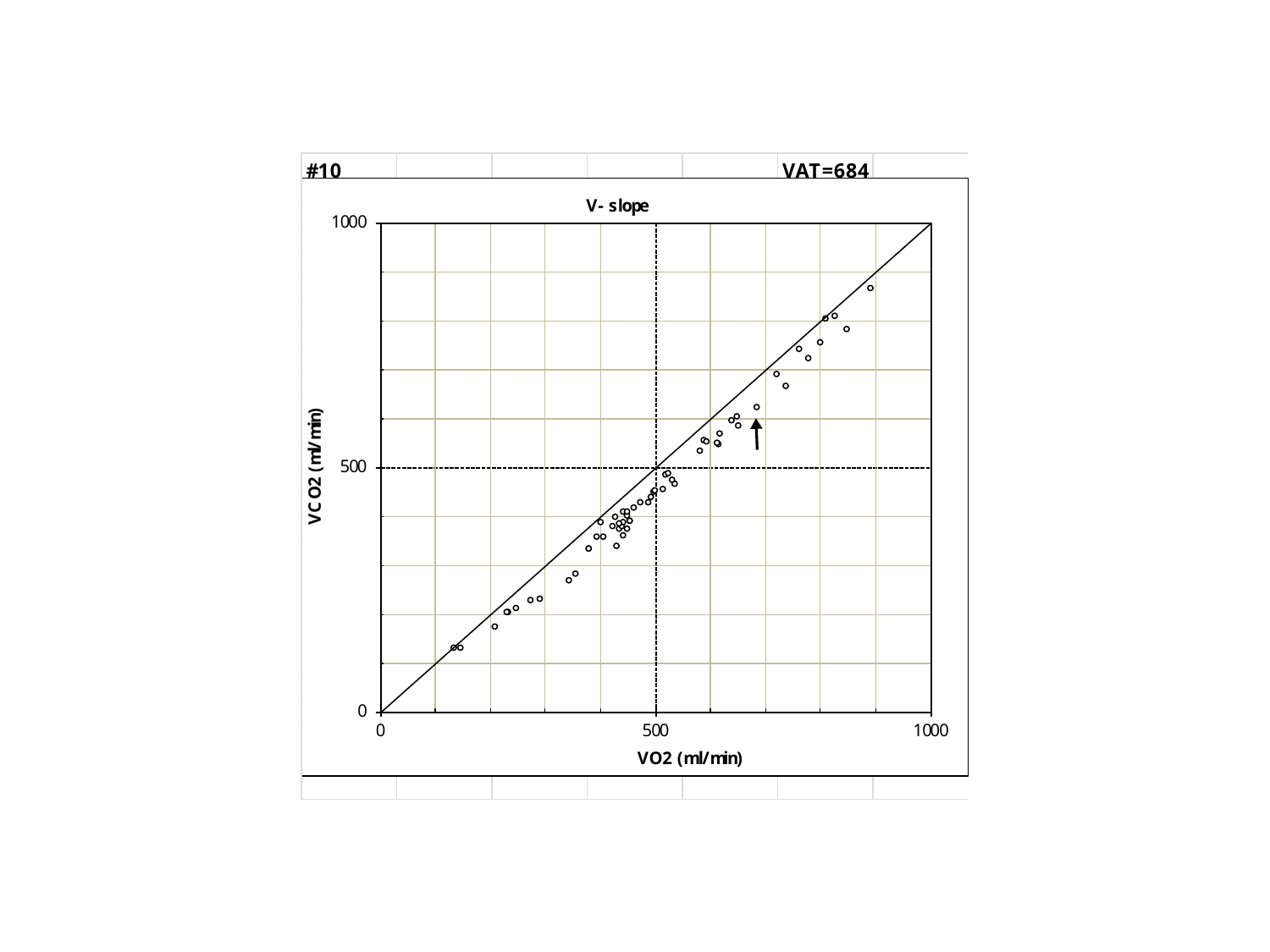

## Slide 13
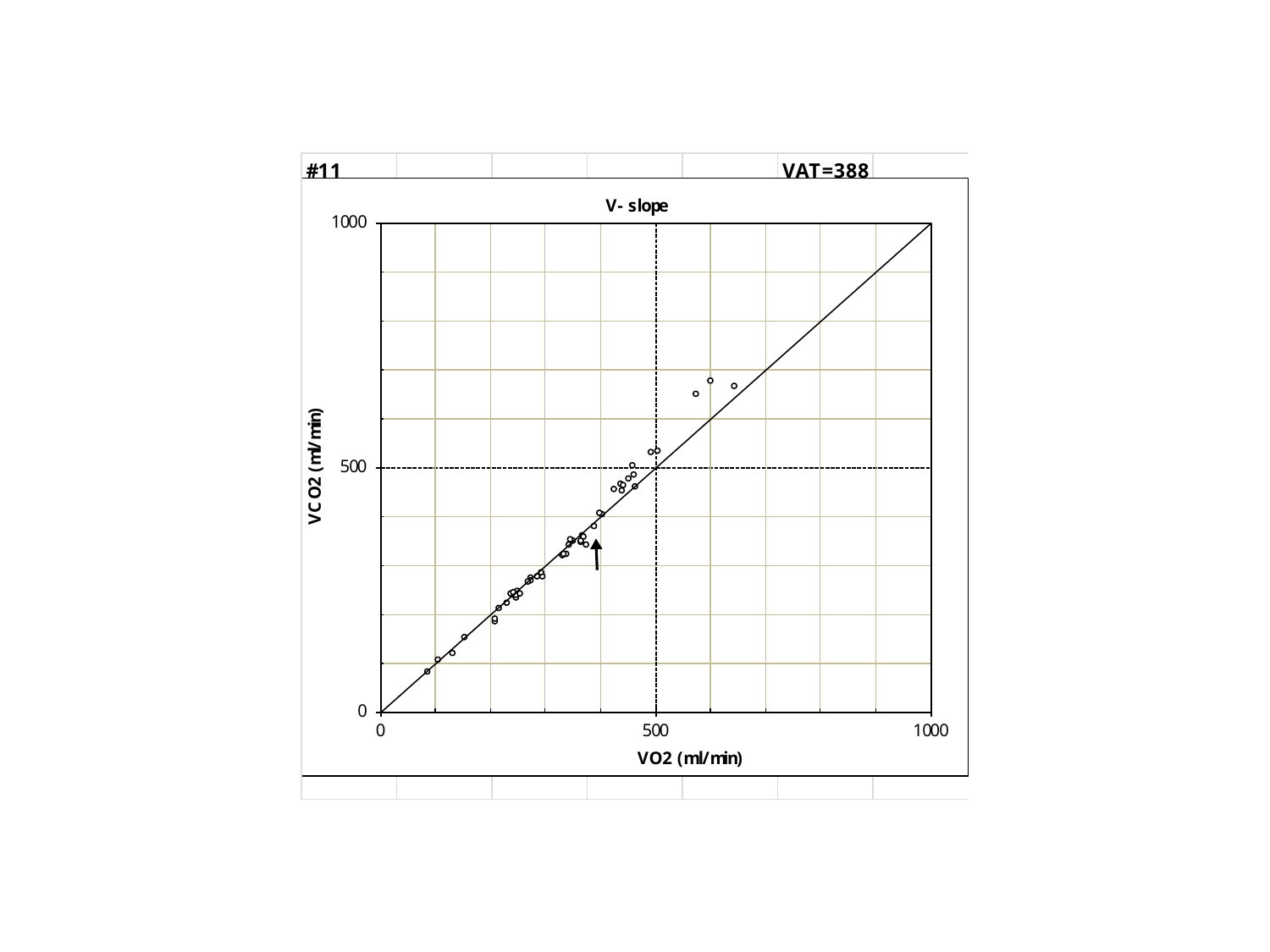

## Slide 14
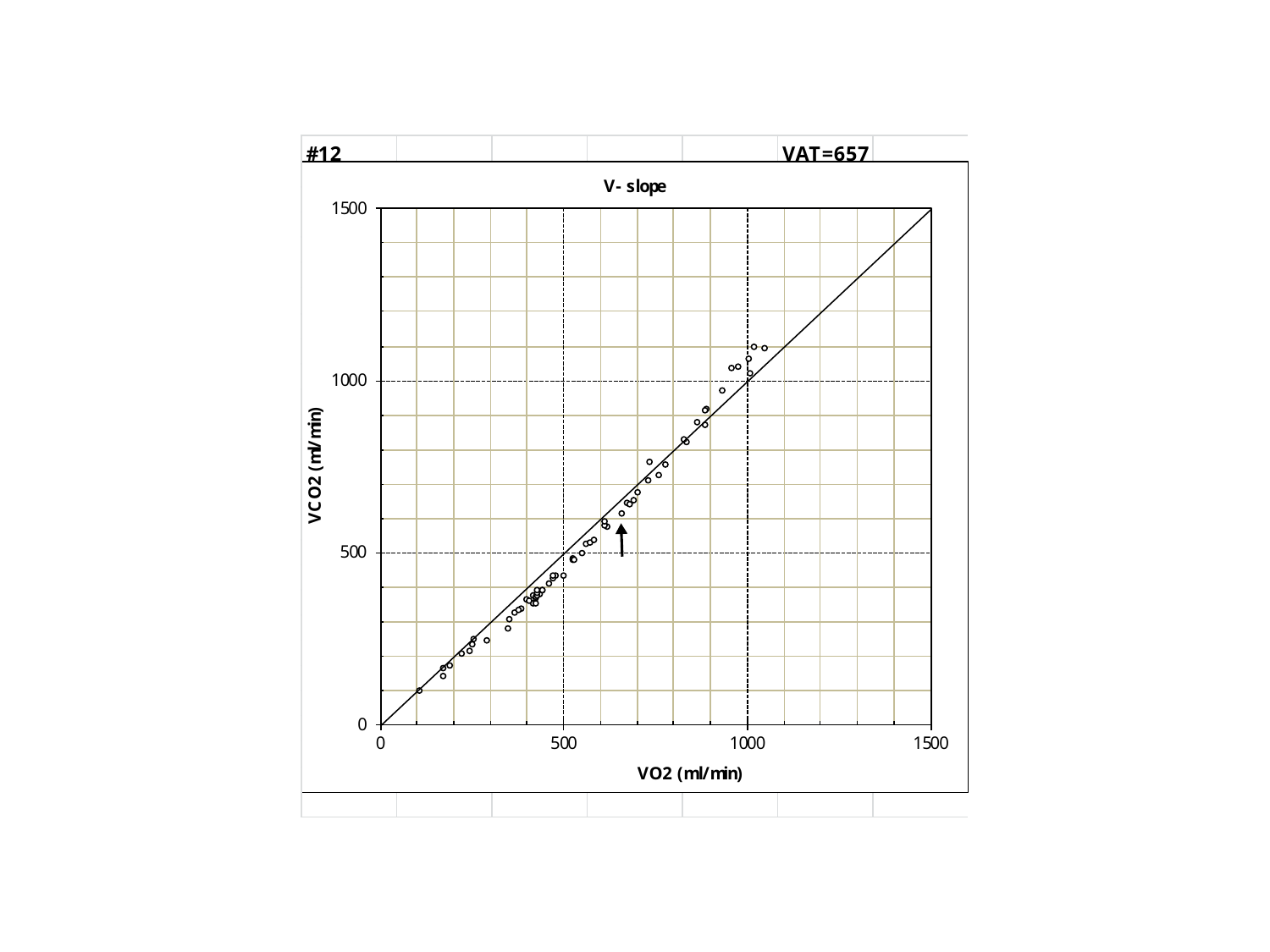

## Slide 15
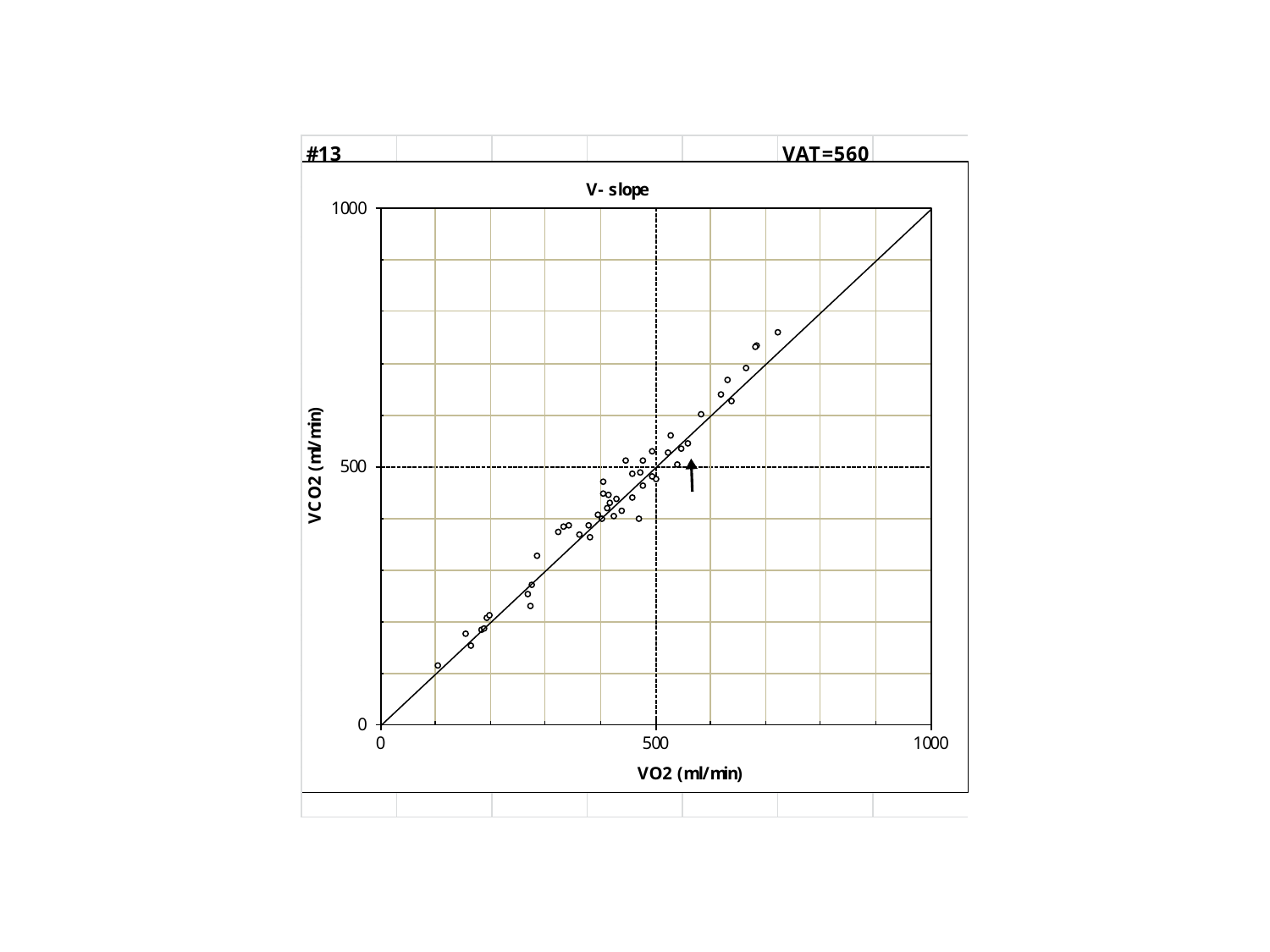

## Slide 16
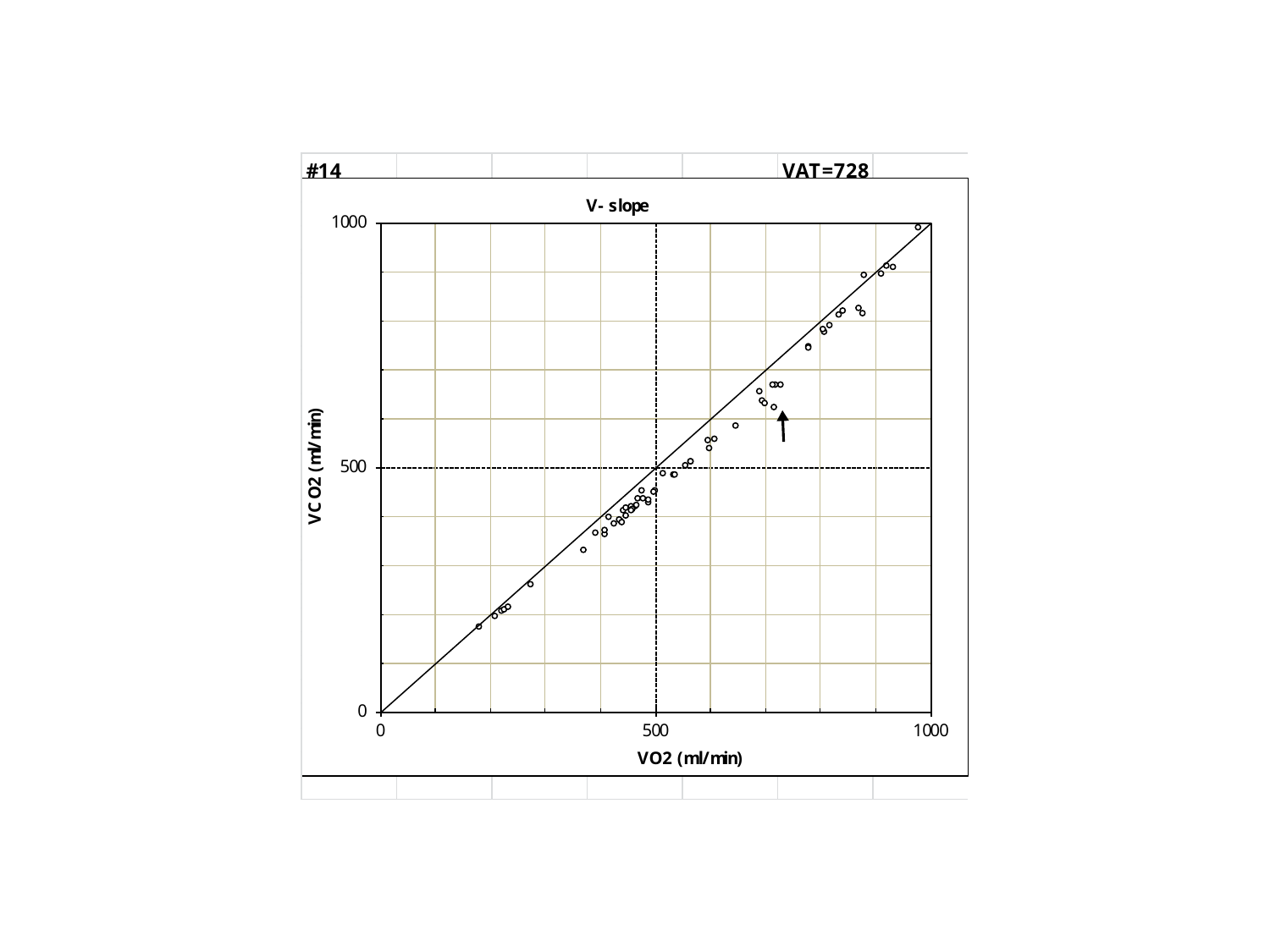

## Slide 17
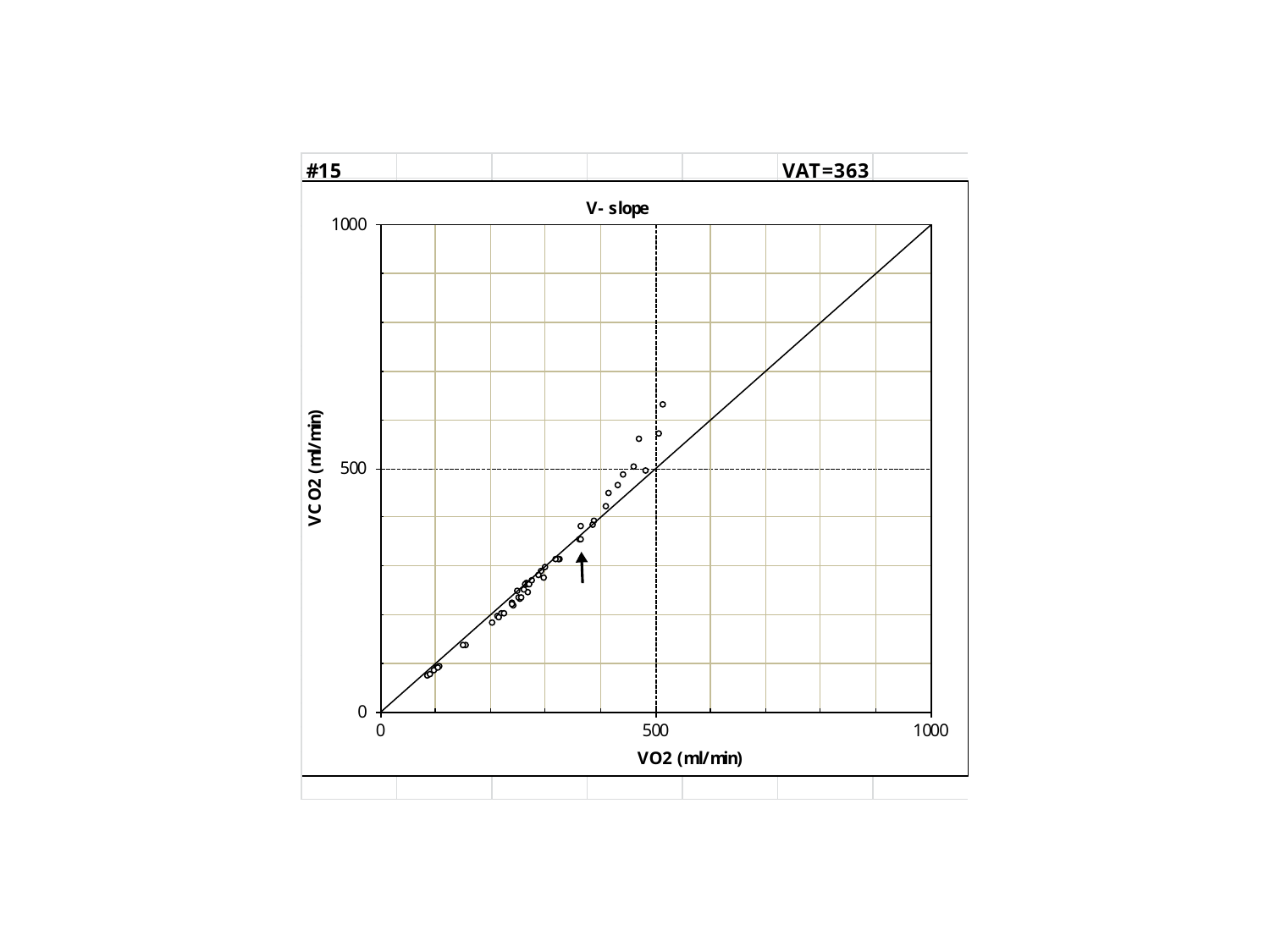

## Slide 18
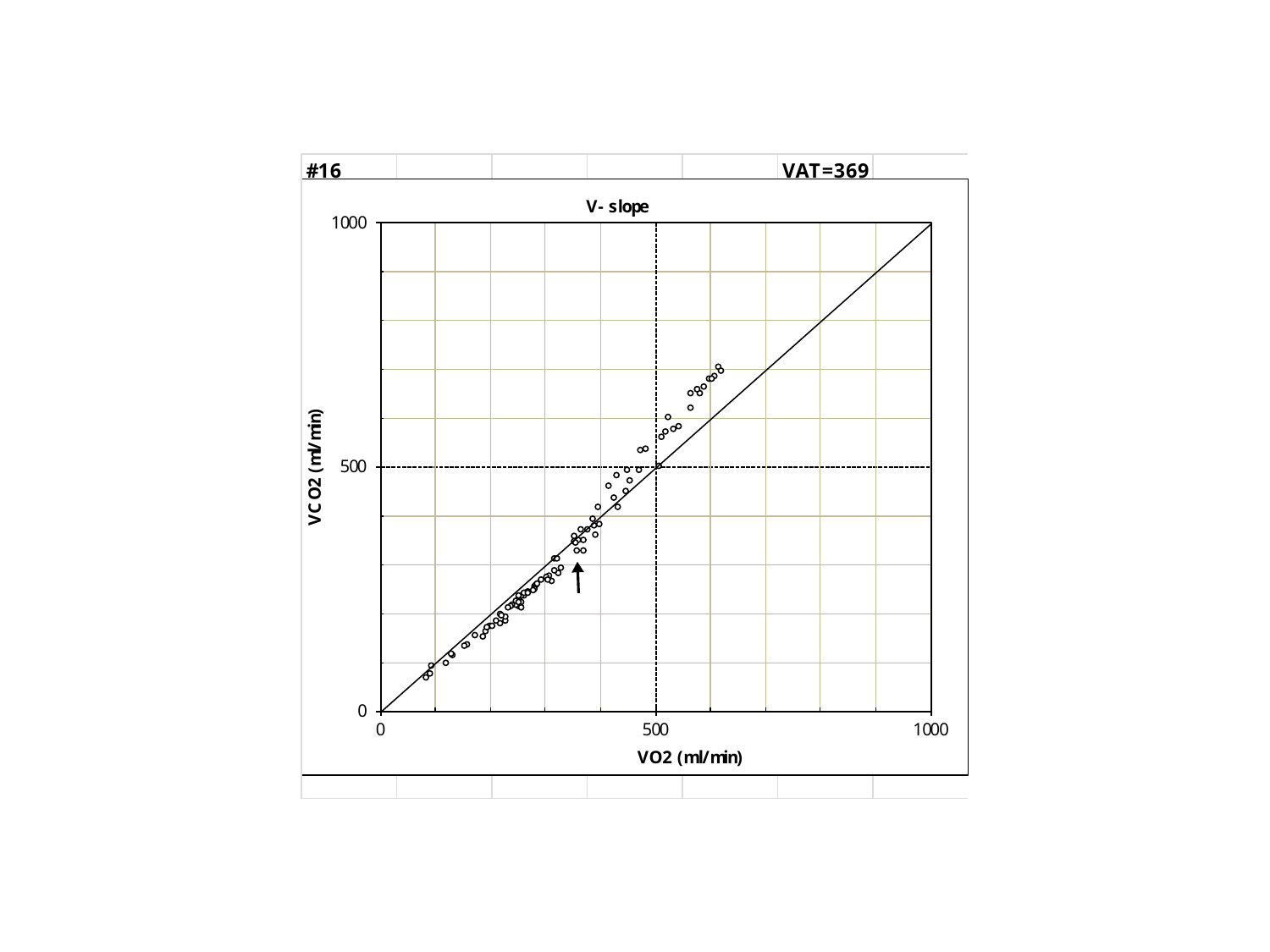

## Slide 19
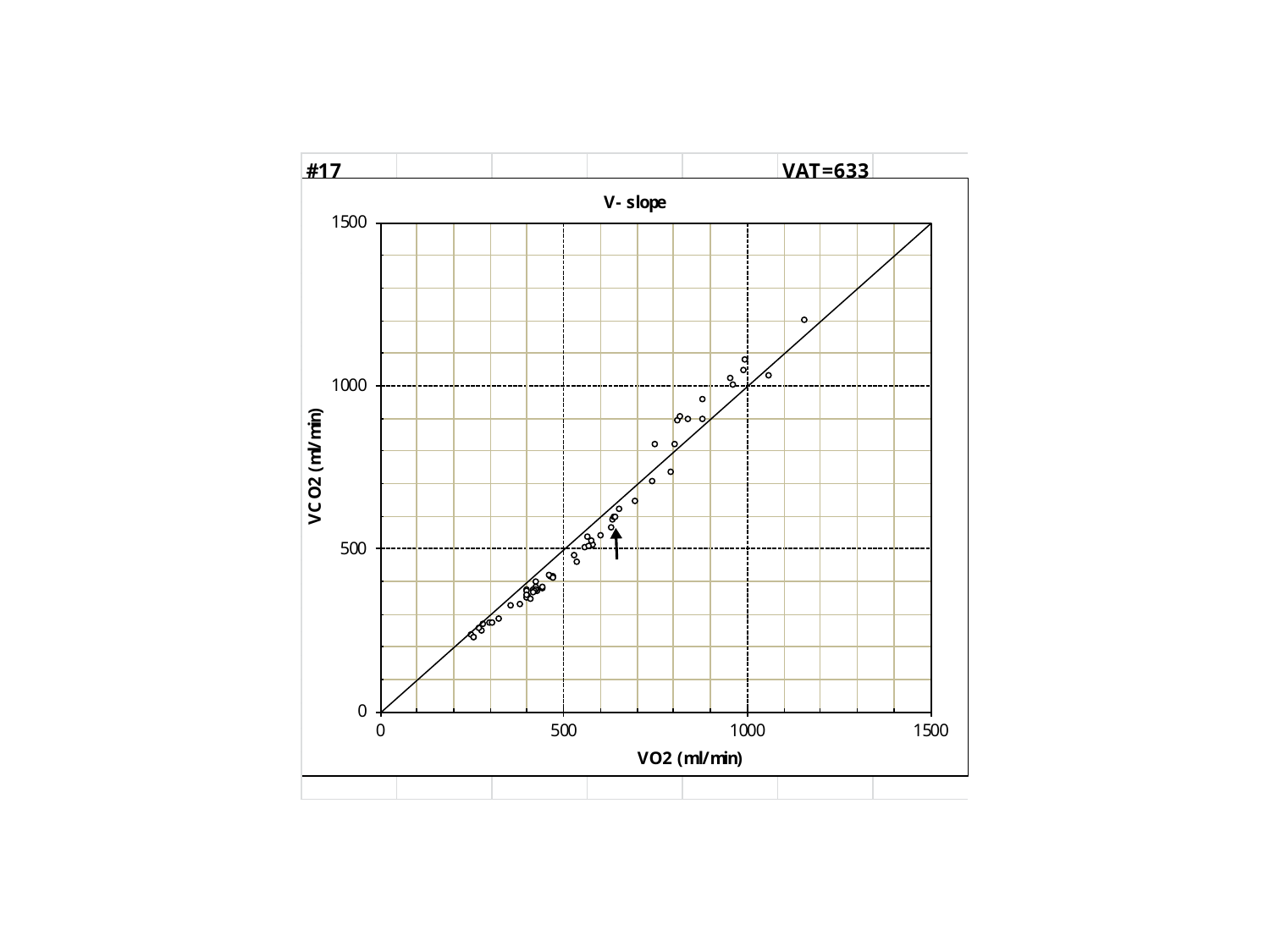

## Slide 20
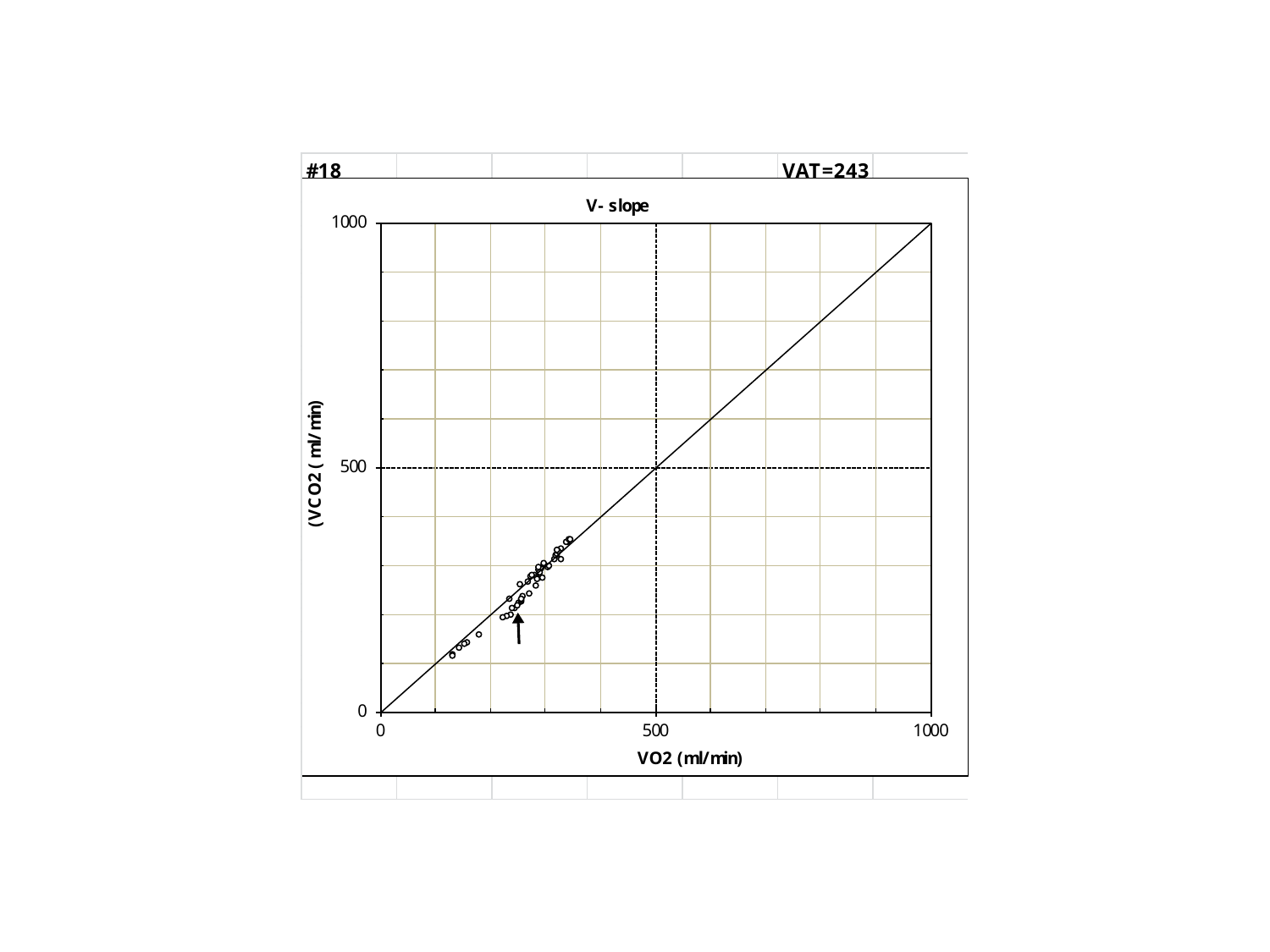

## Slide 21
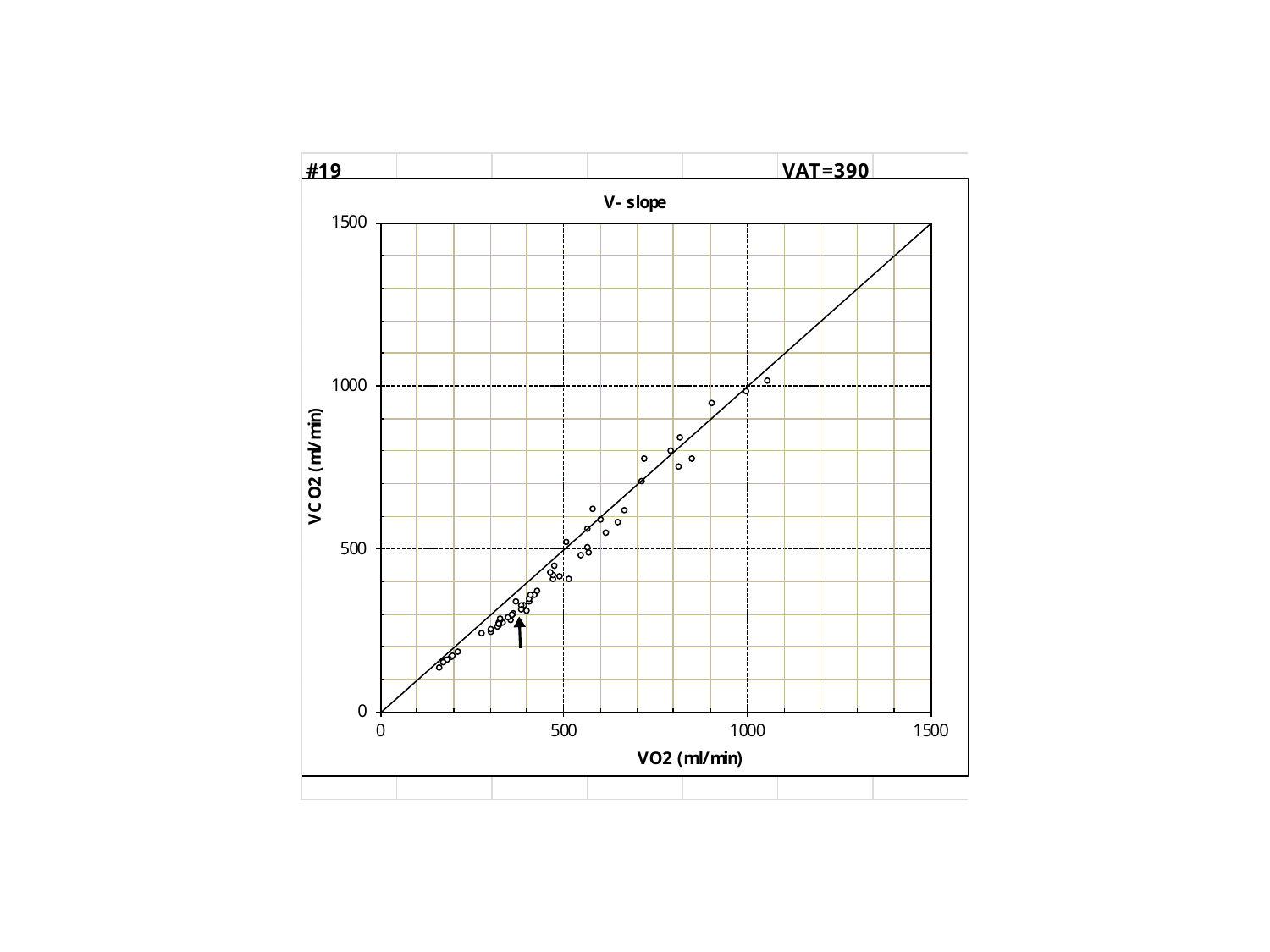

## Slide 22
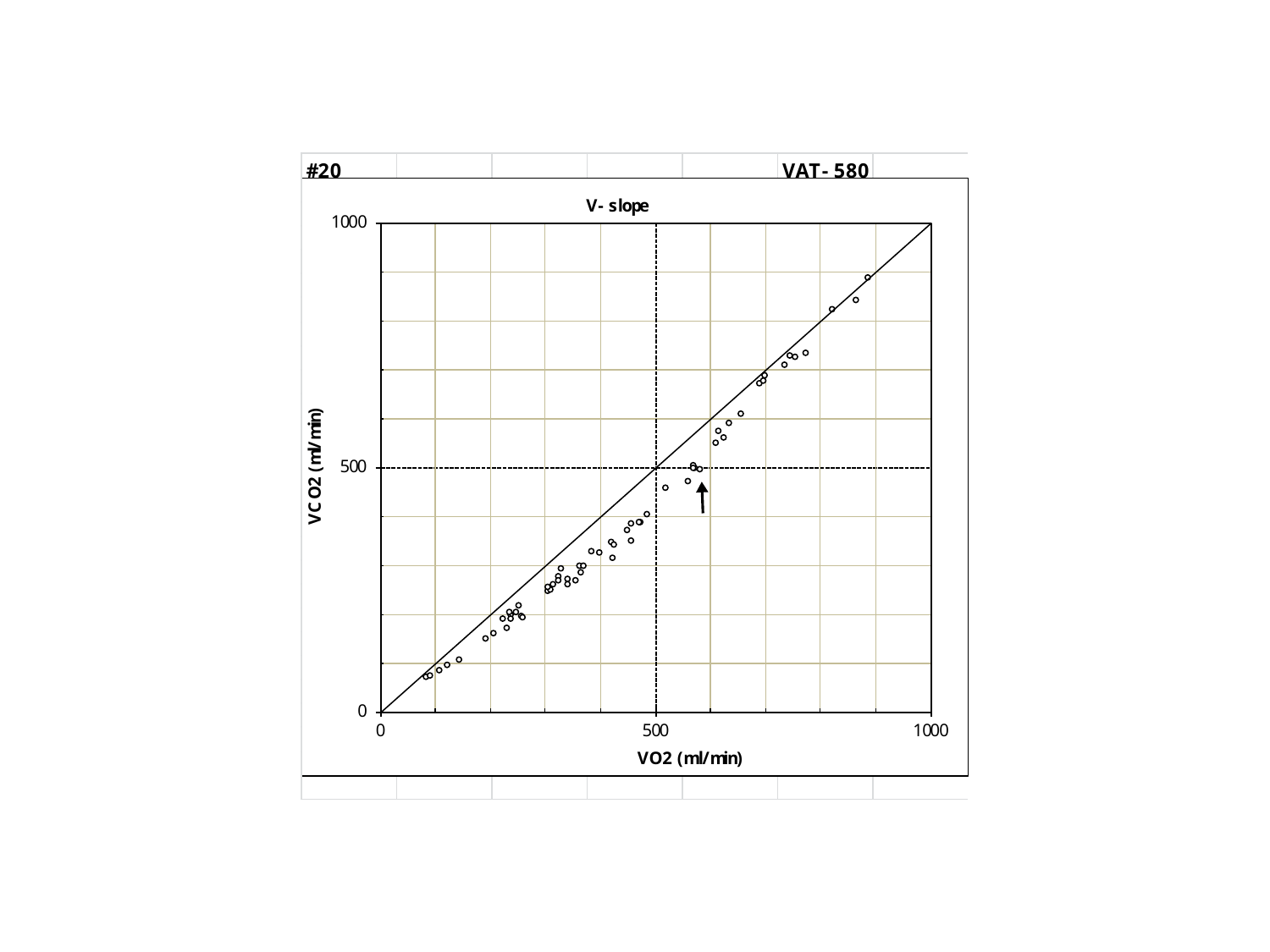

## Slide 23
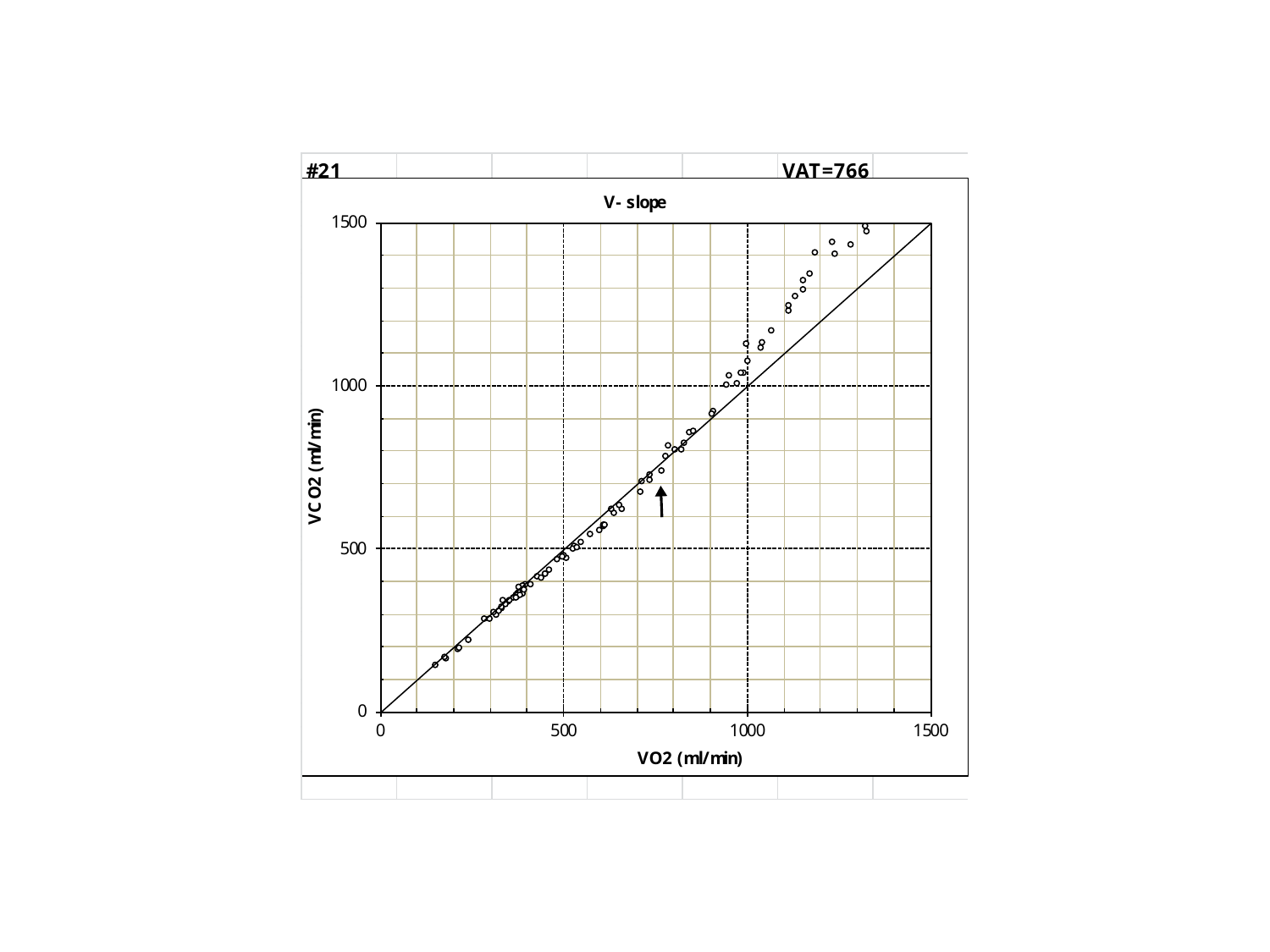

## Slide 24
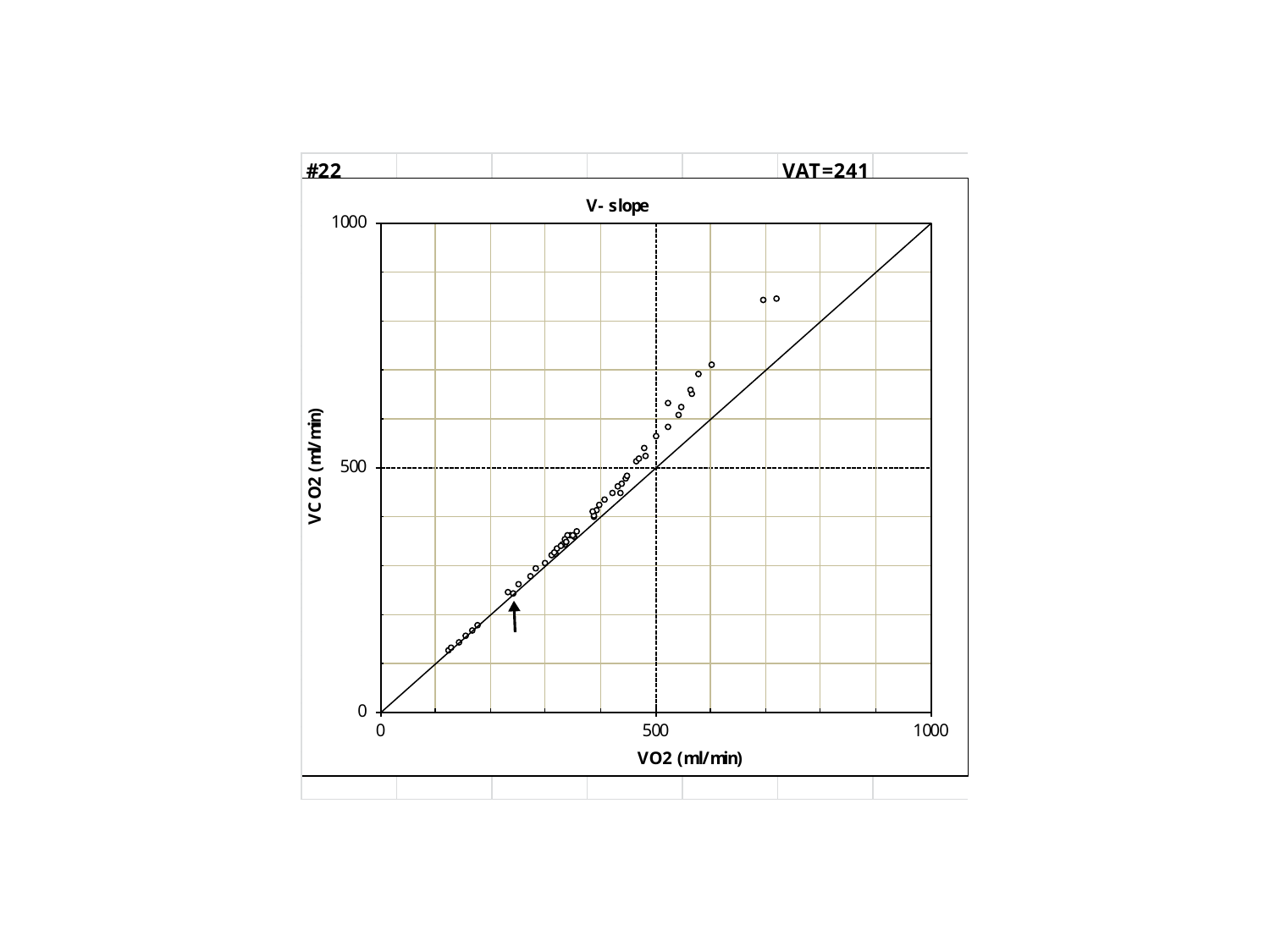

## Slide 25
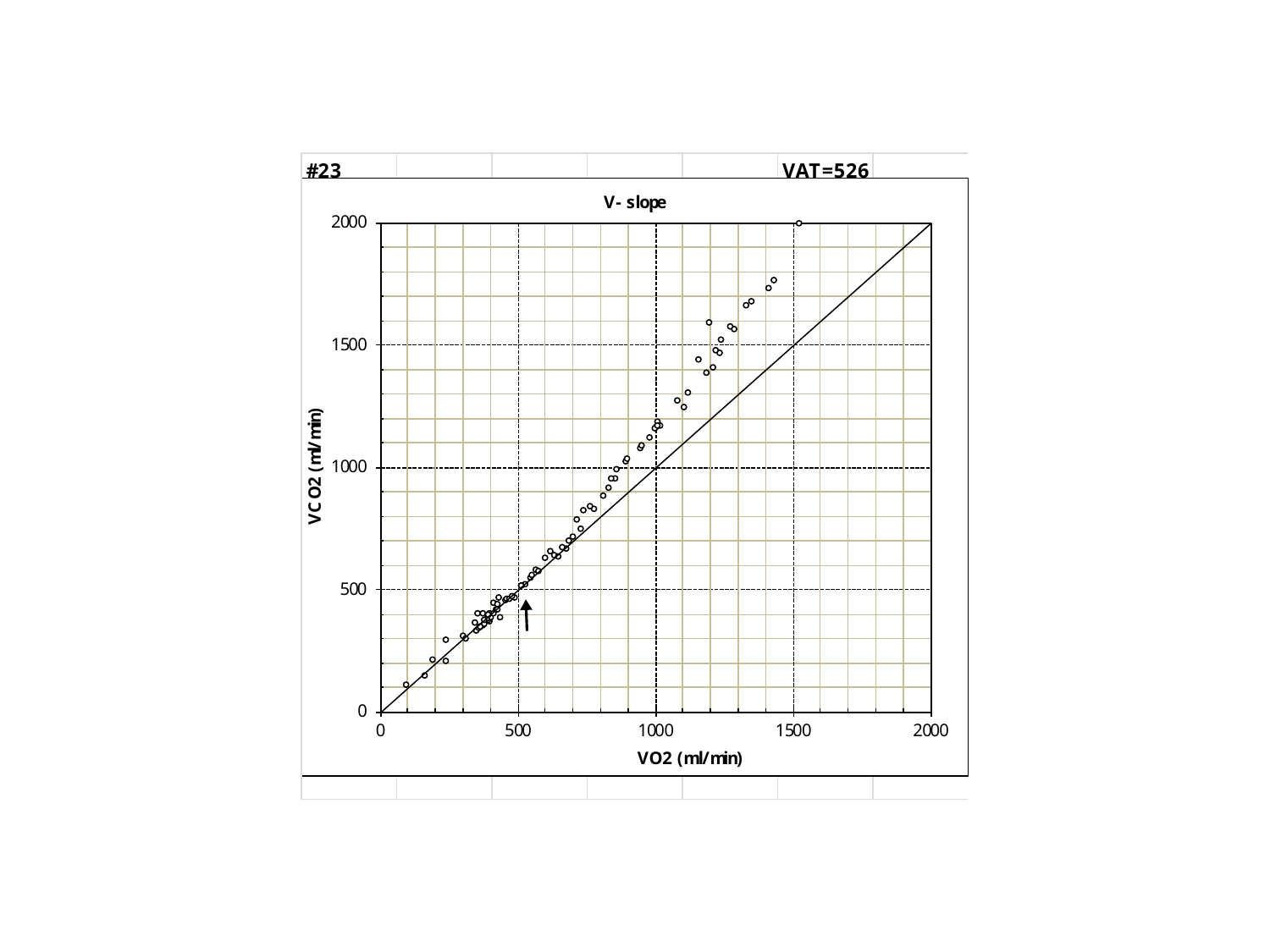

## Slide 26
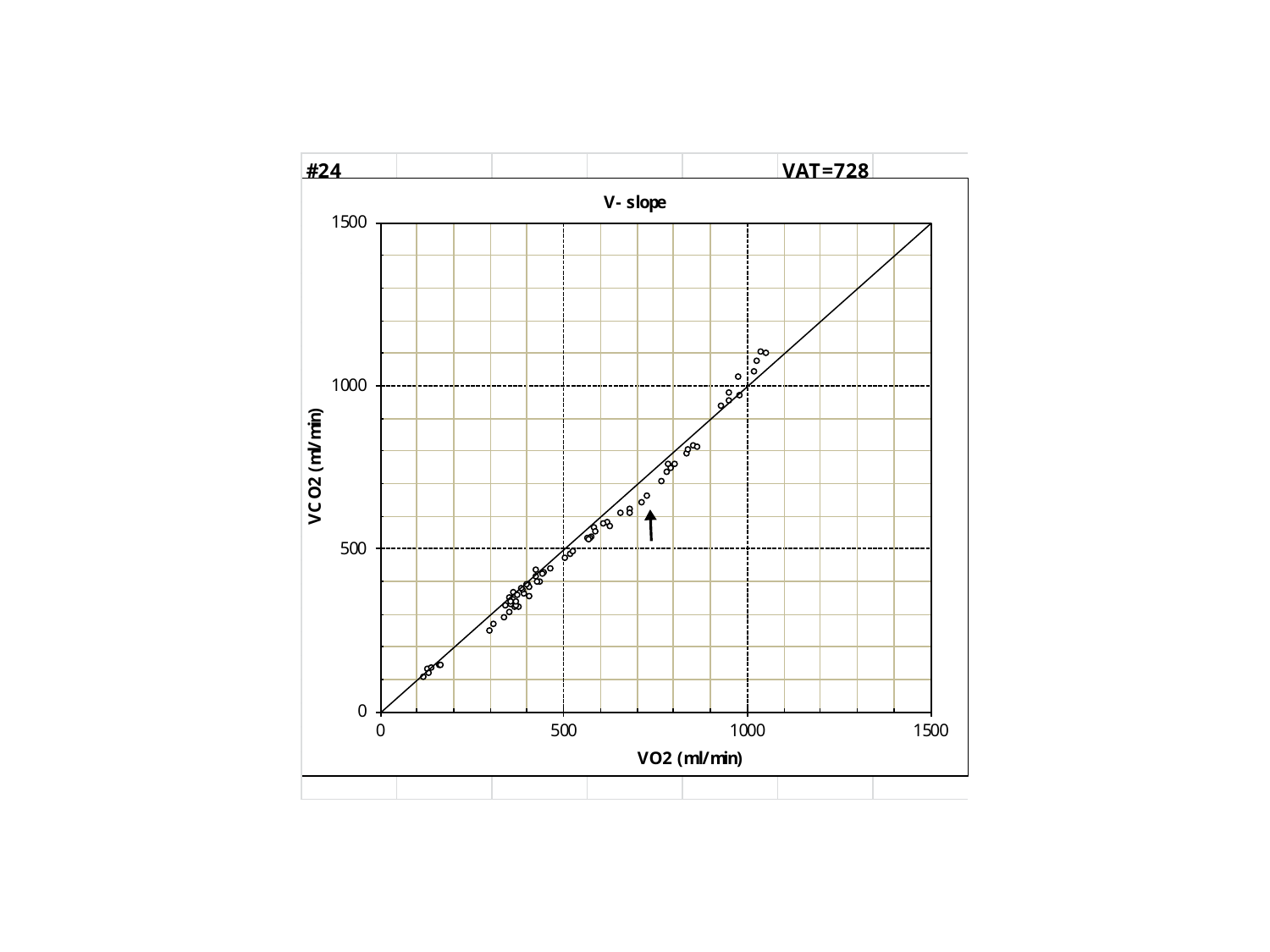

## Slide 27
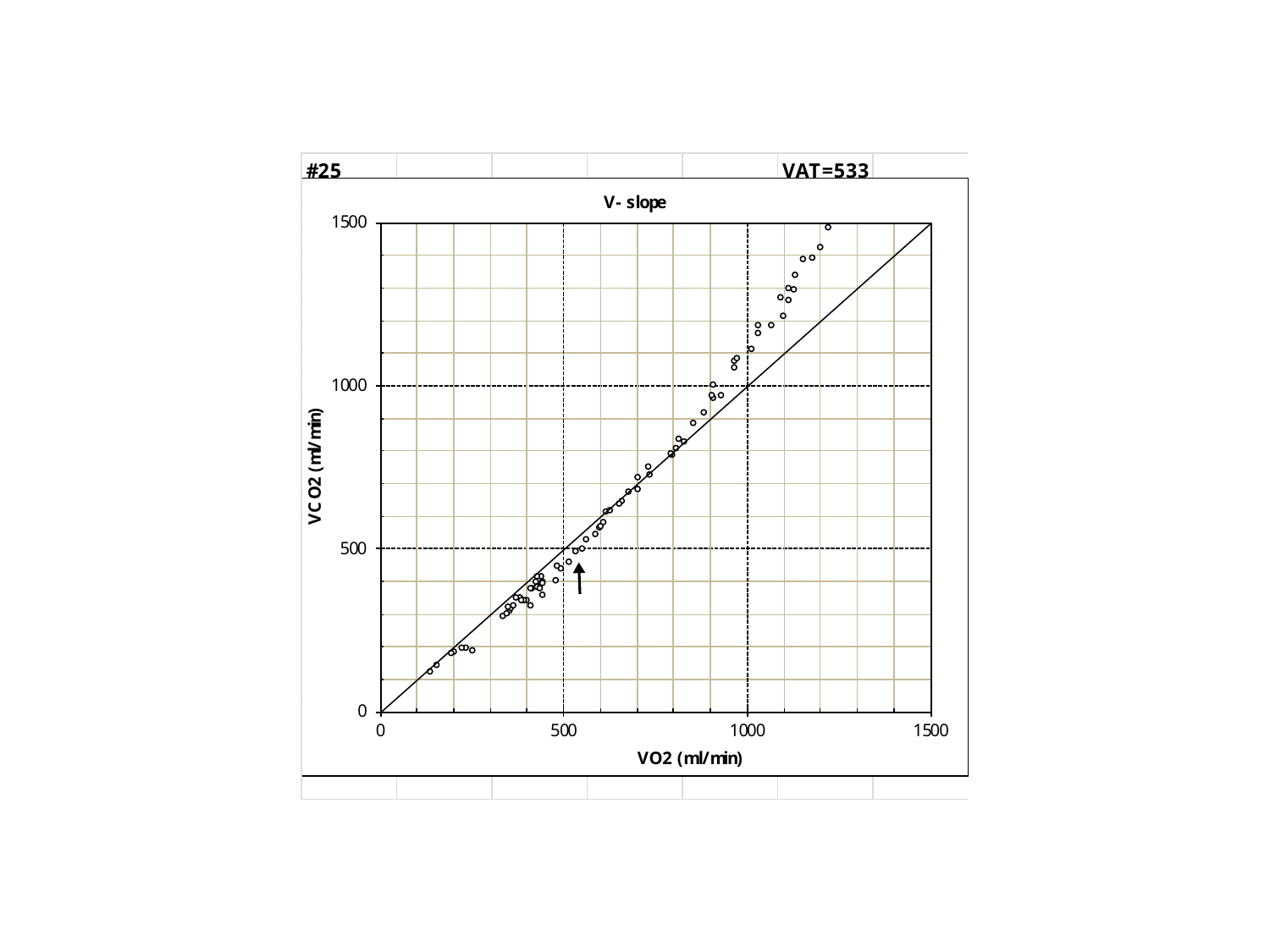

## Slide 28
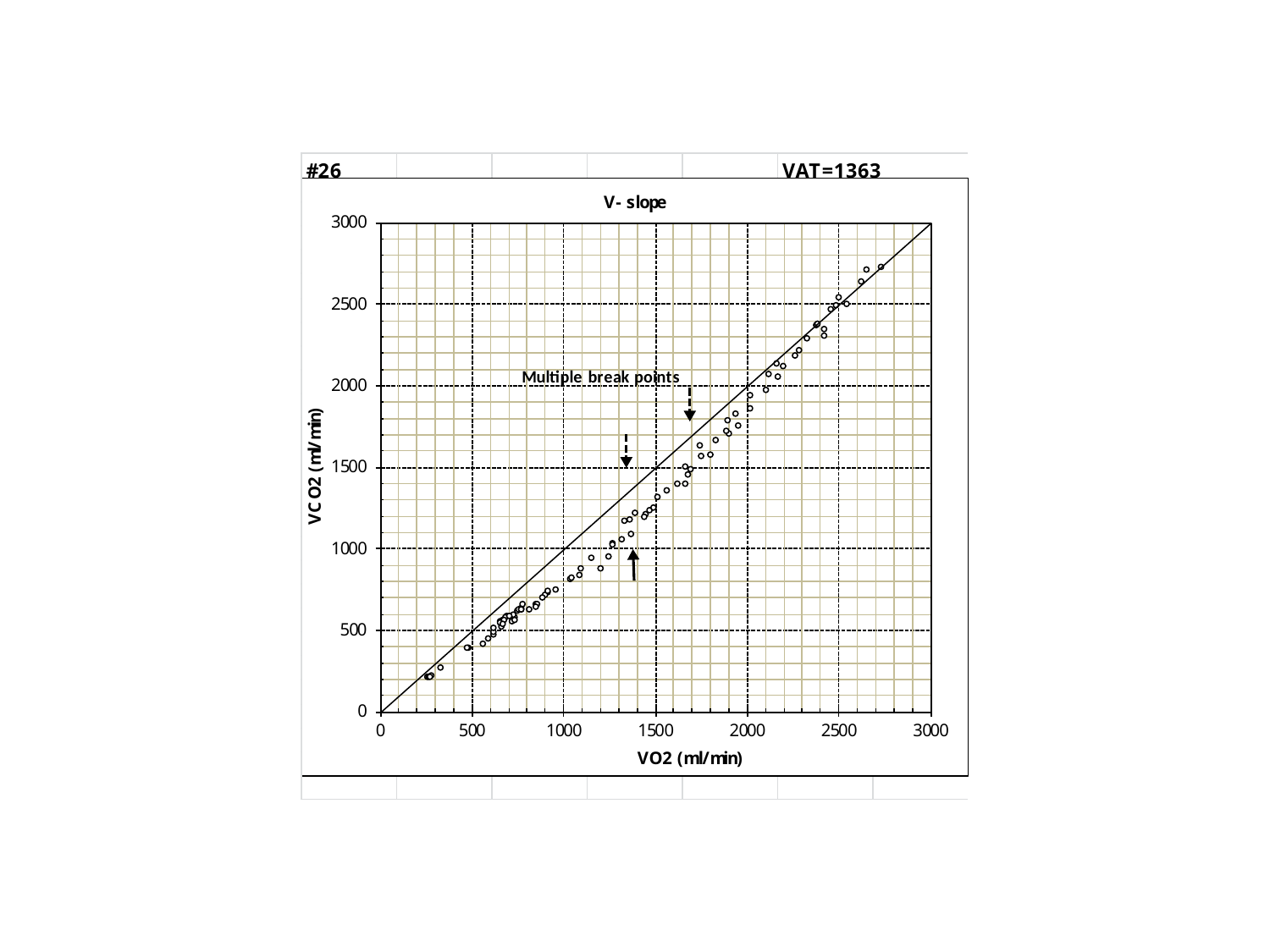

## Slide 29
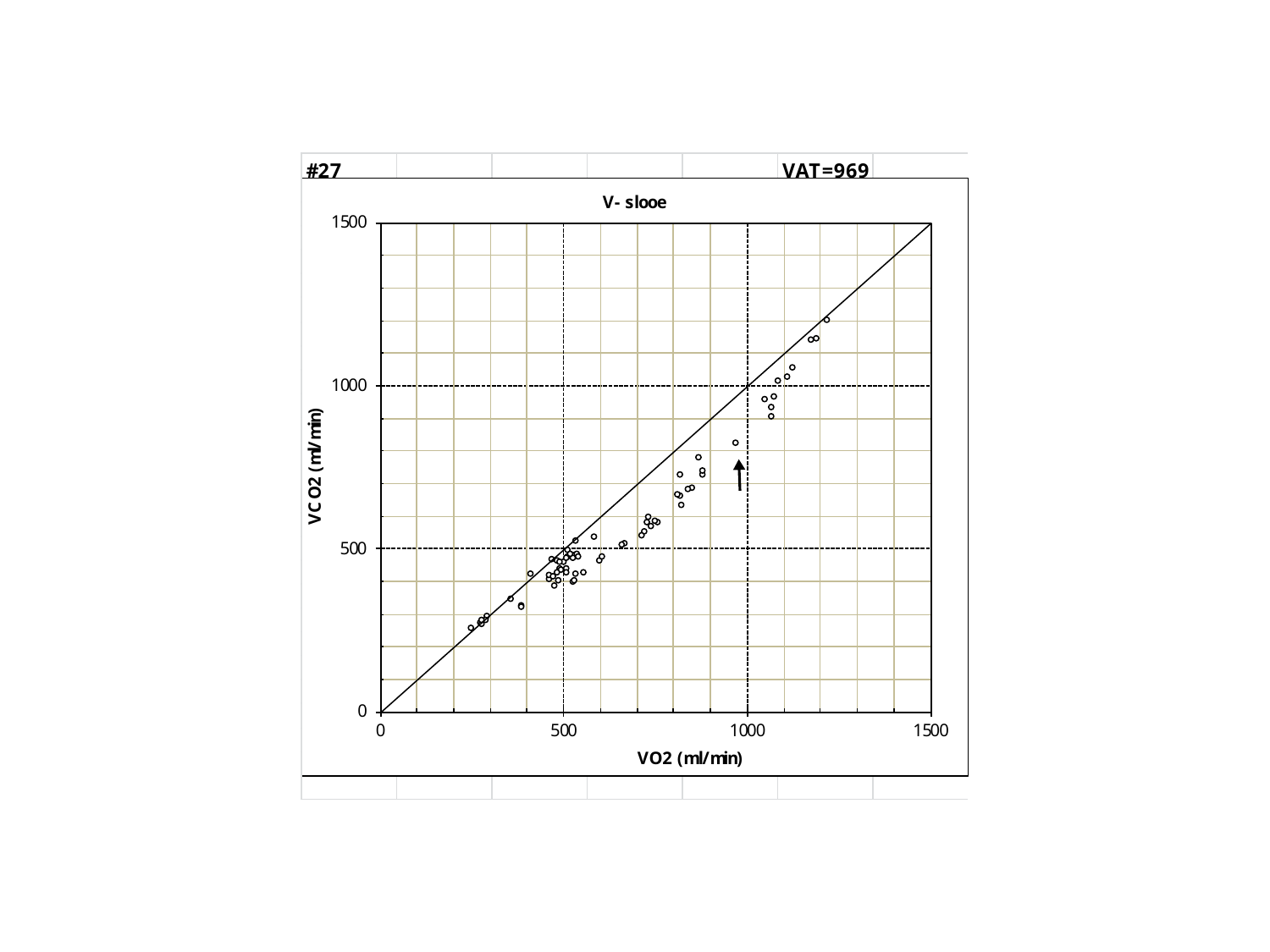

## Slide 30
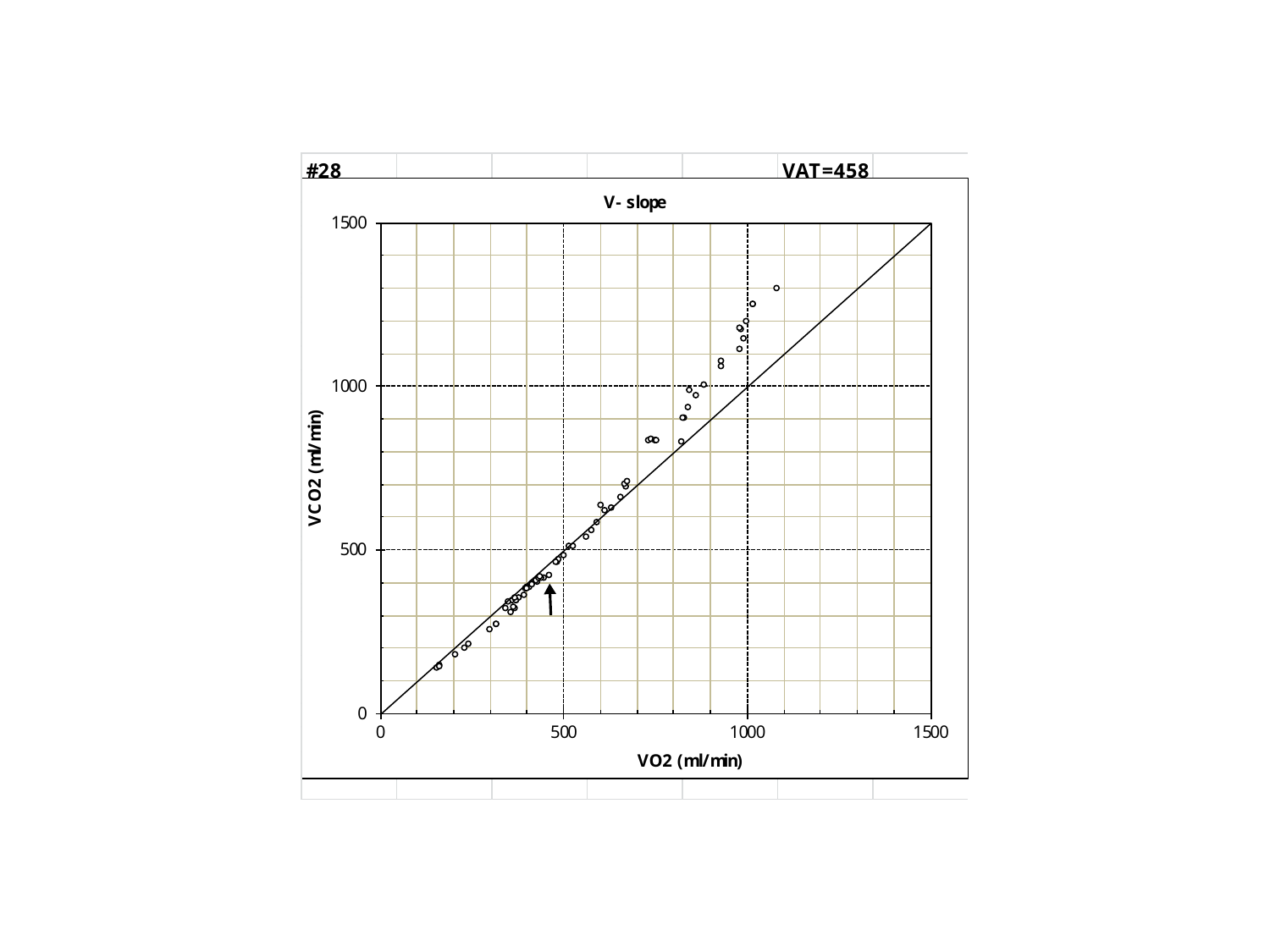

## Slide 31
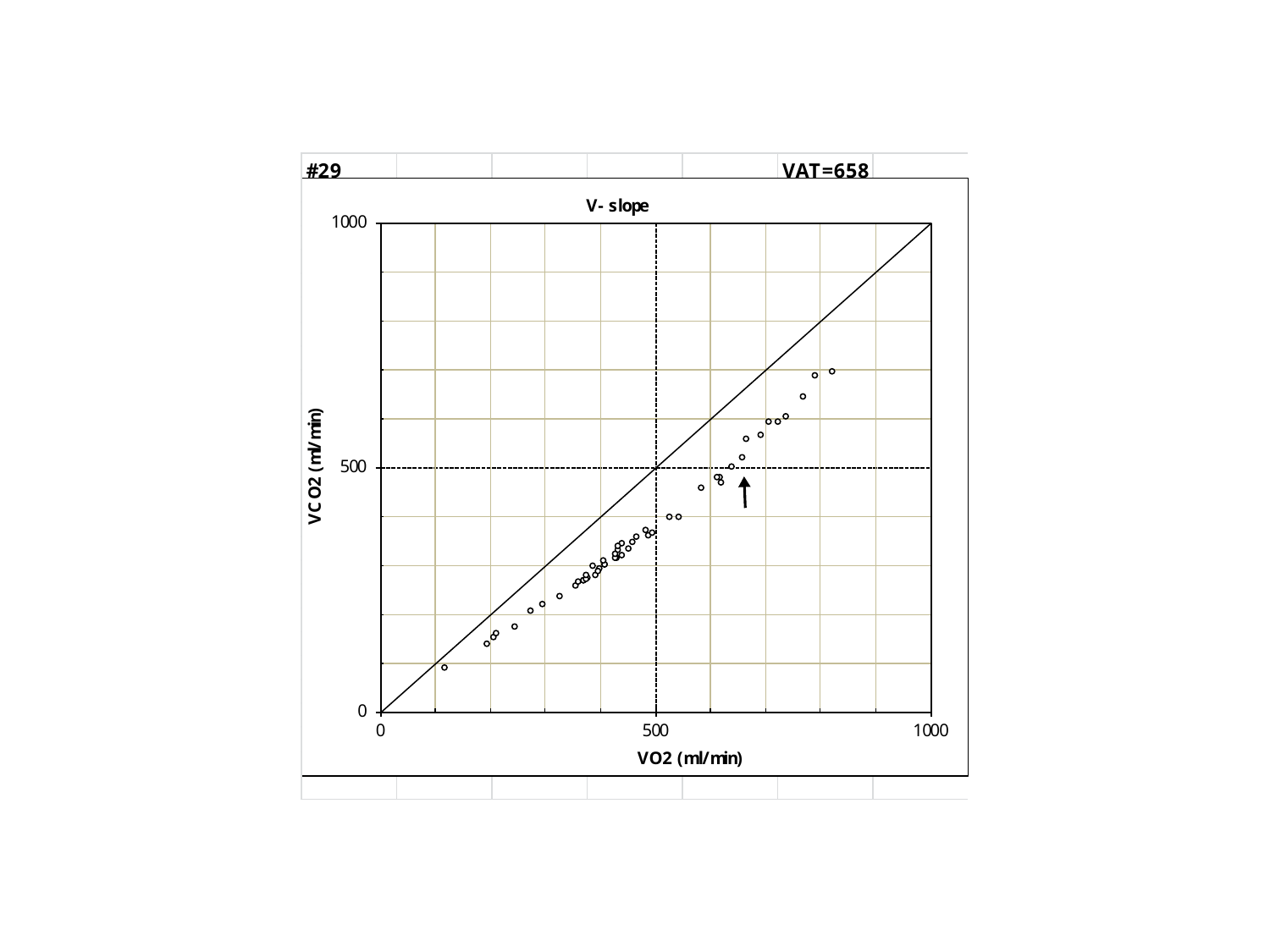

## Slide 32
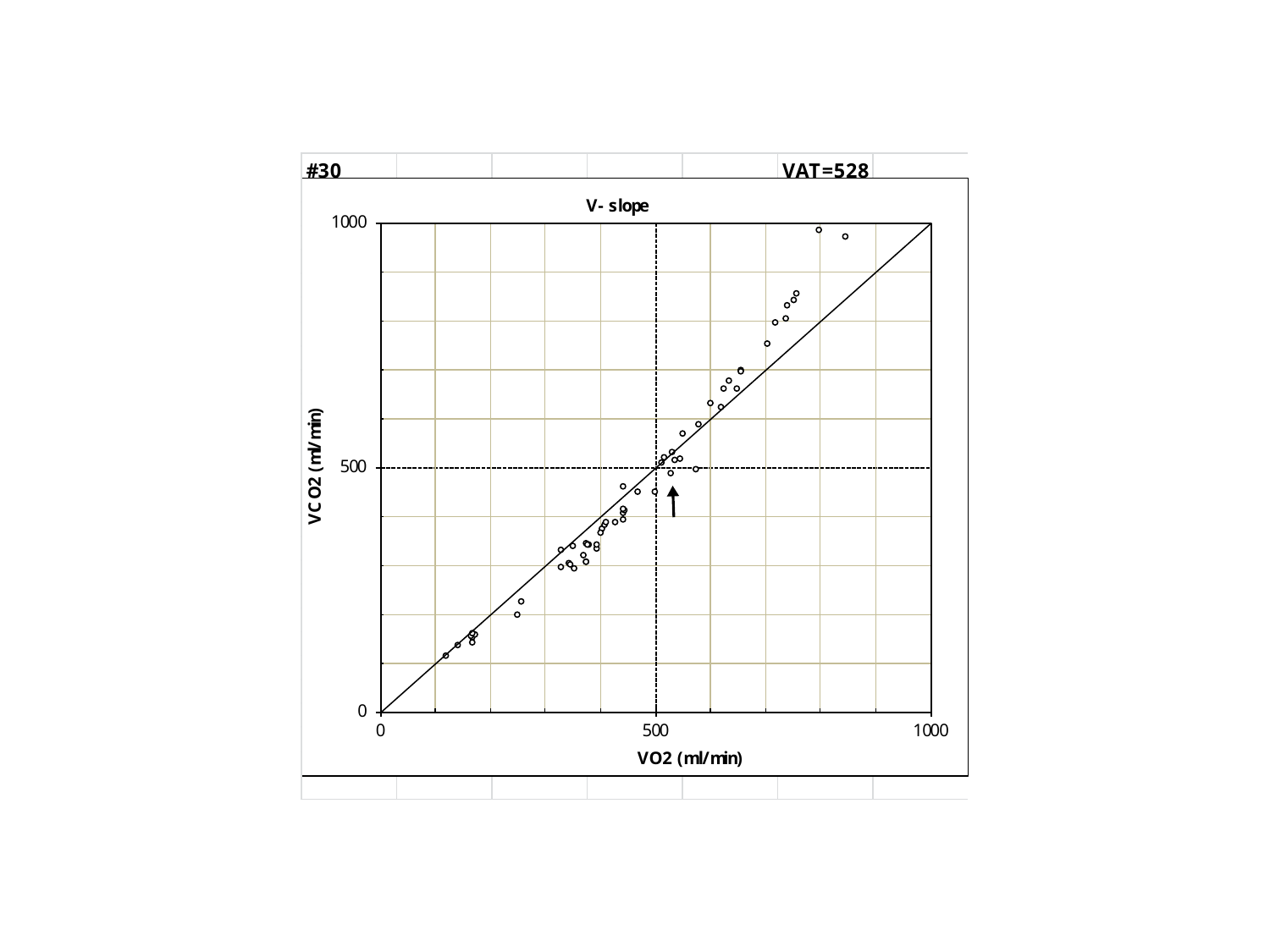

## Slide 33
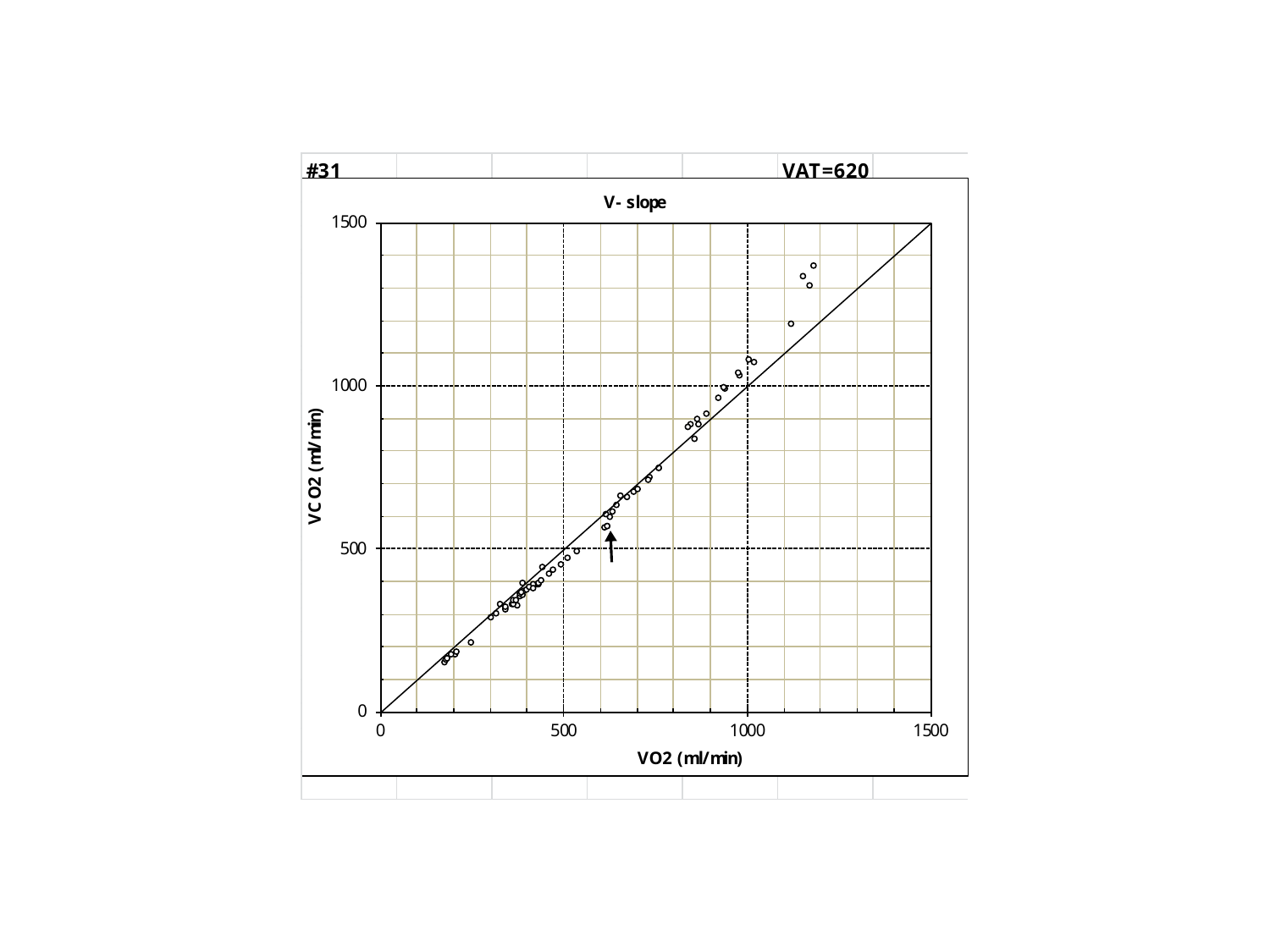

## Slide 34
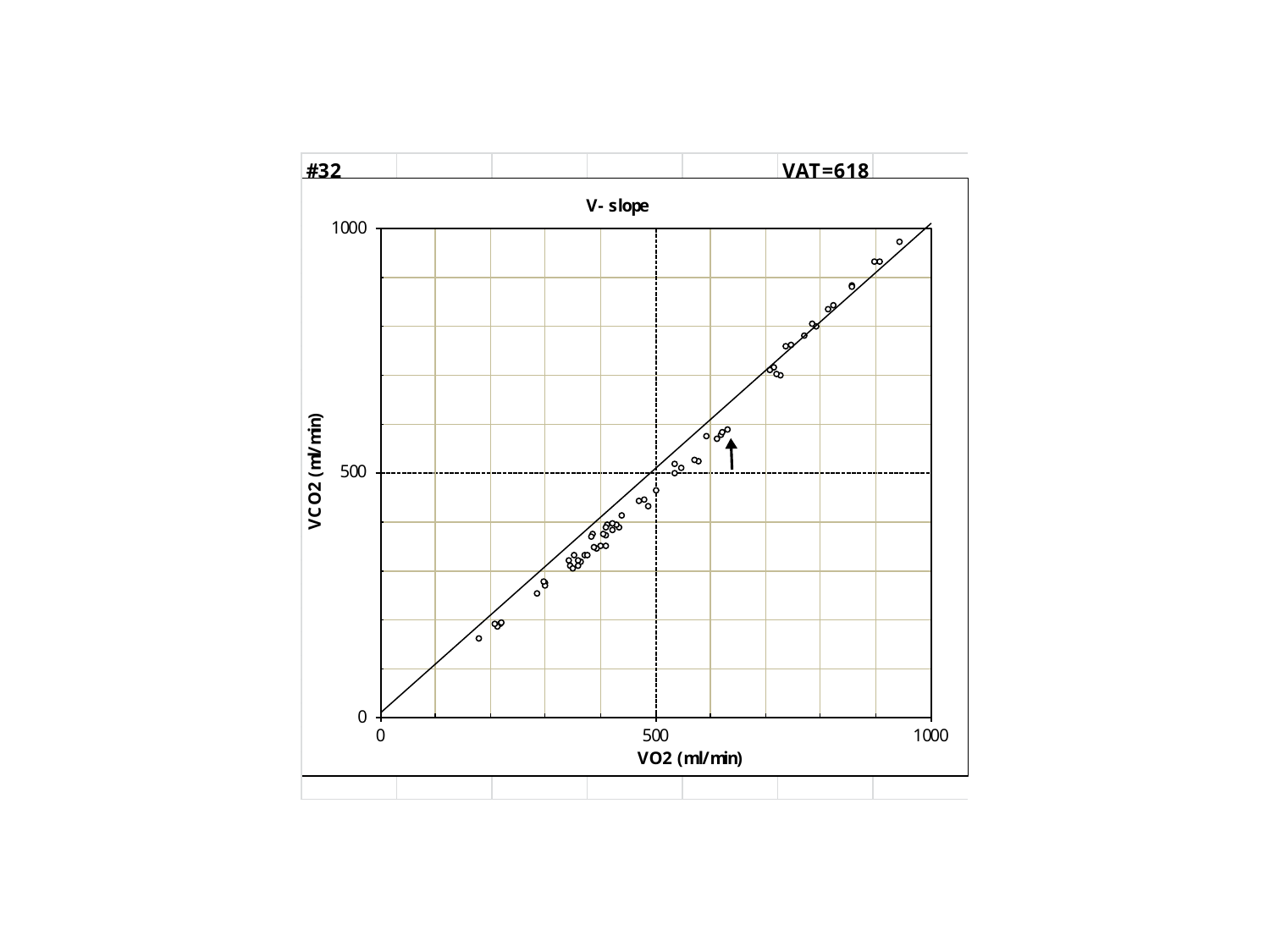

## Slide 35
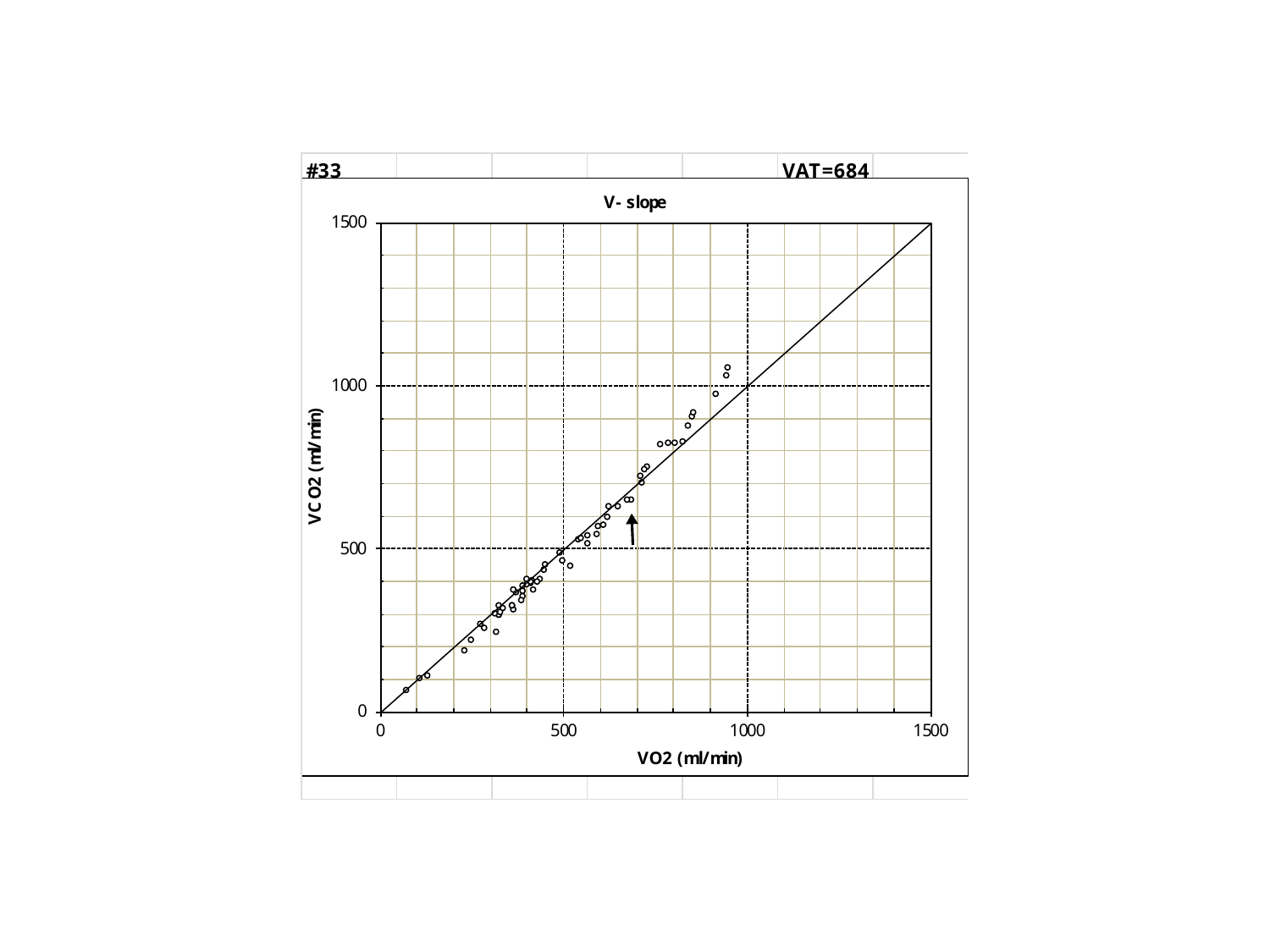

## Slide 36
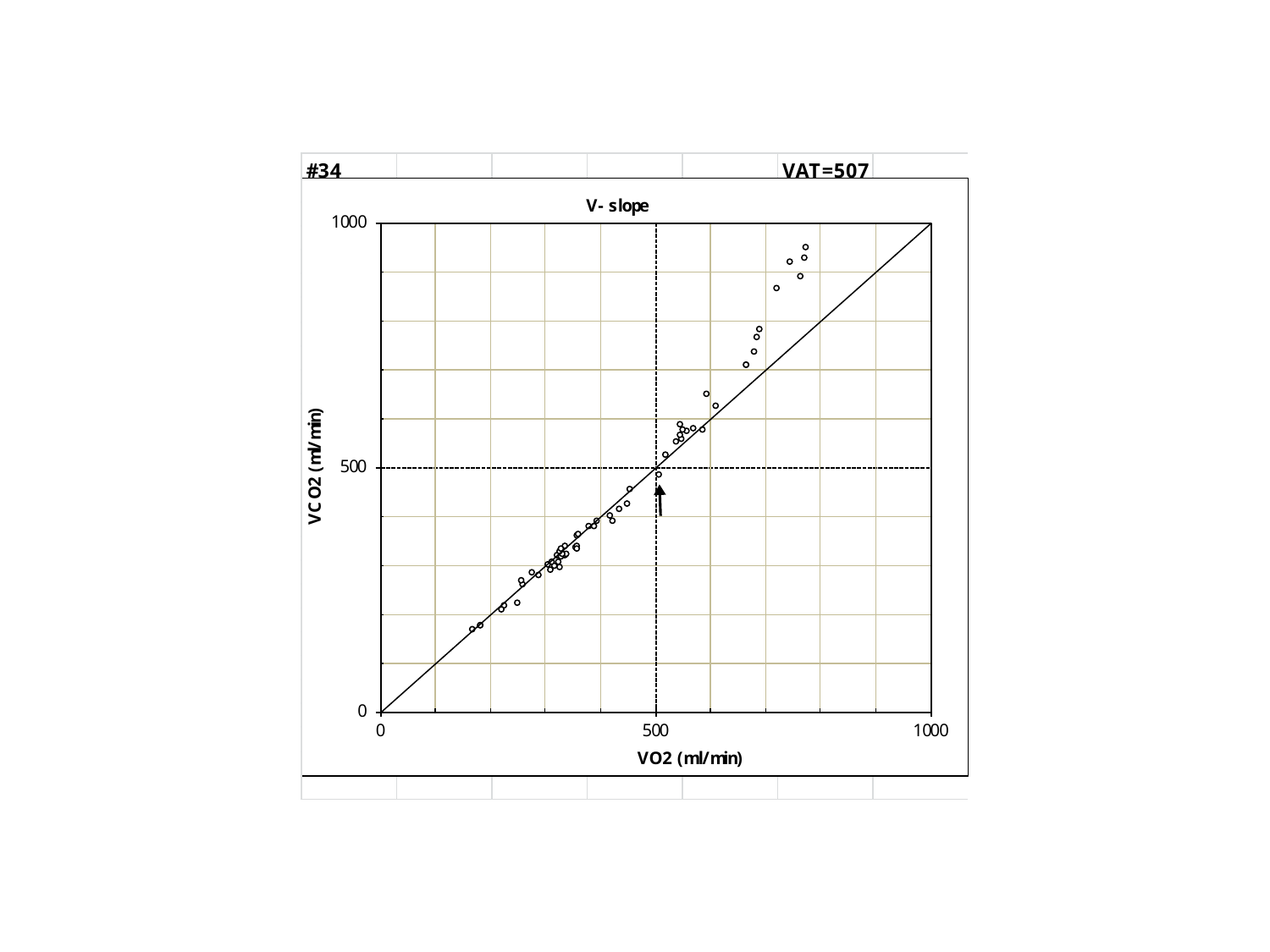

## Slide 37
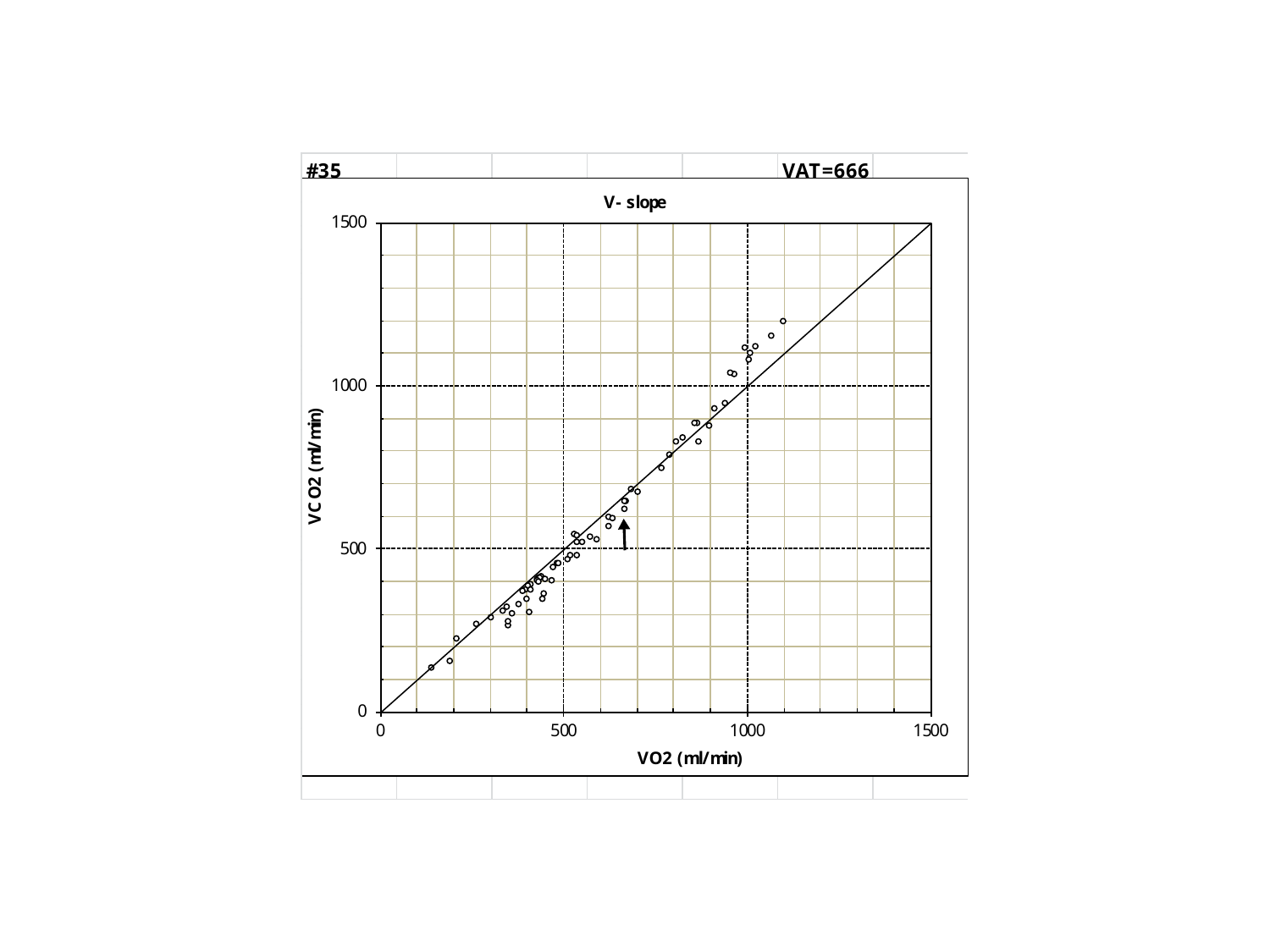

## Slide 38
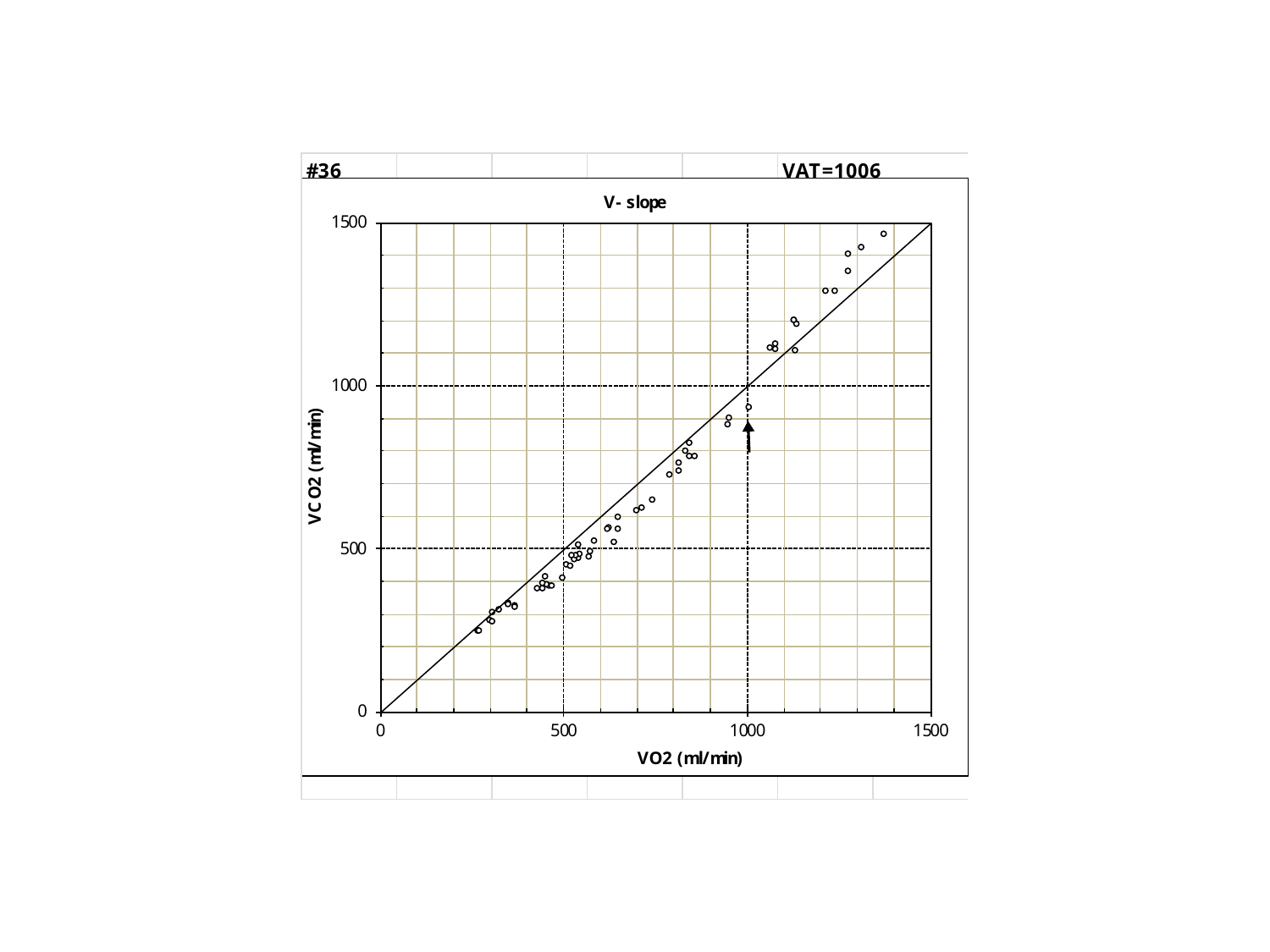

## Slide 39
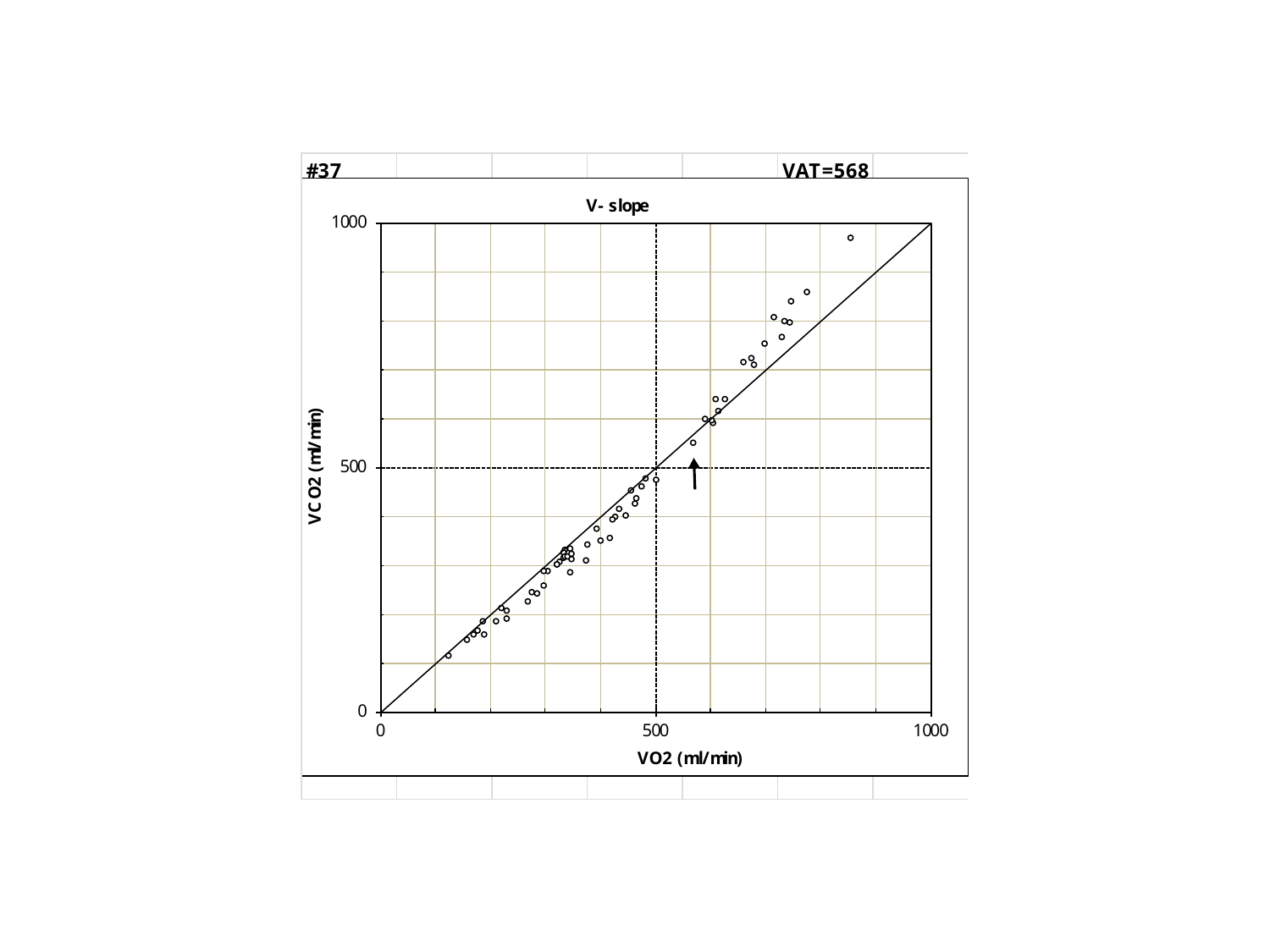

## Slide 40
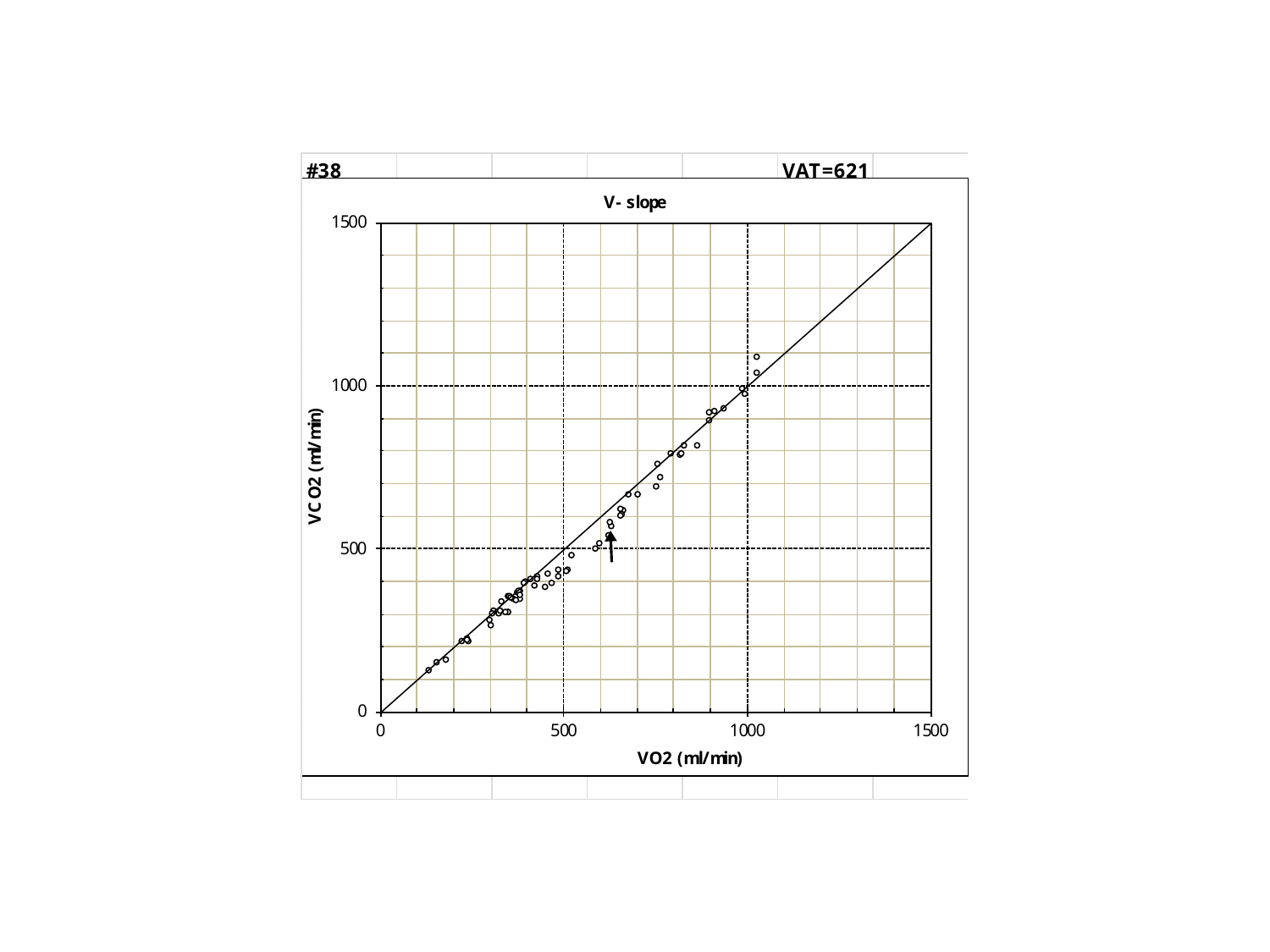

## Slide 41
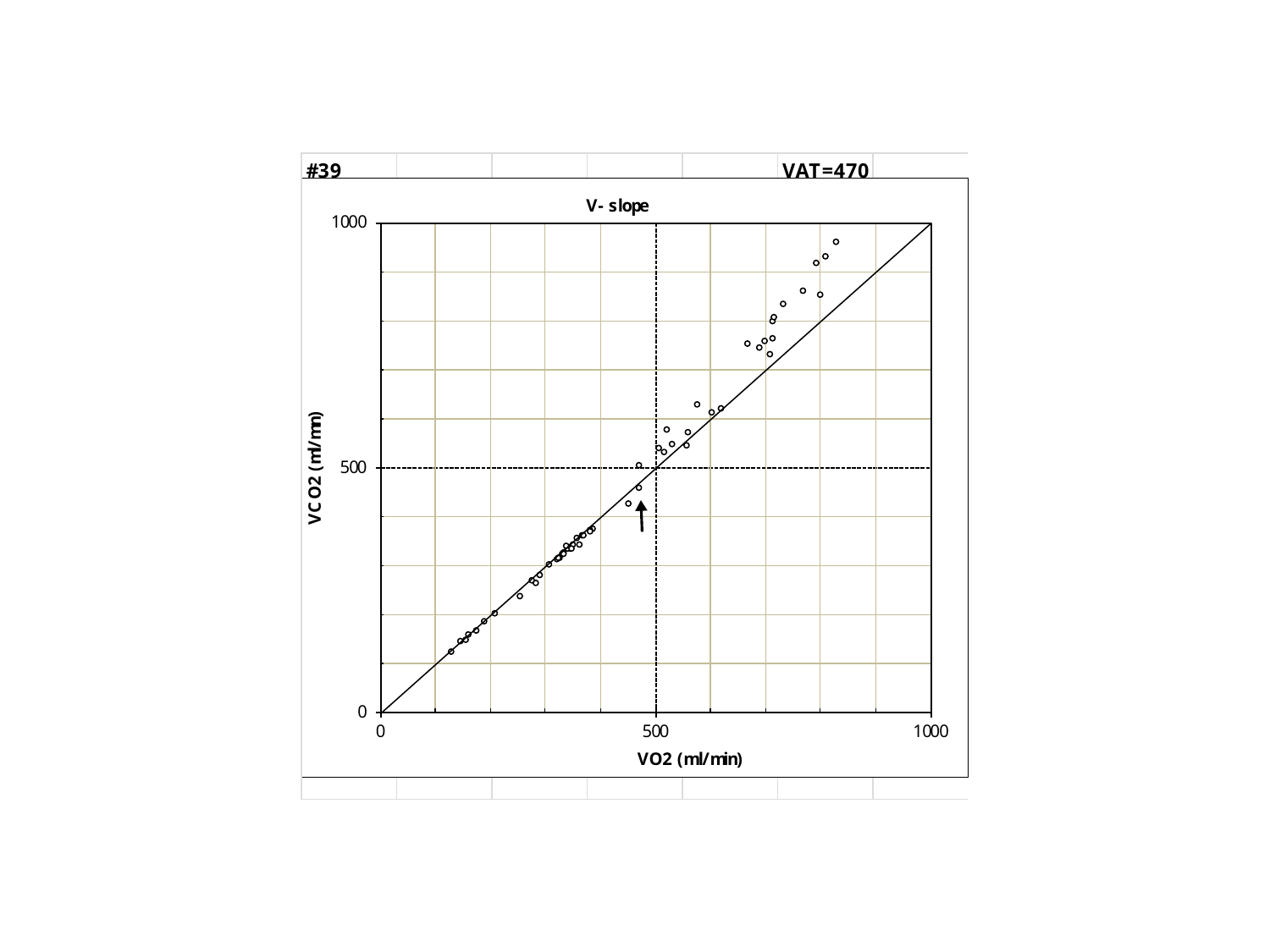

## Slide 42
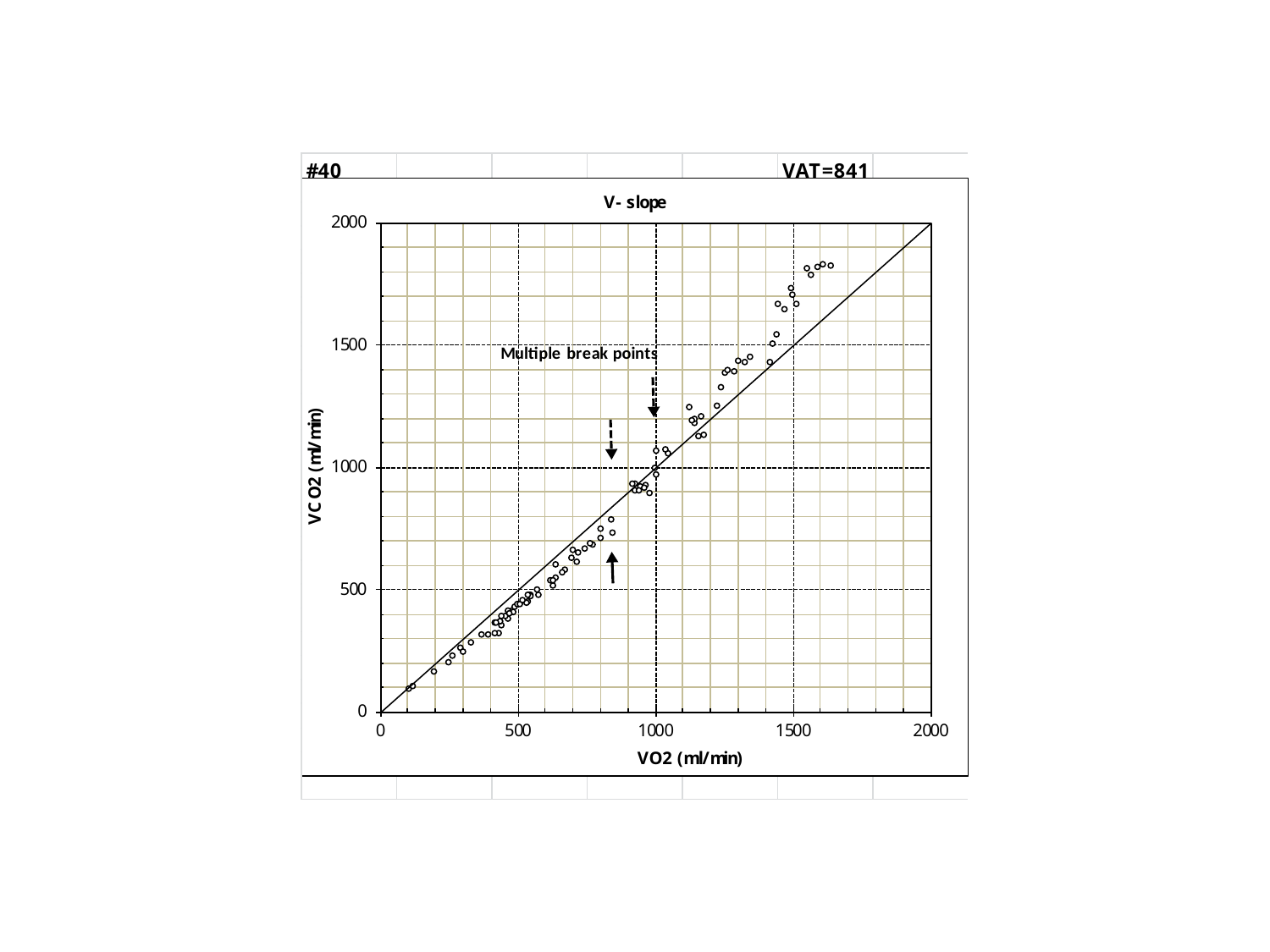

## Slide 43
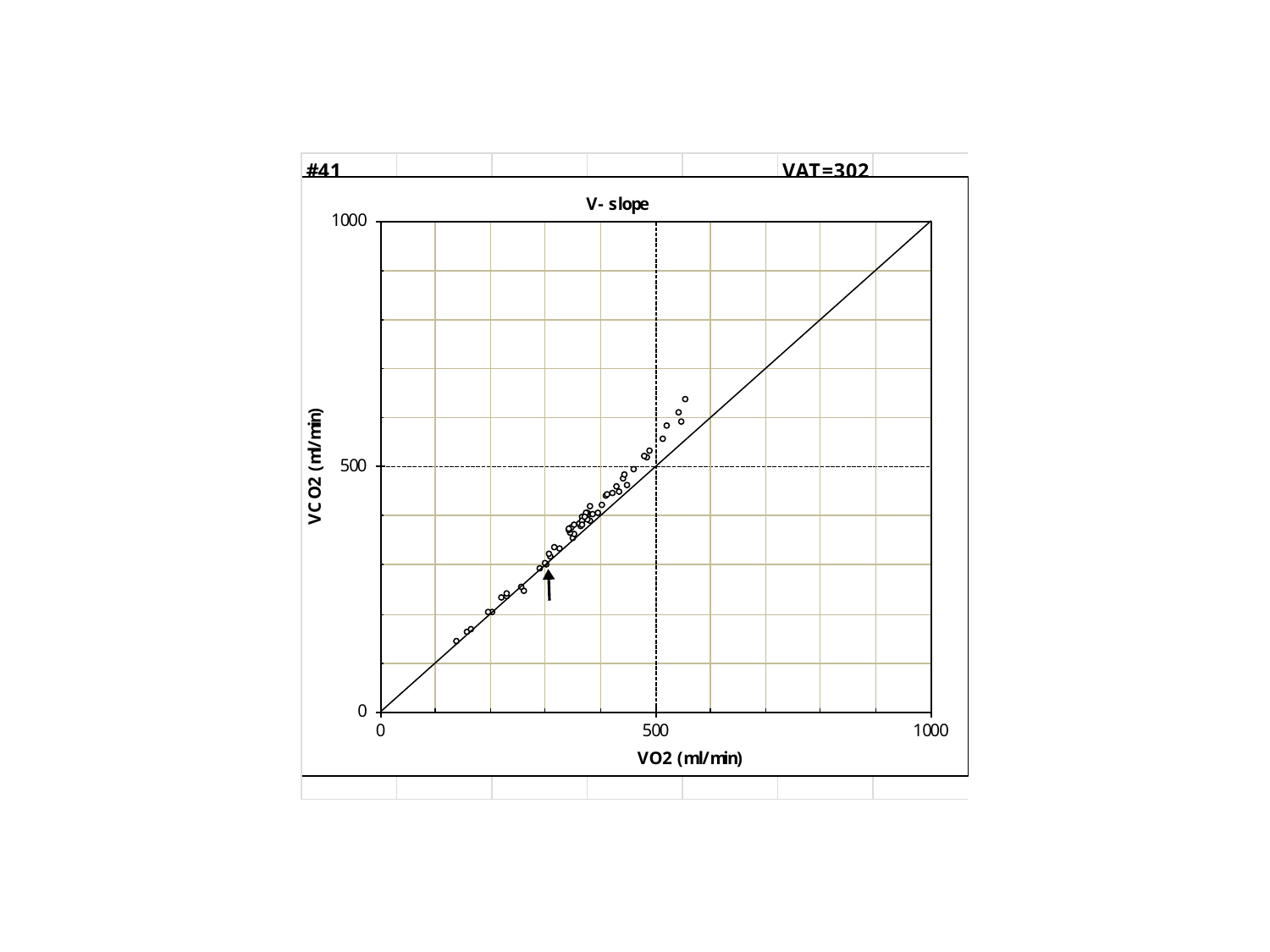

## Slide 44
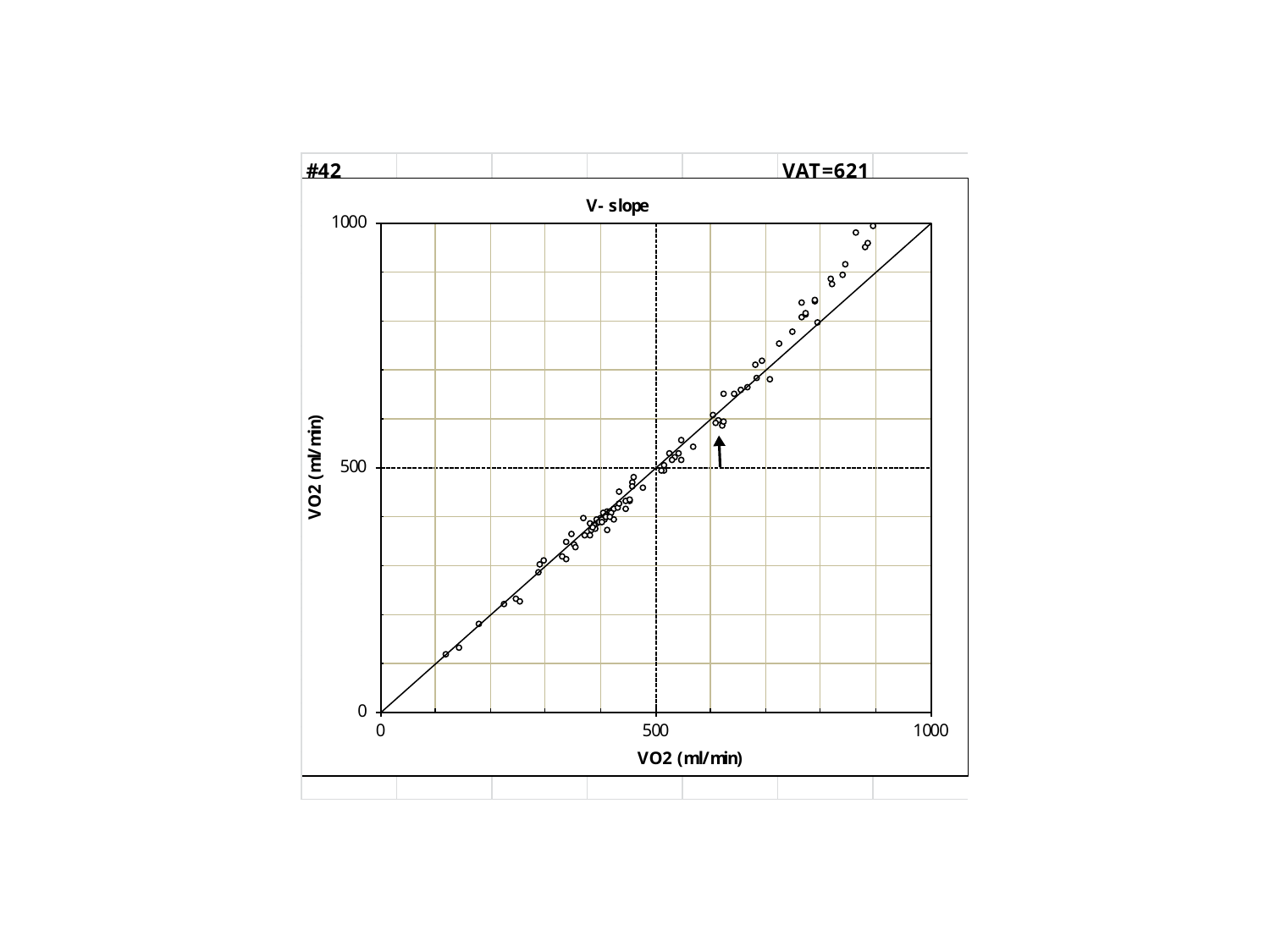

## Slide 45
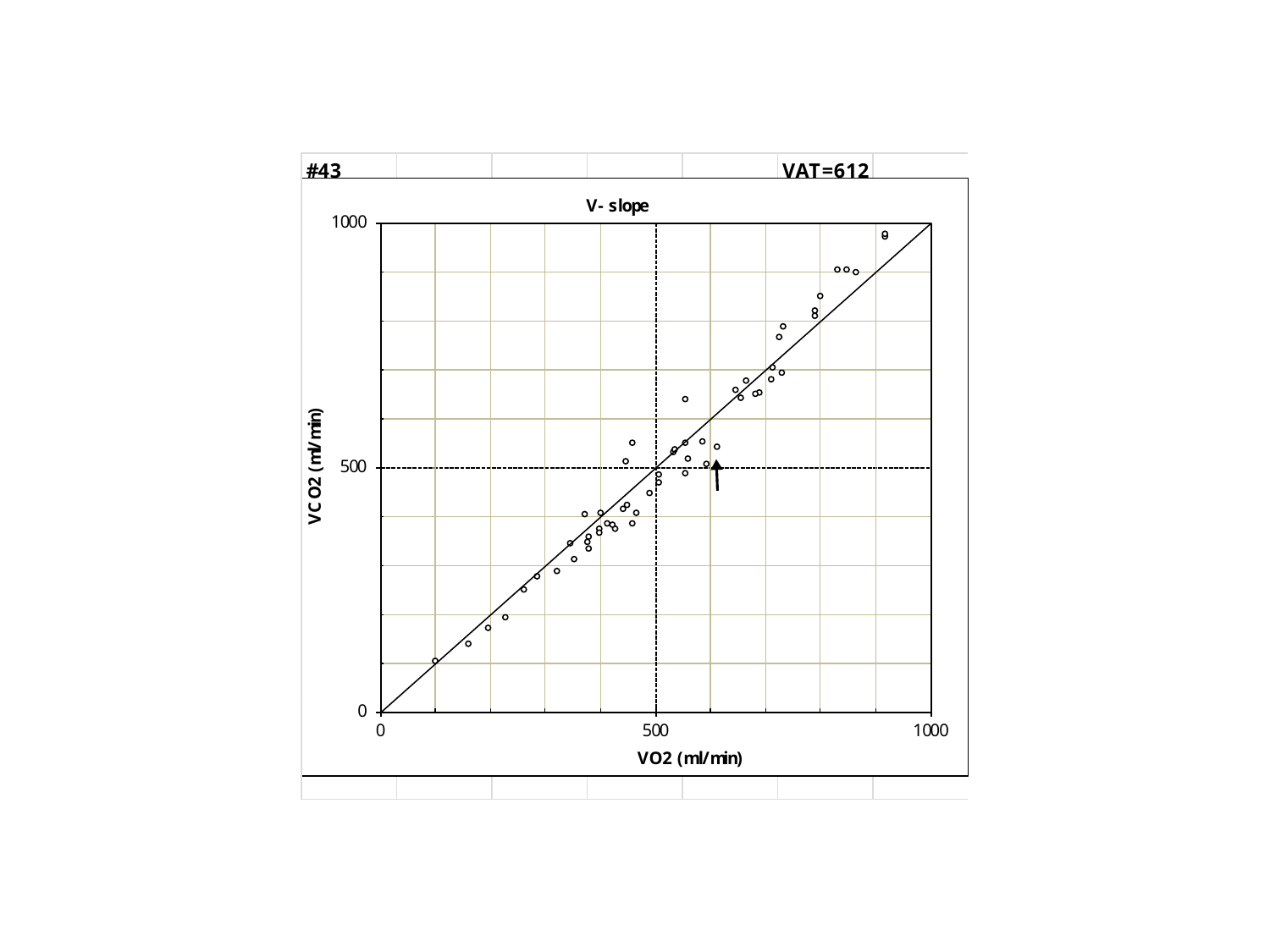

## Slide 46
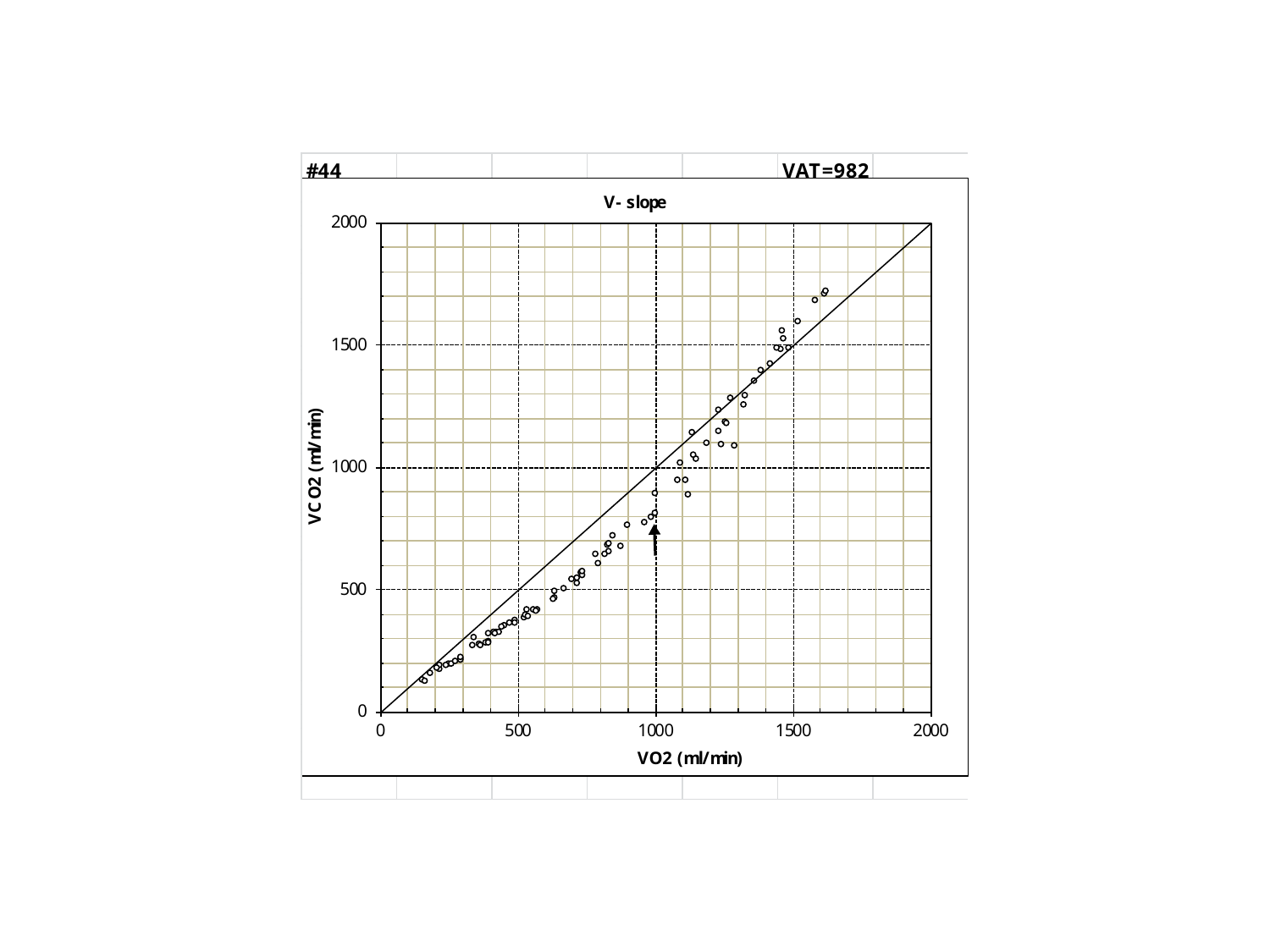

## Slide 47
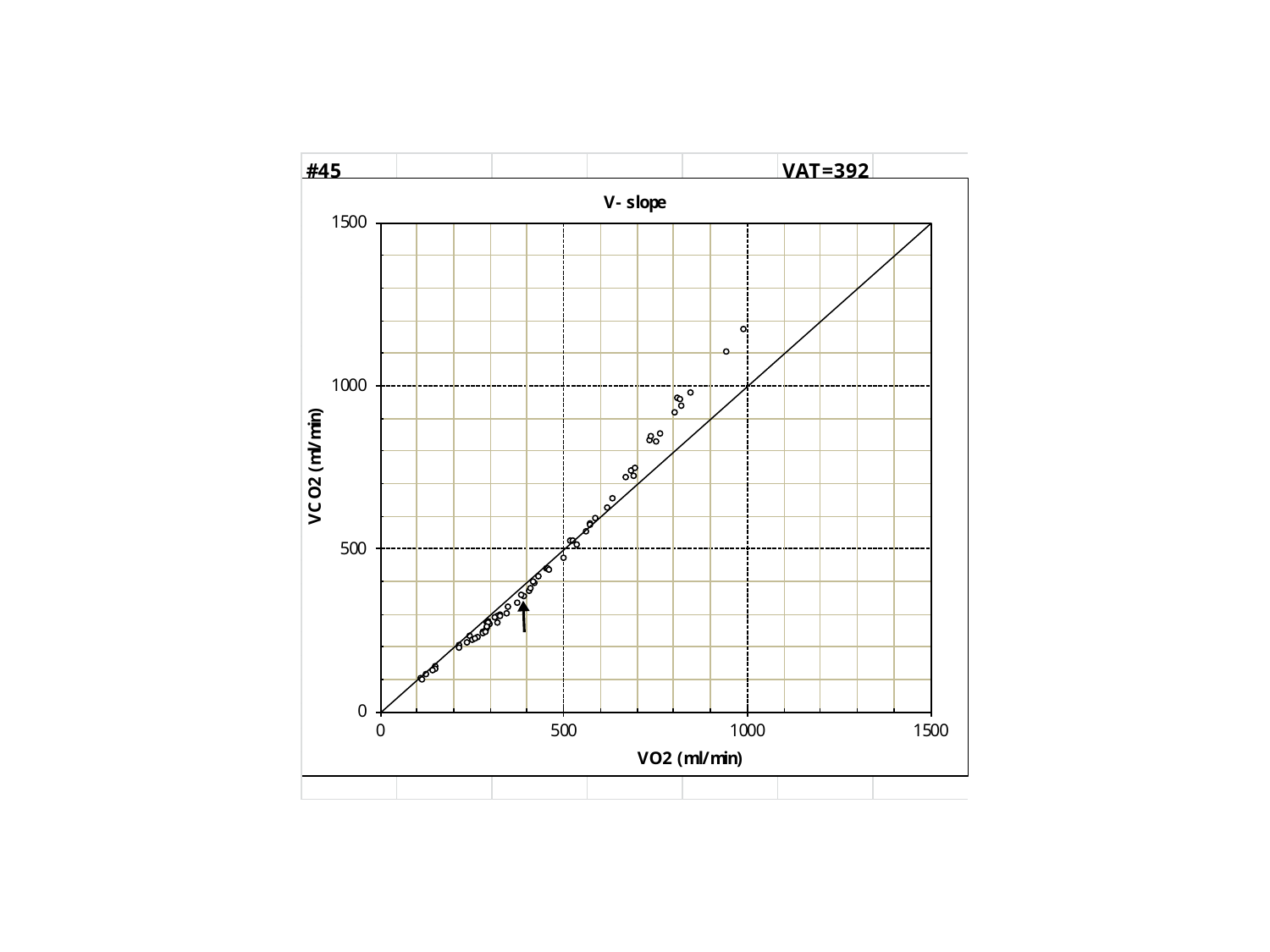

## Slide 48
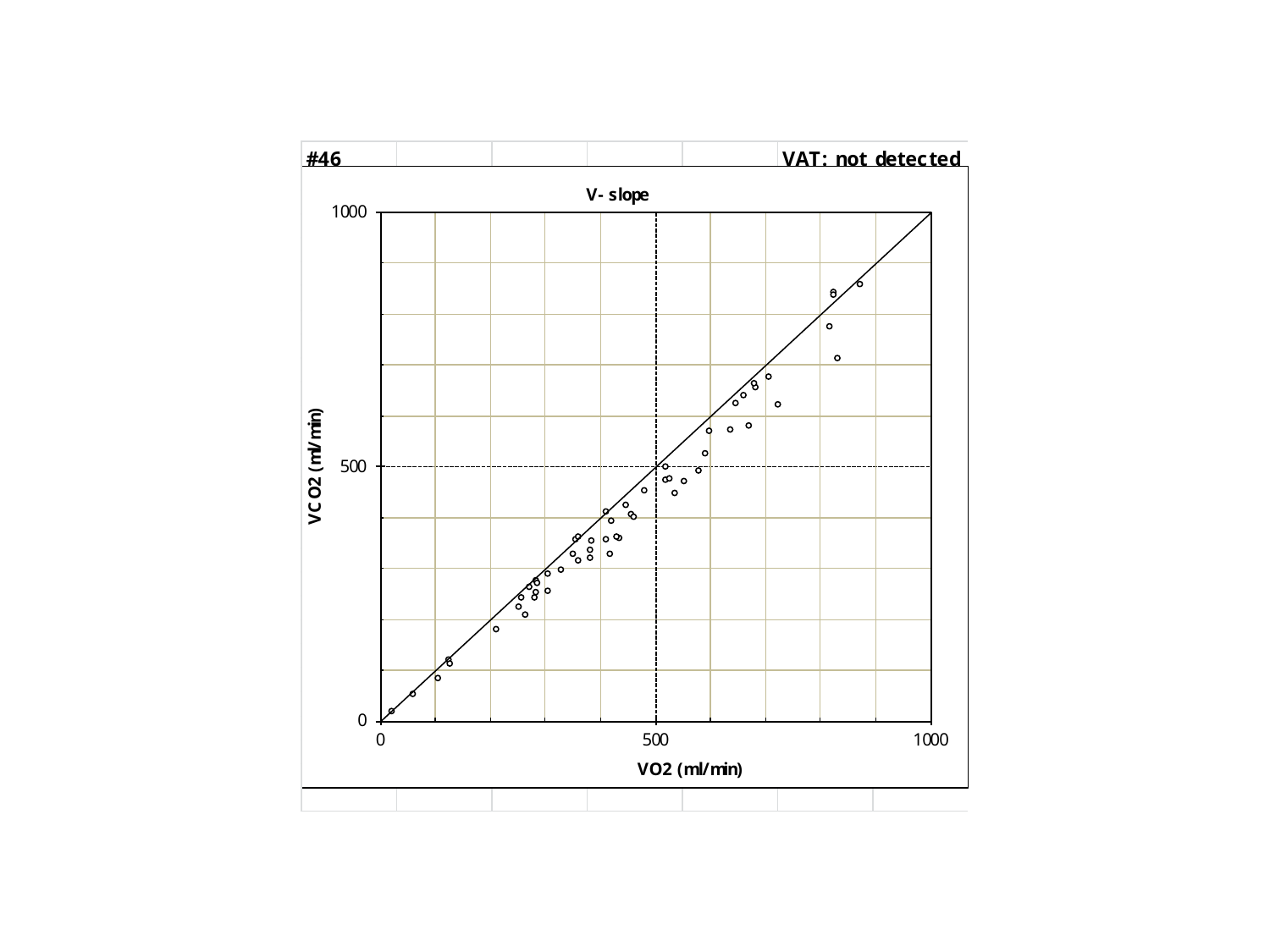

## Slide 49
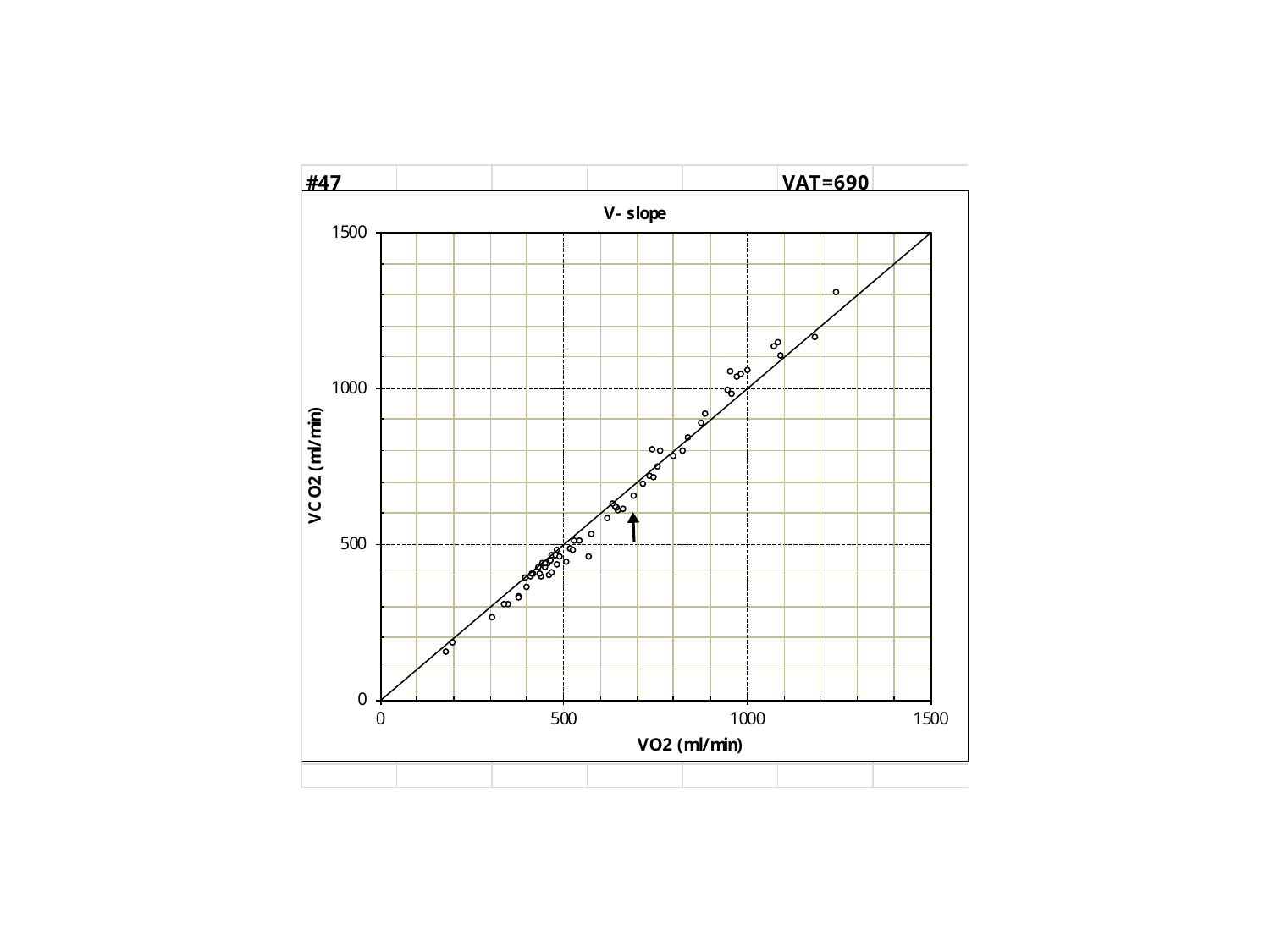

## Slide 50
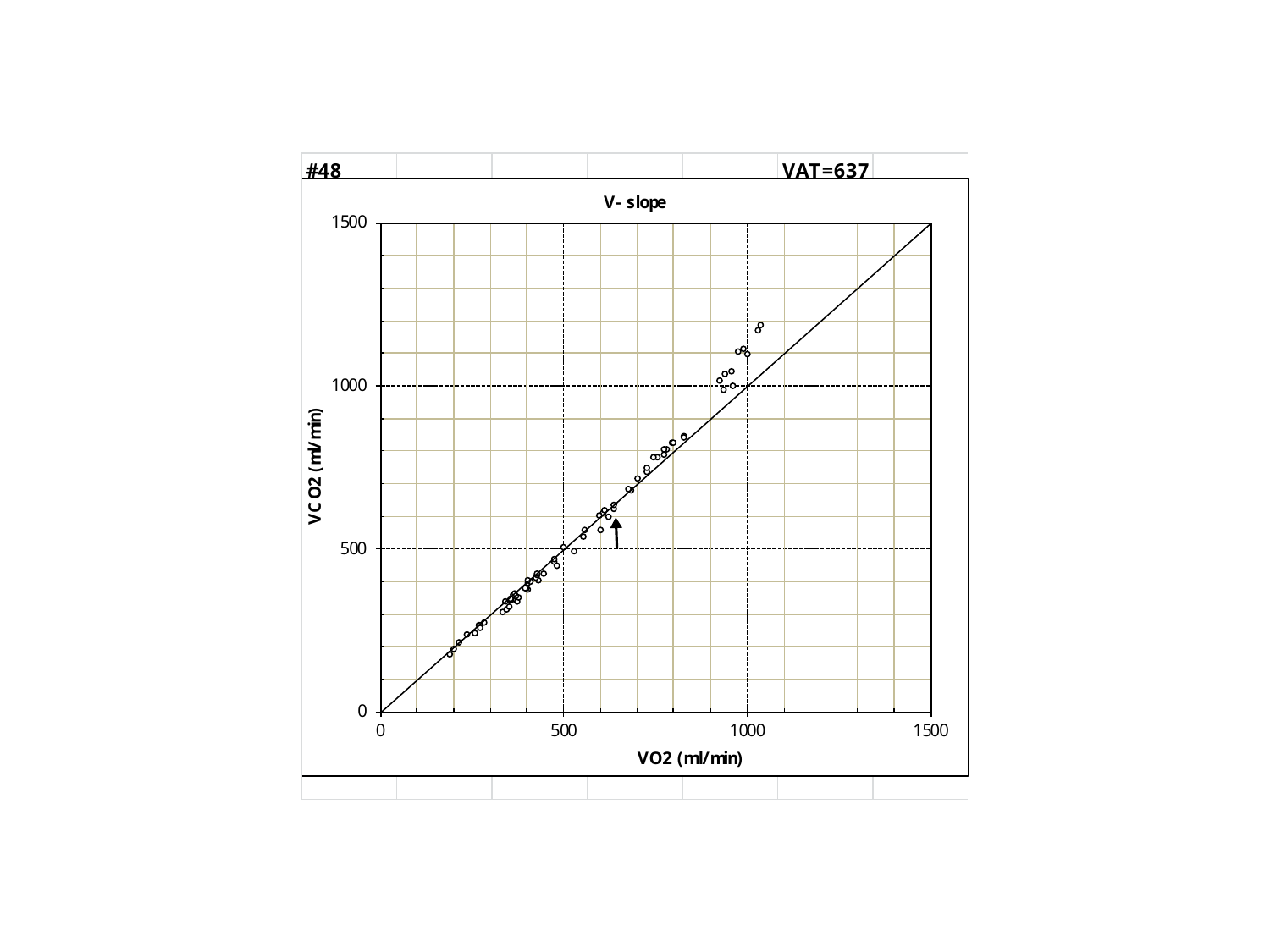

## Slide 51
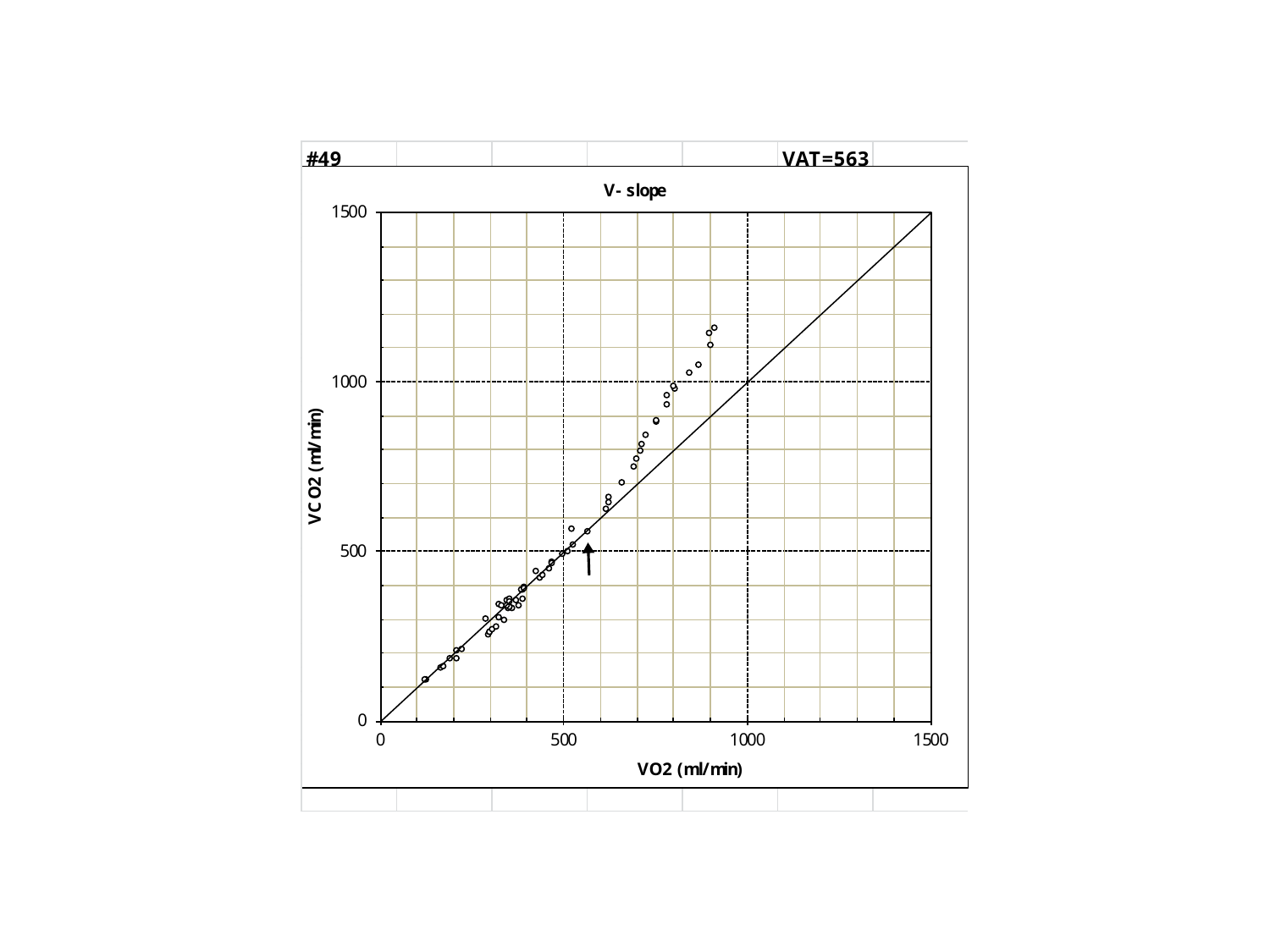

## Slide 52
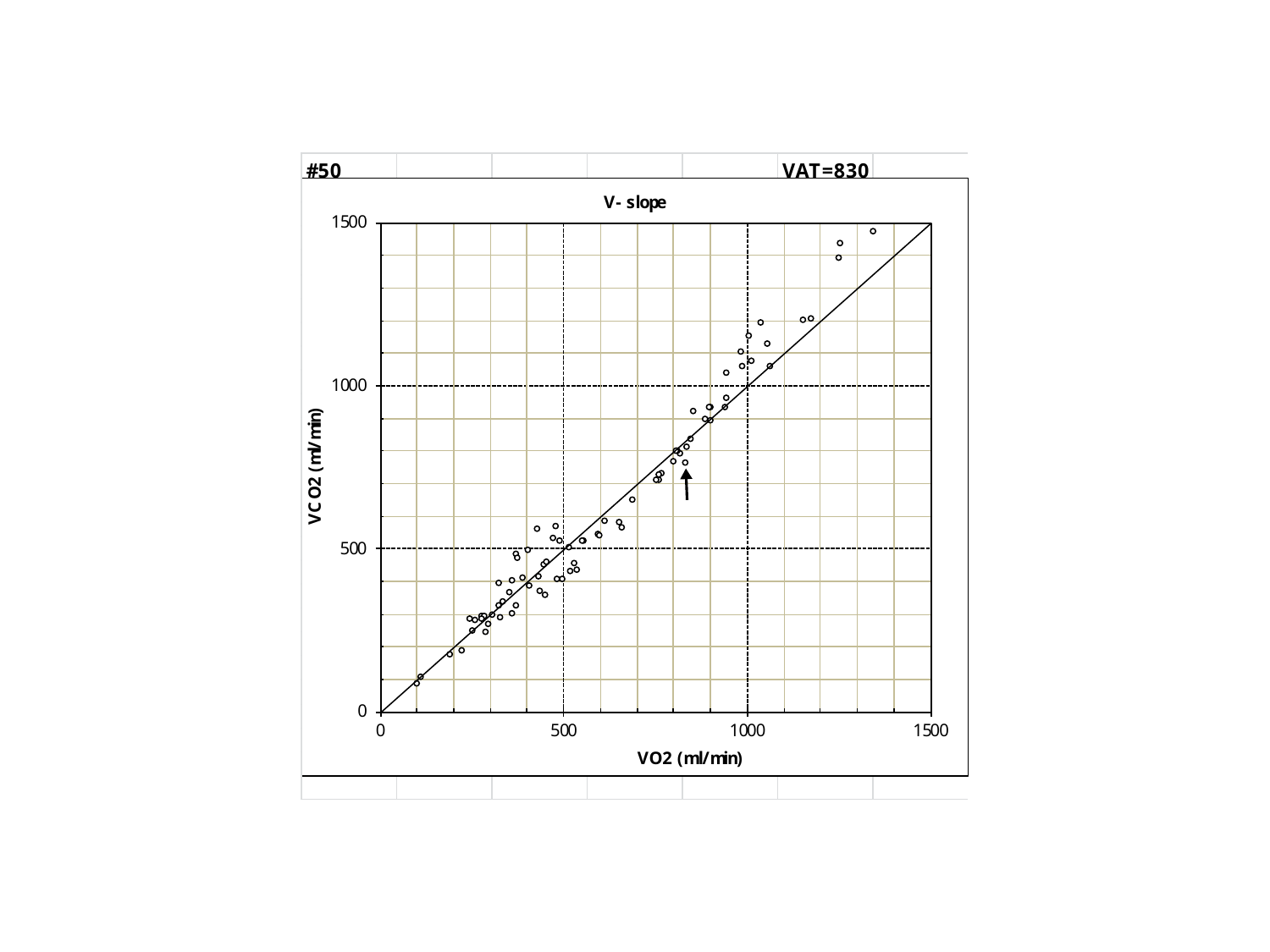

## Slide 53
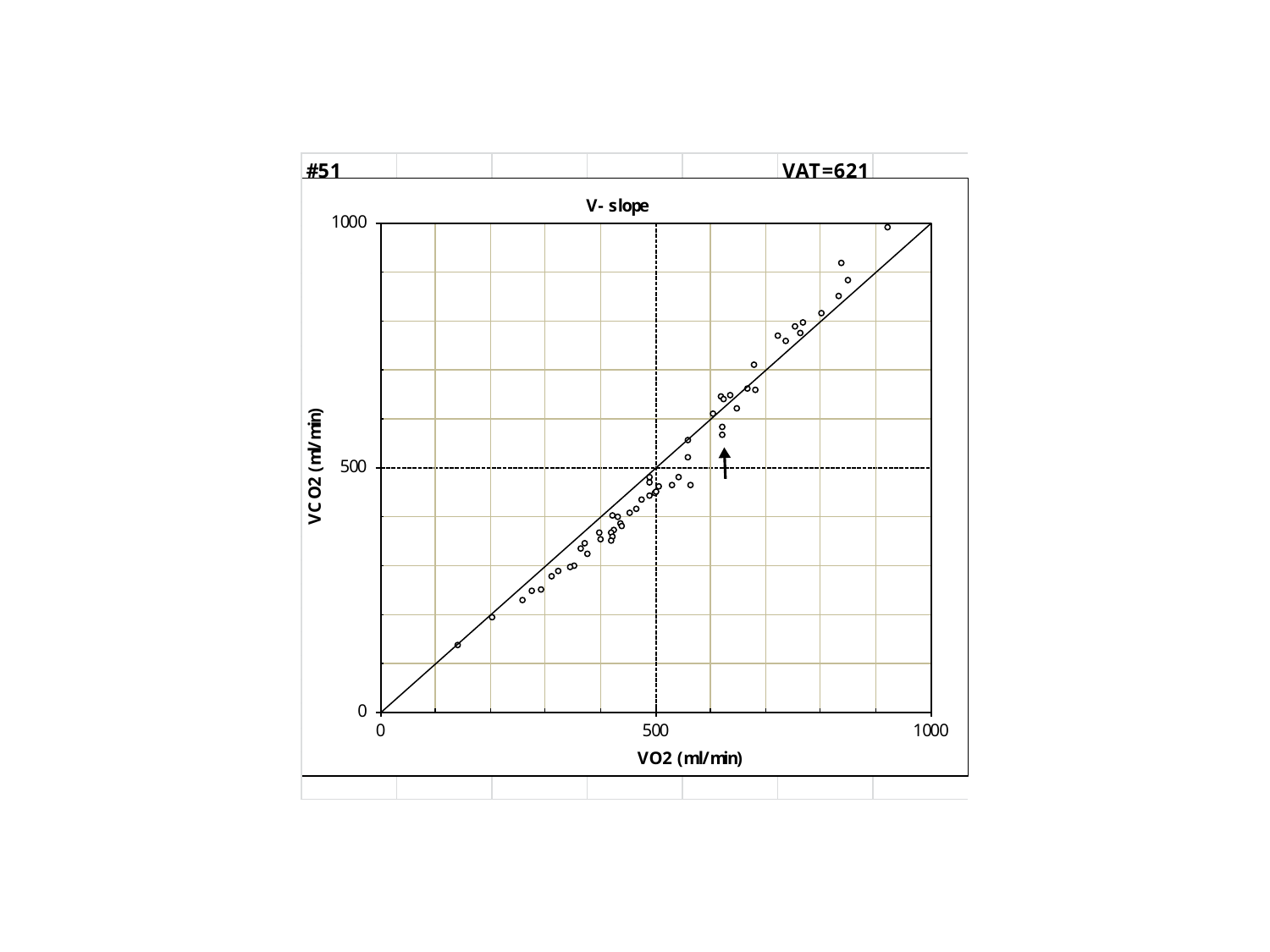

## Slide 54
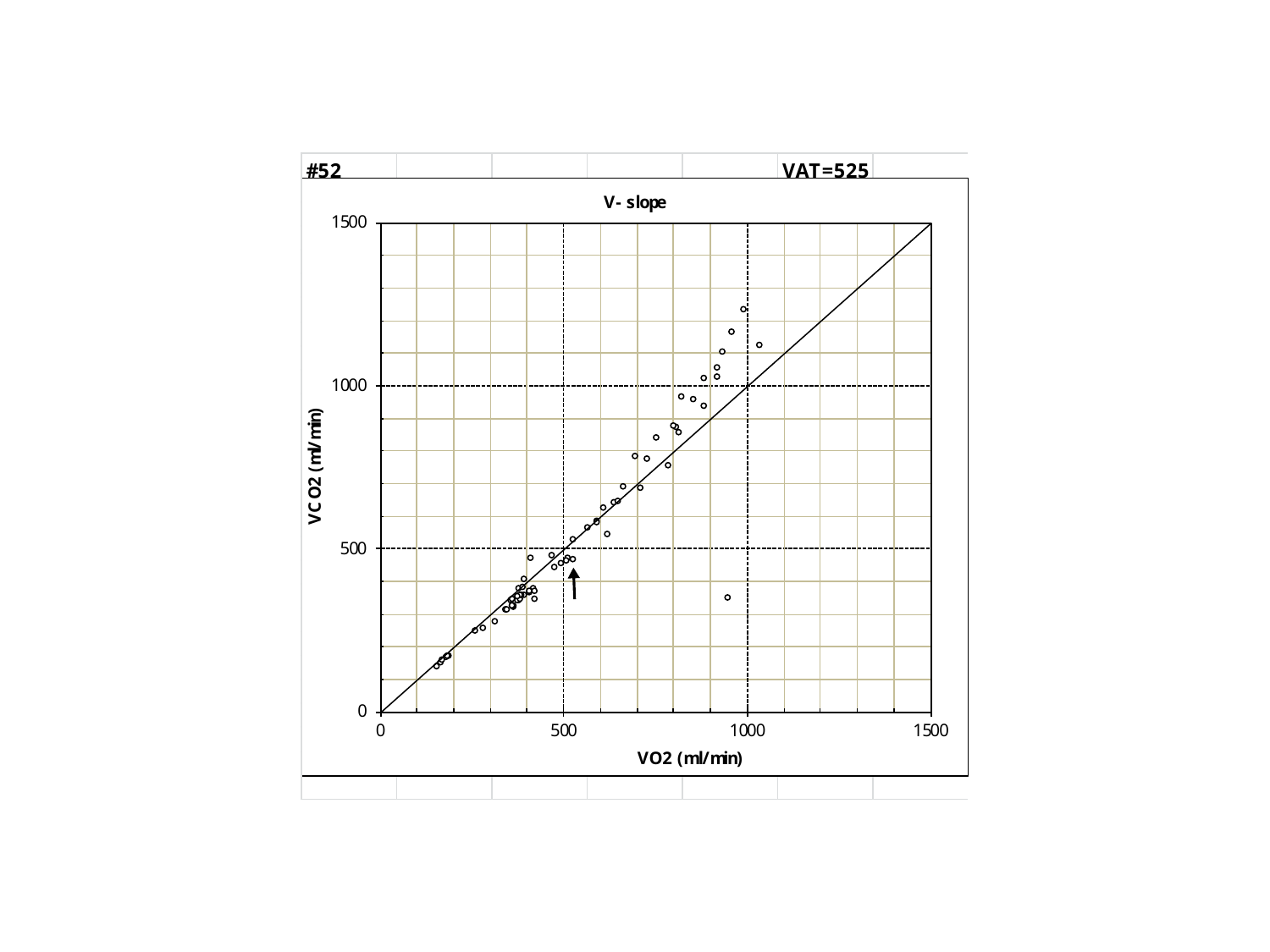

## Slide 55
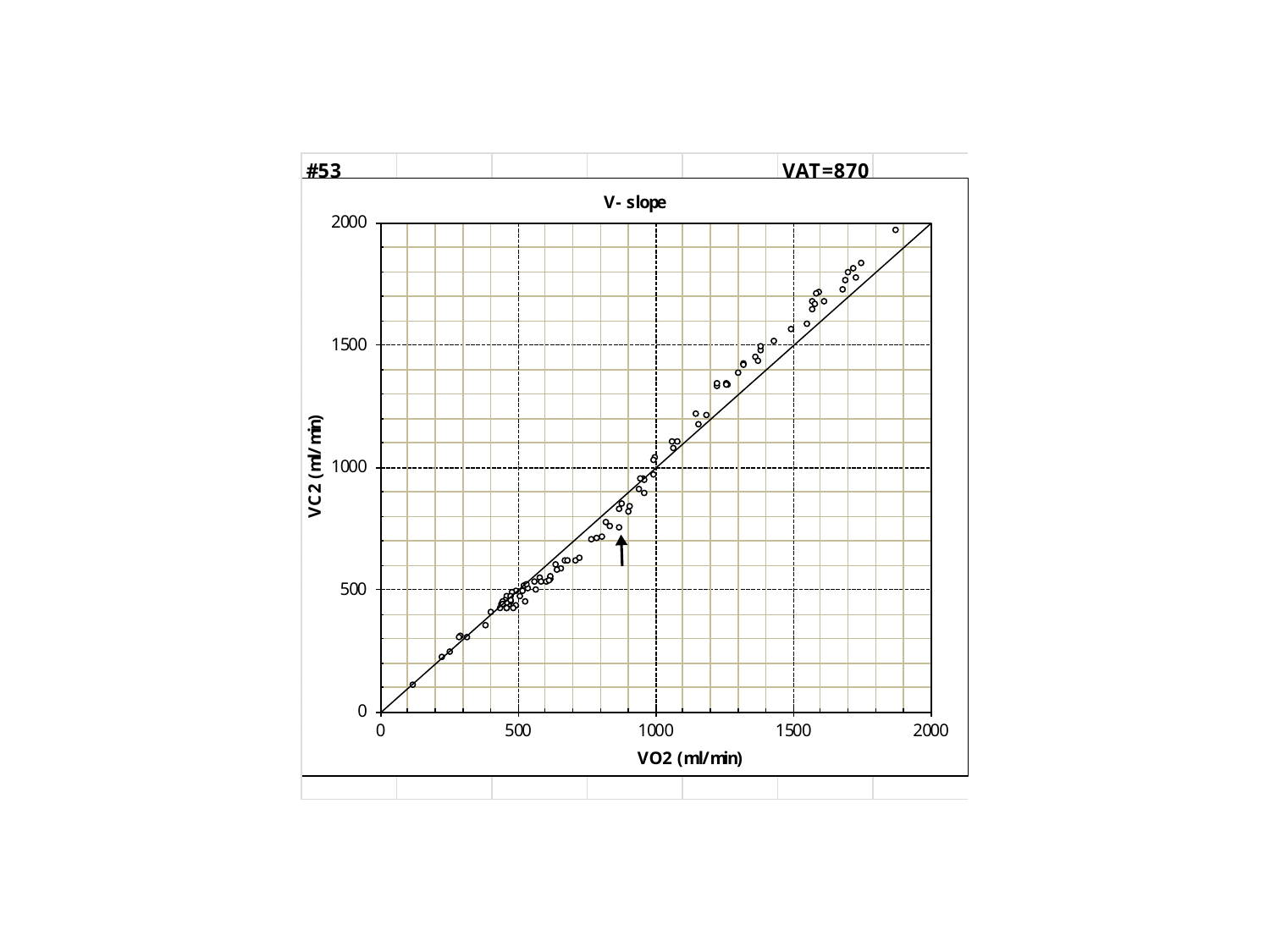

## Slide 56
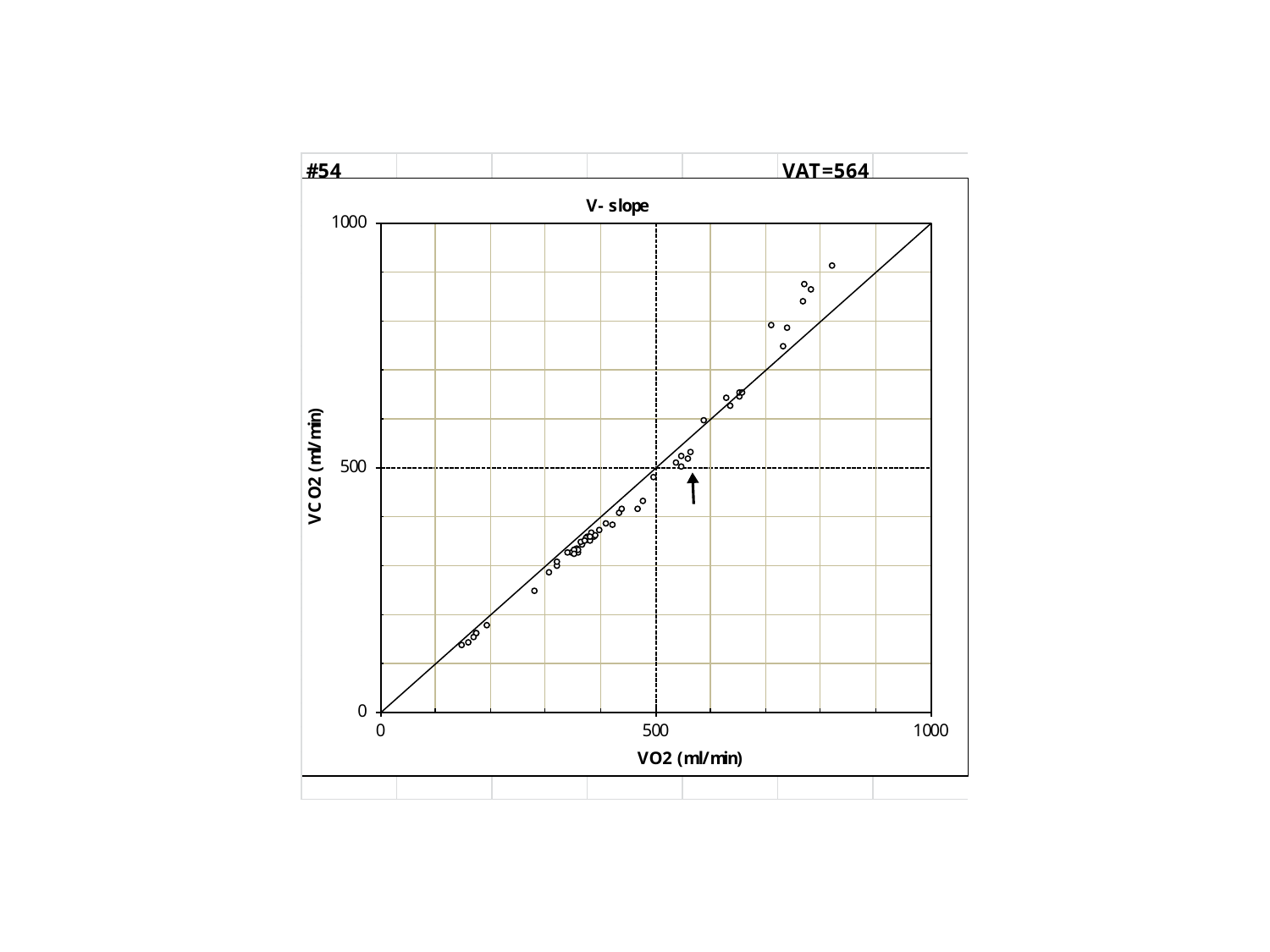

## Slide 57
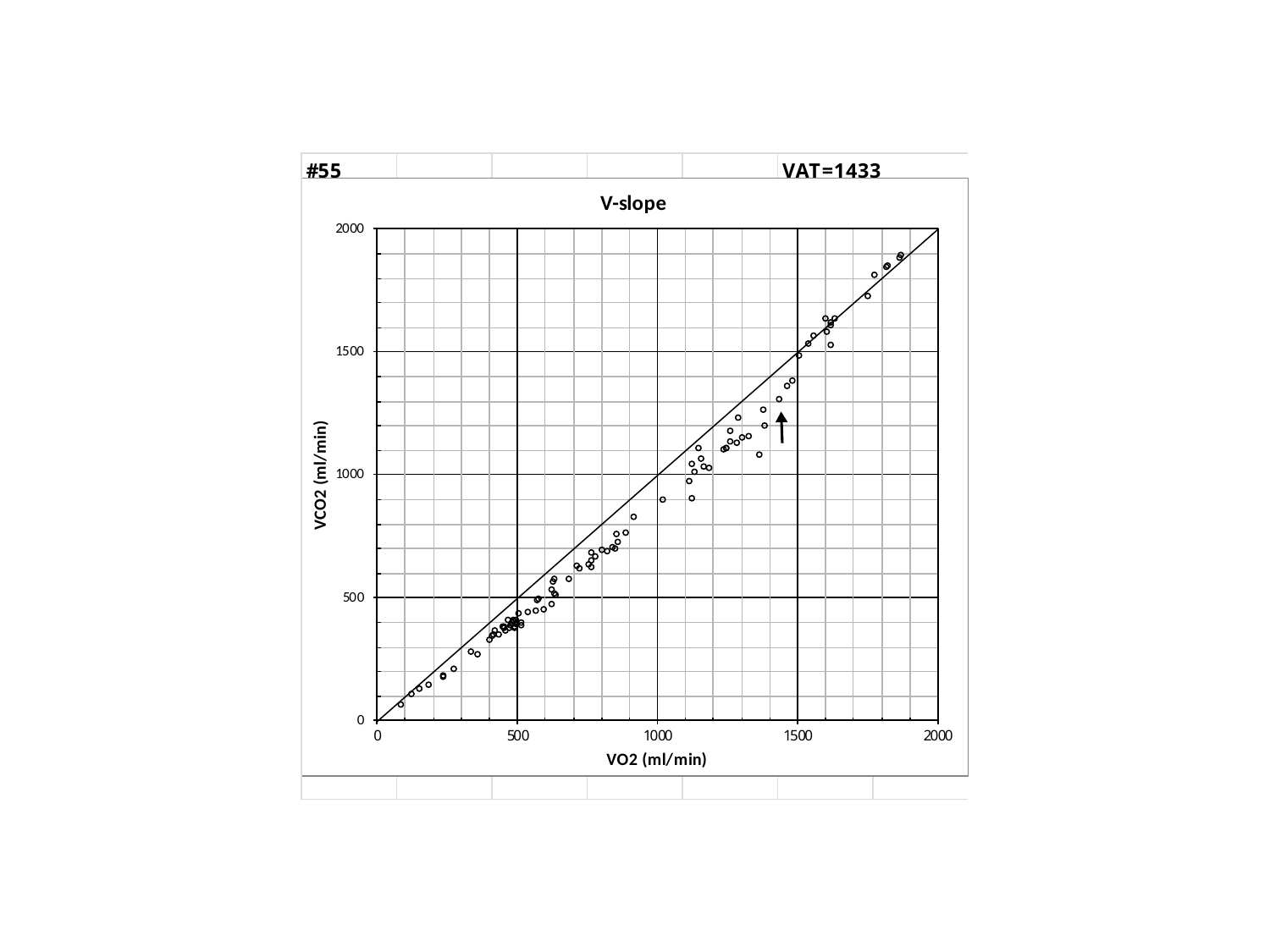

## Slide 58
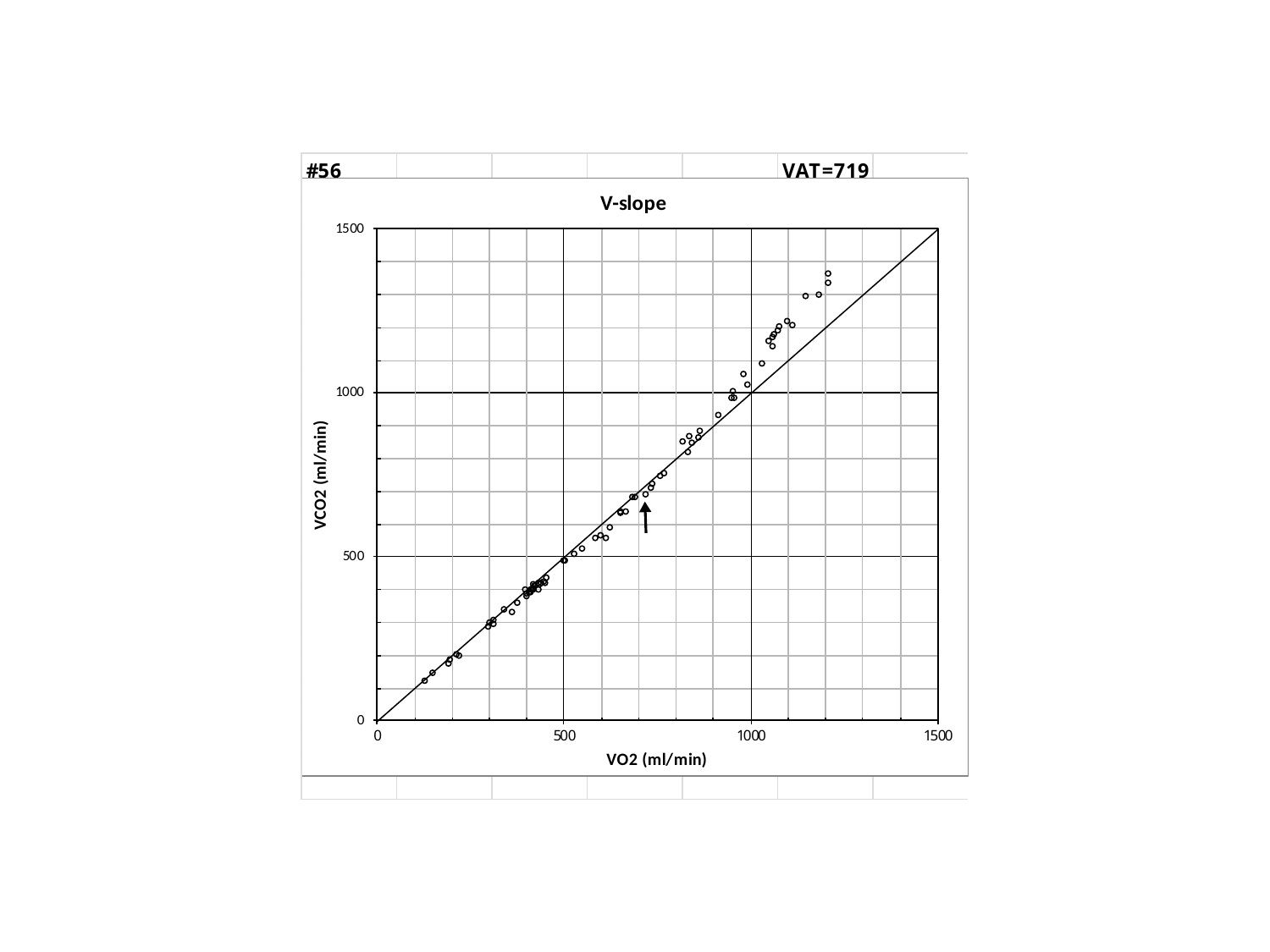

## Slide 59
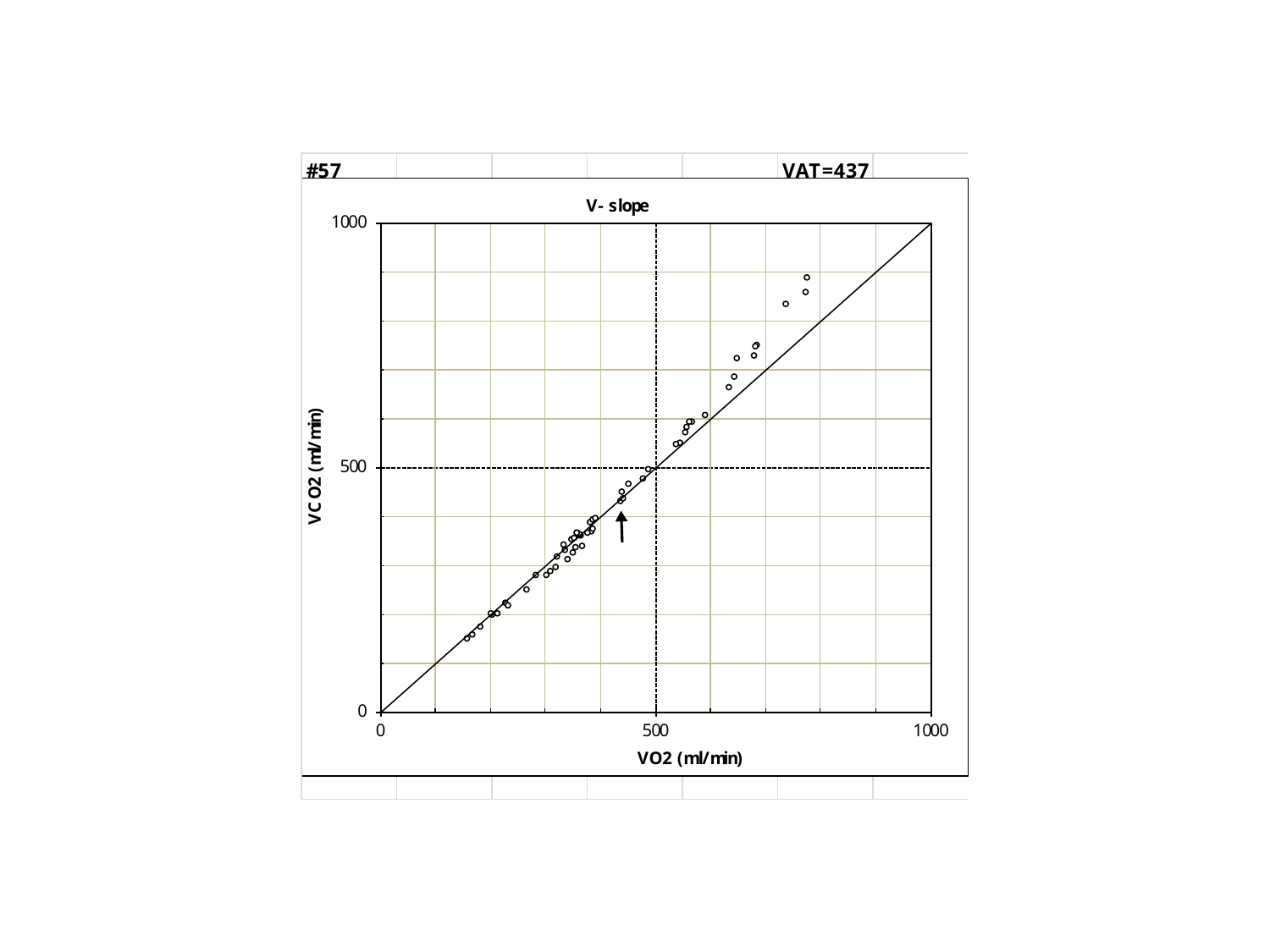

## Slide 60
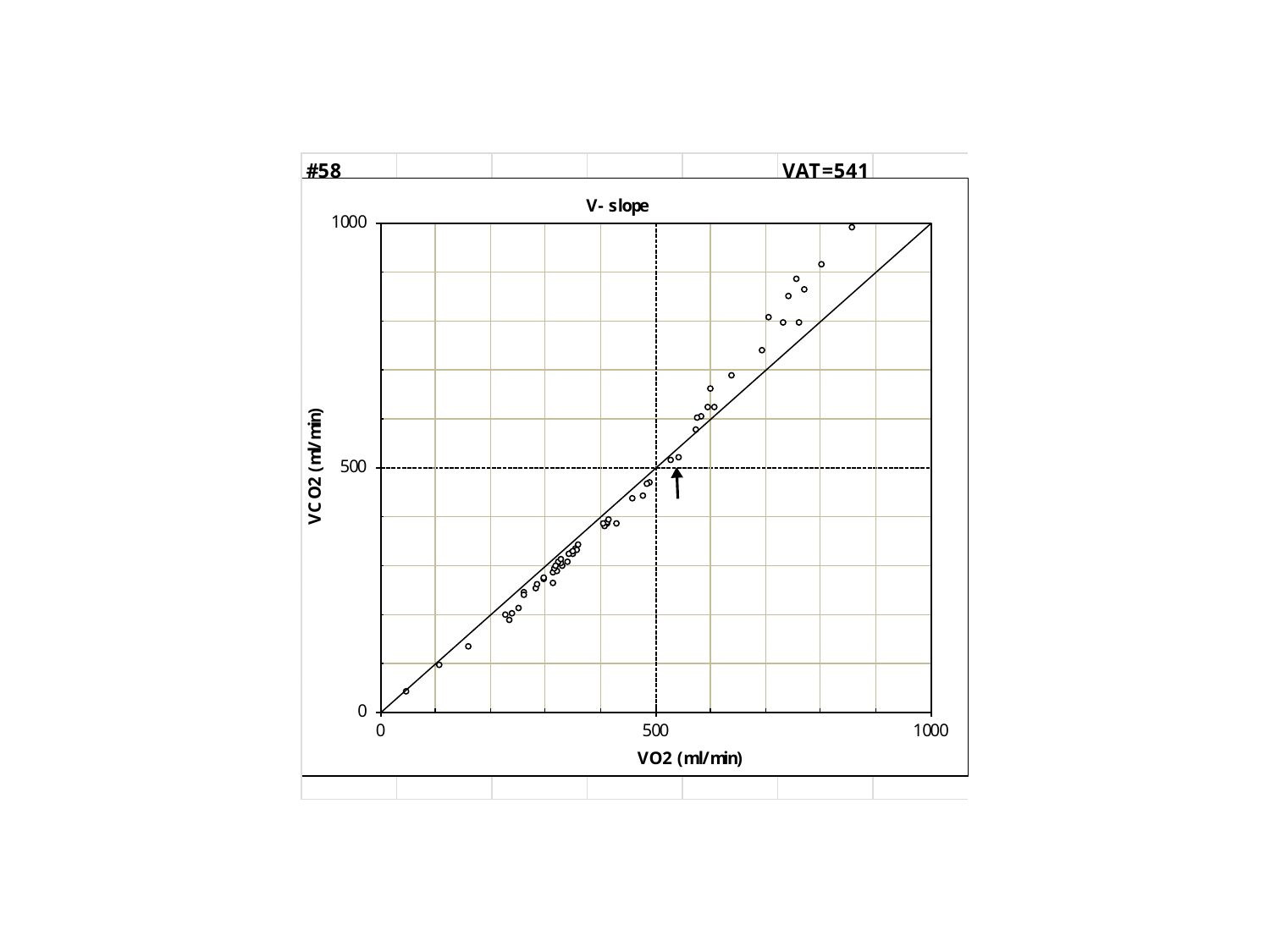

## Slide 61
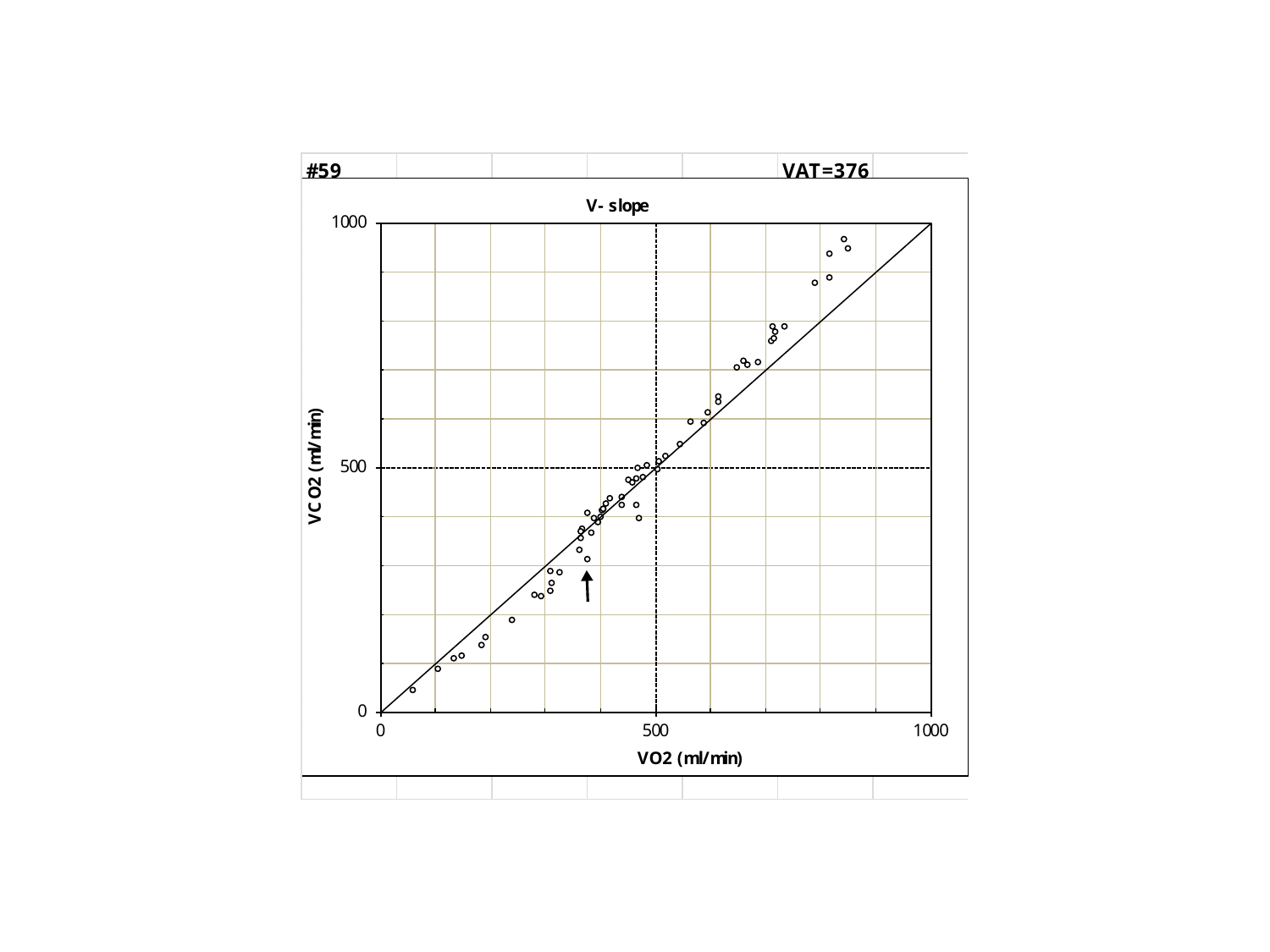

## Slide 62
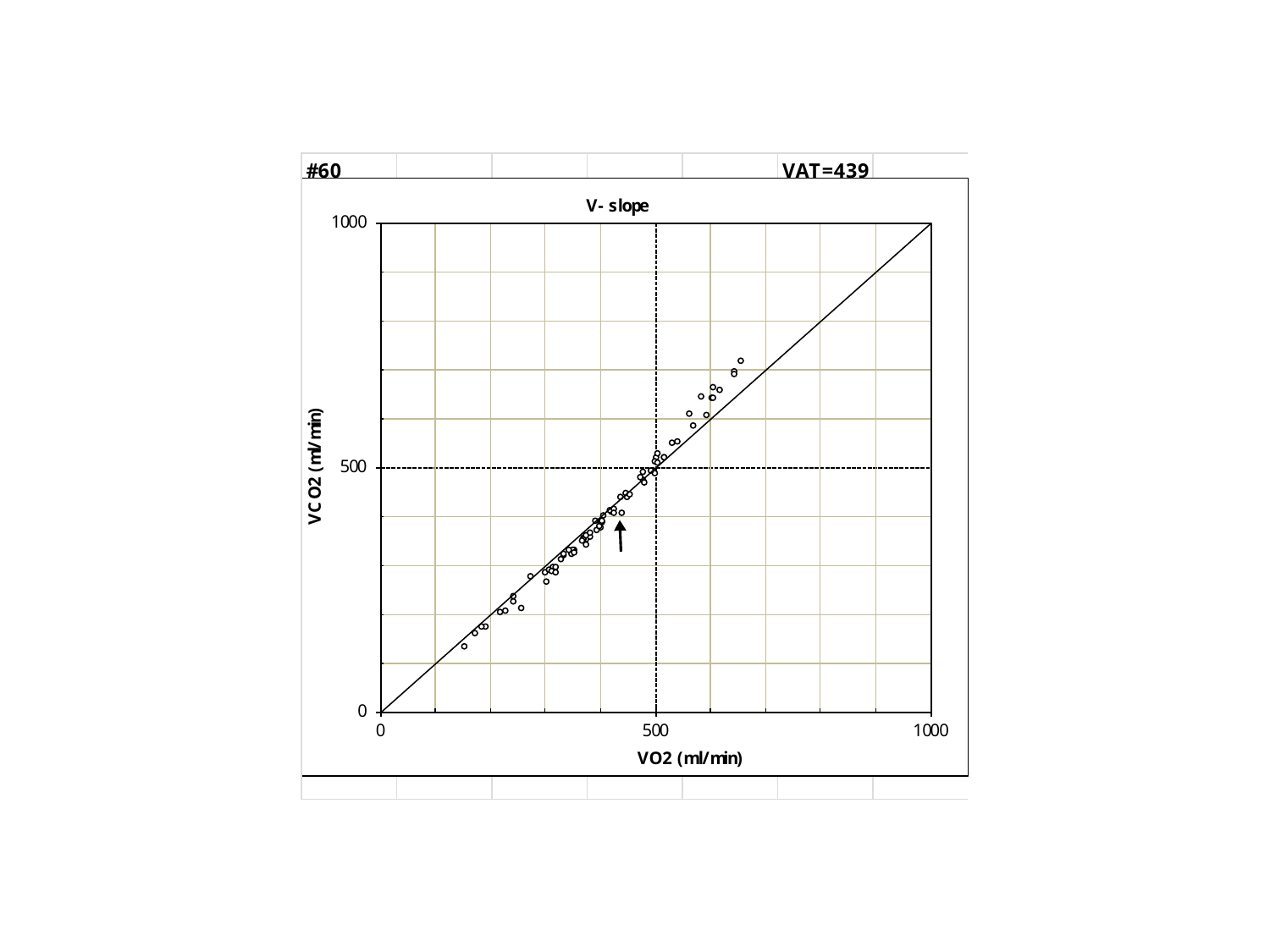

## Slide 63
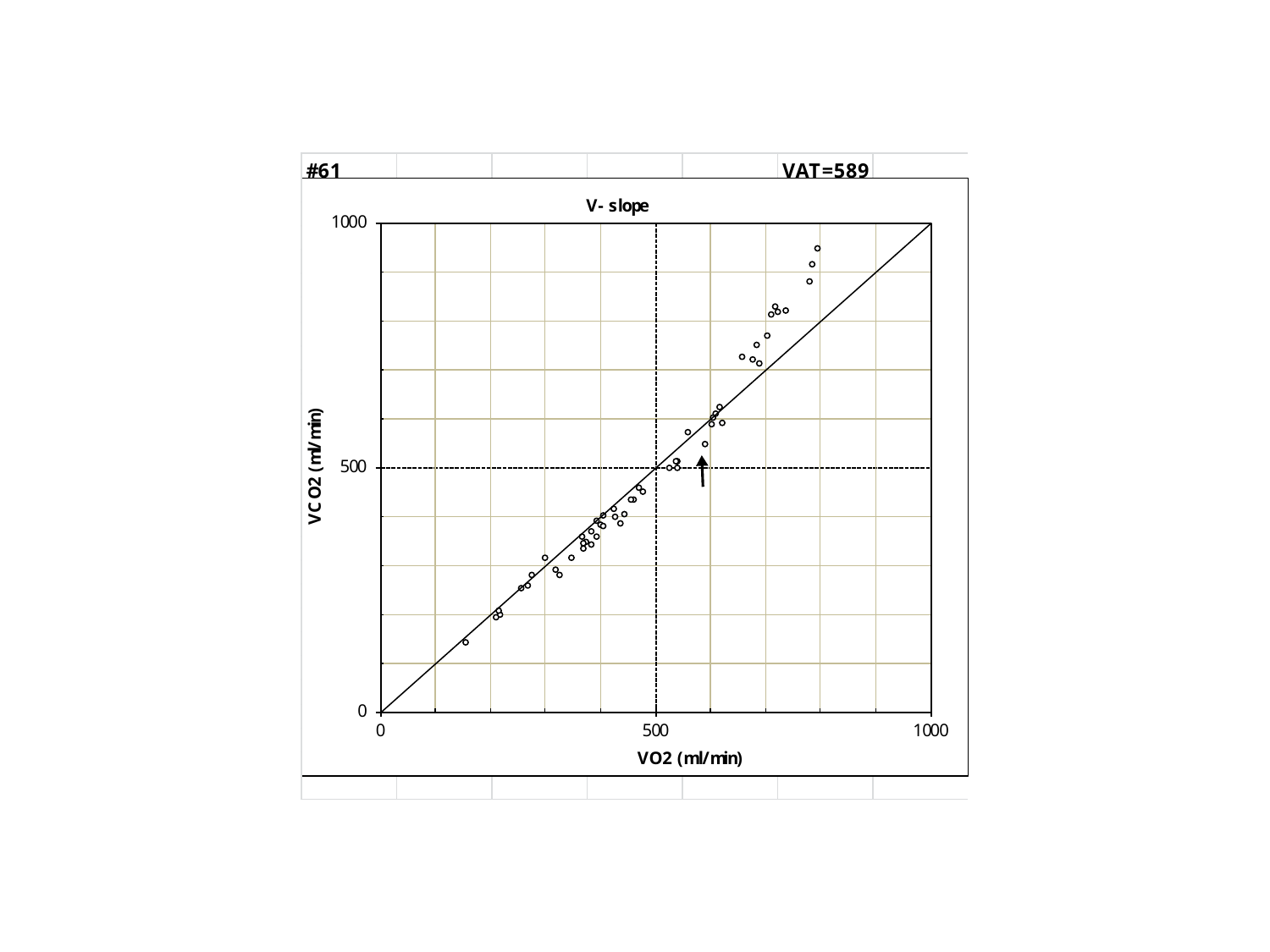

## Slide 64
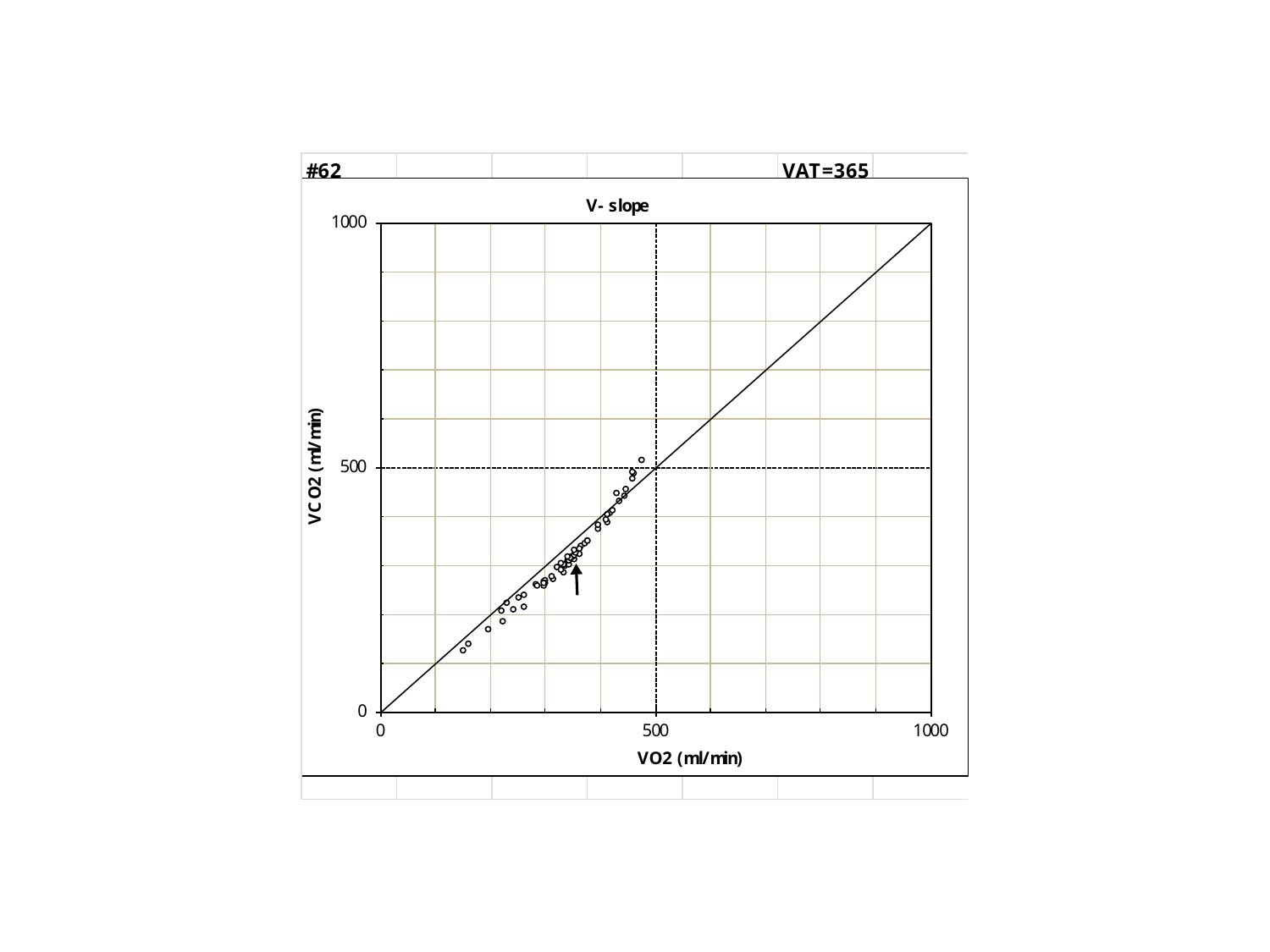

## Slide 65
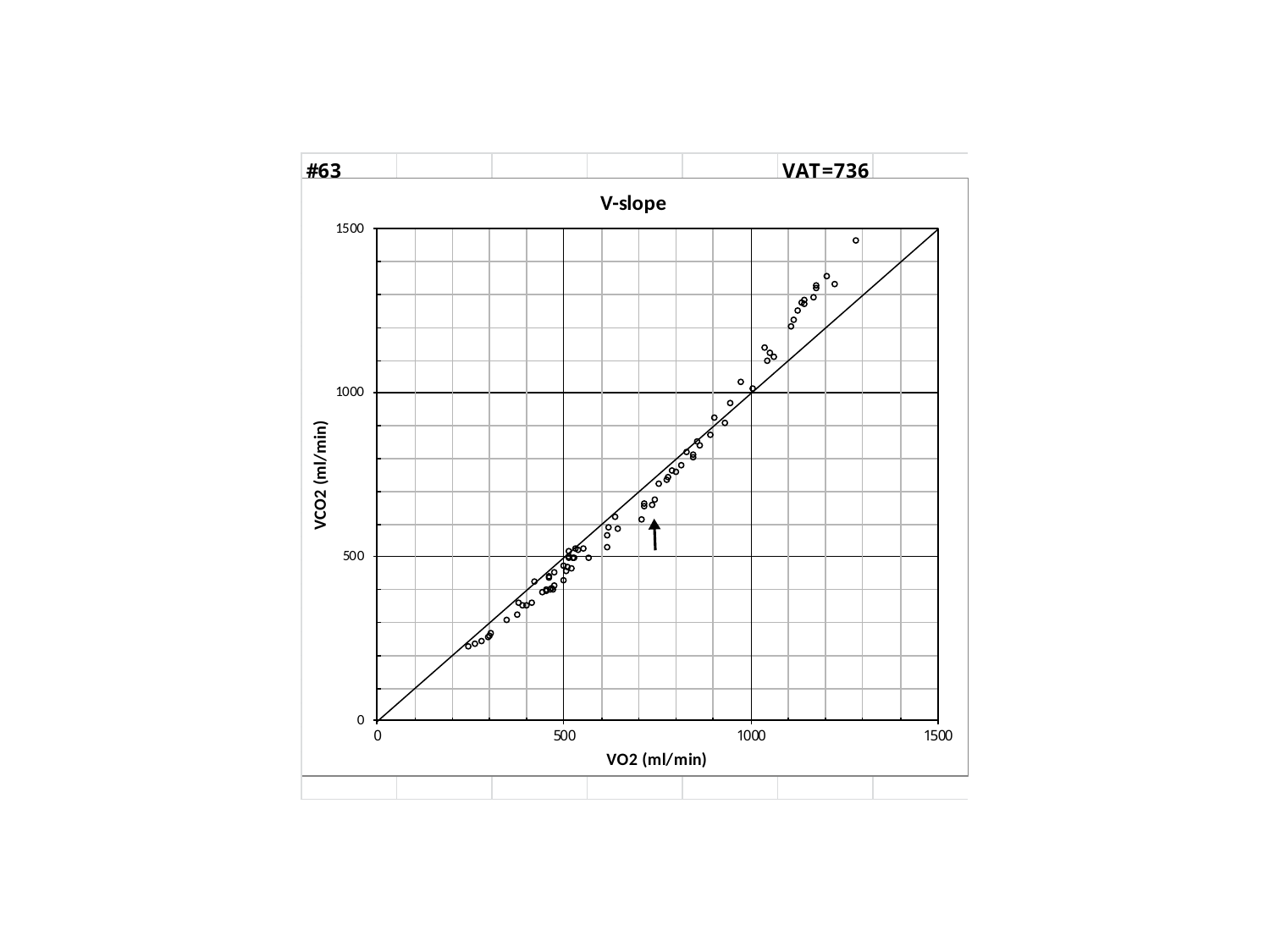

## Slide 66
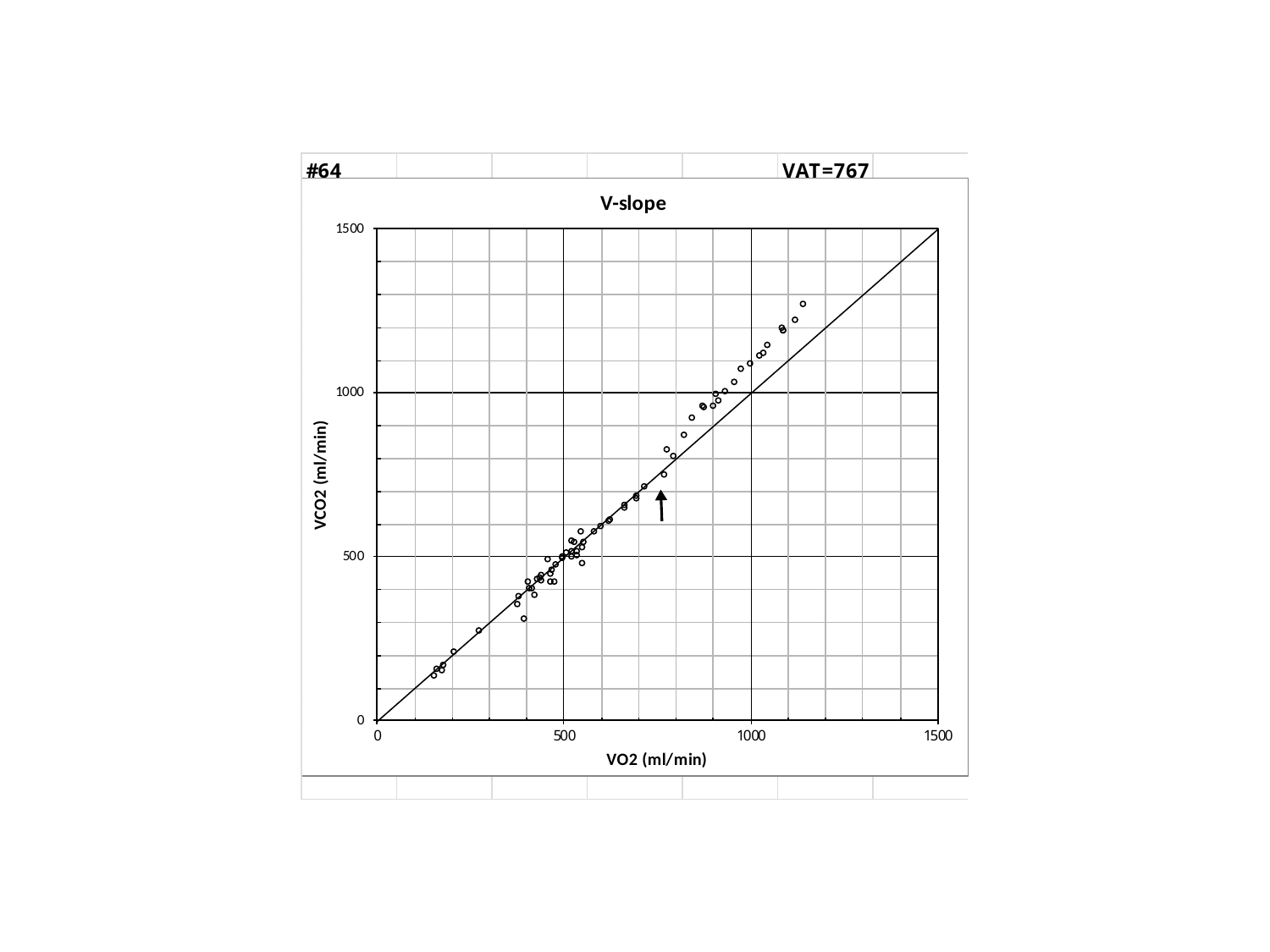

## Slide 67
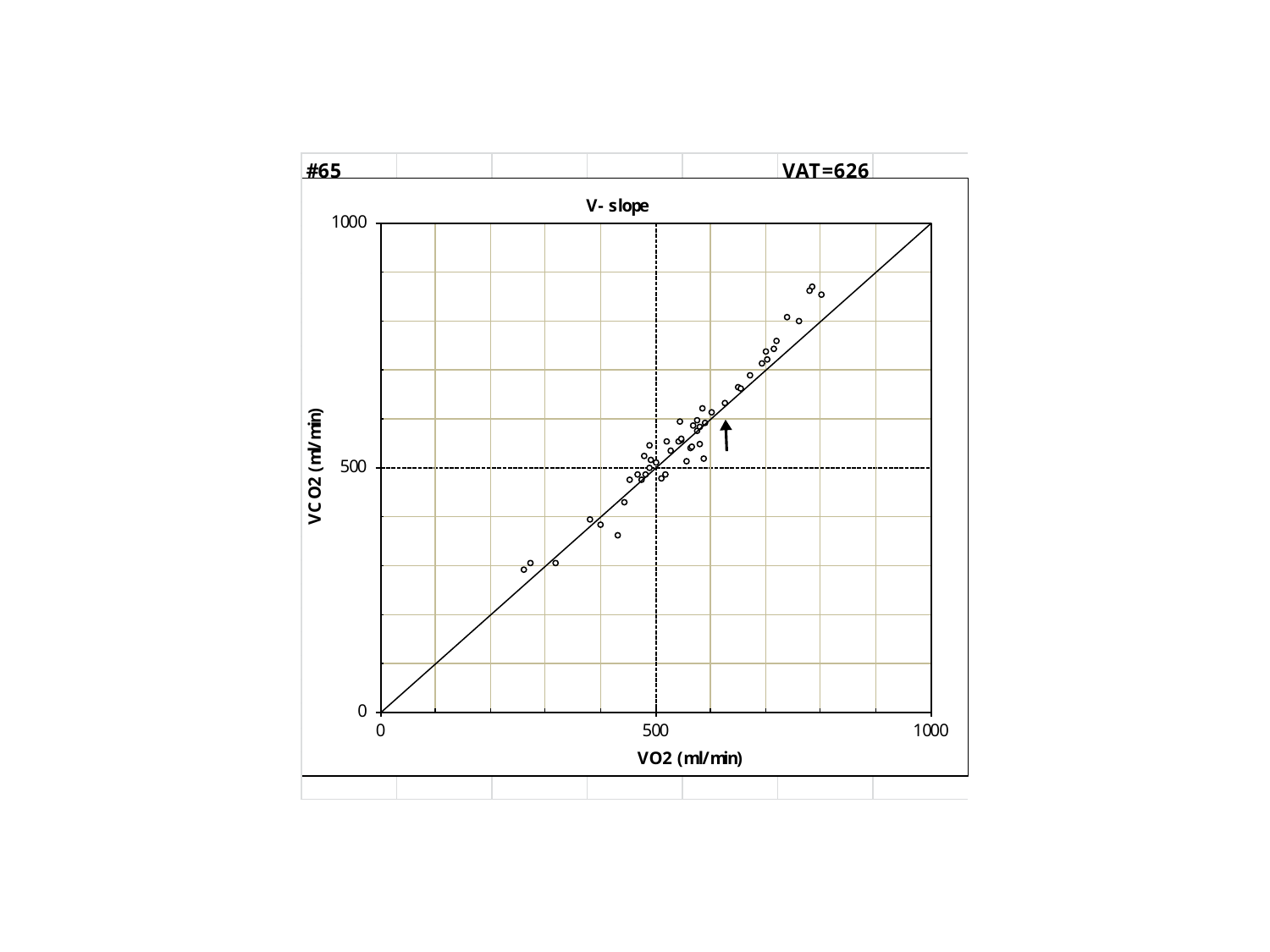

## Slide 68
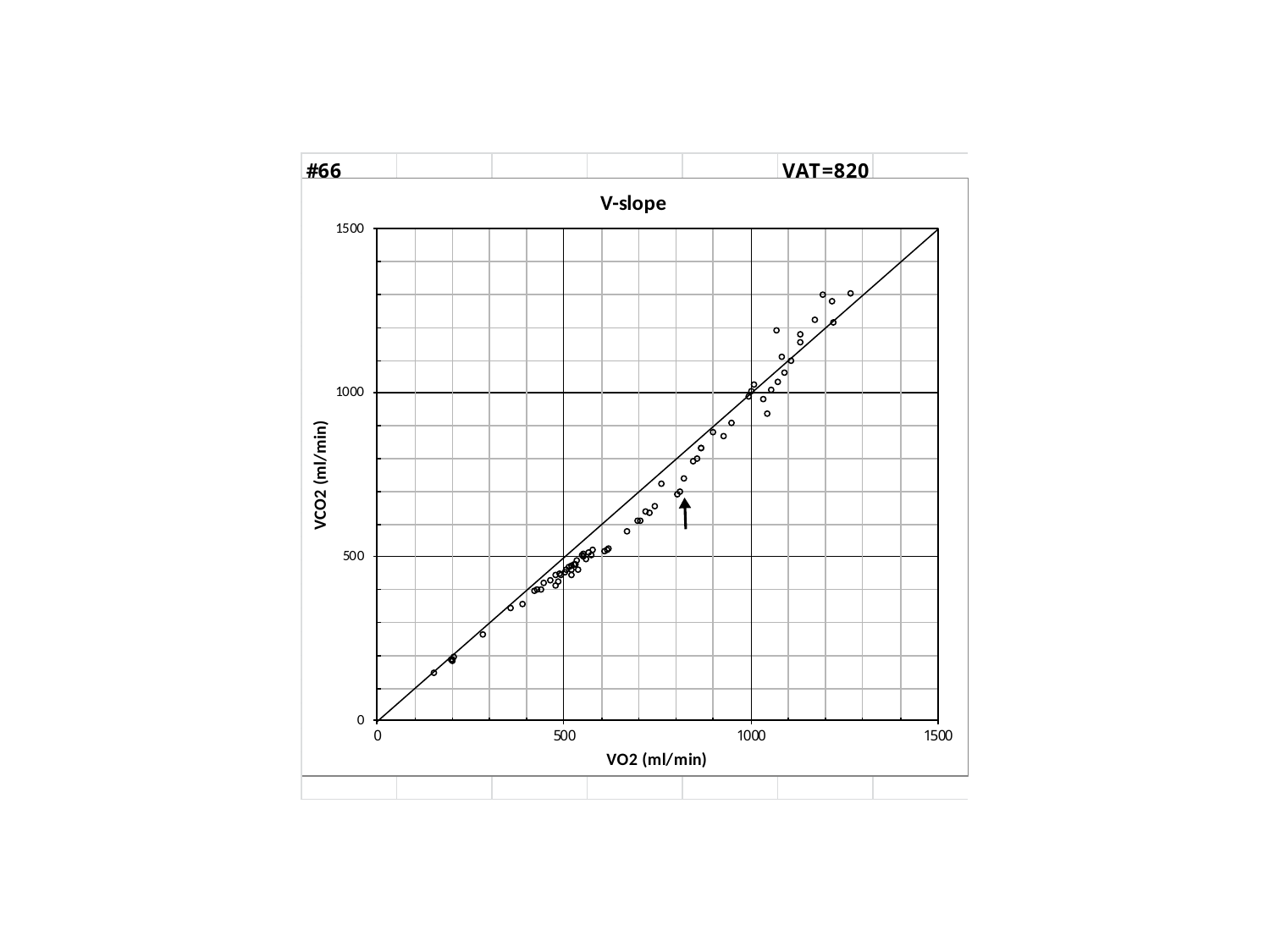

## Slide 69
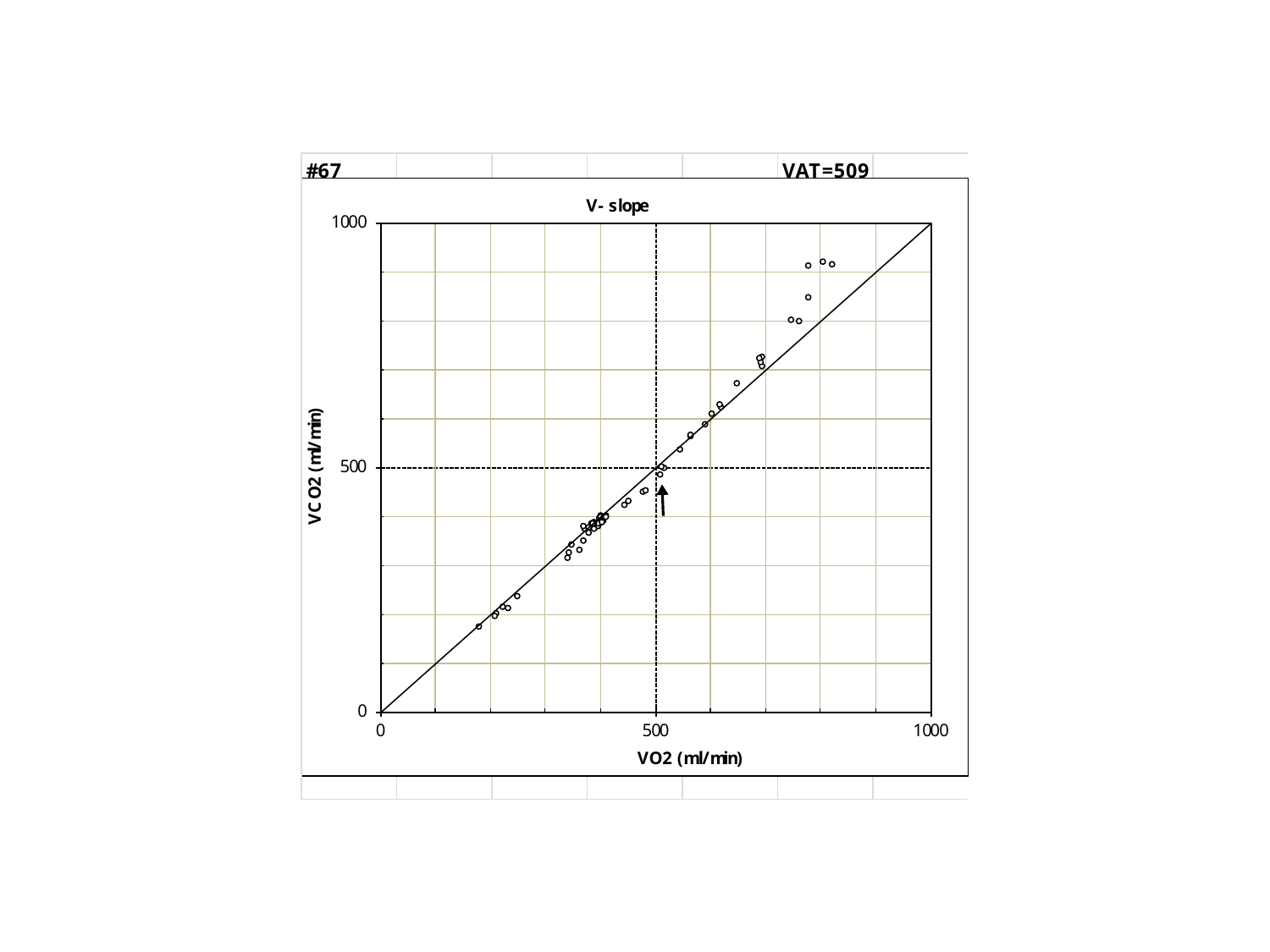

## Slide 70
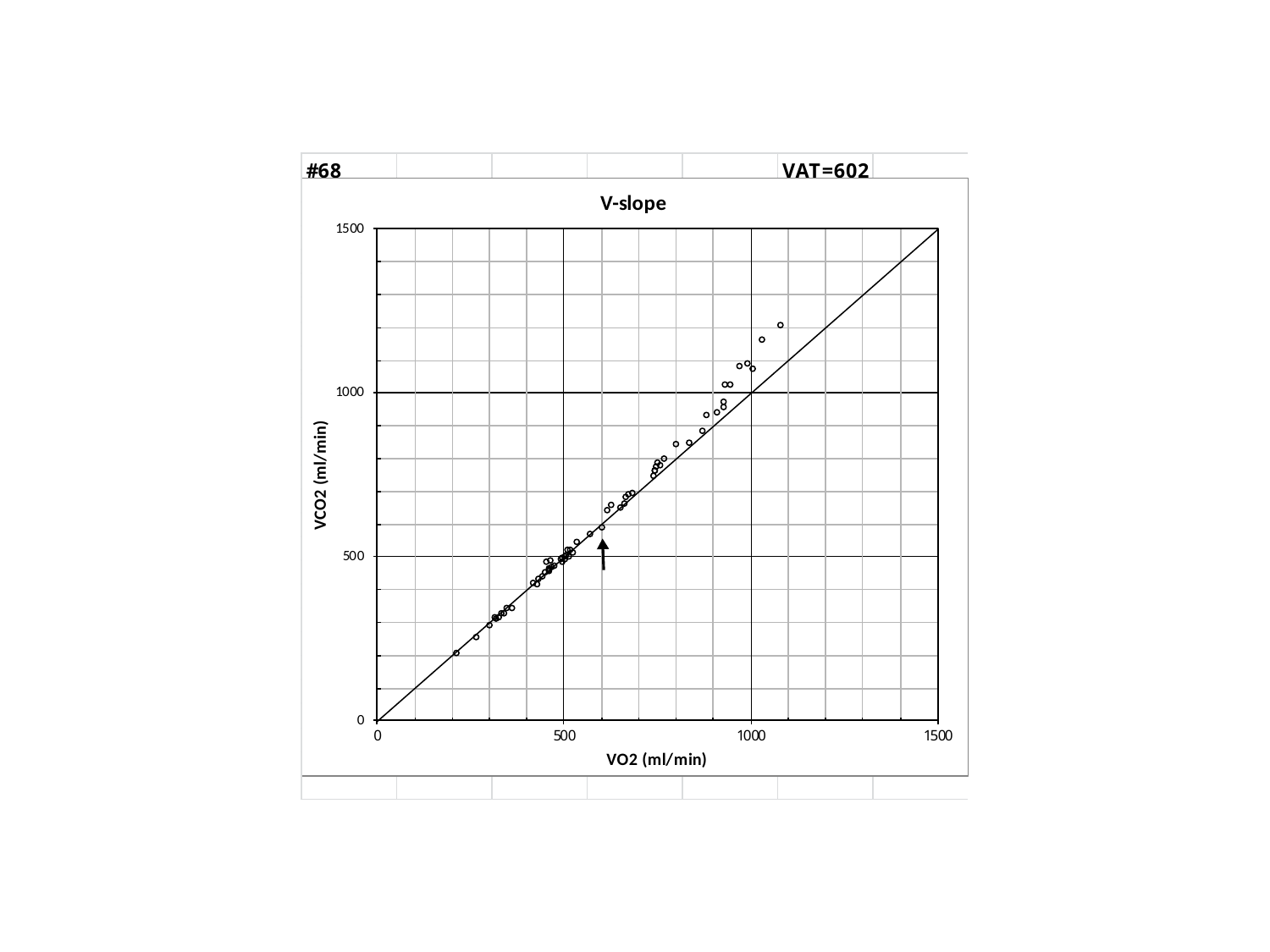

## Slide 71
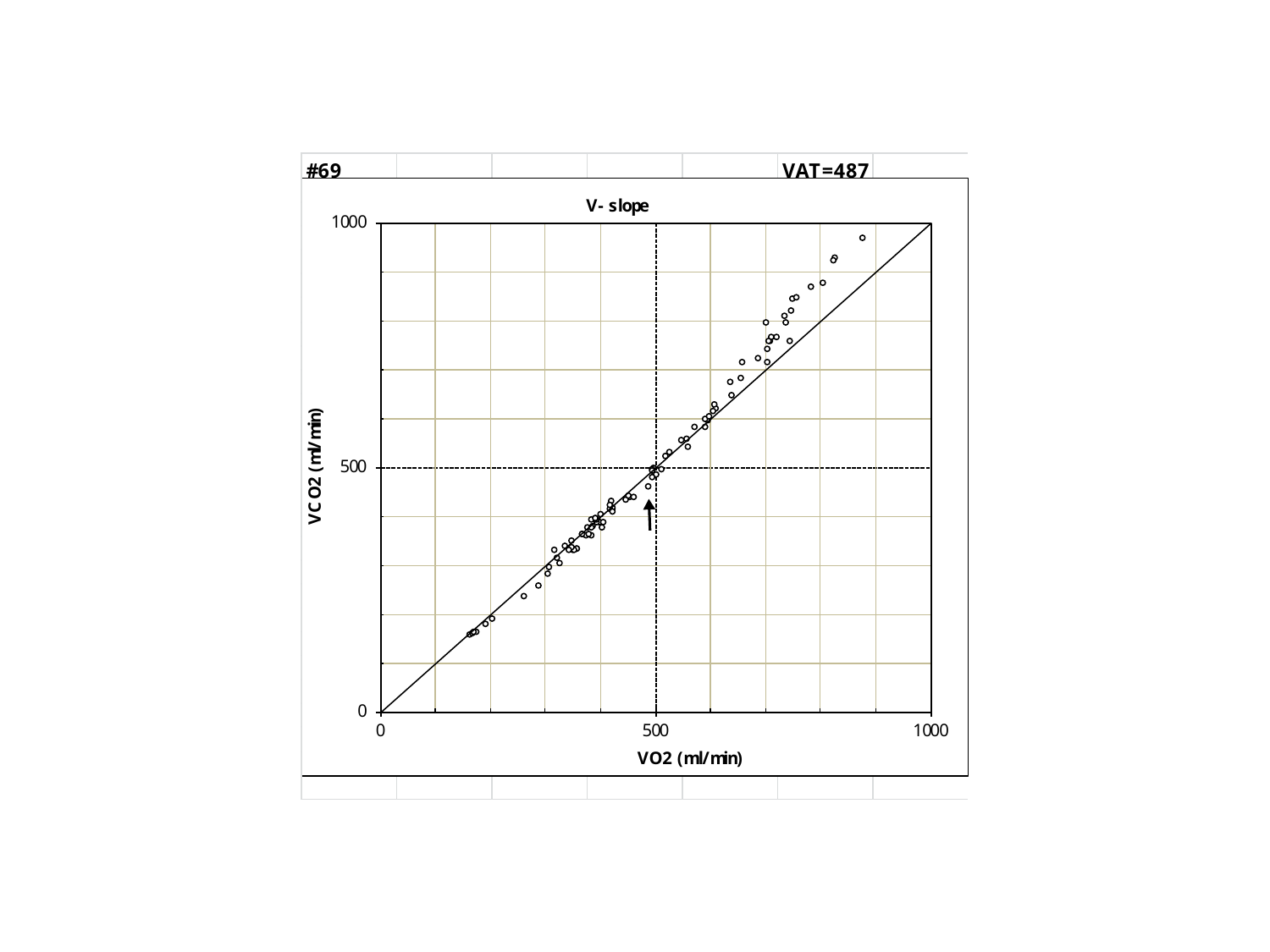

## Slide 72
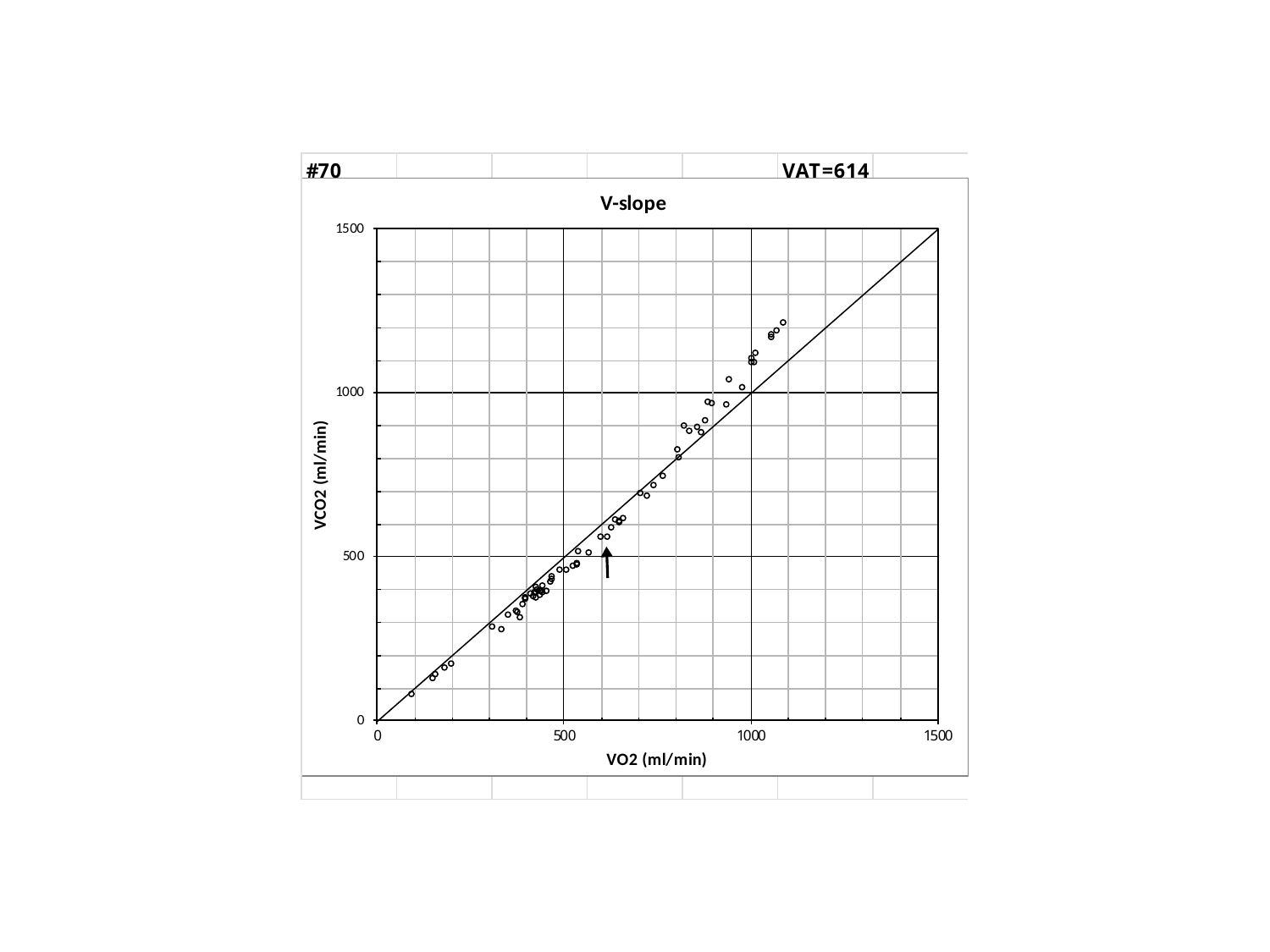

## Slide 73
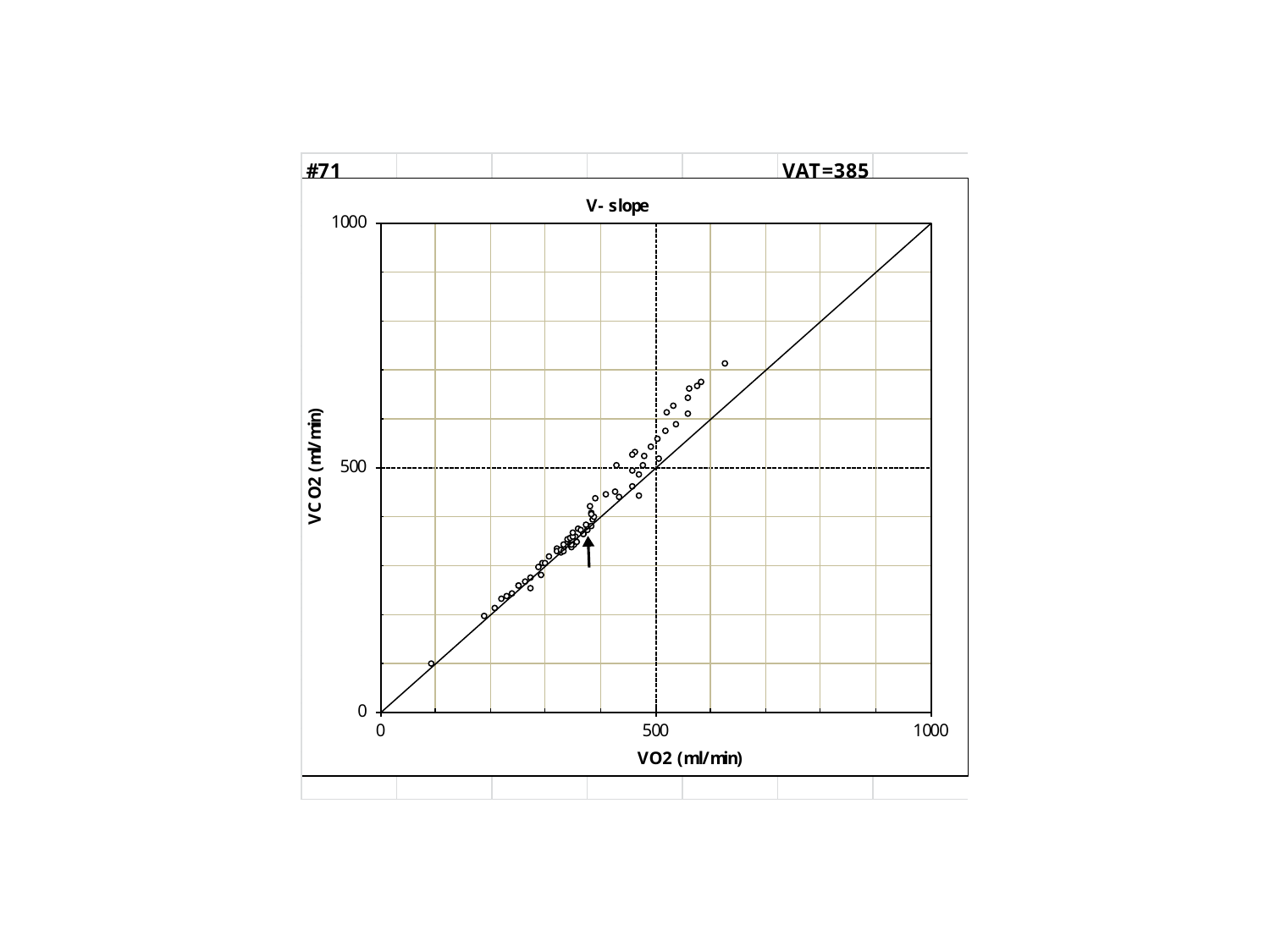

## Slide 74
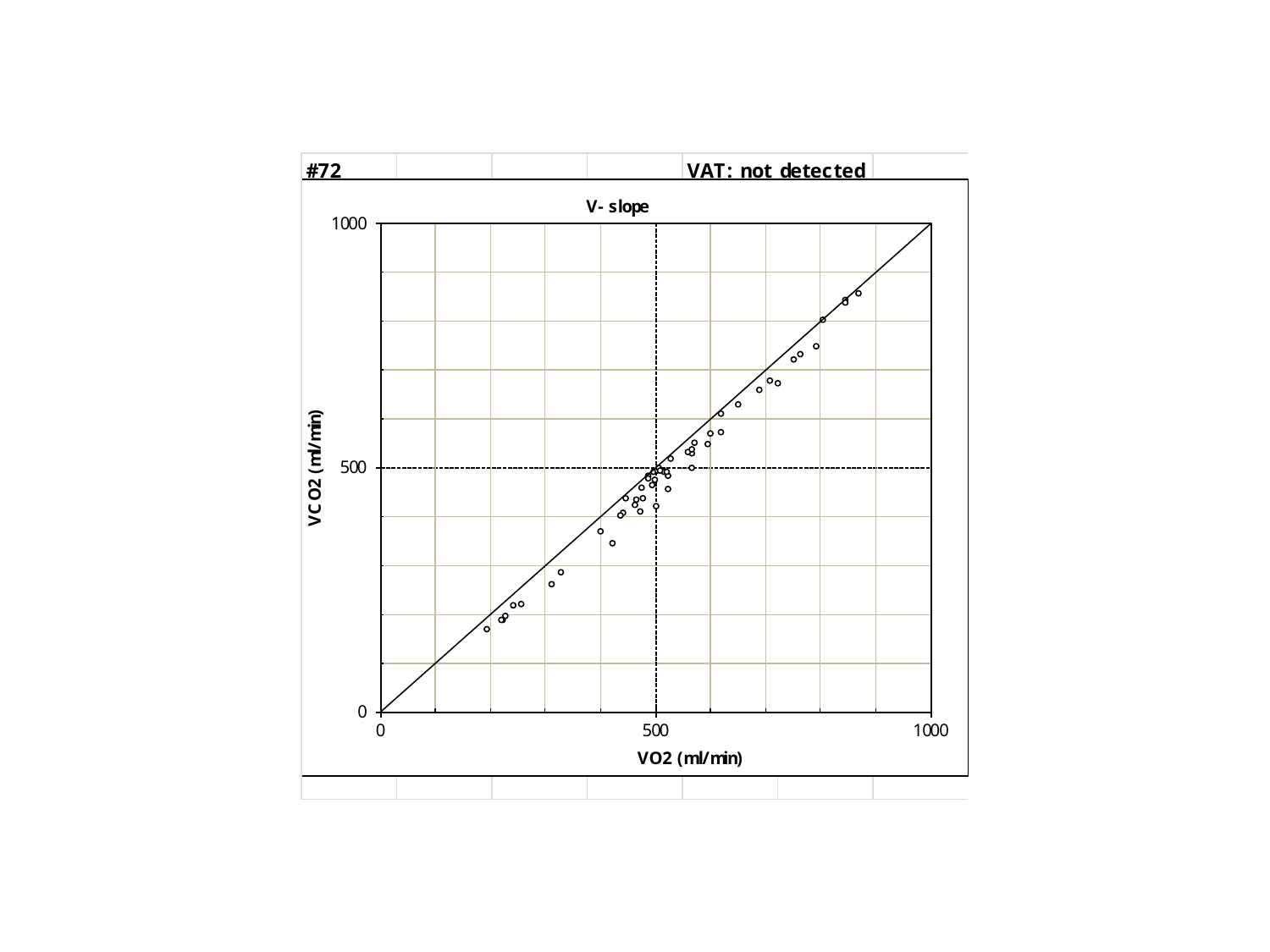

## Slide 75
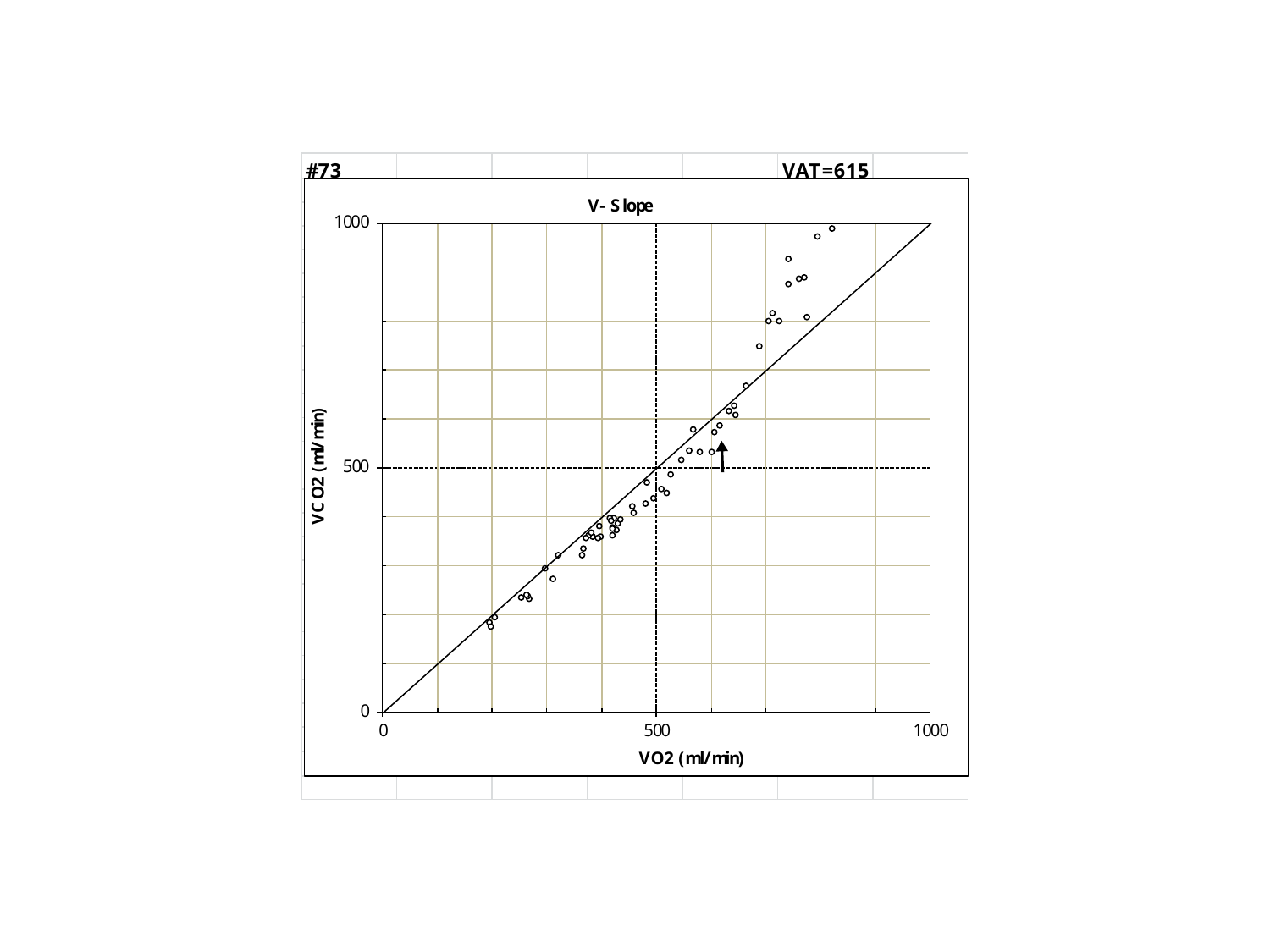

## Slide 76
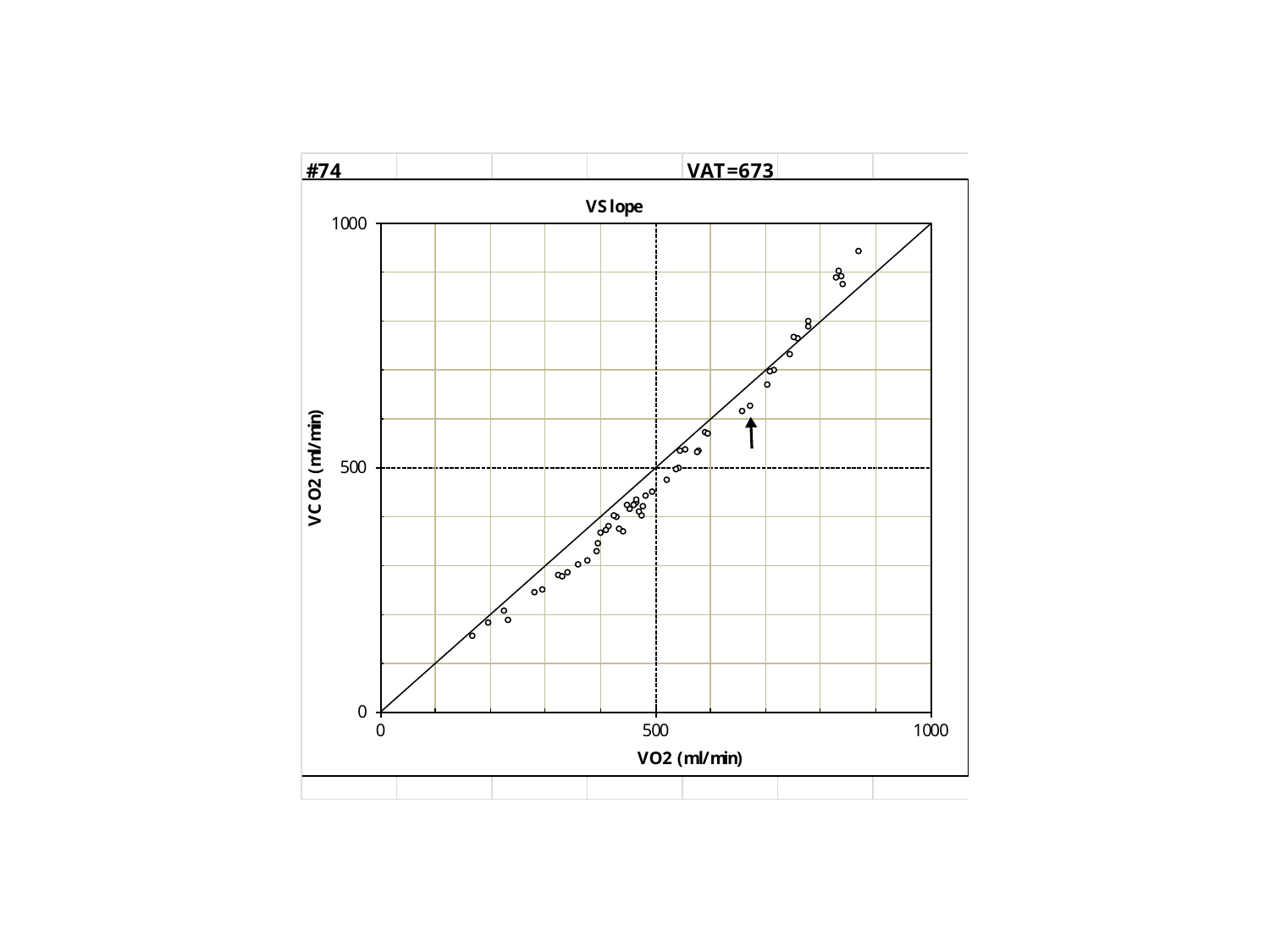

## Slide 77
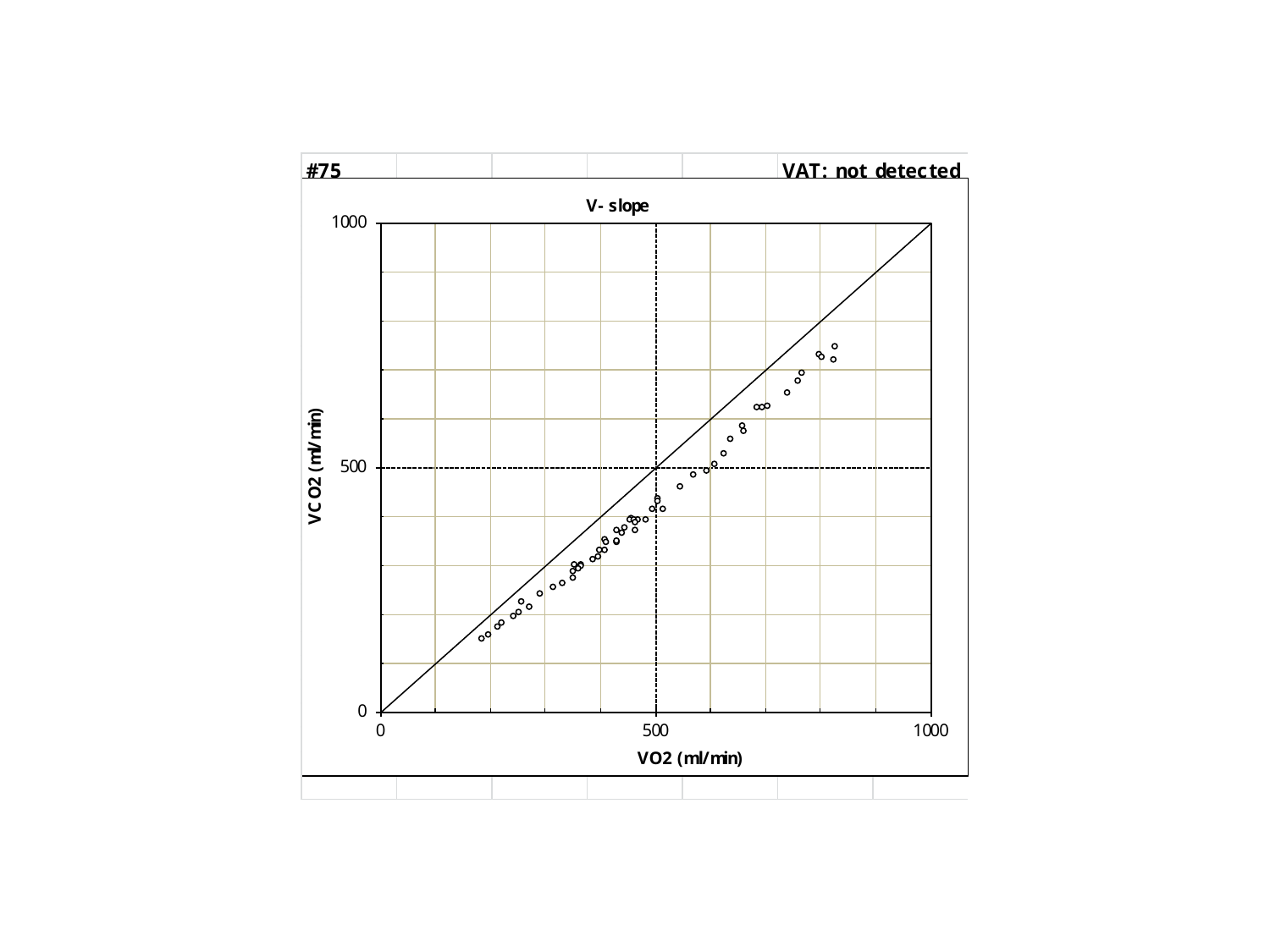

## Slide 78
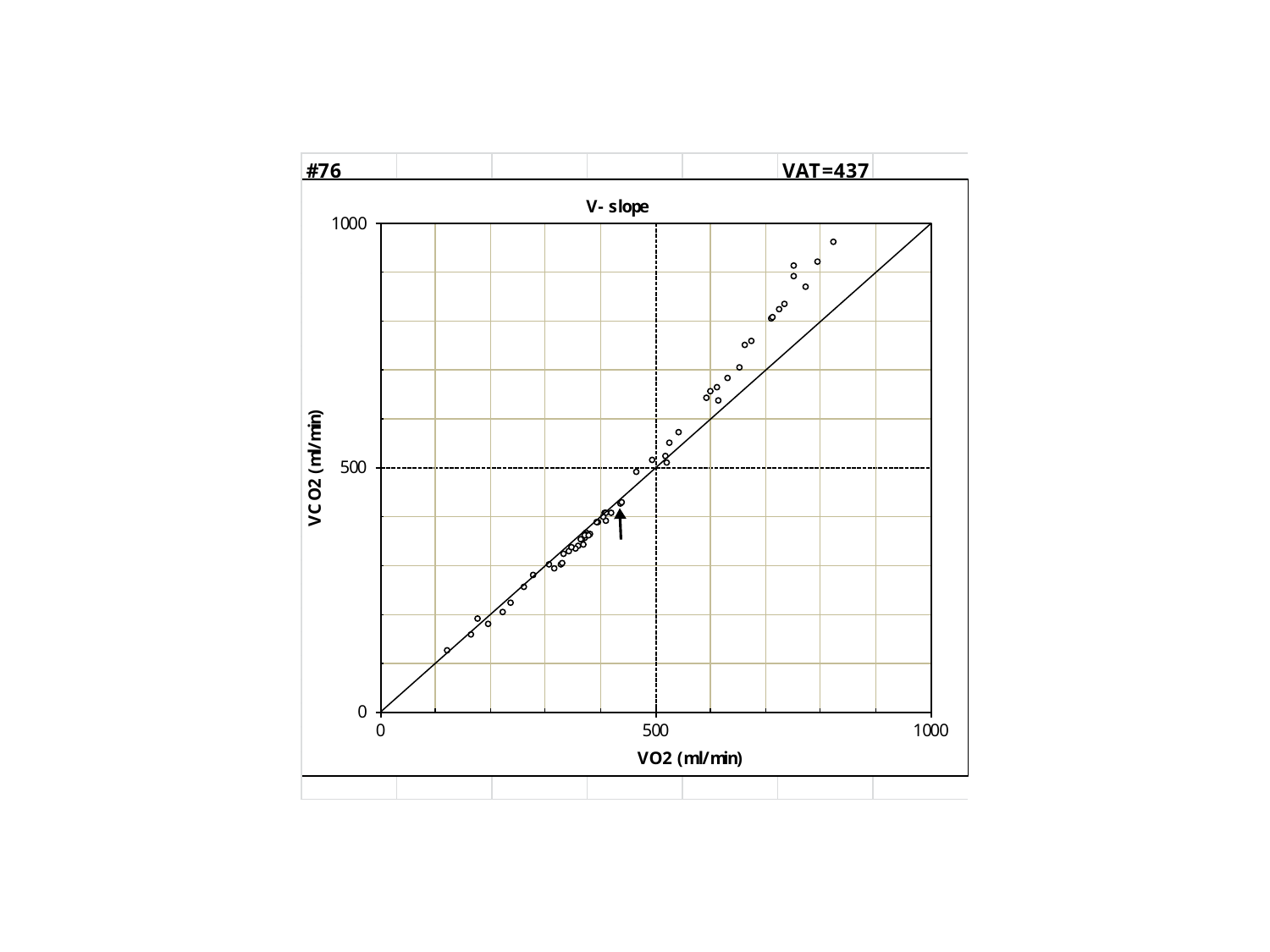

## Slide 79
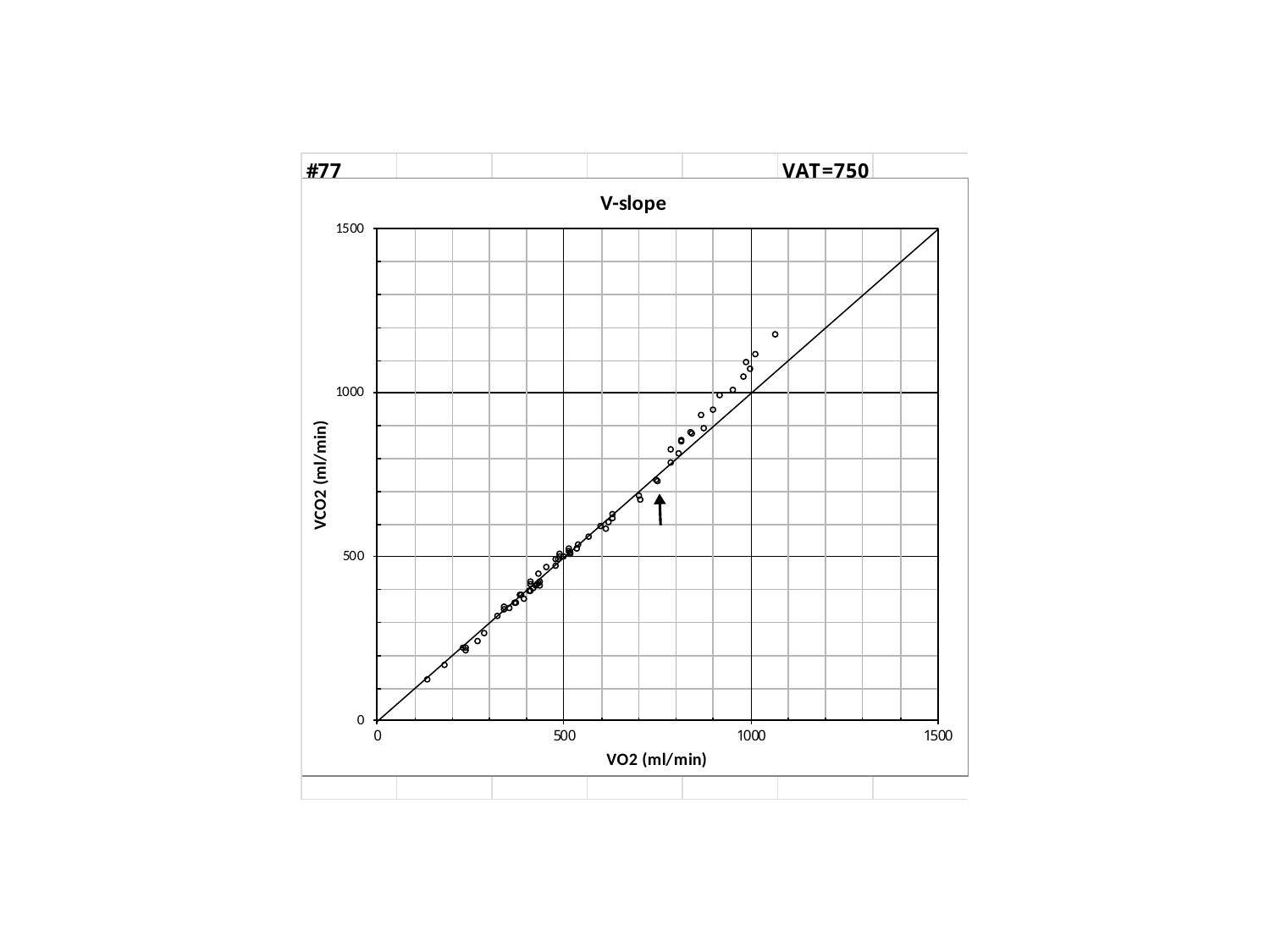

## Slide 80
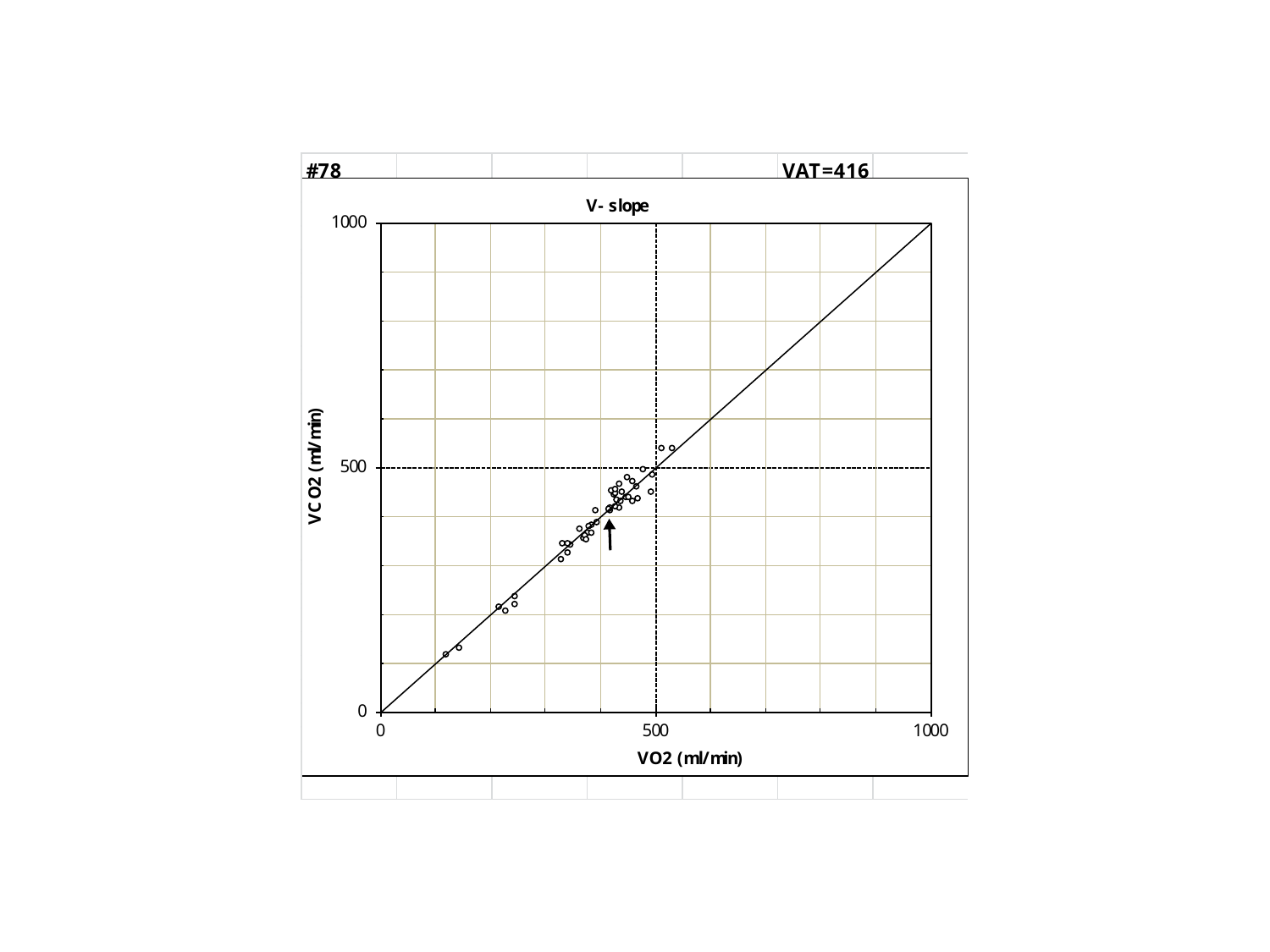

## Slide 81
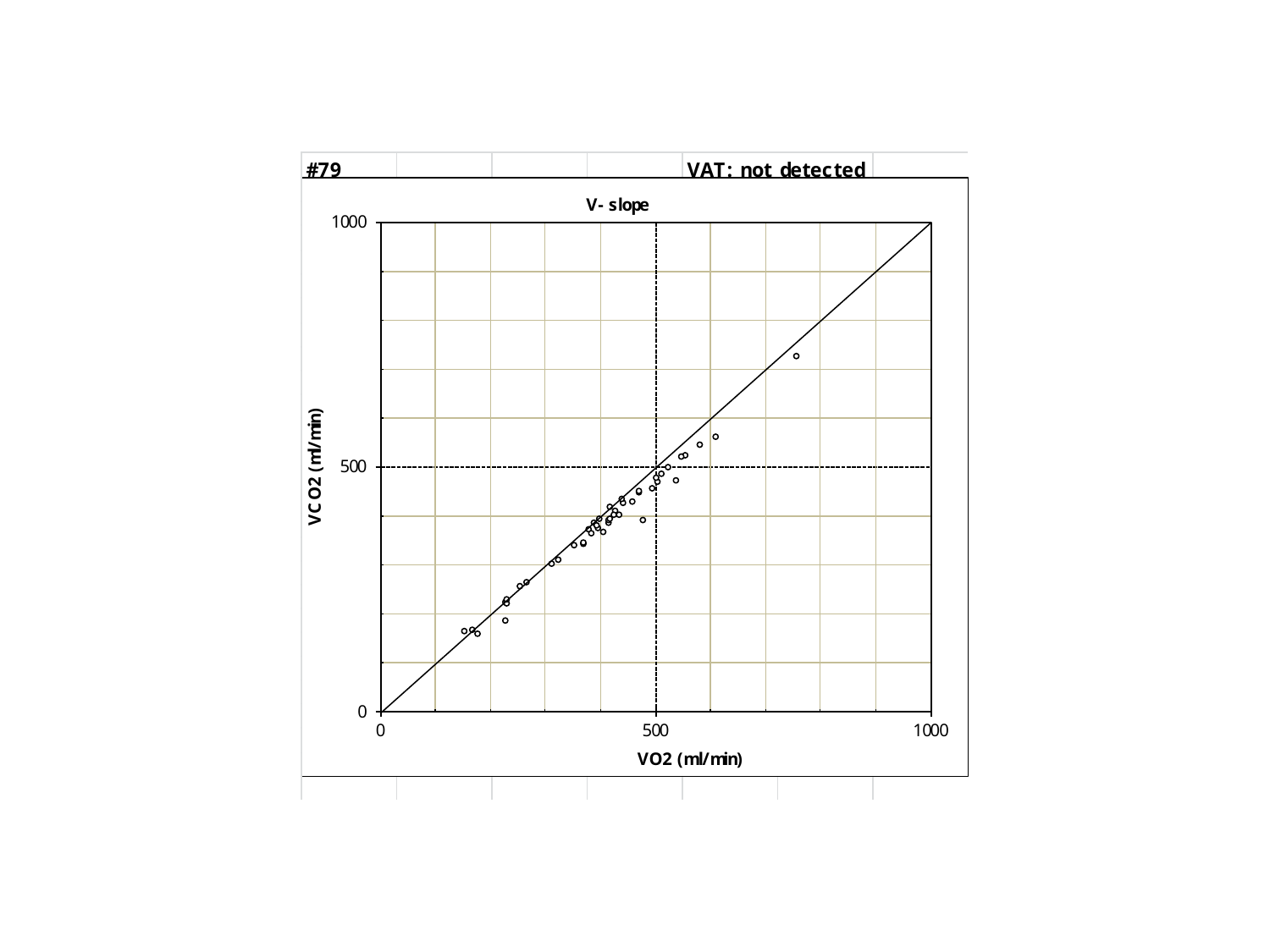

## Slide 82
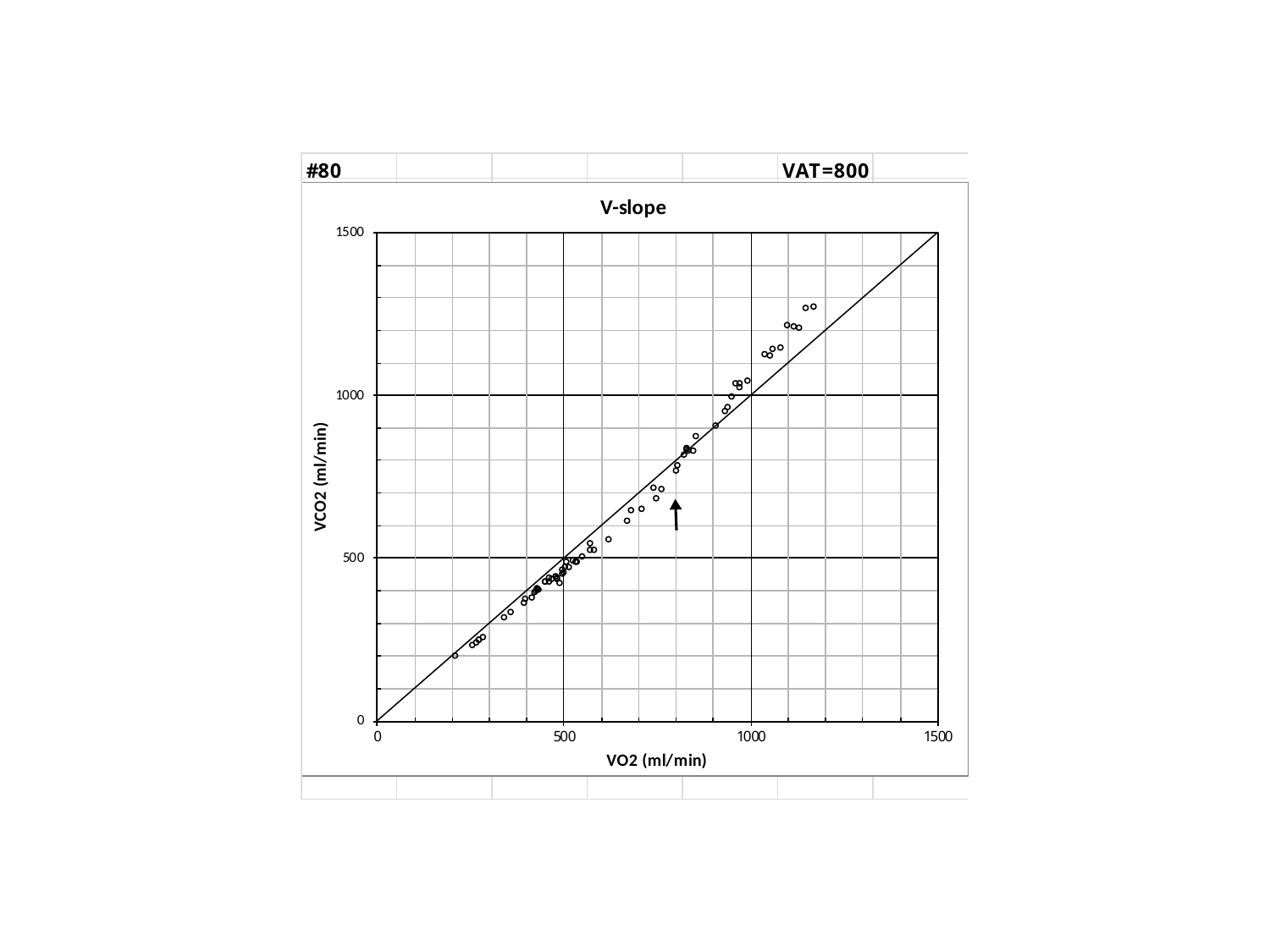

## Slide 83
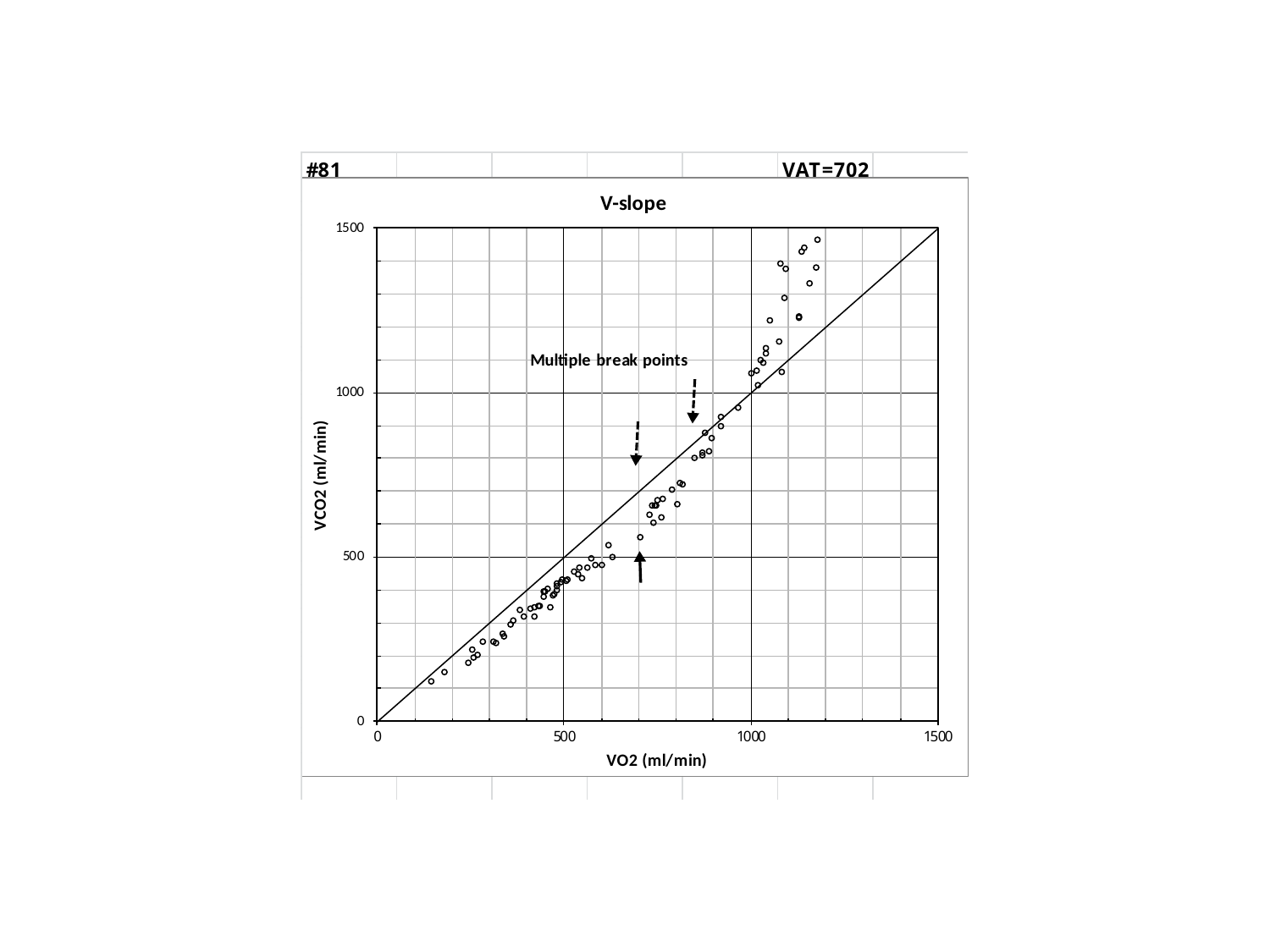

## Slide 84
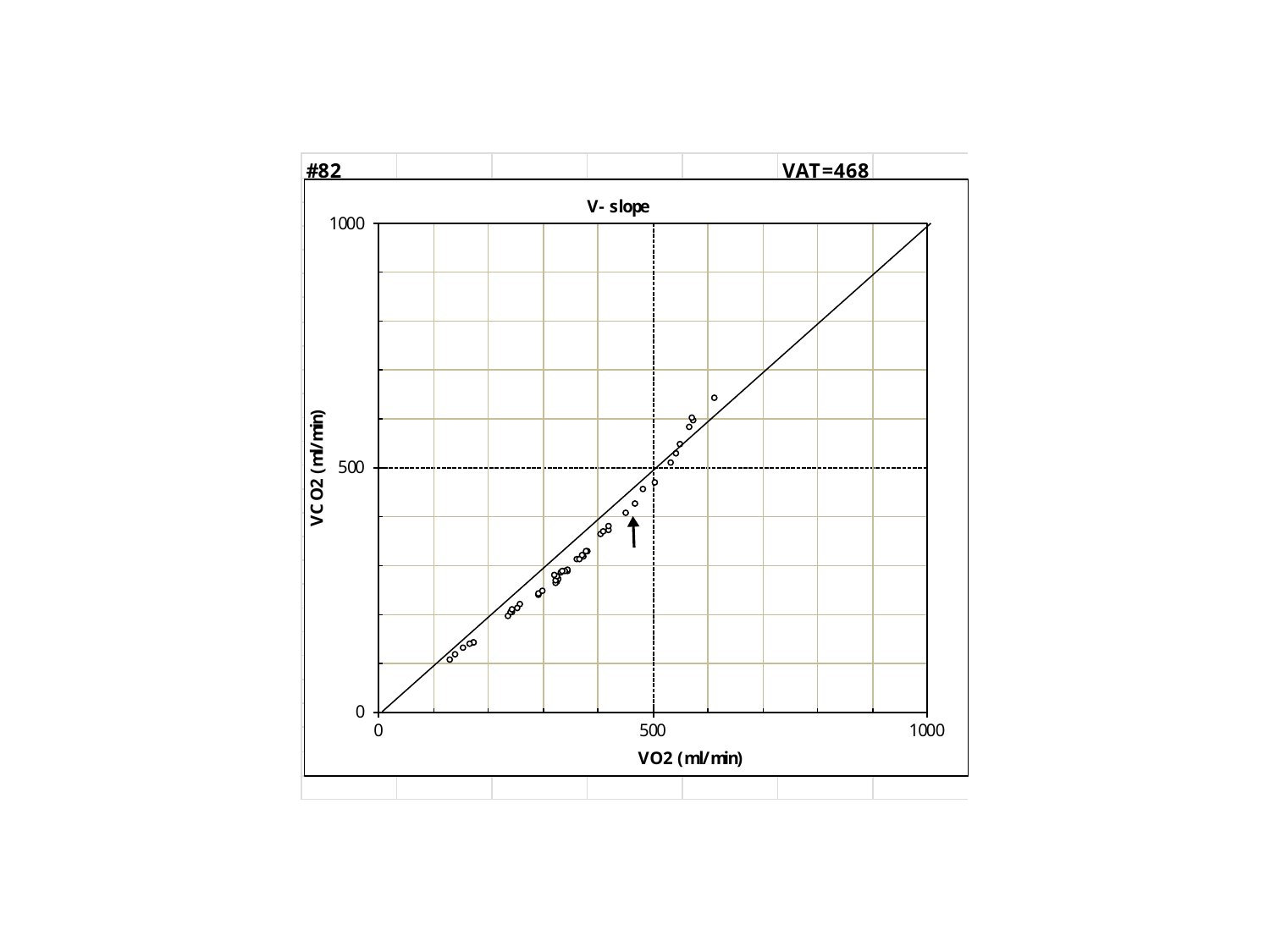

## Slide 85
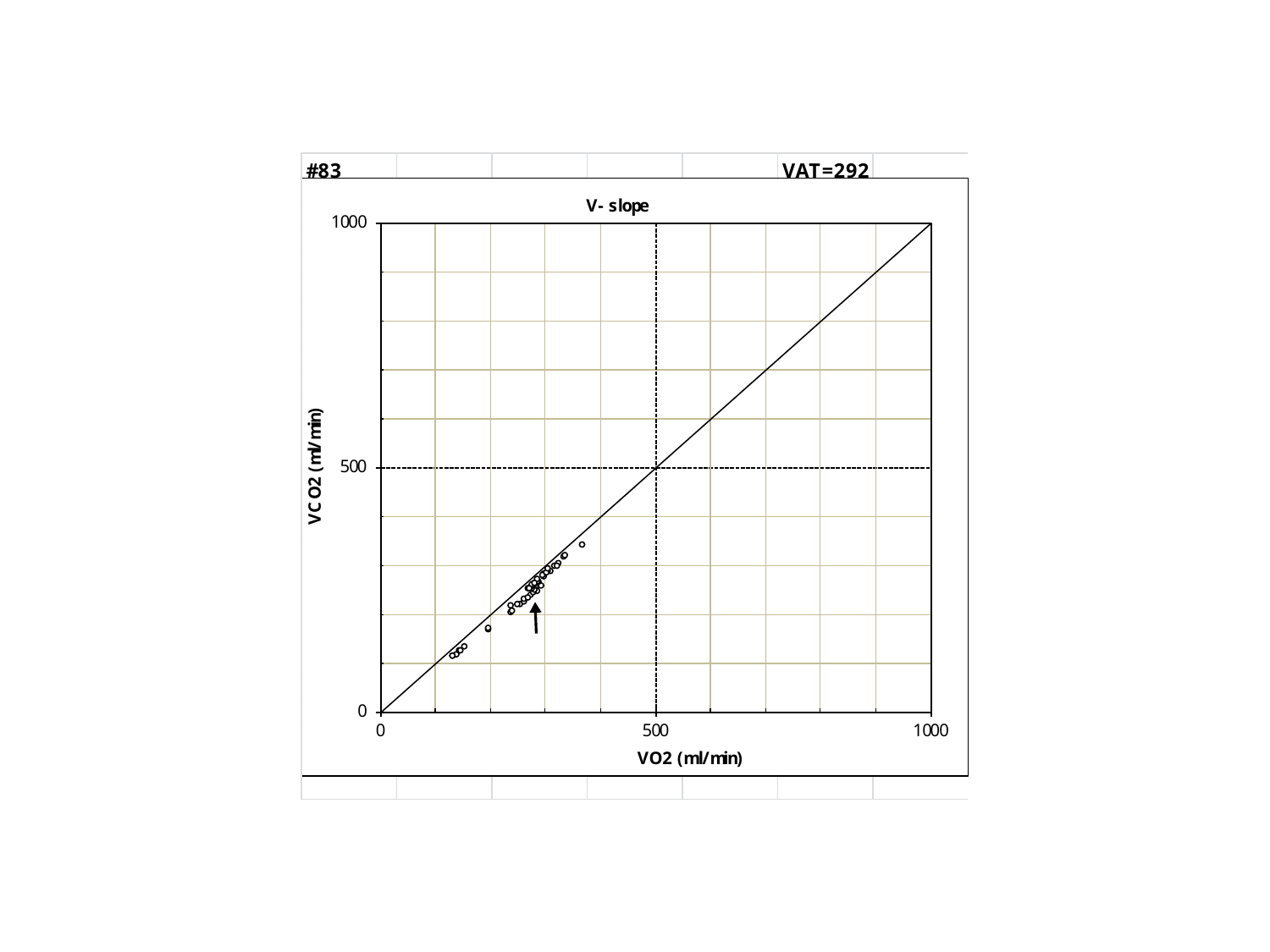

## Slide 86
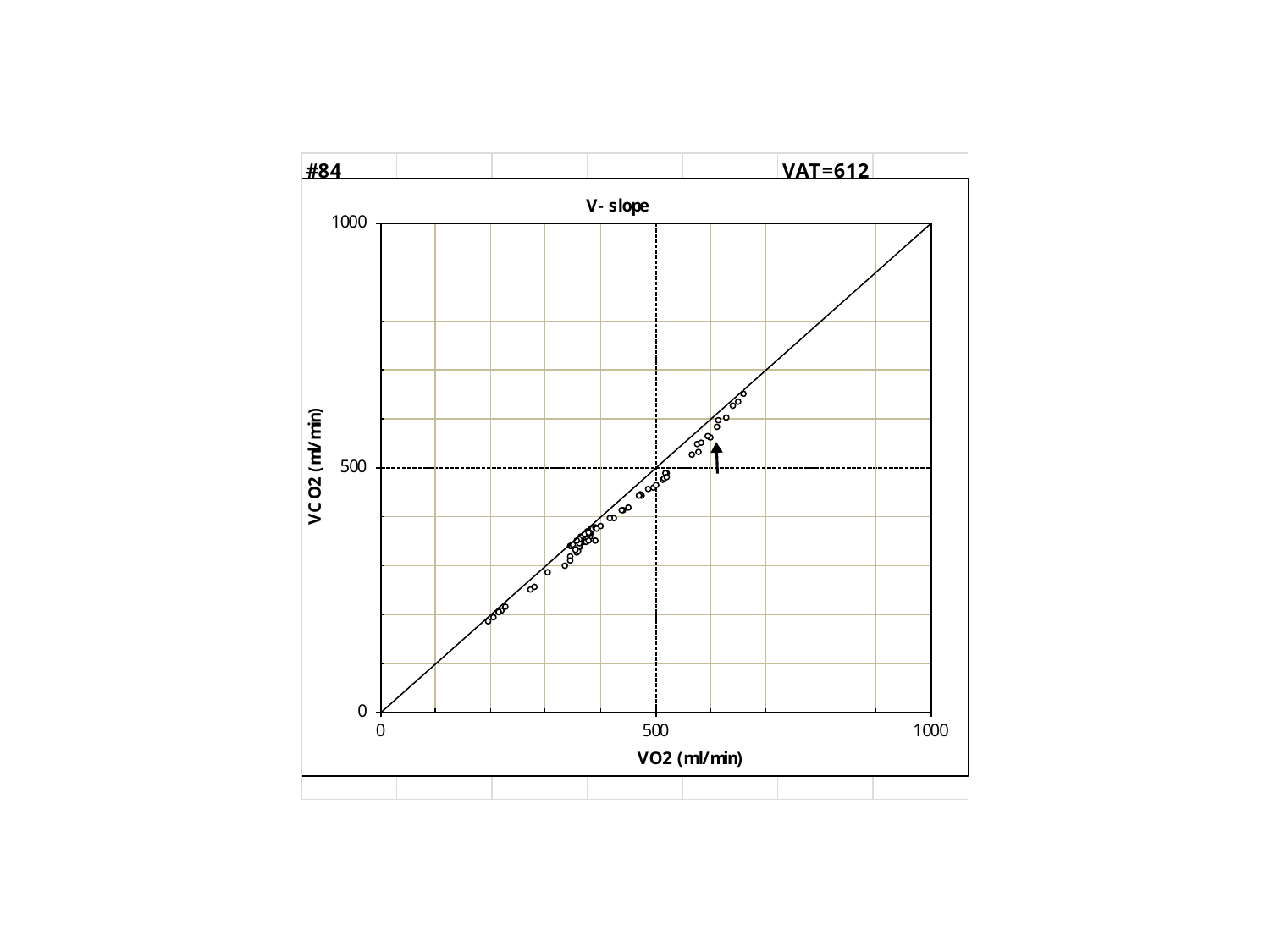

## Slide 87
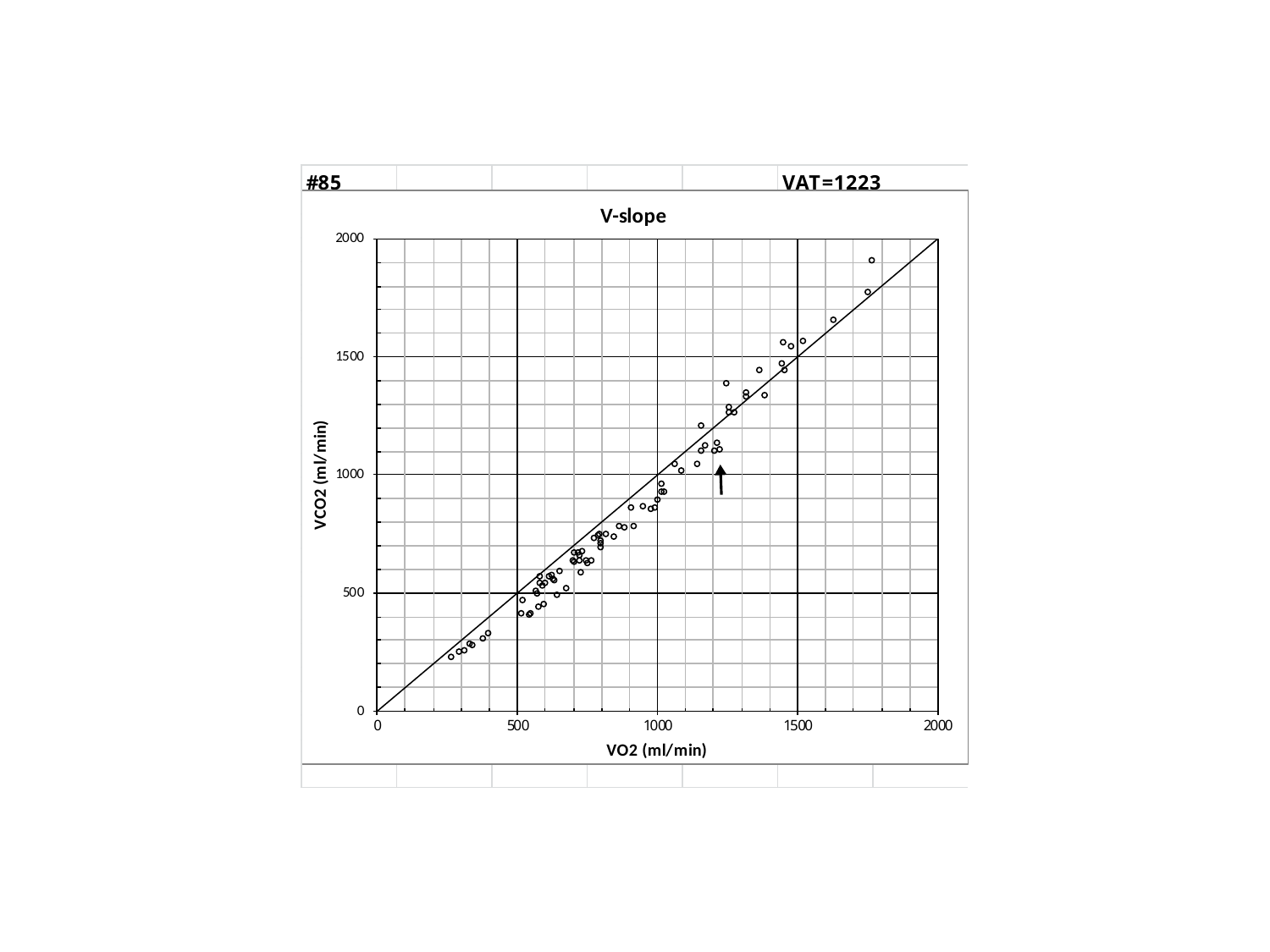

## Slide 88
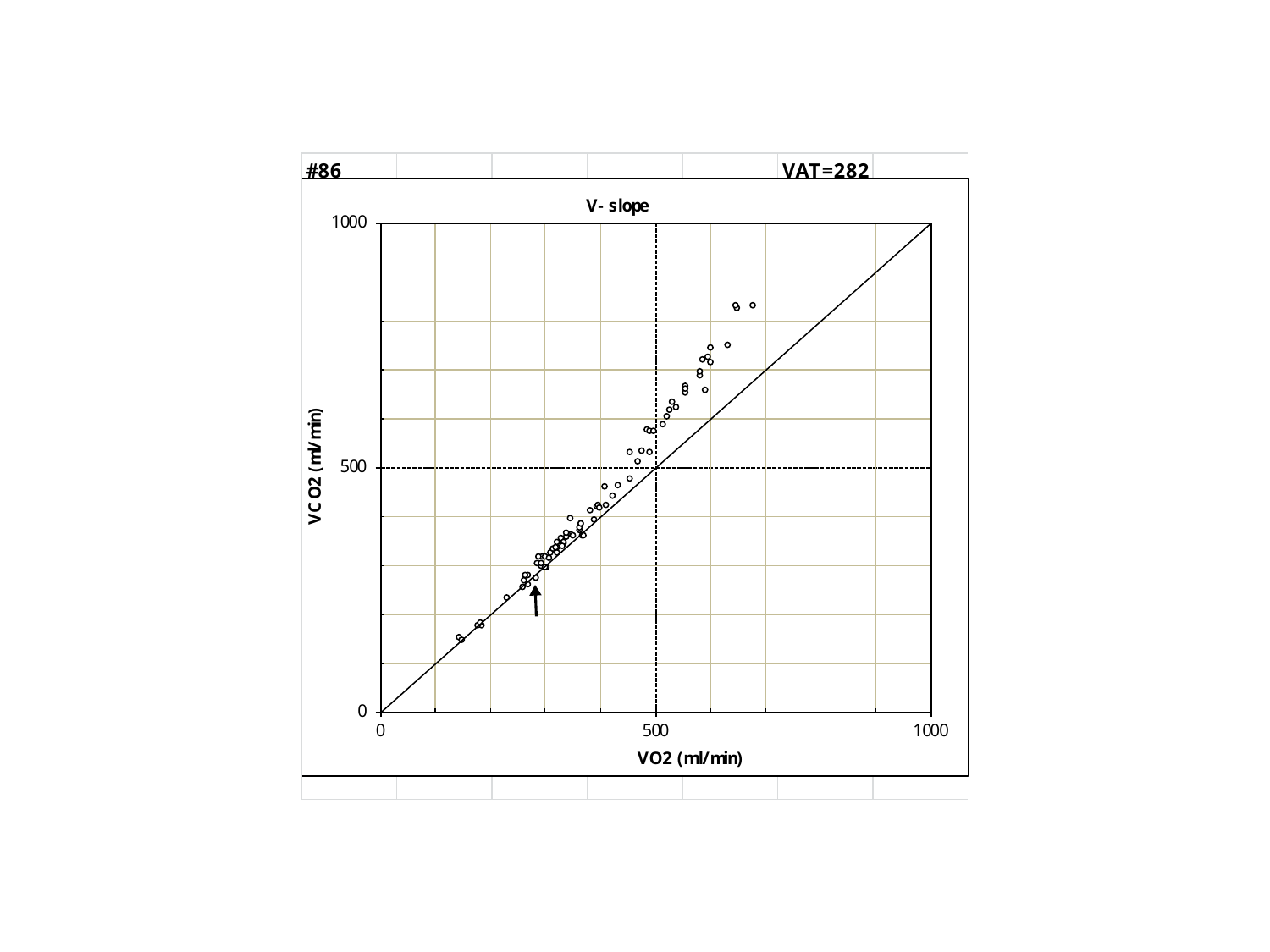

### Chart: V-slope
| Category | VCO2 |
|---|---|

## Slide 89
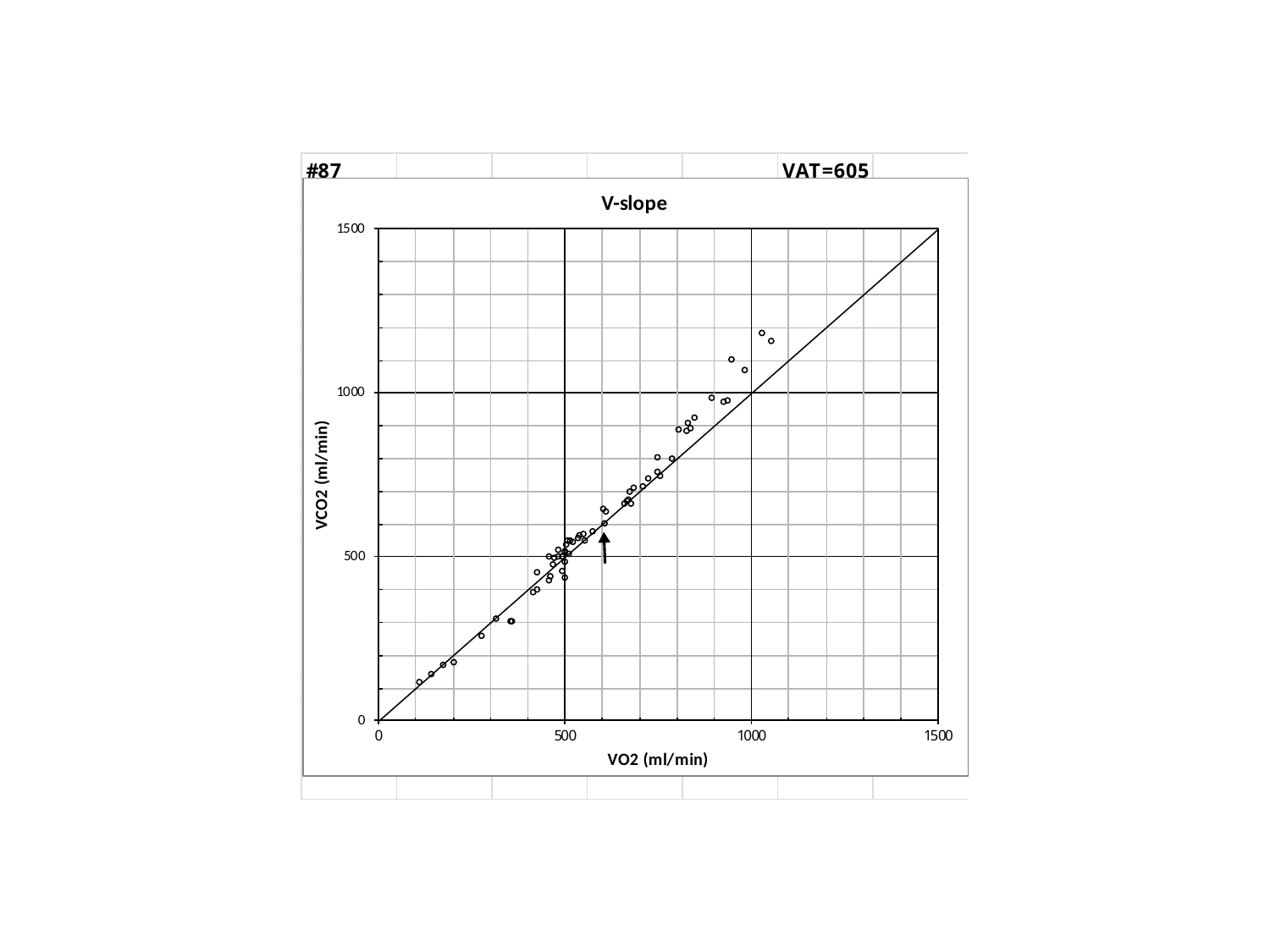

## Slide 90
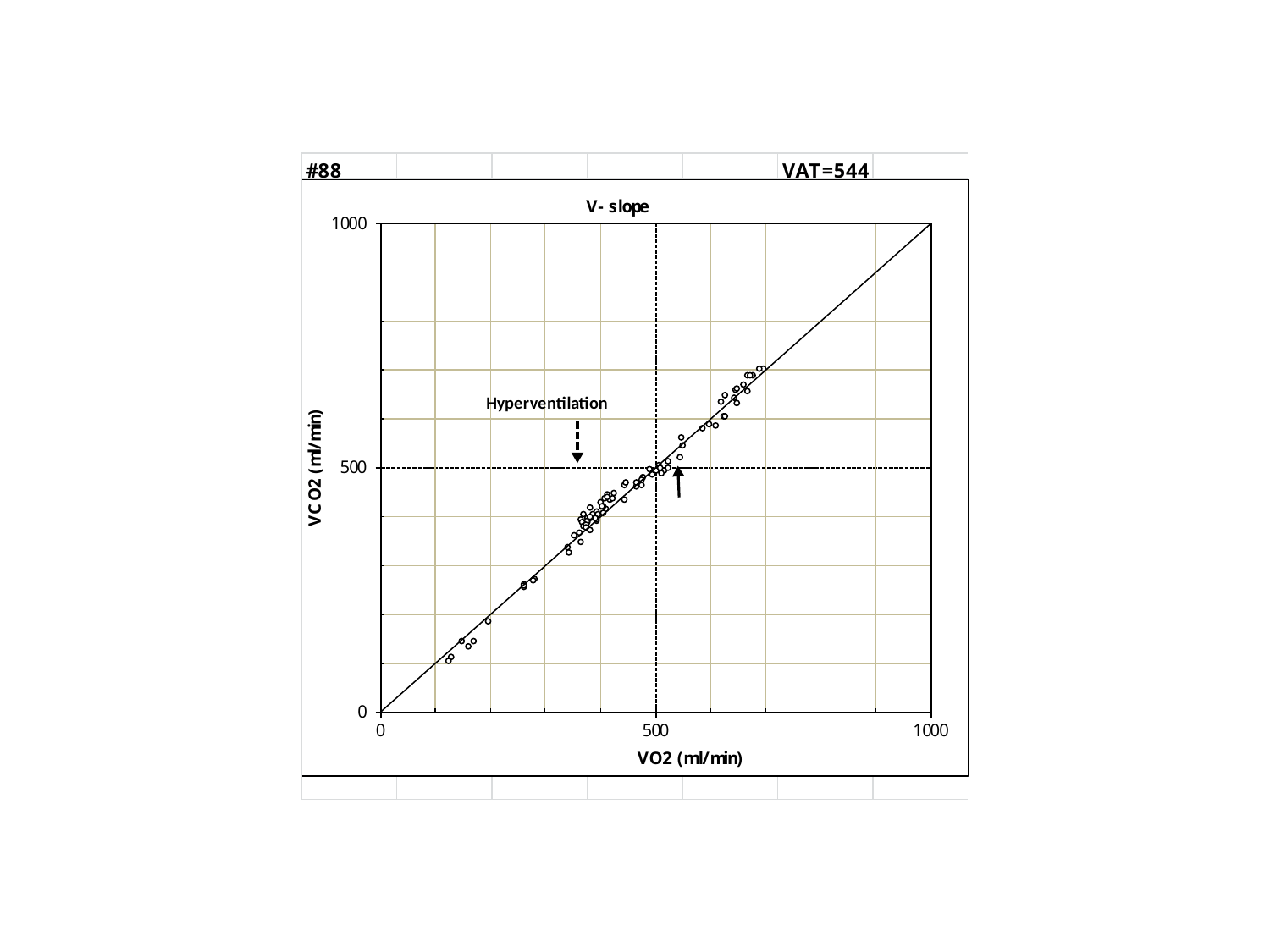

## Slide 91
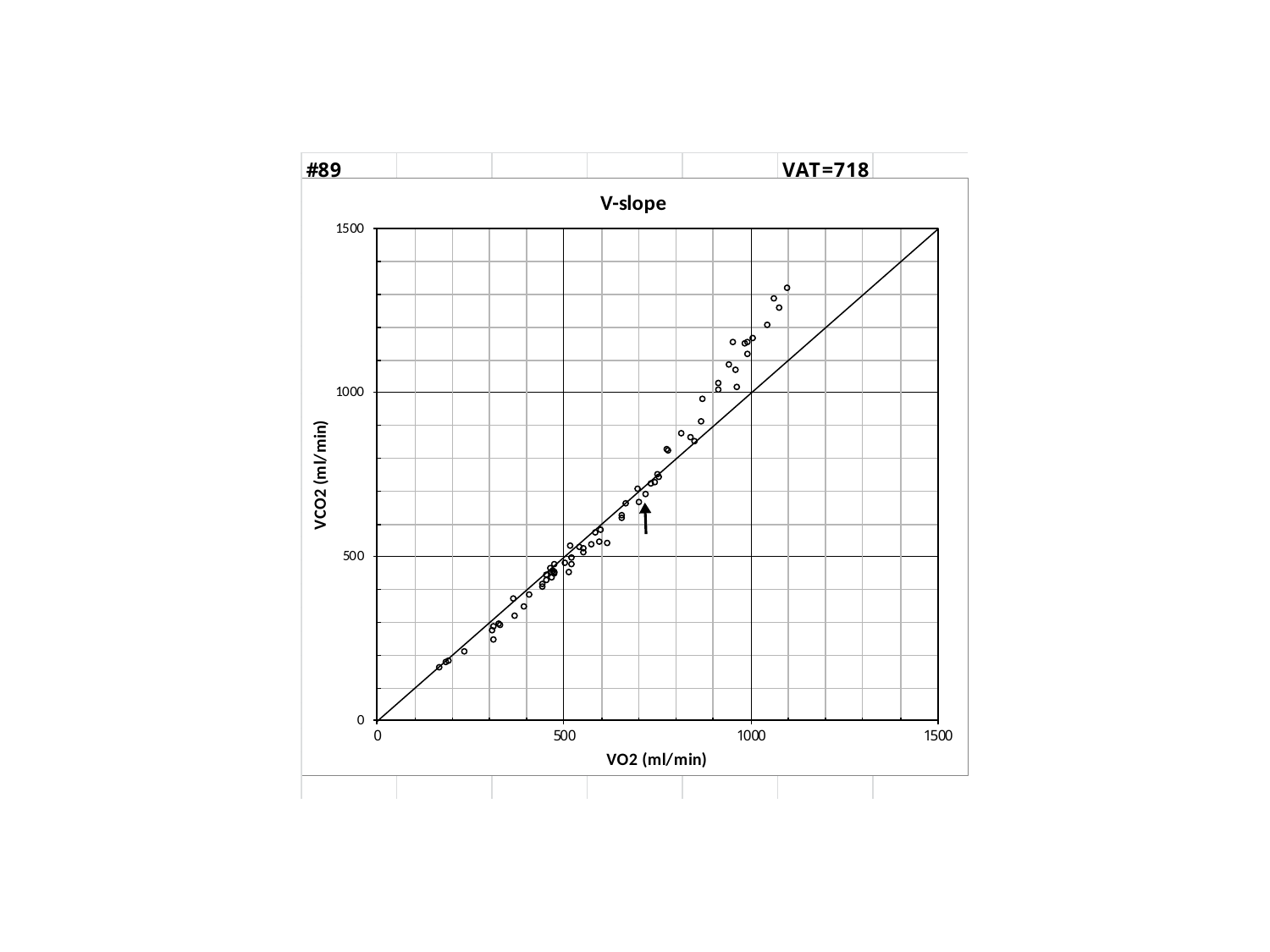

## Slide 92
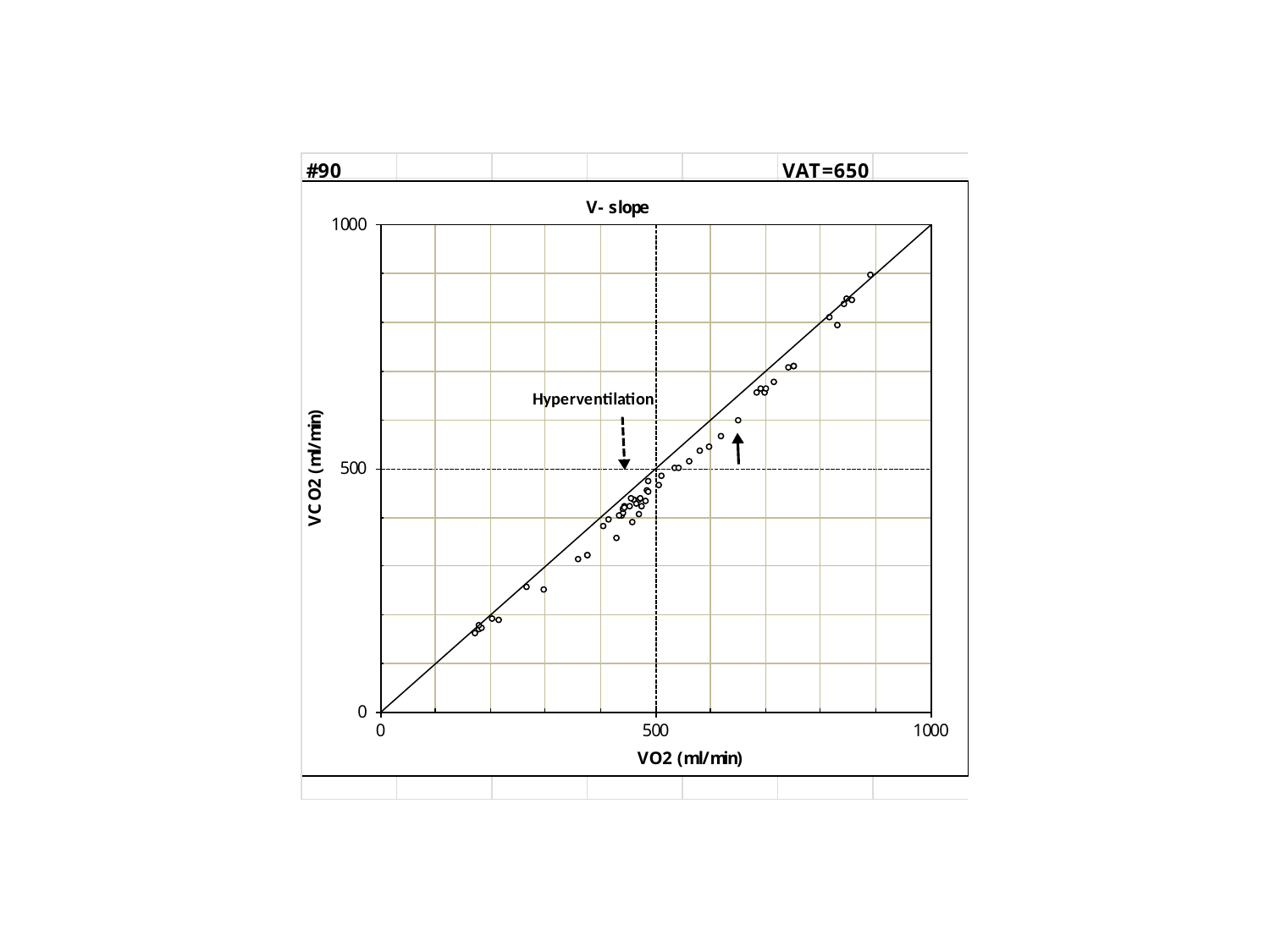

## Slide 93
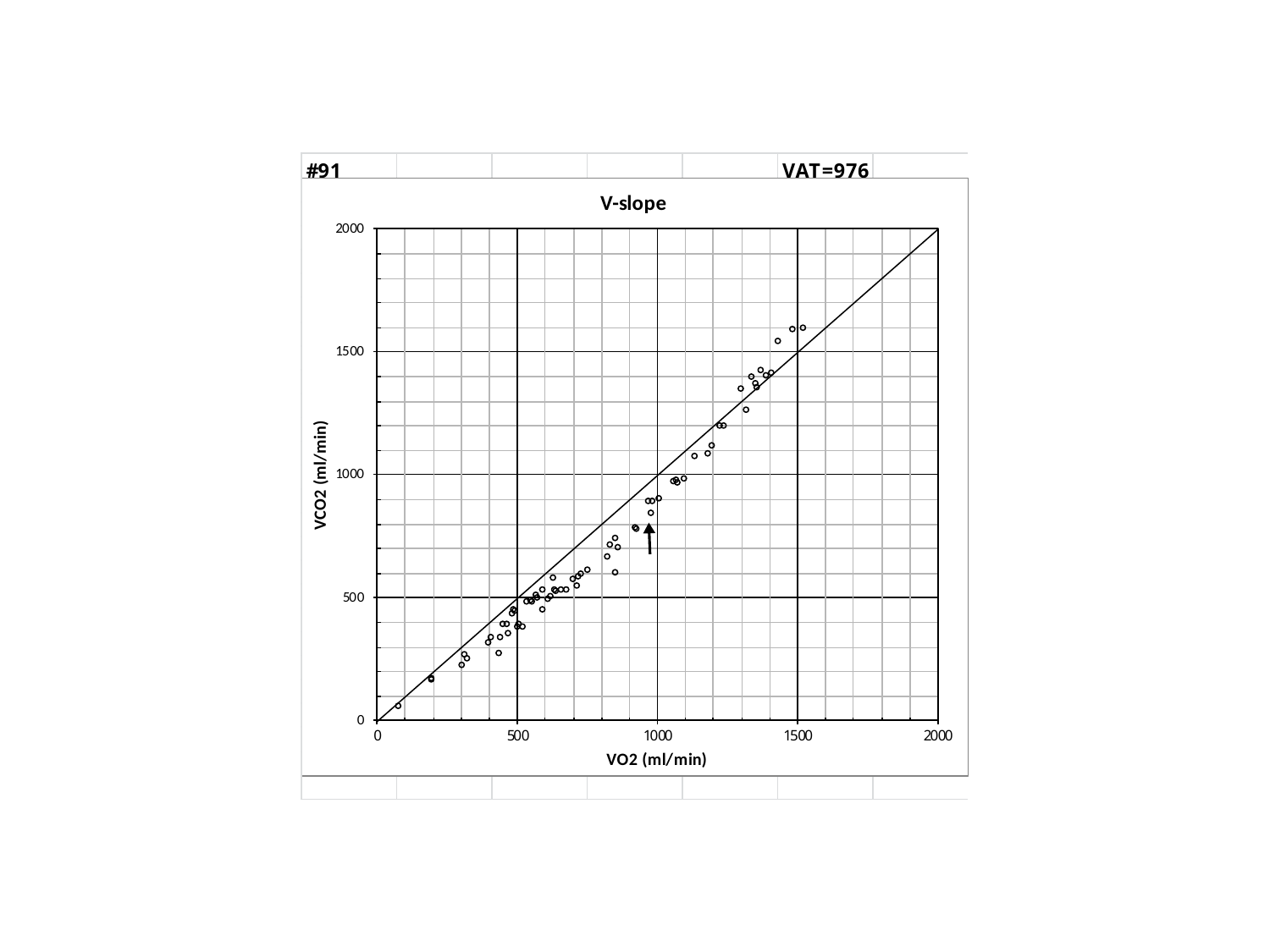

## Slide 94
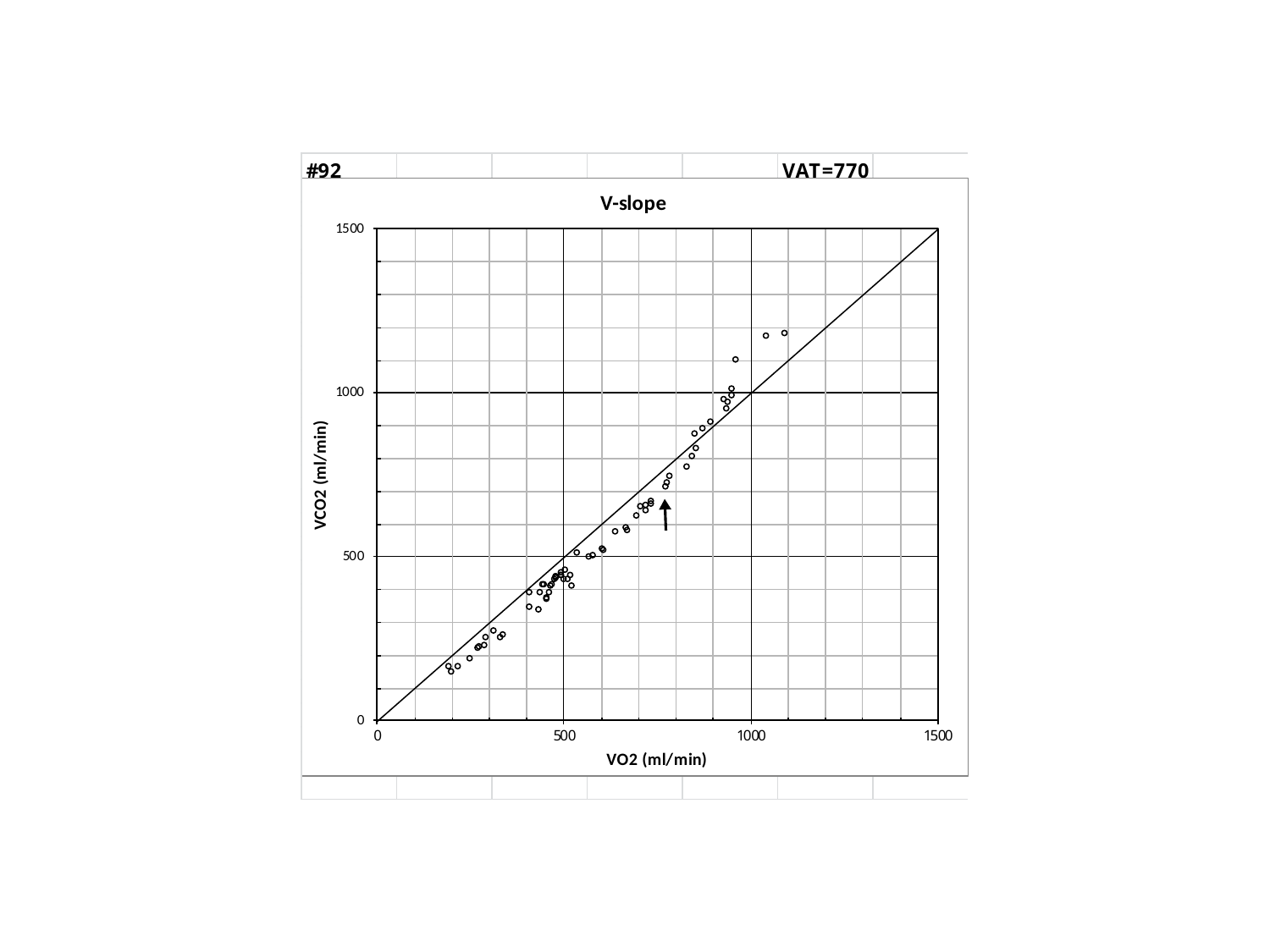

### Chart: V-slope
| Category | VCO2 |
|---|---|

## Slide 95
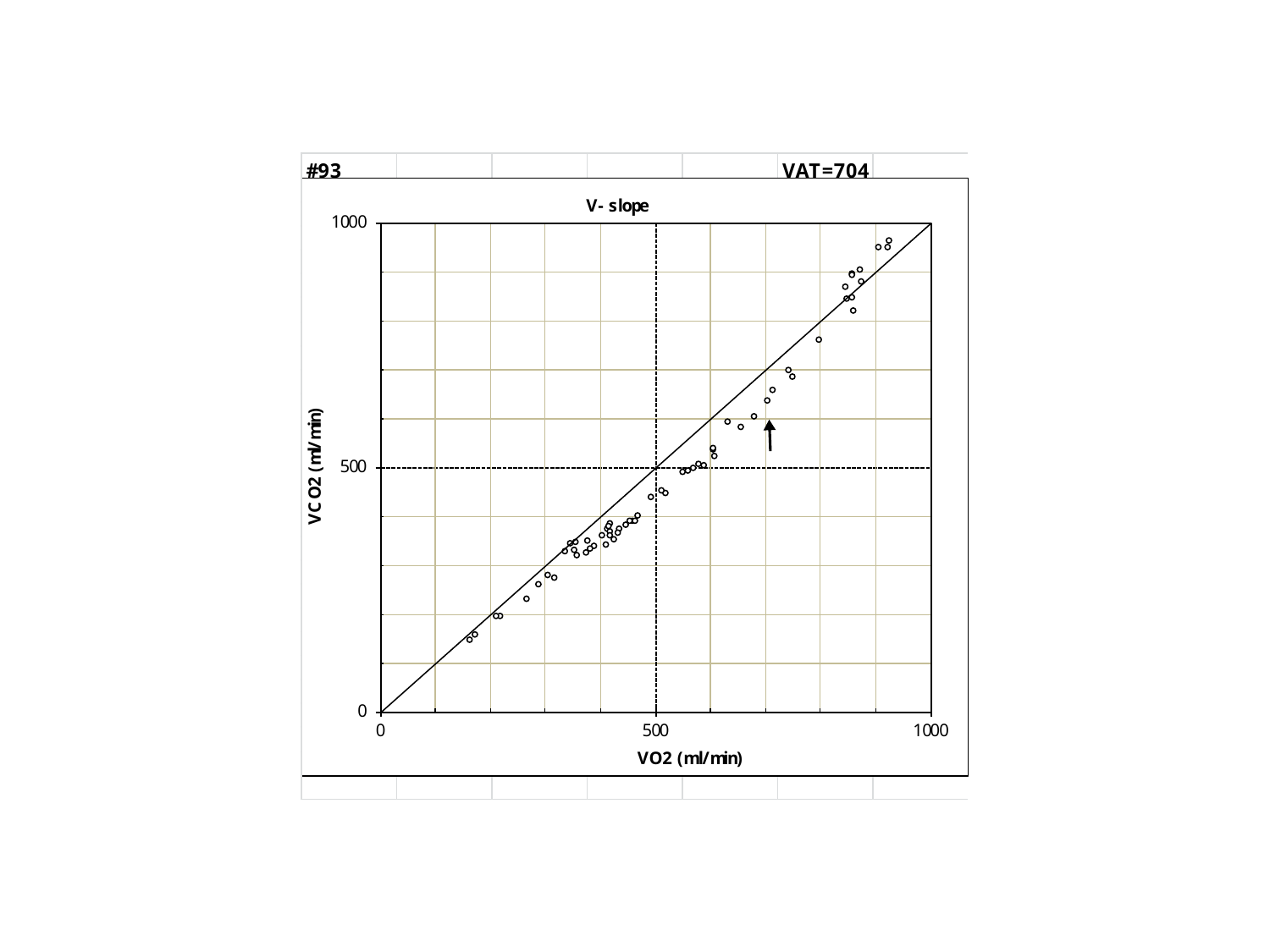

## Slide 96
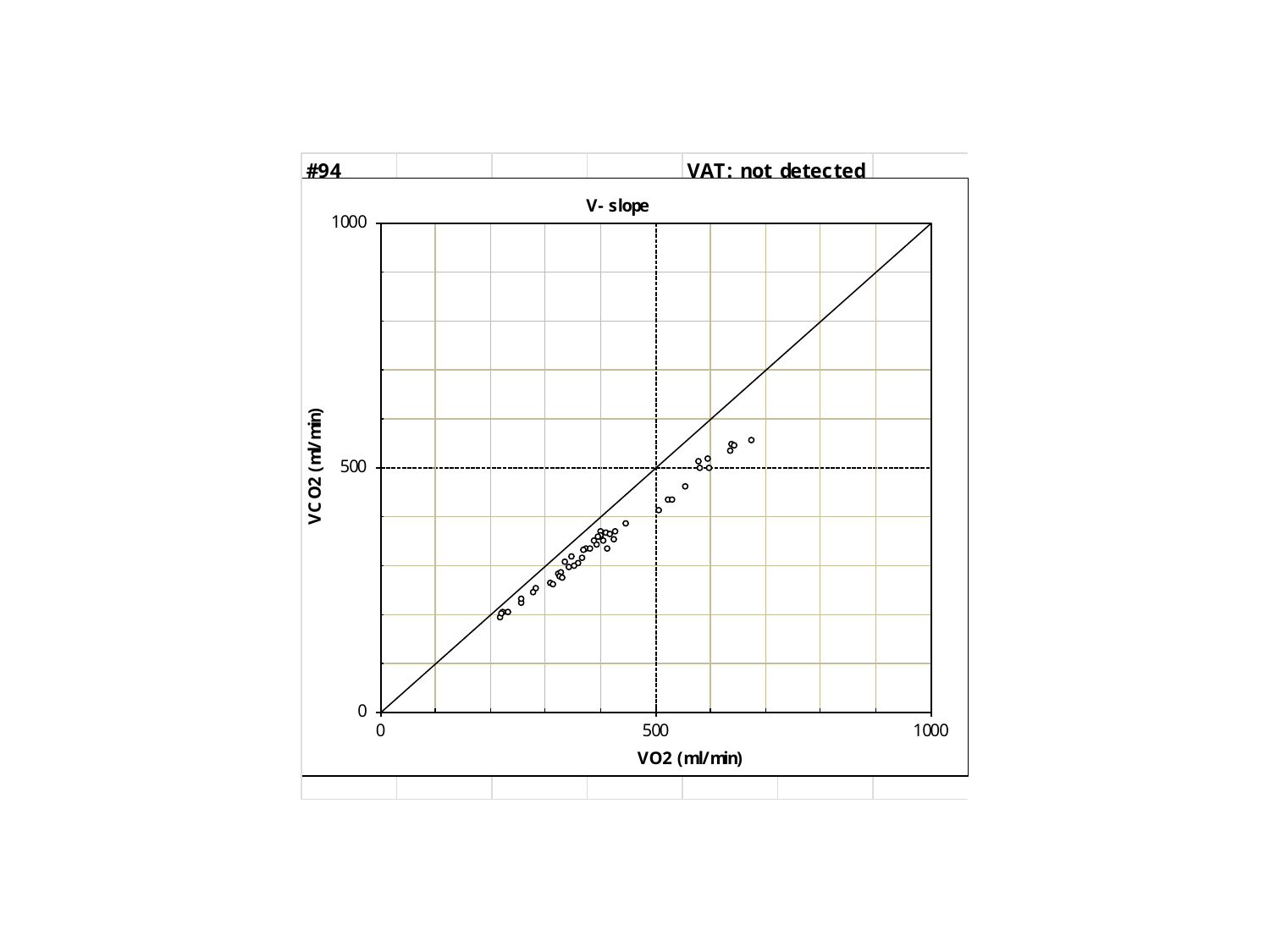

## Slide 97
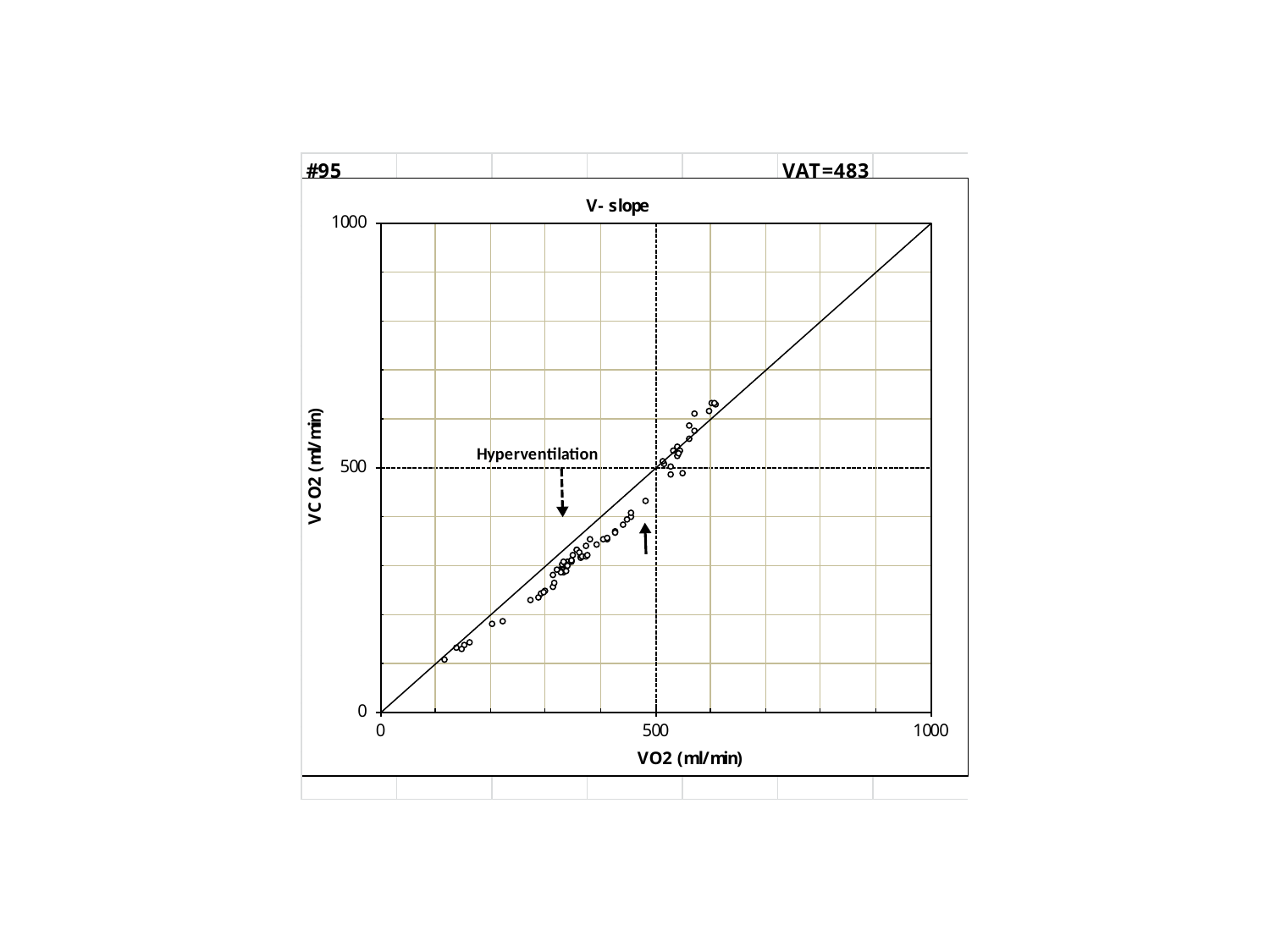

## Slide 98
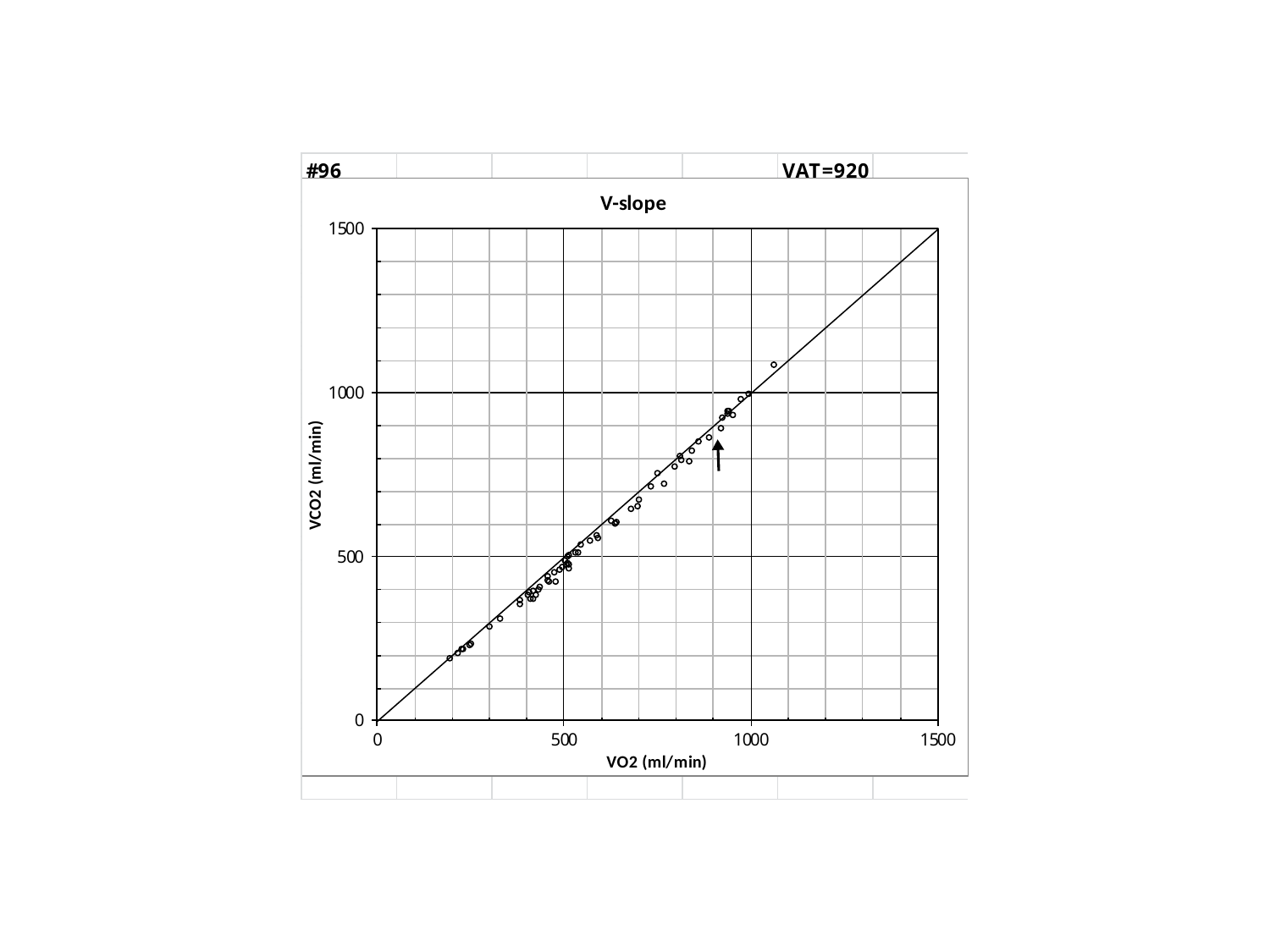

## Slide 99
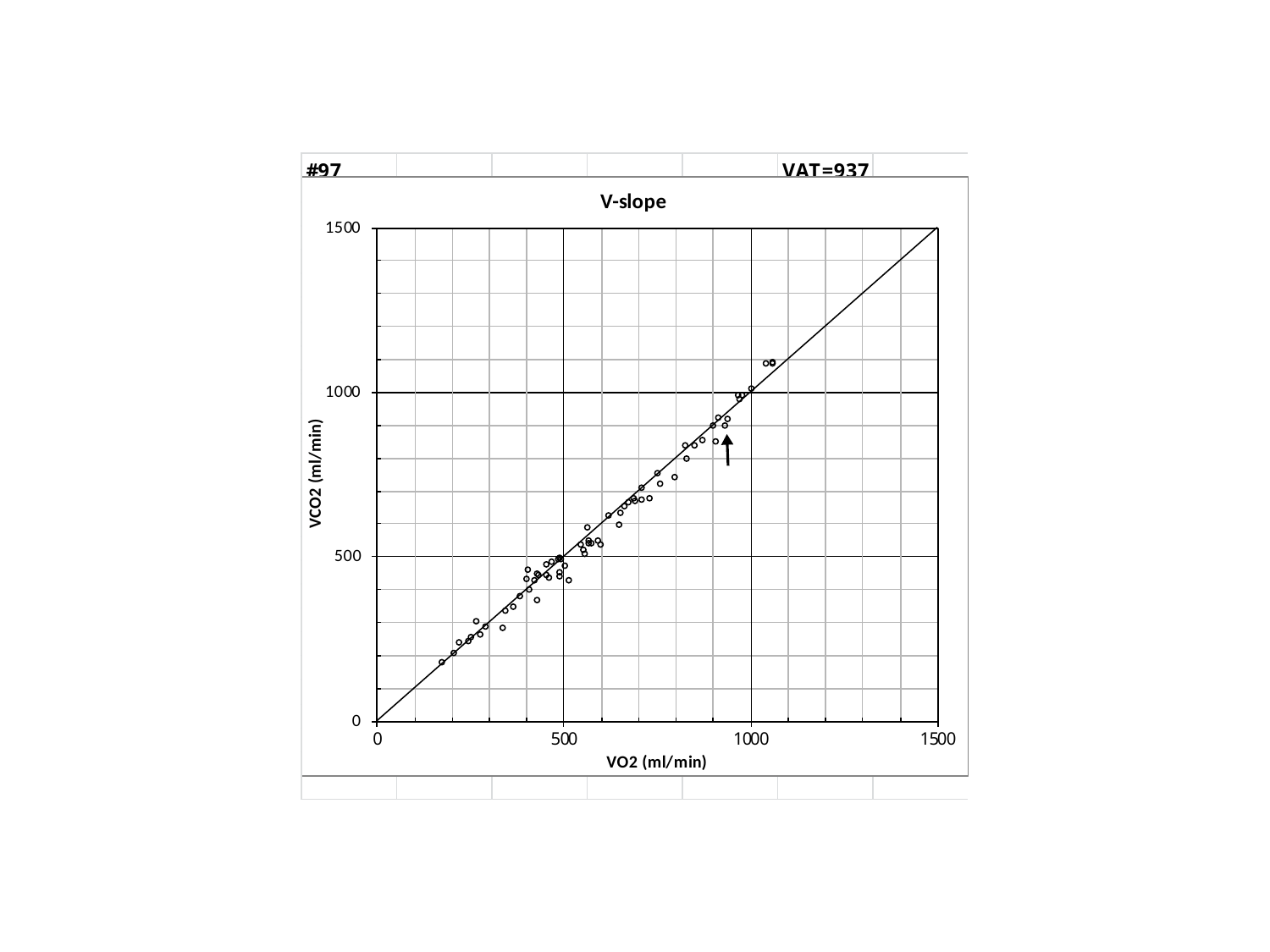

## Slide 100
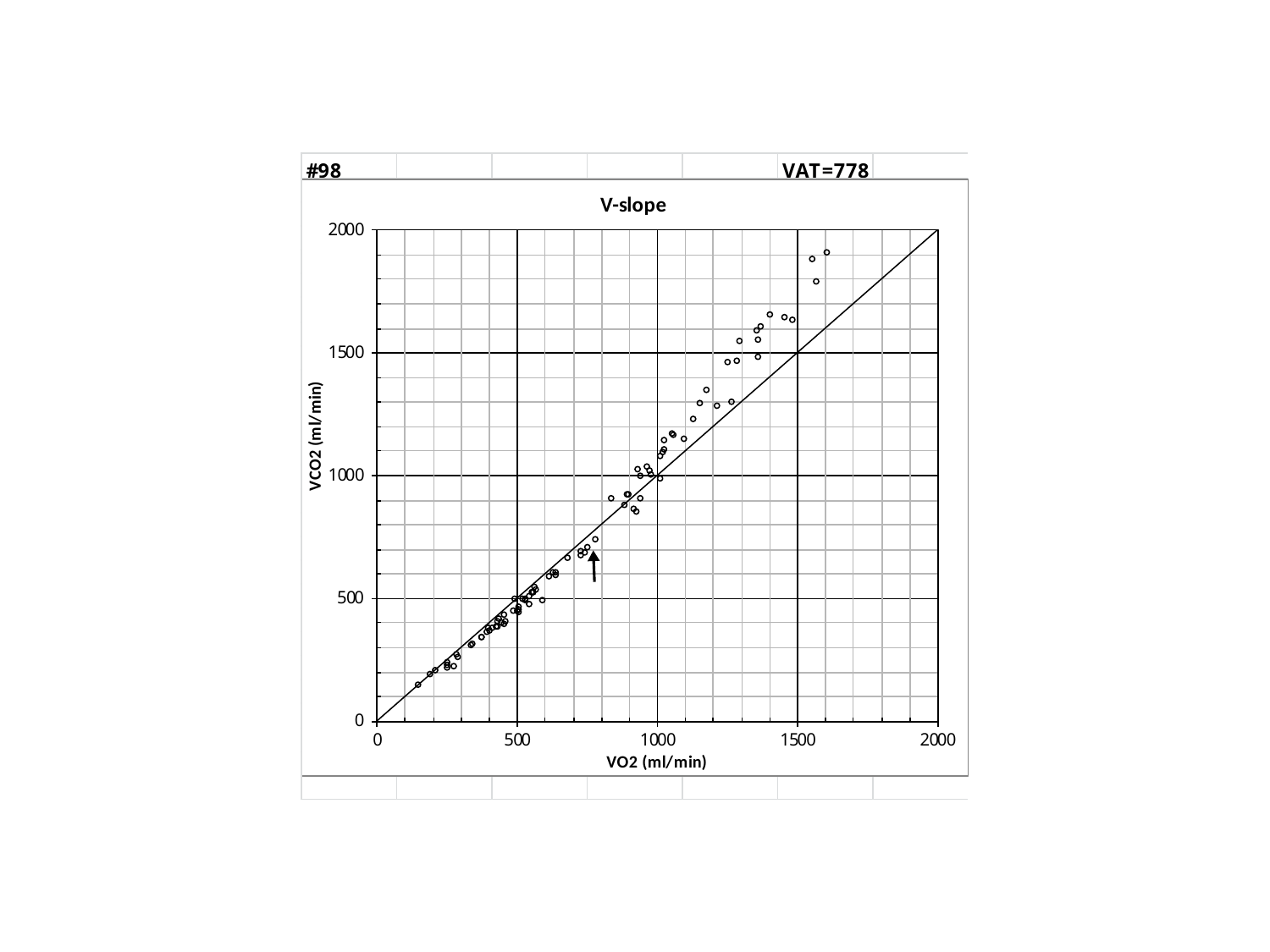

## Slide 101
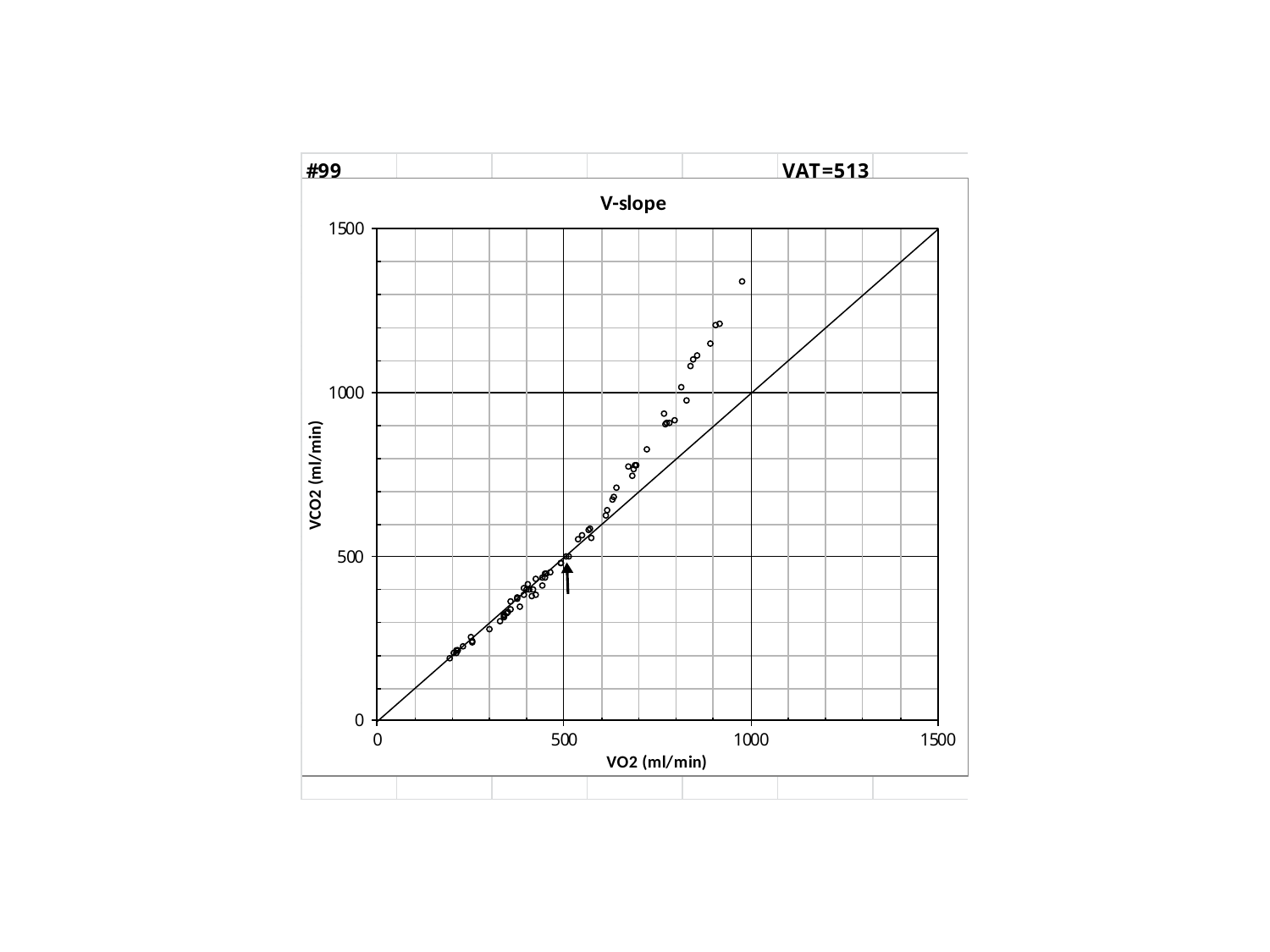

## Slide 102
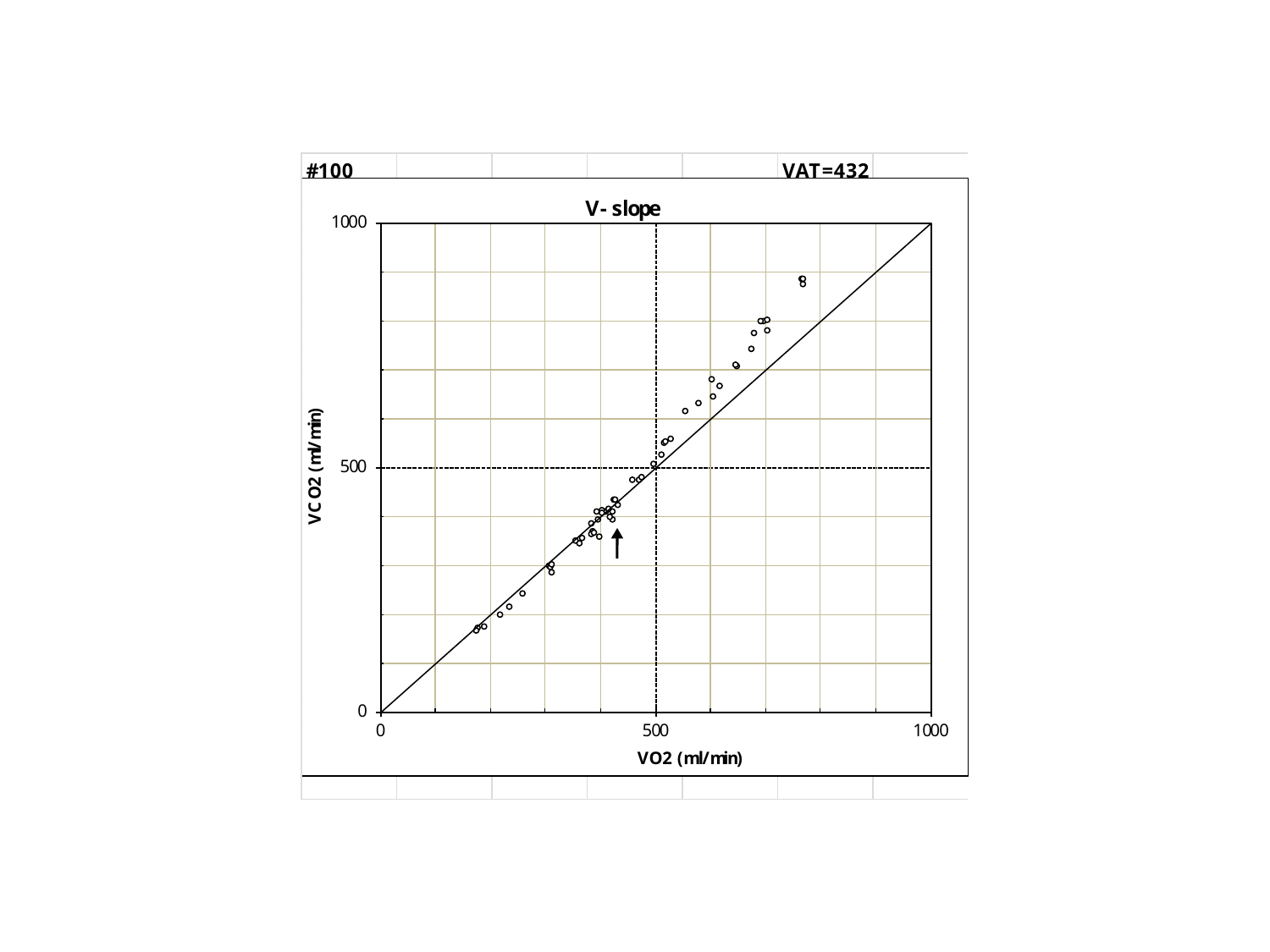

Supplement: Supplementary file 2 — Individual V-slope plots (n = 100). (PPTX 3053 kb) [file 13102_2017_73_MOESM2_ESM.pptx]
