# Supplementary material for: Quantification and physiological significance of the rightward shift of the V-slope during incremental cardiopulmonary exercise testing
Source: BMC Sports Sci Med Rehabil. 2017 Apr 20;9:9. doi: 10.1186/s13102-017-0073-1 (PMC5397810; doi:10.1186/s13102-017-0073-1)

## Slide 1
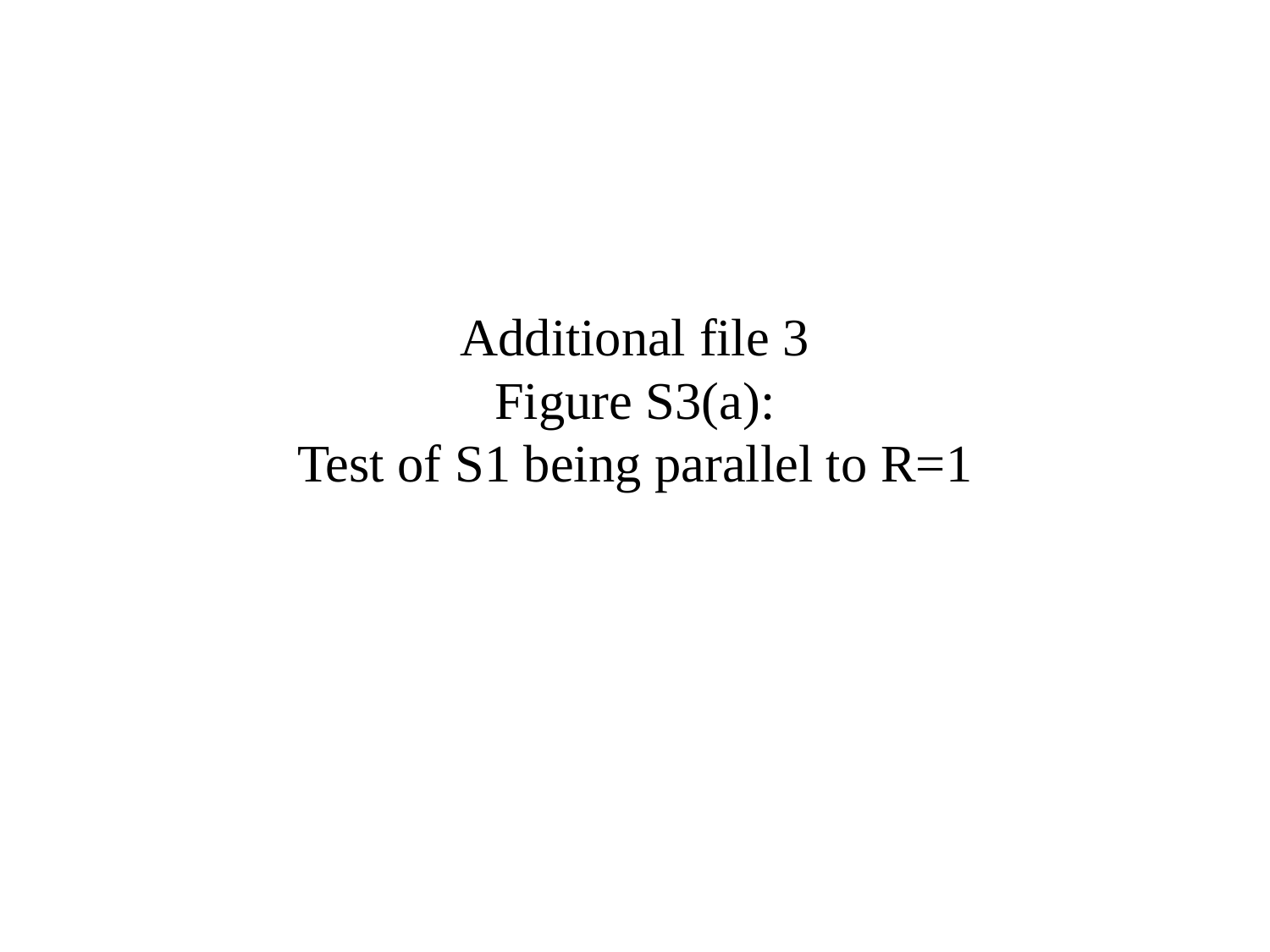

# Additional file 3 Figure S3(a): Test of S1 being parallel to R=1

## Slide 2
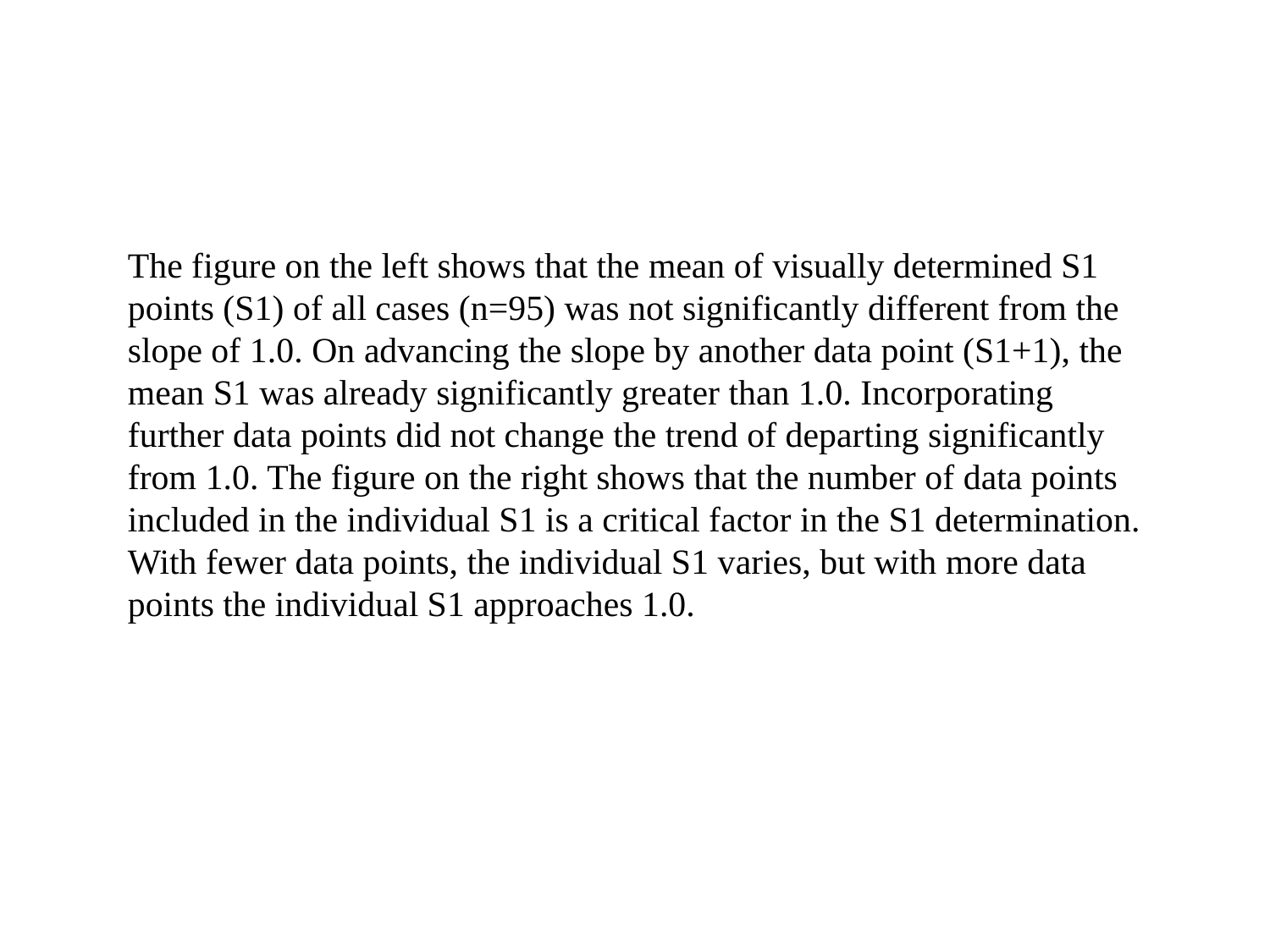

The figure on the left shows that the mean of visually determined S1 points (S1) of all cases (n=95) was not significantly different from the slope of 1.0. On advancing the slope by another data point (S1+1), the mean S1 was already significantly greater than 1.0. Incorporating further data points did not change the trend of departing significantly from 1.0. The figure on the right shows that the number of data points included in the individual S1 is a critical factor in the S1 determination. With fewer data points, the individual S1 varies, but with more data points the individual S1 approaches 1.0.

## Slide 3
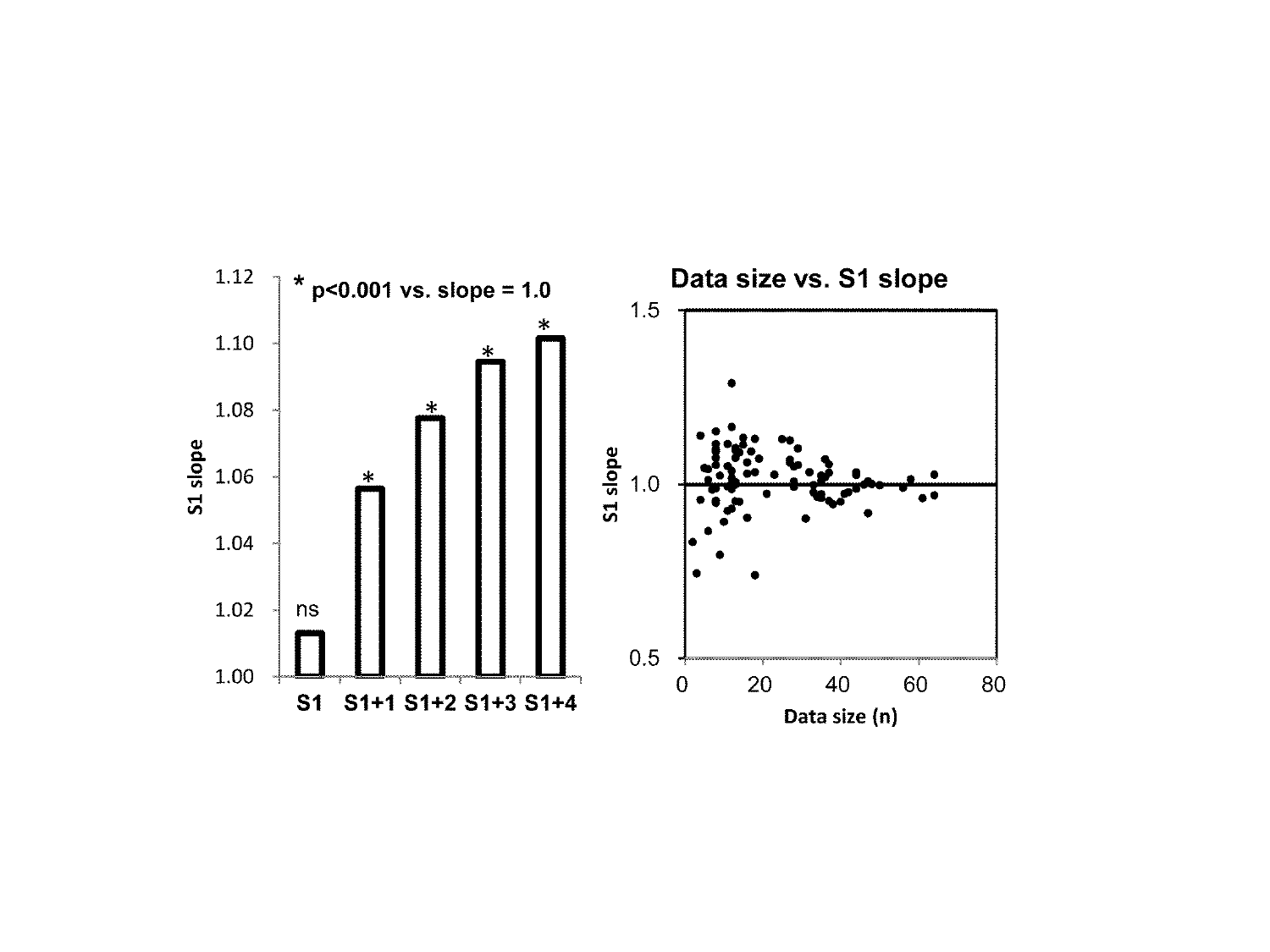

## Slide 4
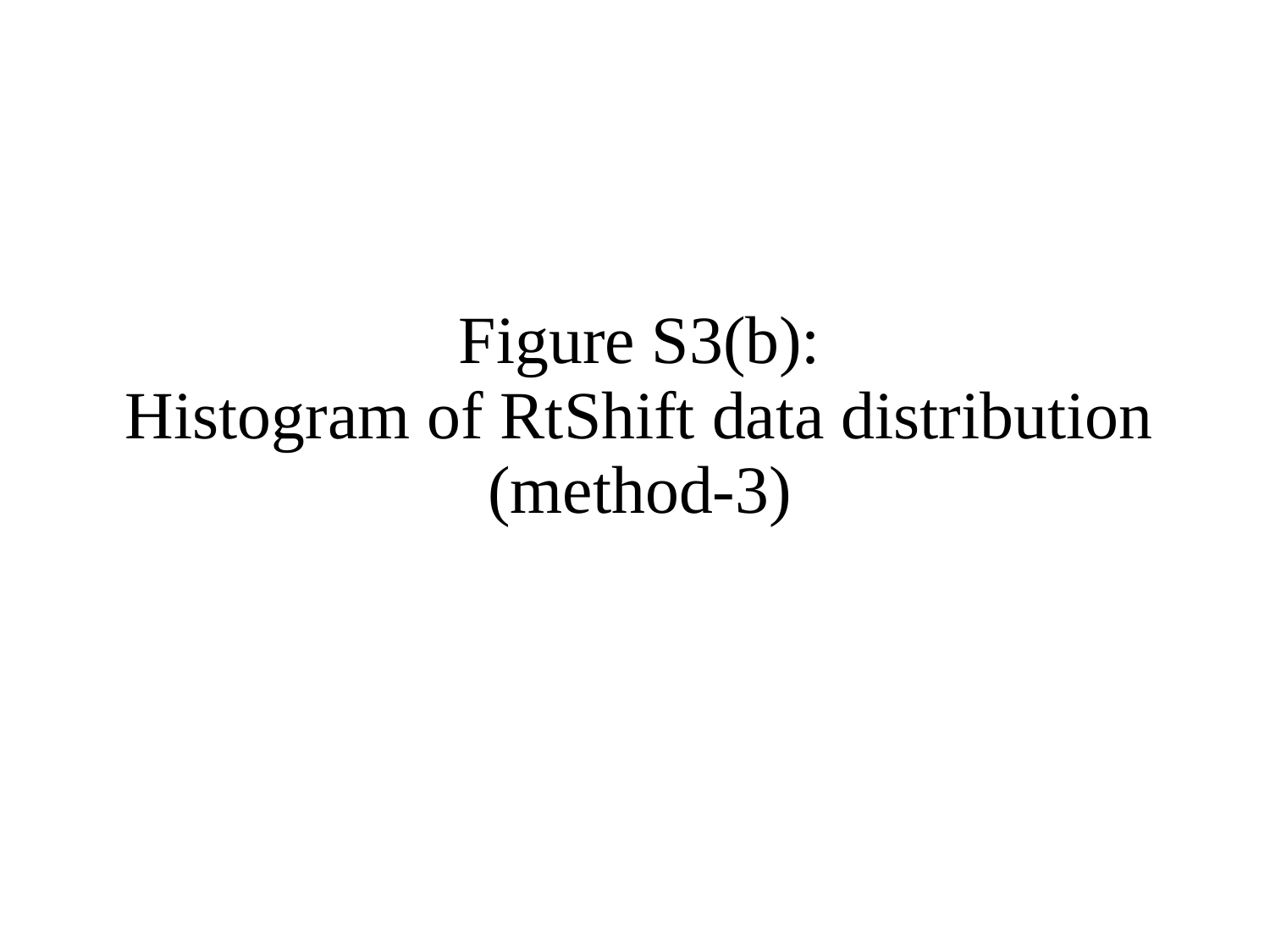

# Figure S3(b): Histogram of RtShift data distribution (method-3)

## Slide 5
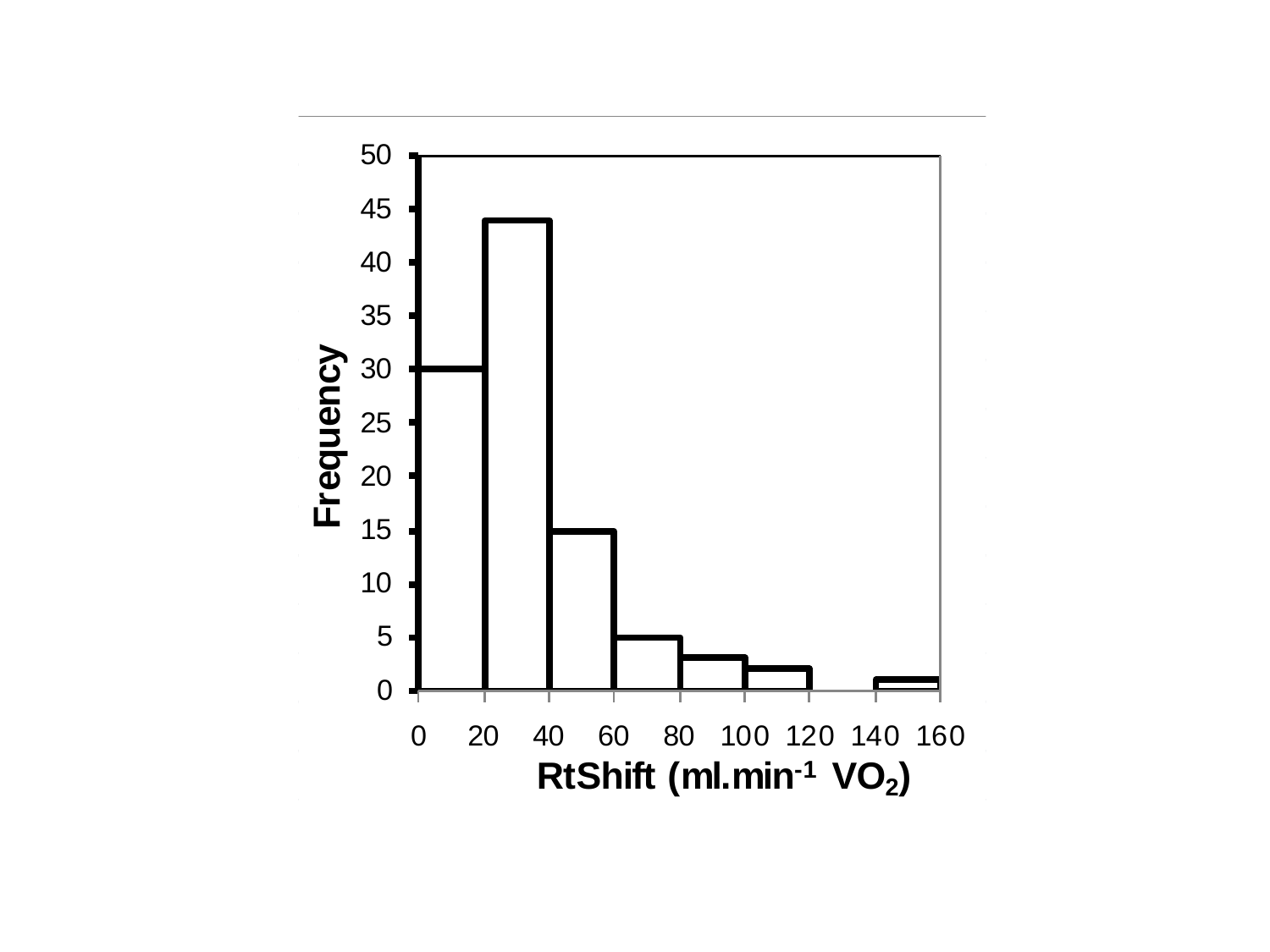

## Slide 6
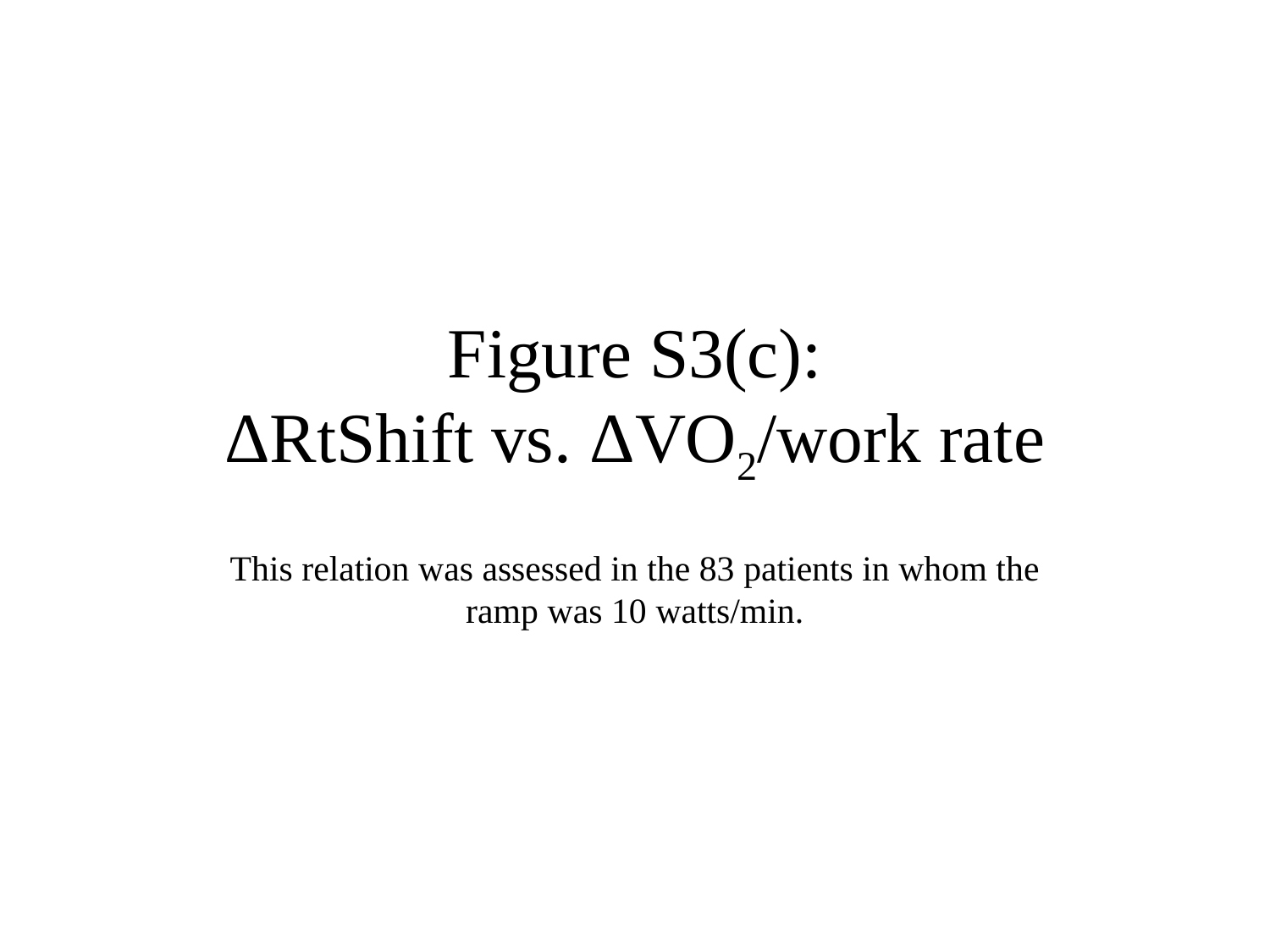

# Figure S3(c): ΔRtShift vs. ΔVO2/work rate
This relation was assessed in the 83 patients in whom the ramp was 10 watts/min.

## Slide 7
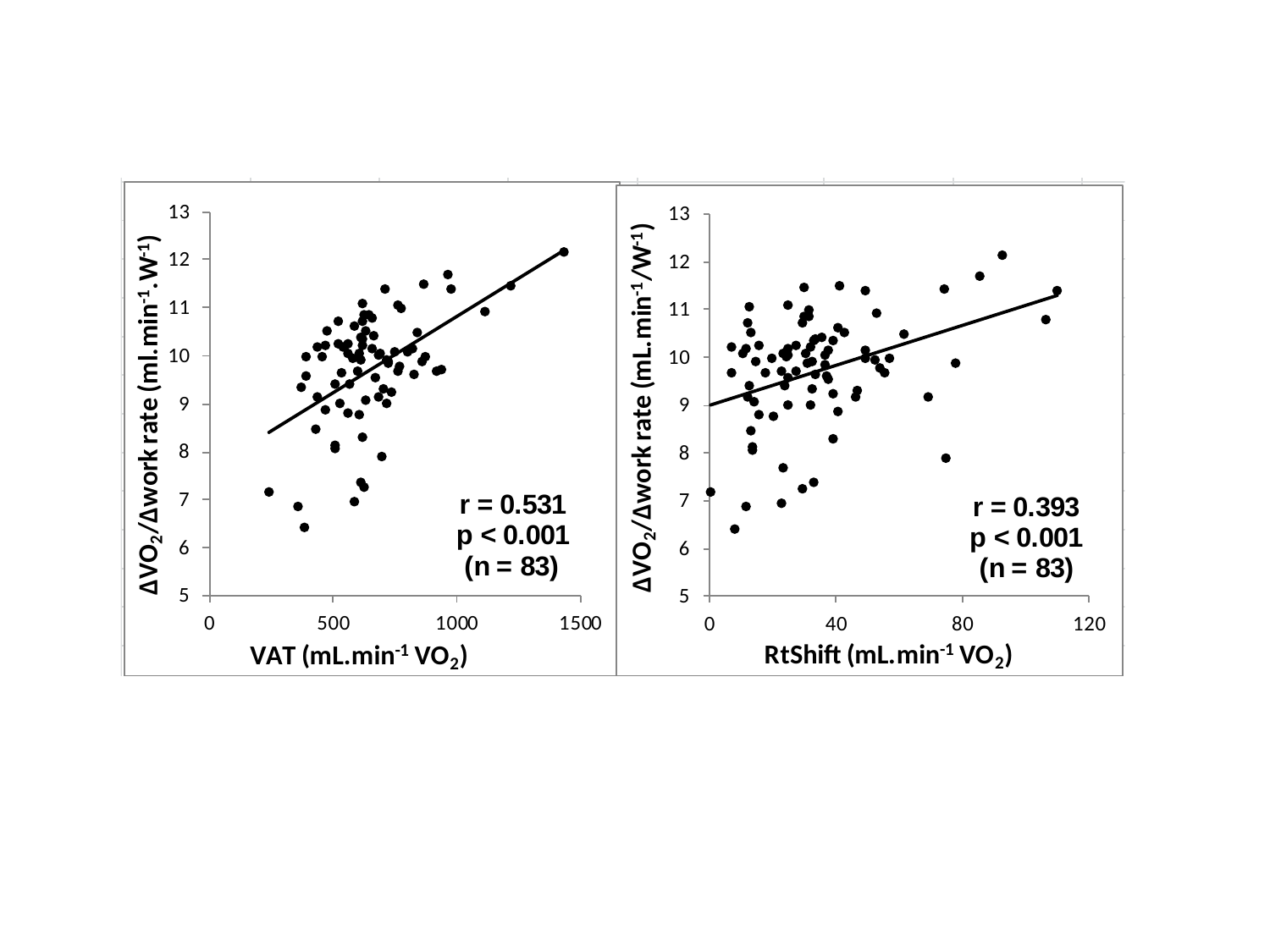

Supplement: Supplementary file 3 — (a) Histogram of RtShift data distribution. (b) RtShift versus ΔVO2/Δwork rate. (c) Test of S1 being parallel to R = 1. (PPTX 108 kb) [file 13102_2017_73_MOESM3_ESM.pptx]
