# Supplementary material for: Quantification and physiological significance of the rightward shift of the V-slope during incremental cardiopulmonary exercise testing
Source: BMC Sports Sci Med Rehabil. 2017 Apr 20;9:9. doi: 10.1186/s13102-017-0073-1 (PMC5397810; doi:10.1186/s13102-017-0073-1)
Supplement: Supplementary file 4 — Result: Results of non-parametric statistical analyses. (DOCX 13 kb) [file 13102_2017_73_MOESM4_ESM.docx]

Additional file 4

Result S4: non-parametric statistical analysis

1) Spearman’s rank order correlation

VAT (m//min) vs. RtShift (ml/min): rho = 0.653 (p <001)

VAT (ml/min/kg) vs RtShift (ml/min/kg): rho = 0.537 (p <0.001)

VAT (m//min) vs. corrected RtShift (ml/min): rho = 0.572 (p <001)

VAT (m//min) vs. ΔVO2/Δwork rate: rho = 0.411 (p <001)

VAT (ml/min/kg) vs. ΔVO2/Δwork rate rho = 0.310 (p =0.004)

Correlations among three methods to estimate RtShift

(First: visual; Second: quadratic fitting; third: simple averaging)

First vs. second: 0.929 (p <0.001)

Second vs. third: 0.990 (p <0.001)

First vs. third: 0.919 (p <0.001)

2) Friedman test

Comparisons among three methods to estimate RtShift

(First: visual; Second: quadratic fitting; third: simple averaging)

Significant at p <0.001)

Post-hoc group comparison (by Scheffe)

All significant at p <0.001

Comparison of three ramp exercise protocols (substudy)

(15 watt/min, 25 watt/min, 50 watt/min)

Not significant (p = 0.778)
